# The 11th NORDIC NUTRITION CONFERENCE NNC2016: “Bridging nutrition sciences for better health in the Nordic countries”: Gothenburg, Sweden, 20–22 June, 2016

**DOI:** 10.3402/fnr.v60.31961

**Published:** 2016-06-16

## TABLE OF CONTENTS

**I. Invited speakers presentations**

***Opening session***

101. Keynote lecture: Diet and genes: personalized nutrition for better health?, *Marju Orho-Melander*

102. Nils-Georg Asp lecture: 50 years of dietary fibre research and future perspective, *Per Åman*

***Clinical Nutrition – Pediatrics***

103. Infancy-onset dietary counseling from childhood to early adulthood – the Special Turku Coronary Risk Factor Intervention Project, *Olli Raitakari*

104. To be or not to be exposed – the role of weaning diet in development of allergy, *Agnes Wold*

105. Infant nutrition and child health – evidence for diet against disease, *Inga Thorsdottir*

***Public Health Nutrition – You are what you eat?***

106. Can we trust dietary intake data?, *Lene F. Andersen*

107. Reconciling conflicting evidence from observational studies and randomized controlled trials – exemplified by dietary protein and weight change, *Mikkel Z. Ankarfeldt*

108. Lean or fit – can exercise and diet modify health-risks of obesity?, *Mikael Fogelholm*

***Plenary Session: Vitamin D in perspective***

109. Vitamin D status in the Nordic countries in the light of methodological standardization, *Rikke Andersen*

110. Fortification as a solution for increasing vitamin D status, *Christel Lamberg-Allardt*

111. Vitamin D and bone health – anything new?, *Haakon E. Meyer*

112. Benefits gained by improving vitamin D status in relation to overdiagnosis of insufficiency, overtreatment, and potential harms?, *Karl Michaëlsson*

***Clinical Nutrition – Disease-related malnutrition***

113. Nutrition diagnosis – how dieticians identify and describe patients’ nutritional problems using standardized terminology, *Elin Lövestam*

114. Defining malnutrition – mission or mission impossible?, *Ingvar Bosaeus*

115. Nutrition and patient safety, *Randi J. Tangvik*

***Public Health Nutrition – Mother and Child***

116. School meals and health – the PROMEAL-study, *Agneta Hörnell*

117. Childhood obesity trends in the Nordic countries, *Berit L. Heitmann*

118. Maternal nutrition and child health, *Ingibjorg Gunnarsdottir*

***Plenary Session: The role of fat in health and disease***

201. Dietary fatty acids – can you divide them into the good, the bad and the ugly?, *Lars I. Hellgren*

202. Changes in fat intake and changes in cholesterol over decades - The FINRISK study, *Tiina Laatikainen*

203. Marine and vegetable sources of fatty acids – future perspective, *Ann-Sofie Sandberg*

***Clinical Nutrition – Nutrition in the elderly***

204. The obesity paradox, *Thorkild I.A. Sørensen*

205. The role of nutrition in sarcopenia and frailty, *Tommy Cederholm*

206. Nutrition in supporting brain health, *Merja Suominen*

***Public Health Nutrition – Nutrition & sustainability***

207. Healthy nutrition and sustainablility – is there a conflict?, *Helle M. Meltzer*

208. Consumer understanding of healthy nutrition and sustainability, *Elling Bere*

209. Green Public Procurement in Denmark: Organic food consumption and healthier diets, *Gregers Hummelmose and Nina N. SØrensen*

***Plenary Session: Nordic Nutrition Recommendations 2012 revisited***

210. NNR 2012 in perspective – what is the future role of nutritional recommendations?, *Ambroise Martin*

211. Nordic Nutrition Recommendations 2012 – lessons and future directions, *Jessica Ahlin*

***Clinical Nutrition – Fighting obesity in society***

212. The role of individual treatment strategies, *Ingrid Larsson*

213. Environmental prevention and treatment strategies, *Knut-Inge Klepp*

214. The role of food companies – barriers or facilitaters?, *Mette Peetz-Schou*

***Public Health Nutrition – Nutrition for healthy ageing***

215. The changing scene of ageing – to become old yesterday, today and tomorrow, *Elisabet Rothenberg*

216. Nutritional status for healthy ageing, *Agnes N. Pedersen*

217. Overweight and obesity – the pros and cons of targeting weight reduction in the overweight and obese elderly, *Anne M. Beck*

***Clinical Nutrition – Future challenges***

301. FODMAP versus gluten sensitivity – do we have clear picture?, *Knut E.A. Lundin*

302. Nutrition, gut microbiota, and health – where do we stand?, *Erika Isolauri*

303. Computational models of energy balance, *Ola Wallengren*

304. Iodine deficiency – a ghost from the past revisiting us, *Lisbeth Dahl*

***Public Health Nutrition – Future challenges***

305. Exploring eating behavior in a changing society – challenges and opportunities, *Eva Roos*

306. Biomarkers for dietary intake-where do we stand? – where do we stand?, *Rikard Landberg*

307. Public health nutrition education, *Liv E. Torheim and Bryndís E. Birgisdottir*

308. A Nordic Food Composition Database – a potential goal for the future?, *Liisa Valsta*

***Plenary Session: Dietary patterns and health – what do we really know?***

309. Recent advances in research into a Healthy Nordic dietary pattern, *Matti Uusitupa*

310. Challenges involved in studying a traditional Sami diet pattern as a determinant of health in modern time, *Lena M. Nilsson*

311. Nutrients versus dietary patterns in nutrition epidemiology – methodological considerations, *Isabel Drake*

**II. Oral presentations**

***Clinical Nutrition – Pediatrics***

O101: Dairy and fish product intake and plasma phospholipid acids in young children, *Nicolai A. Lund-Blix*

O102: Dietary total antioxidant capacity in early school age and subsequent allergic disease, *Anna Bergström*

***Public Health Nutrition – You are what you eat?***

O103: Dietary habits in adolescence and midlife and risk of breast cancer, *Alfheidur Haraldsdottir*

O104: Dietary intakes and gut microbiota composition in a Swedish population, *Louise Brunkwall*

***Clinical Nutrition – Disease-related malnutrition***

O105: Unsatisfactory knowledge and use of terminology regarding malnutrition, starvation, cachexia, and sarcopenia among dietitians in Belgium, the Netherlands, Norway, and Sweden, *Lies ter Beek*

O106: Association between weight change and mortality in community living older people followed for up to 14 years: the Hordaland Health Study (HUSK), *Teresa R. Haugsgjerd*

***Public Health Nutrition – Mother and Child***

O107: Iodine intake in pregnancy is associated with neurodevelopment at age 3: results from The Norwegian Birth Cohort Study (MoBa), *Marianne H. Abel*

O108: Effects of a 2-year individualized and family-based lifestyle intervention on physical activity and diet quality in children, *Aino-Maija Eloranta*

***Clinical Nutrition – Nutrition in the elderly***

O201: Milk intake in middle-aged Norwegians and risk of hip fracture: is there an association? A linkage between the Norwegian Counties Study and the NOREPOS hip fracture database, *Kristin Holvik*

O202: Vitamin D does not predict dementia or cognitive impairment – a 20-year follow-up study in community living old men, *Erika K.E. Olsson*

***Public Health Nutrition – Nutrition & sustainability***

O203: Replacing meat with fish in our diet – are there metabolic consequences beyond n-3 fatty acids?, *Alastair Ross*

O204: Risk of myocardial infarction – impact of dietary exposure to persistent organic pollutants and intake of long-chain fish fatty acids: results from population-based prospective cohorts, *Agneta Åkesson*

***Clinical Nutrition – Fighting obesity in society***

O205: Metabolic outcomes after an 8-week low-calorie-diet in overweight, pre-diabetic individuals: the role of gender in the PREVIEW study, *Anne Raben*

O206: Healthy Nordic diet and obesity: results from a 7-year follow-up in adult Finns, *Noora Kanerva*

***Public Health Nutrition – Nutrition for healthy ageing***

O207: Dairy intake and mortality in Northern Sweden, *Gianluca Tognon*

O208: Adherence to dietary intervention among independently living individuals with elevated risk of dementia: Finnish Geriatric Intervention Study to Prevent Cognitive Impairment and Disability (FINGER), *Jenni Lehtisalo*

**III. Poster presentations**

P101-P128, P201-P211, P301-313, P401-P501

## I. Invited speakers presentations

### Program no. 101

#### Diet and genes: personalized nutrition for better health?

##### Marju Orho-Melander* Clinical Sciences in Malmö, Lund University, Malmö, Sweden
*Presenting author

Cardiovascular disease (CVD), type 2 diabetes (T2D), and obesity, collectively referred to as cardiometabolic diseases, together with cancer are the major morbidities and causes of death, and the risk of these diseases and mortality are all known to be heavily affected by diet. With few exceptions, research on cardiometabolic disease and cancer is funded, studied, and clinically applied separately without fully taking advantage of knowledge on common pathways and treatment targets through interdisciplinary synergies. My research group focuses on connections between diet and health, and on how our genetic factors may modify such connections i.e. we investigate the role of interactions between diet and genes in cardiometabolic diseases. Further, we investigate connections between cardiometabolic diseases and traits (among others BMI, blood lipids, glucose), and common cancer forms. Using genetic tools such as Mendelian randomization, we investigate causal factors connecting and disconnecting cardiometabolic traits and common disease endpoints as type 2 diabetes, coronary disease, cancer, and mortality. More recently, we have initiated a study investigating how dietary factors and genetic factors affect gut microbiota composition and will examine gut microbiome as a potential mediator of such interactions. Further, we have recently identified circulating biomarkers that predict both cardiometabolic and cancer endpoints, and that are regulated by dietary and genetic factors. My lecture will highlight some of our most recent results and other related studies, challenging the question if understanding of interactions between diet, genes, circulating biomarkers, and gut microbiome, and knowledge of connections between cardiometabolic diseases and cancer can lead us to more personalized nutrition advice and better health.

**Disclosure of interest**: None to declare.

### Program no. 102

#### Nils-Georg Asp lecture: 50 years of dietary fiber research and future perspective

##### Per Åman* Food Science, Swedish University of Agricultural Sciences, Uppsala, Sweden 
*Presenting author

Since ancient times, it has been known that consumption of unrefined cereal foods, rich in crude materials, is good for our health, but at that time, little was known about the structure and properties of these fibrous materials. More modern research begun in the late 1960s when definitions and analytical methods of dietary fiber were discussed. However, it was not until recently that the term dietary fiber was defined in Europe. The broad European definition, including resistant oligosaccharides, resistant starch, non-starch polysaccharides, as well as associated non-carbohydrate components, is similar to the international CODEX definition with the exception that the inclusion of resistant oligosaccharides is optional in the latter. Investigations in the 60s and 70s showed that a high intake of dietary fiber was associated with lower incidence of Western diseases, and the fiber hypothesis was formulated. Modern studies including both epidemiological and intervention investigations have confirmed that intake of dietary fiber can be good for our health, and the official recommendations today encourage consumers to eat more dietary fiber. However, physiological effects of dietary fiber vary depending on the type of fiber consumed, and the European Food Safety Authority (EFSA) did not find “dietary fiber” sufficiently characterized in relation to claimed health effects. In Europe, the terms total or soluble dietary fiber is thus considered to be too broad and imprecise to form the basis for health claims. In future, we can therefore expect health studies on more precisely defined and characterized dietary fiber components. In this presentation I will review some early research on dietary fiber, describe the current status, and give some future perspectives.

**Disclosure of interest**: None to declare.

### Program no. 103

#### Infancy-onset dietary counseling from childhood to early adulthood – the Special Turku Coronary Risk Factor Intervention Project

##### Olli Raitakari* The Research Centre of Applied and Preventive Cardiovascular Medicine, University of Turku, Turku, Finland 
*Presenting author

The Special Turku Coronary Risk Factor Intervention Project (STRIP) was launched to study the effect of dietary intervention initiated in infancy and maintained until the age of 20 years on atherosclerosis risk factors.

The study recruited families with 5-month-old infants at well-baby clinics in Turku, Finland, between February 1990 and June 1992. At the age of 6 months, 1,062 infants (56.5% of the eligible age cohort) were randomly allocated to an intervention (*n*=540) or a control (*n*=522) group. The intervention group received individualized dietary counseling at least biannually until the age of 20 years. The main target of the counseling was to replace saturated fat with unsaturated fat in the child's diet (reduction in total fat intake was not targeted). The children in the intervention group also received counseling on, for example, how to reduce salt intake and to favor whole-grain products, fruits, and vegetables. Use of whole-grain products was encouraged to increase fiber intake and to introduce better-quality carbohydrates to the diet. Counseling on fiber and, for example, quality of cereals was given repeatedly during the study. In terms of protein, specific counseling related to plant- or animal-based sources was not given. The counseling was given to the parents until the child was 7 years old, and from then onward, gradually more information was given directly to the child. A fixed diet was never ordered; the counseling was individualized, and the child's recent food record was used as a basis of suggestions for dietary changes. The dietary recommendations were based on Nordic nutrition recommendations (30% of energy from fat, 10–15% of energy from protein, and 50–60% of energy from carbohydrates).

The personalized dietary counseling was safe for the children's growth and development, and led to lower low-density lipoprotein cholesterol concentrations and blood pressure in the intervention group, improved insulin sensitivity, increased ideal cardiovascular health, and reduced the risk of metabolic syndrome.

Supervised dietary counseling of a low saturated fat diet given in the STRIP-project effectively decreases exposure to cardiovascular risk factors without affecting growth and development of healthy children and adolescents.

**Disclosure of interest**: None to declare.

### Program no. 104

#### To be or not to be exposed – the role of weaning diet in development of allergy

##### Agnes Wold* Department of Infectious Diseases, University of Gothenburg, Gothenburg, Sweden 
*Presenting author

Abstract not submitted.

### Program no. 105

#### Infant nutrition and child health – evidence for diet against disease

##### Inga Thorsdottir^1^*, Agneta Hörnell^2^, Britt Lande^3^ and Hanna Lagström^4^
^1^School of Health Sciences, University of Iceland, Reykjavik, Iceland; ^2^Department of Food and Nutrition, Umeå University, Umeå, Sweden; ^3^Division of Public Health, Norwegian Directorate of Health, Oslo, Norway; ^4^Turku Institute for Child and Youth Research, University of Turku, Turku, Finland 
*Presenting author


Parents are advised to breastfeed their babies. Increased knowledge has strengthened the evidence for this recommendation. The results of relevant systematic literature reviews (SLRs) 2013 for the Nordic nutrition recommendations will be reviewed, and possible new evidence will be discussed. According to the SLRs, there is convincing evidence for a protective effect of breastfeeding against overweight and obesity in childhood and adolescence, overall infections, acute otitis media, and gastrointestinal and respiratory tract infections. There was probable evidence that breastfeeding is a protective factor against inflammatory bowel disease, celiac disease, and diabetes (type 1 and 2), provides beneficial effects on IQ and developmental scores of children as well as a small reductive effect on blood pressure and blood cholesterol levels in adulthood. Higher protein intake in infancy and early childhood is convincingly associated with increased growth and higher BMI in childhood. Protein intake between 15 E% and 20 E% in early childhood has been associated with an increased risk of being overweight later in life. Too little is known about infant diet and cancer, allergy and food intolerance. Studies on allergy and intolerance have indicated that early introduction of a variety of other foods into the infant's diet while also giving breastmilk may be protective. Breastmilk is the optimal infant diet and official recommendations worldwide have followed the WHO's recommendation on exclusive breastfeeding for the infant's first six months and continued breastfeeding parallel to giving other food until one or two year of age or as long as it suits the family.

**Disclosure of interest**: None to declare.

### Program no. 106

#### Can we trust dietary intake data?

##### Lene F. Andersen* Department of Nutrition, University of Oslo, Oslo, Norway 
*Presenting author

Accurate estimates of food intake are necessary for nutrition surveillance, research, and clinical practice. It is important to identify populations at risk, as well as those who are meeting the recommendations. Moreover, knowledge about intake should guide health policies. However, are self-reported dietary data good enough (valid and reliable) for use in nutrition science and to guide health policies?

Most of today's nutrition studies rely on participants memory and their capability to report what they have been eaten and how much. But it's difficult to remember everything eaten, even if it was only yesterday. And portion sizes may be an even more challenging one. Studies have shown that people underreport their true energy intake with about 30%, with a range of 10–45% depending on such factors as age, sex, body composition, and socioeconomic factors. People also tend to exaggerate the reported intake of foods believed to be healthy and underreport the unhealthy ones.

So, the bottom line is that measuring diet is difficult and self-reported diet is infested with errors. From a critical point of view, should we then conclude that self-reported data are not worthy of scientific use or should we argue that we shouldn't throw out the baby with the bath water? Are there still something useful into these flawed data which can be used if we are careful in the interpretation and use? Are there available statistical adjustments which can reduce the challenges of errors? Or should we invest more in developing biological markers and observational methods, which may give us more objective methods – however is that a realistic scenario? And are these methods actually more accurate?

The debate about the value of self-reported data is not new; researchers have for decades debated forth and against self-reported data, and whether some dietary assessment methods may be superior to others. However, based on the development of new technology and advanced statistical approaches, are we closer to a conclusion now than earlier?

These questions will be addressed based on the state of the art literature.

**Disclosure of interest**: None to declare.

### Program no. 107

#### Reconciling conflicting evidence from observational studies and randomized controlled trials – Exemplified by dietary protein and weight change

##### Mikkel Z. Ankarfeldt^1,2^* ^1^Julius Center for Health Sciences and Primary Care, University Medical Center Utrecht, Netherlands; ^2^Novo Nordisk A/S, Denmark 
*Presenting author

The utility of observational studies and randomized controlled trials (RCTs) when assessing exposure-outcome relations is widely debated. On the one hand, the two study designs are seen as complementary. On the other hand, the RCTs are deemed more trustworthy than observational studies. With examples where the results from observational studies and RCTs seem to be in conflict, the validity of the observational study design has been questioned. However, systematic reviews across a range of medical topics find that observational studies and RCTs investigating similar populations, intervention, and outcome often show similar results. Also, it is suggested, that was at first looks like conflicting results can be explained if the observational data is modified so it becomes comparable with the RCT. The discussion of conflicting results from different study designs is also relevant in a nutritional context. While RCTs have shown weight loss and maintenance of weight loss with a high intake of protein, observational studies have shown an association between higher intake of protein and weight gain.

The objective of this work was to investigate possible explanations for conflicting results from observational studies and RCTs using the example of dietary protein and subsequent weight change.

Four studies all based on observational data investigated different aspects the apparent conflicting results of RCTs and observational studies of dietary protein and weight change. These studies are part of a PhD thesis with a title similar to the present abstract.

Study I hypothesized that the association between higher protein intake and weight gain seen in observational studies is due to a gain in fat-free mass. However, it was found that the weight gain was mostly in fat mass.

Study II hypothesized that differences in the study populations and the exposures of RCTs and observational studies are important to find an association between higher protein intake and better weight control. Among individuals with a high level of adiposity, similar to a RCT population, a lower weight gain was seen in the high protein group.

In continuation of Study II, it was hypothesized in Study III and Study IV that the association between protein intake and weight change depends on adiposity or genetic predisposition to adiposity, respectively. Such interactions were however not seen.

The conflicting evidence of dietary protein and weight change was not reconciled. It became clear that the observational studies and RCTs investigating dietary protein and weight change differ in several ways that were not investigated in Study I–IV. These differences may influence the findings, and the observational studies and RCTs investigating dietary protein and weight change may not be directly comparable.

**Disclosure of interest**: This work was carried out with no conflicts of interest. Currently M.Z.A. is employed at Novo Nordisk A/S as PostDoc with affiliation to University Medical Center Utrecht, which does not affect the presented work.

### Program no. 108

#### Lean or fit – can exercise and diet modify health-risks of obesity?

##### Mikael Fogelholm* Department of Food and Environmental Sciences, University of Helsinki, Helsinki, Finland 
*Presenting author

Obesity and weight-gain are well-known risk factors for both cardiovascular diseases and type 2 diabetes. By means of a systematic literature review, the speaker has shown that high physical activity and at least moderate aerobic fitness clearly attenuate the obesity-induced risk for cardiovascular diseases. There was also an interaction effect, since positive effects of physical activity were even stronger among obese, compared to normal-weight individuals. The evidence was strong enough for a conclusion that, compared with overweight (body mass index, BMI 25–29) or grade I obesity (BMI 30–34), poor aerobic fitness is a greater risk-factor for cardiovascular morbidity and mortality.

The interaction between obesity and diet as risk-factors for cardio-metabolic diseases has been studied much less, compared to the interaction between obesity and physical activity. By utilizing data from DASH-study, Fung et al. (2008) suggested that a healthy diet (an index based on the DASH-recommendations) might be more strongly related to lowered cardiovascular risk in normal-weight, compared to overweight and obese individuals. In this study, diet could not overcome the health-risks related to obesity.

The two recommended designs for a study on obesity-diet interaction are prospective cohorts and interventions. A cohort study has the possibility to use disease outcomes, which are more difficult in interventions. If the latter design is used, a 2 x 2 design approach is needed (a normal-weight and obese group, both randomized in two different diet treatments).

**Disclosure of interest**: None to declare.

### Program no. 109

#### Vitamin D status in the Nordic countries in the light of methodological standardization

##### Rikke Andersen*, Ida M. Grønborg and Inge Tetens Risk-Benefit Research Group, National Food Institute, Technical University of Denmark, Søborg, Denmark 
*Presenting author

The vitamin D status in the general population is an issue of concern world-wide due to the importance of vitamin D in multiple physiological functions. The concentration of 25-hydroxyvitamin D (25OHD) measured in serum or plasma reflects an individual's intake as well as the skin production of vitamin D, and 25OHD is the accepted biomarker of vitamin D status despite methodological challenges. Knowledge about the distribution of 25OHD concentrations in representative population samples is essential for quantification of vitamin D deficiency and for developing strategies for its prevention, as well as for setting dietary reference values.

The vitamin D status throughout Europe is determined by a number of factors such as sun exposure and habits, intake of supplements and fortified foods, color of skin. On top of these habitual and demographic differences between countries, we see methodological differences affecting the degree of comparability between countries and even within countries that would otherwise be expected to be similar.

It is an analytical challenge to measure serum 25OHD accurately and it has been shown that 25OHD assays can yield markedly differing results. There is substantial within-assay variation in 25OHD measurements and even greater between-assay variability. Such assay variation confounds comparison of 25OHD concentrations between studies and countries. Therefore, it is essential to standardize 25OHD measurements in both clinical and research laboratories.

The Vitamin D Standardization Program (VDSP) is an international collaborative effort promoting standardized laboratory measurement of total 25OHD. Nearly, all published vitamin D research is based on unstandardized 25OHD measurements, and it is impossible to standardize all old data. VSDP developed protocols for standardizing 25OHD measurements in existing national health/nutrition surveys.

The VDSP protocols were applied to existing serum 25OHD samples from two Danish studies, one Norwegian, and one Finnish study. A specifically selected subset of the bio-banked samples were reanalyzed for 25OHD by LC-MS/MS and a calibration equation developed between old and new 25OHD data, and this equation was applied to the entire data-set from each study. Compared to estimates based on the original 25OHD data, the percentage with vitamin D status below 30 nmol/l decreased by 21.5% in one of the Danish studies, by only 1.4% in the Norwegian study, and was relatively unchanged in the Finnish and the other Danish study, following VDSP standardization. In conclusion, standardization of 25OHD concentrations is important in order to compare vitamin D status across different study populations, which is needed to quantify and prevent vitamin D deficiency.

**Disclosure of interest**: None to declare.

### Program no. 110

#### Fortification as a solution for increasing vitamin D status

##### Christel Lamberg-Allardt* Calcium Research Unit, Department of Food and Environmental Sciences, University of Helsinki, Finland 
*Presenting author

Vitamin D deficiency leads to impaired bone health and may be related to increased risk of many non-skeletal health outcomes. In Northern Europe, exposure to sunlight is low or non-existing in the wintertime. Thus, between November and March, dietary or supplementary vitamin D intake is essential to maintain adequate 25-hydroxyvitamin D concentration (S-25(OH)D). During the summer period, the use of covering clothes or sunscreen prevents vitamin D synthesis in the skin. There are only a few good natural dietary sources of vitamin D: fish, cod liver oil, egg yolks, offal, and some wild mushrooms.

A huge challenge for the authorities is to increase vitamin D intake and improve vitamin D status in the population and vulnerable population groups. Although supplements are an attractive alternative, studies have shown that the impact on vitamin D intake and status is not impressive as e.g. compliance is not good. Thus, fortification of food is a feasible alternative for increasing vitamin D intake from a public health point of view. Fortification should be based on modeling of alternative, potential food sources in different age groups, and gender using national intake data. Special attention should be given to vulnerable groups in the population. The aim is to increase the intake especially in the population segments with the lowest intake bearing in mind that no one should have an intake exceeding the tolerable upper intake level (UL).

The nutritional policy strategy to improve vitamin D status in the population of Finland started with a recommendation for voluntary vitamin D fortification of particular staple foods in 2003 followed by a new recommendation in 2010, both based on modeling and simulation. The recommendation from 2010 was to add 1.0 µg/100 g vitamin D to all fluid milk products and respective lactose-free milk-, soy-, and cereal-based drinks and 20 µg/100 g to fat spreads. The recommendation was rapidly implemented by the food industry. Data from the Findiet 2012 study shows that fortification has increased vitamin D intake in adults to meet the present Nordic dietary recommendation. Data from the Health 2000/2011 cohort shows increase in 25(OH)D-concentrations as well as vitamin D intake that is attributable to vitamin D fortification of foods.

**Disclosure of interest**: None to declare.

### Program no. 111

#### Vitamin D and bone health – anything new?

##### Haakon E. Meyer^1,2^* ^1^Department of Community Health, University of Oslo, Oslo, Norway; ^2^Norwegian Institute of Public Health, Oslo, Norway *Presenting author

In the systematic literature review for the 5th Edition of the Nordic Nutrition Recommendations, we concluded that intervention with vitamin D (dose 10–20 µg/day) combined with calcium reduces the risk of total fracture and hip fracture, whereas intervention with vitamin D alone has not shown an effect in the doses tested out. No new studies have been identified changing this conclusion. However, a randomized controlled trial (RCT) published in 2015 concluded that intervention with high dose vitamin D increased the risk of falling. This is in line with two previous published RCT's reporting increased risk of falls and fracture in one and increased risk of hip fracture in the other after annual megadoses of vitamin D.

Several very large RCTs testing out the effect of high dose vitamin D are underway, and the first results are expected to be published during 2016.

**Disclosure of interest**: None to declare.

### Program no. 112

#### Benefits gained by improving vitamin D status in relation to overdiagnosis of insufficiency, overtreatment, and potential harms?

##### Karl Michaëlsson* Department of Surgical Sciences, Uppsala University, Uppsala, Sweden *Presenting author

Vitamin D stimulates enhanced intestinal calcium absorption and renal calcium conservation to maintain adequate levels of ionized calcium in serum. During periods of vitamin D deficiency, bone resorption increases as a result of reduced active calcium absorption and the bone mineral, therefore, decreases. In recent years, not only skeletal but also non-skeletal actions of vitamin D have been justifiably indicated. The major source of vitamin D is from synthesis in the skin by the action of UV-B radiation from sunlight. The strength of radiation depends on season and latitude and at high latitudes, the UV-B radiation is insufficient for the production of vitamin D during the winter season. In individuals residing at high latitudes, one would therefore expect considerable variation in vitamin D status between seasons. Serum 25-hydroxyvitamin D (S-25OHD) is the generally accepted indicator of vitamin D status. At what concentrations of S-25OHD should physicians in Nordic countries recommend supplementation? Differences in assay performances, seasonal variation, fat mass, nutritional status (e.g. calcium intake), and individual bioavailability of 25-hydroxyvitamin D by genetically determined concentrations of vitamin D-binding protein render detection of uniform threshold effects troublesome. Moreover, existing evidence does not support the commonly held belief that vitamin D supplementation in general prevents osteoporosis, fractures, and non-skeletal diseases. Consequently, the impression that vitamin D is a sunshine vitamin and that increasing doses lead to improved health is far from clear. Without stringent indications, that is, supplementing those without true insufficiency, there is a legitimate fear that vitamin D supplementation might actually cause net harm. The report from the Institute of Medicine also emphasized that there may be risks from both low and high levels of vitamin D and that there may be a U-shaped curve of risk, which has been seen with many other nutrients as well. Indeed, both recent observational studies and randomized clinical trials have displayed higher risk of disease occurrence at high S-25OHD concentrations and with high dose supplementation. Furthermore, seasonal differences of 15–35 nmol/L between the winter nadir and the summer zenith have been reported. This seasonality is not only seen at high latitudes but also in temperate climates. With such large fluctuations, it is somewhat surprising that season is rarely taken into account in studies of vitamin D and health outcomes. Novel results on how to define vitamin D insufficiency by season will be presented in my talk.

**Disclosure of interest**: None to declare.

### Program no. 113

#### Nutrition diagnosis – how dietitians identify and describe patients’ nutritional problems using standardized terminology

##### Elin Lövestam* Department of Food, Nutrition and Dietetics, Uppsala University, Uppsala, Sweden *Presenting author

Structured decision-making and clear communication are seen as essential parts of a high-quality and patient-safe health care. Among dietitians, the standardized Nutrition Care Process (NCP) and its connected terminology have been internationally implemented during the last years, as a framework and language for dietitians’ decision making and logical thinking. The NCP consists of four main steps: Nutrition Assessment, Nutrition Diagnosis, Nutrition Intervention, and Nutrition Monitoring and Evaluation. Connected to this process, the Nutrition Care Process Terminology (NCPT) is connected, providing standardized terms for each NCP step.

The nutrition diagnosis is seen as an essential NCP step, meaning the identification and labeling of an existing nutrition problem which the dietitian plan to address. For a patient with the medical diagnosis Diabetes Mellitus, the nutrition diagnosis might rather focus at carbohydrate intake, as that would be the problem that the dietitian is directly addressing. The nutrition diagnosis is expressed through a PES statement, combining the nutrition (P)roblem with its (E)tiology and connected (S)igns and symptoms. This can, for example, be formulated as follows: “[P:] Inadequate energy intake [E:] related to lack of appetite and poor food selection [S:] as evidenced by a daily intake of less than 75% of estimated needs.” The NCPT provides about 80 standardized diagnostic terms for the nutrition problem, grouped into the three main domains Intake, Clinical, and Behavioral–Environmental.

Recent studies show that dietitians using the NCP/NCPT have experienced benefits such as getting a consistent structure for nutrition care and critical thinking as well as an increased recognition of the dietitians and a facilitated communication with other health care professionals. However, dietitians also have expressed difficulties balancing the standardized process and terminology with a flexible, patient-centered approach.

During recent years, international interest regarding the implementation of NCP/NCPT has increased among dietitians. The NCPT has been translated to 11 languages and dialects, and the NCP/NCPT is currently being used or implemented in diverse parts of the world. Several national and international associations for clinical dietitians have supported the development and implementation of a standardized nutrition care process and terminology.

**Disclosure of interest**: None to declare.

### Program no. 114

#### Defining malnutrition – mission or mission impossible?

##### Ingvar Bosaeus* Clinical Nutrition Unit, Sahlgrenska University Hospital, Gothenburg, Sweden *Presenting author

The interest in detection and management of disease-related malnutrition (DRM) is increasing, and screening for DRM has expanded during recent years. However, a generally accepted standard for the definition of malnutrition is still lacking. The lack of a widely accepted definition prevents an adequate diagnosis of malnutrition and may affect the development of more effective interventions. Several malnutrition screening tools are increasingly used due to clinical feasibility. These screening tools most often use about the same variables, that is, weight loss, body mass index (BMI), eating difficulties, and a grading of on-going disease severity. Among the numerous tools available, ESPEN endorses the use of Nutritional Risk Screening 2002 (NRS-2002), Mini Nutritional Assessment-Short Form (MNA-SF) and Malnutrition Universal Screening Tool (MUST) to be used in the hospital, elderly care and community settings. The major use of these tools is to screen for malnutrition risk. The next step in the care process should be assessment, in order to design optimal nutritional therapy. The diagnostic procedure usually stops there. One reason is the lack of consensus over diagnostic criteria. In the absence of such criteria, it is difficult to distinguish the effectiveness and efficacy of nutritional therapies when applied in different phases of the malnutrition trajectory. The effects of nutritional therapy given preemptively at an early stage, before body protein and energy stores have been depleted, might differ as compared to when given at a late stage with overt depletion.

A study in 2010 showed the lack of agreement among experts on the elements defining malnutrition and diagnostic criteria. After that, discussion within nutritional societies has led to efforts to provide consensus on a definition and diagnostic criteria.

In the US, the Academy of Nutrition and Dietetics and A.S.P.E.N. propose etiologic-based definitions that consider time (acute vs. chronic) and degree of inflammatory response, and the identification of two or more of six characteristics is recommended for diagnosis. On the other hand, a recent ESPEN consensus statement aims to provide a consensus-based minimum set of criteria for the diagnosis of malnutrition to be applied independent of clinical setting and etiology, recommending that in individuals identified by screening as at risk of malnutrition, the diagnosis of malnutrition should be based on either a low BMI, or on the combined finding of weight loss together with either reduced BMI (age-specific) or a low fat free mass index (FFMI) using sex-specific cut-offs.

On-going and future validation studies will provide data that may confirm the feasibility of, or the need for revision of the malnutrition criteria of the consensus statements.

**Disclosure of interest**: None to declare.

### Program no. 115

#### Nutrition and patient safety

##### Randi J. Tangvik^1^*, Anne B. Guttormsen^2^, Anette H. Ranhoff^3^ and Grethe S. Tell^4^
^1^National Advisory Board on Disease Related Malnutrition, Oslo University Hospital, Oslo, Norway; ^2^Intensive Care, Haukeland University Hospital, Bergen, Norway; ^3^Research Center for Ageing and Dementia, Haraldsplass Hospital, Bergen, Norway; ^4^Department of Global Health and Primary Care, University of Bergen, Bergen, Norway *Presenting author


A strategy to improve nutritional care during hospitalization was introduced at Haukeland University Hospital in 2006. Nutritional guidelines were introduced, hospital staff was educated in basic clinical nutrition and mealtime routines were improved. To facilitate the initiative, nutrition surveys were established. During eight prevalence surveys in 2008–2009, 3,279 patients were included. Twenty-nine percent of the patients were at nutritional risk, according to NRS 2002. This risk was a significant risk factor for morbidity, increased use of hospitalization and death. Patients at nutritional risk were identified in all disease categories and all adult ages. Every item of the initial screening tool was found to be a significant independent risk predictor. A positive response to one or more of the initial four questions in NRS 2002 was associated with increased risk of morbidity and mortality, and positive answers to all four questions were associated with a 13 times greater risk of dying during the following year. Our findings support the need for nutritional screening in hospitals. A screening tool is immensely valuable for categorizing patients at nutritional risk, and NRS 2002 was found suitable for identifying high-risk patients. Introducing a nutrition strategy improved the screening performance among the hospital staff, but did not improve nutritional care to the patients. Therefore, more intense efforts are necessary to improve nutritional practice and staff knowledge in hospitals.

The Norwegian Patient Safety Programme: In Safe Hands, originally launched in 2011 as a patient safety campaign, continues as a five-year program (2014–2018), commissioned by The Norwegian Ministry of Health and Care Services, and carried out by the Norwegian Knowledge Centre for the Health Services. The overall aim is to reduce patient harm and improve patient safety. During June 2015, nutrition was launched as a new target area in the Norwegian campaign to contribute to patient harm prevention. A group of nutrition experts is established to identify a package of intervention to carry out. Screening for nutritional risk, preventing and treating disease-related malnutrition, and monitoring nutritional practices should be given priority. Pilot projects will be carried out in a university hospital and a nursing home during 2016. The pilot project will be evaluated prior to national implementation of the interventions in 2017.

**Disclosure of interest**: None to declare.

### Program no. 116

#### School meals and health – the PROMEAL-study

##### Agneta Hörnell^1^*, Maria Waling^1^, Anna S. Ólafsdóttir^2^, Hanna Lagström^3^, Hege Wergedahl^4^, Bert Jonsson^5^, Cecilia Olsson^1^, Eldbjørg Fossgard^4^, Asle Holthe^4^, Sanna Talvia^3^ and Ingibjörg Gunnarsdottir^6,7^
^1^Department of Food and Nutrition, Umeå University, Umeå, Sweden; ^2^School of Education, University of Iceland, Reykjavik, Iceland; ^3^Turku Institute of Child and Youth Research, University of Turku, Turku, Finland; ^4^Faculty of Education, Bergen University College, Bergen, Norway; ^5^Department of Psychology, Umeå University, Umeå, Sweden; ^6^Unit for Nutrition Research, Landspitali, The National University Hospital of Iceland, Reykjavik, Iceland; ^7^Faculty of Food Science and Nutrition, School of Health Sciences, University of Iceland, Reykjavik, Iceland *Presenting author


School meals, if both nutritious and attractive, provide a unique opportunity to improve health equality and public health. Despite this, only a few studies have been conducted with the aim to evaluate the effect of school meals and different ways of school meal organization on total dietary intake, classroom learning behavior and cognitive function. The study “Prospects for promoting health and performance by school meals in Nordic countries” (ProMeal), financed by NordForsk, is a multidisciplinary school-based study, which sets out to study this. We also wanted to study the children's perspectives of school lunches.

The four participating countries represent three different school meal organization systems: cooked school lunch free of charge in Finland and Sweden; partly subsidized cooked lunch or a lunch brought from home in Iceland, and lunch brought from home in Norway. About 200 10-year old children were recruited in each country, and a large amount of data was collected using mixed methods. Data collection included assessment of dietary intake from school meals through photographs of the school lunch trays/boxes (including extra helpings and/or leftovers) during one week, followed by systematic observations of the classroom behavior and measures of cognitive function (e.g. working memory, ability to concentrate and remain focused) after each lunch. In addition, pupil's experiences and perspectives of school meal situations were studied through focus group discussions and empathy-based stories.

The data are being analyzed and will result in several publications.

The study will give new insights into what future interventions need to focus on to improve pupils’ school lunch intake and learning, as well as providing valuable information for policy making, not least in countries where the history of school meals is shorter than in some of the Nordic countries.

**Disclosure of interest**: None to declare.

### Program no. 117

#### Childhood obesity trends in the Nordic countries

##### Berit L. Heitmann* Research Unit for Dietary Studies, The Parker Institute, Frederiksberg and Bispebjerg University Hospital, The Capital Region, Copenhagen, Denmark *Presenting author

Until recently, obesity rates have increased worldwide for children as well as for adults. However, most Western countries, including the Nordic countries, now see plateauing, or even declining rates of obesity among children. Still, childhood obesity is a significant public health concern, because of the high rates, as well as the tendency for socio economic differences in the recent developments, where a social gradient in obesity, favoring more advantaged children, seems present also in the Nordic countries, in spite of their relative affluence and equity. In addition, trends in BMI seem different than trends in waist circumferences that still generally suggest an increase over time. Finally, decreasing participation rates, the use of reported rather than measured data, small sample sizes, random fluctuations, urbanization, or lack of representativeness, all may contribute to explaining the plateaus.

**Disclosure of interest**: None to declare.

### Program no. 118

#### Maternal nutrition and child health

##### Ingibjorg Gunnarsdottir^1^*, Bryndis E. Birgisdottir^1^, Laufey Hrolfsdottir^1^, Thorhallur I. Halldórsson^1^ and Hildur Hardardottir^2,3^
^1^Unit for Nutrition Research, University of Iceland, Landspitali National University Hospital, Reykjavik, Iceland; ^2^Faculty of Medicine, University of Iceland, Reykjavik, Iceland; ^3^Department of Obstetrics and Gynecology, Landspitali University Hospital, Reykjavik, Iceland *Presenting author


Epidemiological studies have shown that adhering to a healthy diet during pregnancy is associated with a decreased risk for excessive weight gain during pregnancy as well as complications during pregnancy. Pre-pregnancy weight is recorded in standard maternity care, and weight is measured on several occasions during pregnancy. This provides unique opportunity to examine on a population level associations between pre-pregnancy weight and gestational weight gain with pregnancy complications. Knowledge on how dietary intake in pregnancy relates to health of the mother and child is currently mainly based on results from large, expensive epidemiological studies. Dietary intake is, however, rarely recorded in standard maternity care. As a result taking advantages of epidemiological findings on diet is difficult in clinical practice and development of screening tools that can be used in clinical setting to define women at nutritional risk who would benefit from a dietary treatment in pregnancy is urgently needed. Intervention studies aiming at improving diet and/or increasing exercise during pregnancy partly provide justification to this approach as they have shown that it is possible to decrease the risk of excessive pregnancy weight gain through changes in dietary habits. The importance of dietary interventions in preventing other complications including gestational diabetes needs further clarification. In previous intervention studies, women have either been selected into the study based on pre-pregnancy body mass index or the results presented in groups of normal weight women, overweight or obese. It might be suggested that dietary interventions in pregnancy that are targeted toward women who have a low quality diet in the beginning of pregnancy would be more effective than if women are selected for interventions based only on their pre-pregnancy weight, as many overweight and obese women might already have an relatively healthy diet.

A screening tool to identify nutritional risk in pregnancy has been developed and is currently being pilot tested at the Department of Maternal-Fetal Medicine of Landspitali University Hospital in collaboration with the Unit for Nutrition Research. Women attending ultrasound screening at 12–14 weeks of gestation, in the period from October 2015 to September 2016 (*n=*3,000, about 70% of pregnant women in Iceland) are invited to answer a simple electronic food frequency questionnaire (5–10 min), covering intake of basic food groups and socioeconomic background. Pre-pregnancy weight, weight gain during pregnancy, gestational length and pregnancy complications will be registered from hospital records. The aim of the study is to assess if a simple screening tool may be used to define women at nutritional risk in early pregnancy that predicts complications later in pregnancy.

**Disclosure of interest**: None to declare.

### Program no. 201

#### Dietary fatty acids – can you divide them into the good, the bad and the ugly

##### Lars I. Hellgren* DTU Systems Biology, Technical University of Denmark, Lyngby, Denmark *Presenting author

For many years, the message from nutritional science on the health impact of different types of fatty acids has been very simple; intake of saturated fatty acids should be reduced, intake of unsaturated, in particular polyunsaturated, fatty acids should be increased. In recent years, this very simplistic picture has been somewhat challenged. Several meta-analyses have indicated that the negative impact of the saturated fatty acids has been overestimated, and the differences between different saturated fatty acids have gained increased attention. Furthermore, the relative high intake of w-6 polyunsaturated fatty acids has raised some concern due to their alleged negative health effect through their role as substrate for synthesis of pro-inflammatory eicosanoids. In this lecture, I will give an overview on the current state of the evidence for classifying the fatty acid groups as having detrimental or positive health effects as well as briefly review the biochemistry behind some of the health effects.

**Disclosure of interest**: None to declare.

### Program no. 202

#### Changes in fat intake and changes in cholesterol over decades – the FINRISK Study

##### Tiina Laatikainen* Department of Health, National Institute for Health and Welfare, Helsinki, Finland *Presenting author

In Finland, the coronary heart disease mortality both among men and women has declined over 85% since 1970s. The major reduction observed in the mean serum cholesterol level has been one of the main contributors to this decline. Simultaneously, notable improvements have been seen in the diet of Finnish population.

Serum cholesterol levels of the Finnish population have been monitored since 1972 by the National FINRISK Studies and the diet since 1982 by the Findiet surveys conducted for a stratified random sample of adult population every five years. Altogether over 50,000 Finns have participated in these surveys.

Serum total cholesterol declined steadily from 1972 to 2007 but turned to a rise in 2012. In North Karelia and Northern Savo, where the surveys were started in 1972, the mean total cholesterol level was nearly 6.8 mmol/l in 1972. In 2007, the mean cholesterol level of Finnish men was 5.3 mmol/l and of women was 5.2 mmol/l. However, in 2012 a statistically significant increase in the mean serum cholesterol levels was observed total cholesterol being 5.3 mmol/l both in men and women.

Energy intake from fat in men declined from 38 E% to 33 E% from 1982 to 2007 and in women from 36 E% to 31 E%. Also, fat intake increased between 2007 and 2012 being among men 37 E% and among women 36 E% in 2012. Concerning the intake of fatty acids, the biggest decline from 1982 to 2007 was observed in the saturated fatty acids (SAFA). In men, the reduction was from 18 E% to 13 E% and in women from 18 E% to 12 E%. No major change was observed in the intake of monounsaturated fatty acids (MUFA) and only a slight increase in the intake of polyunsaturated fatty acids (PUFA). From 2007 to 2012 the intake of all fatty acids increased both among men and women. Unfortunately, the biggest increases were seen in SAFA and MUFA.

Analogous changes are seen in self-reported food choices. Since 1972, the use of butter both as a spread and in cooking and the consumption of fatty milk have decreased remarkably until 2007. Simultaneously, the consumption of vegetable oil has increased. Between 2007 and 2012, the use of butter and especially the butter-vegetable oil mixtures increased remarkably. The increased intake of SAFA was mainly explained by the increased use of products with milk fat, fatty meat and meat products.

The Finnish experience has shown that changes in diet have immediate effects on population risk factor levels and with relatively short delay effects are seen also in morbidity and mortality. Finnish Nutrition Recommendations following the Nordic Nutrition Recommendations give a solid basis for counseling. The challenge today is the mixed messages delivered especially in social media.

**Disclosure of interest**: None to declare.

### Program no. 203

#### Marine and vegetable sources of fatty acids – future perspective

##### Ann-Sofie Sandberg* Biology and Biological Engineering, Chalmers University of Technology, Gothenburg, Sweden *Presenting author

Seafood is the best and generally safe source of long-chain omega 3 fatty acids. Scarcity of marine food resources implies that new alternative sources are required such as microalgae, krill, and transgenic plants. Increasing recognition of the importance of marine fatty acids (the long-chain omega 3 fatty acids EPA and DHA) is due to their beneficial effects on cardiovascular health and for DHA also for the brain development in the fetus and the newborn. The short-chain omega 3 fatty acid ALA which is present in certain plant foods (e.g. canola oil and linseed oil) can be converted to EPA and DHA by the n-3 PUFA elongation and desaturation pathway. This conversion has been considered to be very limited, but new data suggest that the conversion might be underestimated, at least in women.

Several vegetable oils like corn oil and sunflower oil are rich in the essential omega 6 fatty acid linoleic acid (LA). Is there a difference in metabolic response to marine fatty acids and plant fatty acids? To advance our knowledge, concerning key pathways activated by marine and vegetable fatty acids animal models and systems biology can be applied. One example is shown to illustrate systems biology analysis that provided a mechanistic explanation for differences in response to an EPA and DHA supplemented high fat diet compared to a high fat diet supplemented with corn oil.

Furthermore, the whole food matrix may possess healthy qualities that cannot be reproduced by isolated components of the food. The oil fraction or the isolated fatty acids thus can be expected to differ in health effect from the whole vegetable or seafood.

**Disclosure of interest**: None to declare.

### Program no. 204

#### The obesity paradox

##### Thorkild I. A. Sørensen* Institute of Preventive Medicine, Bispebjerg and Frederiksberg Hospital, Copenhagen, Denmark *Presenting author

Obesity is associated with increased risk of multiple diseases of which diabetes, cardiovascular disorders and cancer are among the most prominent ones, all eventually leading to increased mortality. However, there are several paradoxes hidden in these well-established associations. The triglycerides accumulated as fat droplets in the adipocytes do not seem to be biologically harmful in itself, so why is obesity harmful and worse the heavier the person is? Reducing body weight in obese persons improves several risk factors for the co-morbidities, but contrary to the expectations, doing this by dietary restrictions is in most long-term studies associated with an increased mortality compared to keeping the body weight stable, and it remains unclear why this happens. Some studies suggest that when obese person do get some of the co-morbidities that they are at increased risk of, their prognosis is better than when non-obese persons get the same diseases, which is thought to reflect that the obese patients are better off during the possible malnutrition that follows the development and the co-morbidity. These paradoxes implies four important challenges: the first is the purely methodological one about the evidence, the second is about the theory that could possibly explain the paradoxes, the third is how to investigate such explanations, and the fourth is about the implications for prevention and treatment of obesity.

**Disclosure of interest**: None to declare.

### Program no. 205

#### The role of nutrition in sarcopenia and frailty

##### Tommy Cederholm* Public Health and Caring Sciences, Clinical Nutrition and Metabolism, Uppsala, Sweden *Presenting author

In the ageing societies it is crucial to identify factors that limit the functional capacity among the older adults and to find the remedies. Sarcopenia and frailty are two geriatric syndromes that are based on the partly inevitable catabolic processes accompanying aging. Muscle wasting of sarcopenia paves the way for vulnerability and non-resilience that are characteristics of frailty. Most of the pathogenic mechanisms behind sarcopenia and frailty are treatable in a way that may postpone the onset of these unwanted consequences of aging. Intuitively, a high protein intake could be expected to support muscle mass maintenance. This hypothesis has been confirmed in several prospective longitudinal epidemiological studies as well as in intervention studies, leading to the up-graded protein intake recommendation (to elderly) of 1.2–1.4 g protein/kg bw/day in the latest Nordic Nutrition Recommendation 2012. Whether animal, dairy (e.g. whey/casein), or vegetable proteins are superior is still not established. Although some studies indicate that essential amino acids and especially the branched-chain amino acid leucine may be an especially strong anabolic agent, this issue is still under investigation. Also timing of the protein intake over the day is an interesting issue where some question marks remain. Vitamin D is a nutrient that may prove to be a good anabolic stimulator of protein synthesis, although evidence is not clear yet. On the anabolic side, there are also interesting data, old as well as emerging, that essential poly-unsaturated fatty acids of both n-3 (e.g. eicosapentaenoic acid) and n-6 (linoleic acid) origin may have the capacity to trigger muscle protein synthesis. Since sarcopenia and ensuing frailty are partly the result of increased inflammatory and catabolic activities related to aging itself and to prevalent chronic disorders, it is hypothesized that some food that may have anti-inflammatory and anti-catabolic properties could be used as well for this purpose of supporting muscle mass and function. Once again n-3 and n-6 PUFA of marine or vegetable origin may play such anti-inflammatory roles. On the other hand, caution with high intakes of saturated fatty acids may be advocated due to their potential inflammatory effects, whereas anti-oxidants of vegetables may limit catabolic activities.

Thus, nutrition may have the potential to counteract muscle catabolism leading to sarcopenia and frailty. The dietary pattern that best stimulates protein synthesis and limit protein catabolism appears to correspond to the traditional Mediterranean diet or to its Healthy Nordic Diet equivalent high in protein, PUFA, and anti-oxidants.

**Disclosure of interest**: Receive unrestricted research grants from Nutricia and Nestle. Give lectures organized by Nutricia, Nestle, Arla and Fresenius-Kabi.

### Program no. 206

#### Nutrition in supporting brain health

##### Merja Suominen* Department of General Practice and Primary Health Care, and Helsinki University Central Hospital, Unit of Primary Health Care, University of Helsinki, Helsinki, Finland *Presenting author

The causes of memory disorders (MD) are multifactorial and result from both genetic and environmental factors. Prevention of cognitive impairment is important as the population is aging worldwide. Vascular and lifestyle-related risk factors have been associated with risk of late-life cognitive impairment and MD. Smoking, depression, low education, midlife hypertension, midlife obesity, diabetes, physical inactivity, and nonmodifiable risk factors like age, sex, and genetics like apolipoprotein E genotype (APOE ɛ4) have been associated with increased risk for MD. Physical activity and cognitive engagement have been associated with decreased risk for MD and protective in brain health. Malnutrition and nutrient deficiencies such as omega-3 fatty acids, B-vitamins, and antioxidants have been associated with cognitive impairment and MD in several studies.

Mediterranean-type diet rich in fish, fruits, and vegetables has been associated with reduced risk of cognitive decline. Mediterranean diet has affected positively in the four-year intervention, the primary outcome measure, neuropsychological test battery (NTB) in the groups of Mediterranean diet supplemented with olive oil and nuts. On the contrary, MD risk has been shown to increase with diets containing high amounts of meat, butter, high-fat dairy products, and refined sugars.

Studies in the general population have shown that higher dietary n-3 PUFAs intake is associated with lower dementia risk and slower age-related cognitive decline. n-3 PUFA-supplements have had no effect in healthy aging but they might be useful in early MD. If nutrient intake is already at optimum levels, vitamin supplements have not been effective. Mixed formulations, including n-3 PUFAs, antioxidants and B vitamins, and other nutrients, have had an effect on memory domains among mild MD.

More RCTs, however, are needed to confirm protective findings in preventing cognitive impairment and investigate strategies to maintain cognitive functioning. Recently some RCTs have been conducted aiming to investigate the prevention of cognitive decline with nutrition and other lifestyle factors. Findings from large, long-term, randomized controlled FINGER-trial suggest that a multidomain intervention could improve or maintain cognitive functioning in at-risk older population. Significant intervention effects were also seen in secondary cognitive outcomes such as executive functioning and processing speed, but no significant effect on memory was found. It is unknown whether the elements in nutrition that may protect against MD have an effect on brain vascular health or directly interfere with the pathogenesis in development of Alzheimer disease.

**Disclosure of interest**: None to declare.

### Program no. 207

#### Healthy nutrition and sustainability – is there a conflict?

##### Helle M. Meltzer* Norwegian Institute of Public Health, Oslo, Norway *Presenting author

There is global agreement as to what constitutes a healthy diet: plenty of vegetables, fruits and berries, pulses, regular intake of fish, vegetable oils, wholegrain, low-fat alternatives of dairy and meat, and limited intake of red and processed meat, sugar, salt, and alcohol. A diet scoring high on these indicators is associated with reduced risk of most non-communicable diseases, including coronary heart disease, diabetes, and several cancers.

With a fair distribution of food and low levels of food waste, such diets will also be among the best for sustainability. The projected world population makes further increases in total meat and fish consumption problematic, while the plant derived part of the diet should, in sum, become larger and more diverse. Diversity is also one of the keys to agricultural practices that preserve and develop the soil's fertility – a prerequisite for sustainability.

This is largely opposite to the current global dietary transition towards more refined sugars, refined fats, oils, and meats: Diet now emerges as the largest risk factor for non-communicable diseases. Improvements in the global health situation, such as the huge drop in childhood mortality and the global increase in life expectancy, are counter-balanced by the consequences of flawed dietary patterns, e.g. for the first time in history, there are more overweight and obese than undernourished people.

Present-day food production has a negative overall impact on biodiversity, soil-, air-, and water-quality, eutrophication, ocean acidification, contaminant release, and land use, in addition to causing excessive greenhouse gas (GHG) emissions.

These trends must change, and eating patterns and food choices could contribute substantially to that. A reduction in meat consumption together with reduced intake of unhealthy foods would improve the sustainability of our food consumption, especially if production methods approach long term best practices.

**Disclosure of interest**: None to declare.

### Program no. 208

#### Consumer understanding of healthy nutrition and sustainability

##### Elling Bere* Department of Public Health, Sport and Nutrition, University of Agder, Norway *Presenting author

Food has a great impact on health, and most official national dietary recommendations agree that a mainly plant-based diet with whole foods is the best option. However, dietary recommendations are changing according to best available scientific evidence, and there exist several more specific diets advocated by different experts. Describing an optimal diet is difficult, and the consumers might be confused.

Most official dietary recommendations are solely for improving health. What we eat also impact the environment. Food might be responsible for as much as 30% of manmade GHG emissions. Other examples of food related environmental effects are plentiful; depletion of fish stocks, soil erosion, over-consumption of fresh water, monoculture (reduces both plant and animal biodiversity), and pesticides and fertilizers polluting soil, water, and air.

All people have to eat, but we might change our food related behaviors to reduce environmental impact; options might be to eat less meat and more plant foods, to eat local and seasonal foods, to eat organically produced foods, to waste less foods, and to buy foods with less packaging.

The ambitious goal of the recent Paris Agreement adopted by 195 countries in December 2015, entailing carbon neutrality before the end of the century, demands that initiatives need to be generated within all areas of society. In other areas than food people are told to e.g. drive and fly less, change to low emission cars, lowering the temperature at home, insulating homes, powering down electronics, using less water, changing to energy saving light bulbs, and recycling.

In order to make changes, people must know the importance of food related to GHG emissions and environmental sustainability in general. But, do consumers really understand the (1) food related environmental impacts compared to other areas such as transportation and housing, and (2) do consumers understand the large differences between the different food related behaviors; i.e. the differentiated importance of reducing meat, eating locally, eating foods in season, eating organically produced foods, wasting less and less food, and buying foods with little packaging? This will be the topic of the present talk.

**Disclosure of interest**: None to declare.

### Program no. 209

#### Green Public Procurement in Denmark: Organic food consumption and healthier diets

##### Gregers Hummelmose^1^* and Nina N. Sørensen^2^* ^1^Danish Veterinary and Food Administration, Glostrup, Denmark; ^2^National Food Institute, Technical University of Denmark, Lyngby, Denmark *Presenting author

Whether organic food is healthier than conventional food has for long been the subject of scientific studies and part of a recurring public debate. From a nutritional point of view, an overall conclusion on this question seems to be that there are generally no distinct differences in micronutrient content between the individual organic and conventional food products. However, when looking at the composition of the diet, this picture might change.

Studies have shown that consumers with a high level of organic food consumption or equivalent professional kitchens with specific focus on procurement of organic food tend to follow the official dietary guidelines more closely.

The national political initiative “Danish Organic Action Plan 2020” was adopted in 2012. A prominent feature of this plan was directed towards an organic conversion of public kitchens, e.g. in childcare, schools, and hospitals. The starting point has been a mindset of a market-driven organic growth by providing financial support for the skills development of the kitchen staff with the secondary effect of increasing demand for organic food. Direct financial support for purchase of organic food has not been provided.

Introduced in 2009, The Organic Cuisine Label measures the organic percentage of food products used in kitchens and restaurants in Denmark based on invoices of procurement and are subject to official control by The Danish Veterinary and Food Administration. By dividing the kitchens into a bronze, silver, and gold category, the label signifies an official acknowledgement and marketing statement of the efforts implemented within the individual kitchens in terms of increasing the share of organic food, and is considered to be an important element in the overall organic agenda.

The number of kitchens using the Organic Cuisine Label have risen to more than 1,500, and sales of organic food to the Danish kitchens (public and private) have almost tripled since 2009, when the Organic Cuisine Label was introduced, reaching 1.3 billion DKK in 2014.

Since 2012, a PhD project has been researching the effects of organic food conversion in Danish public kitchens participating in the scheme, applying the Organic Cuisine Label method for measuring organic food percentages. Measured effects included changes to the organic food percentage in more than 600 public kitchens as well as psychological and physical wellbeing at work among public kitchen employees in more than 200 public kitchens, following an organic food conversion project. Curriculum components identified among the organic food conversion projects may also suggest side-effects of organic food conversion in other areas including food waste reductions, increased focus on plant-based meals, and limiting meat consumption. These results are in line with findings from earlier experiences of organic food conversion but further research is called for.

**Disclosure of interest**: None to declare.

### Program no. 210

#### NNR 2012 in perspective – what is the future role of nutritional recommendations?

##### Ambroise Martin* Lyon-Est Medical School, Claude Bernard University, Lyon, France. Former Chair of the Panel on Dietetic Products, Nutrition and Allergy (NDA Panel) of the European Food Safety Authority (EFSA) *Presenting author

European Food Safety Authority (EFSA) is currently finalizing the setting of Dietary Reference Values (DRVs), upon request of the European Commission. Since this request did not explicit any specific management perspective, and taking into account some comments received during public consultations in the process, the EFSA NDA Panel proposed to clearly consider the distinction between scientific assessment of nutrient reference values, only based on the relationship of the nutrient to human health, and nutrient goals and recommendations relevant for nutritional risk management. Considering all the uncertainties around DRVs and the failure of the automatic and simplistic application of the traditional probabilistic approach of DRVs for public health in western societies, nutrition management for optimizing health in relation to diets in a given population should explicitly take also into account other considerations than only the ingested amounts of micronutrients on the basis of their DRVs, focusing more on e.g. food patterns, dietary habits, socioeconomic issues, actual food composition in a given context or possibly environmental impact. As a consequence, establishing nutrient goals and recommendations and translating them into pragmatic and efficient food-based dietary guidelines require a lot of multidisciplinary scientific work which must be, and can only be, developed at the national or regional level as part of health, nutrition, and food policy, whereas DRVs could be developed at the European or even world level, in a fully transparent process and with a clear analysis of all the sources of variability and uncertainty (as recommended by the Efsa Scientific Committee) that would allow easy adaptation to any specific context. As an illustration, the current and yet unpublished 4-year work performed in France for the revision of the recommendations of the Nutrition-health policy (PNNS), using Efsa DRVs as one of the scientific bases, will be briefly presented. Obviously, there is an urgent need of more focused research on these topics so important for public health but still relatively neglected.

**Disclosure of interest**: None to declare.

### Program no. 211

#### Nordic Nutrition Recommendations 2012 – lessons and future directions

##### Jessica Ahlin* Swedish National Food Agency, Stockholm, Sweden *Presenting author

The Nordic countries have collaborated for decades with providing the Nordic Nutrition Recommendations (NNR). The work behind NNR 2012 was led by the NNR5 working group and other groups involved were selected experts and librarians, an external reference group, as well as a steering group. As a final part, an evaluation of the project was performed aiming at investigating the overall organization, possible improvements and prerequisites for a future edition of NNR.

The evaluation project consisted of web-based questionnaires which were sent to the scientific experts, librarians, Nordic authorities, university departments and research institutions, professional organizations, as well as organizations within the food industry. The questionnaires were developed in esMakerNX3 and targeted questions regarding the information and instructions given, workload and time frames, database searches, supporting tools given, communication between target groups, overall credibility of NNR, views on future updates, and the public consultation.

The overall opinion of the target groups regarding the project organization was that it was well prepared, and most information and instructions were clear. When selecting the expert groups, it would be optimal to have at least one person in each group, who is familiar with earlier NNR work. At the same time, some comments suggested that it is important to exchange several participants in the scientific writing groups, as well as the reviewers, for the revision. For several groups, the most difficult task during the whole process was to address and focus the research questions. The literature search was limited to post-2000 research, which several participants do not think is scientifically justified. All experts had other obligations aside from the NNR work which sometimes caused problems. One suggestion is to increase the number of experts involved, thus reducing their workload and allowing greater focus on their research questions. Another suggestion is to consider increasing the compensation for the experts and to give them full-time or part-time employment. The recommendations in NNR are widely used within authorities, universities, and nutrition organizations for research, seminars, and education. All the participants think that it is very important to produce a new NNR. The timing and extent of any future version should be carefully considered.

The overall opinion on the project organization and the work process is that it worked well. The outcome of NNR 2012 was good and had high credibility. A future revision is both wanted and needed. The most important changes that need to be addressed are to make sure that the literature searches for the systematic reviews cover a broader time frame and to reduce the workload for the experts, by involving more experts or by increasing their financial compensation.

**Disclosure of interest**: None to declare.

### Program no. 212

#### The role of individual treatment strategies

##### Ingrid Larsson* Department of Gastroenterology and Hepatology, The Unit of Clinical Nutrition, The Regional Obesity Center of Västra Götaland, Sahlgrenska University Hospital, Gothenburg, Sweden *Presenting author

Obesity is defined as BMI ≥ 30 kg/m^2^. Thus, a single treatment strategy cannot be expected to be sufficiently effective at BMI 45 kg/m^2^ as compared with BMI 30 kg/m^2^. International guidelines on obesity management conclude that different strategies should be introduced depending on BMI, that is, the higher BMI the more energy restrictive methods compared to lower BMIs. Bariatric surgery at BMI 40 kg/m^2^ or 35 kg/m^2^ with severe co-morbidities induces 30–35% weight loss 1–2 year after surgery. At BMI 28–35 kg/m^2^, 5–15% weight loss over 6–12 months are expected when a treatment strategy includes energy restriction combined with behavior therapy and/or pharmacological treatment. At BMI ≥35 kg/m^2^, 6-months weight loss of ≥20% can be expected. The higher BMI, the larger initial energy restriction can be used to improve long-term weight loss maintenance. Thus, an initial period of Very/Low Energy liquid Diets (V/LED) gives greater weight loss than more liberal energy restrictions. Five-year follow-up confirm the advantages on weight when V/LEDs are a part of weight loss treatment. Behavior therapy adds components to a structured obesity treatment that reinforces energy restriction and a negative energy balance and includes several documented components: self-monitoring, stimulus control, problem solving and pre-planning, relaxation, rewards and cognitive restructuring. The pharmacological treatment with orlistat^®^ will in near future, at least to some obese patients, be accompanied with liragutlid^®^ (GLP-1-analogue), now used in type 2-diabetes. The National Weight Control Registry shows that the components used for 10 years weight loss maintenance are the same as for weight loss: eating a low-energy, low-fat diet; eating breakfast regularly; maintaining a consistent eating pattern across weekdays and weekends; self-monitoring weight; and high levels of physical activity (about 1 hour per day). The greatest challenge of obesity treatment is prevention of weight regain and one of the consequences of this may be found in the inability of long-term follow-up studies on lifestyle change to show reductions in cardiovascular morbidity/mortality. However, these interventions remain important for obese patients to reduce cardiovascular risk and improve physical functioning and health-related quality of life.

Treatment of obese patients includes several options, although a negative energy balance is the common denominator of all weight loss strategies. An evidence-based treatment plan includes energy restriction, behavioral components, and pharmacological treatment of weight and risk factor management and regular physical activity as well as reduced physical inactivity. Dieticians/nutritionists as key members of the obesity team need to secure that evidence-based effective components for weight loss are part of the weight loss plan including prevention of weight regain by appropriate follow-up for the individual obese patient.

**Disclosure of interest**: None to declare.

### Program no. 213

#### Environmental prevention and treatment strategies

##### Knut-Inge Klepp^1,2^* ^1^Mental and Physical Health, Norwegian Institute of Public Health, Oslo, Norway; ^2^Nutrition, University of Oslo, Oslo, Norway *Presenting author

According to the World Health Organization (WHO), worldwide obesity has more than doubled since 1980. This increase is seen not only in high-income countries. Overweight and obesity are on the rise also in low- and middle-income countries, particularly in urban settings. Thus, prevention and treatment strategies clearly need to go beyond individual level factors and address the social causes that drive the epidemic across diverse settings worldwide.

A number of population-based strategies are available in order to impact both physical activity levels and healthy eating across the lifespan. The recent report from the WHO Commission on Ending Childhood Obesity presents a comprehensive list of 36 recommendations directly addressing childhood obesity across various sectors.

A strong and consistent social gradient in overweight and obesity rates is observed, as lower socio-economic status is associated with higher rates of overweight and obesity. This gradient is evident already at a young age, and it points to the need of also addressing more distal social determinants such as education, employment, and income levels when fighting obesity.

A number of systematic literature reviews have been published, summarizing the known effects of various intervention and policy measures addressing overweight and obesity at a population level. This presentation will review this literature, what is known and what are pressing research questions that need to be investigated. Furthermore, ethical issues related to environmental population-based strategies will be discussed.

**Disclosure of interest**: None to declare.

### Program no. 214

#### The role of food companies – barriers or facilitators?

##### Mette Peetz-Schou* Confederation of Danish Industries, Copenhagen, Denmark *Presenting author

The food industry has a natural role to play in the fight against obesity. We supply nutrients, ingredients, foods, and meal solutions to satisfy individual needs and are actively involved in health promotion in many different ways. Product formulation is one, but also information and education initiatives and voluntary marketing restrictions serve that purpose as well as research in new ingredients and technologies.

To solve the nutritional challenges, we face all stakeholders need to join forces and apply our individual strengths to the benefit of common nutritional goals. No stakeholder alone holds the key to resolve the challenges we face. Collaboration is key to success.

The presentation will provide examples of results obtained from collaboration as part of public–private partnerships and self-regulation approaches in Denmark. Furthermore, it will highlight the need to build trust across stakeholders, and to acknowledge the individual goals of stakeholders. For industry, the ability to innovate is of high importance. However, if the innovations cannot be communicated or the market potential of the innovations is too small, the incentive is low. Therefore, realism in goal setting and the ability to communicate is detriment to success for the industry.

The presentation will illustrate how realism in goal setting may also be detriment to the success of current public health efforts applying the Nordic green key-hole as an example.

**Disclosure of interest**: None to declare.

### Program no. 215

#### The changing scene of ageing – to become old yesterday, today, and tomorrow

##### Elisabet Rothenberg* Food and Meal Science, Kristianstad University, Kristianstad, Sweden *Presenting author

Life expectancy has risen rapidly in the last century due to economic growth, improved living standards, better lifestyles and education, and increased quality and availability of health care. The share of the population 65+, defined as older adults, is heterogeneous ranging from newly retired to centenarians. Covering more than a generation, they represent a great variation in living conditions and exposure to environmental factors with relevance for health. Mortality from all causes of death continues to decline. Non-communicable diseases with cardiovascular diseases (CVD) as the leading cause are responsible for the largest share of mortality, in the European Region (1). In Sweden, the key to increasing life expectancy in recent decades is decreasing mortality from CVD (2). Life-style factors including nutrition are important qualifiers for health and longevity. It has been shown that food habits among 70 year olds have changed in the same direction as in the population as a whole meaning better food habits in later born cohorts. As long as elderly stay healthy, they seem to maintain good food habits. A specific diet for healthy ageing does not exist. The Nordic Nutrition Recommendation 2012 (NNR) (3) pinpoints specific (higher) recommendations for elderly, only for vitamin D and protein. To conclude, there is convincing evidence of a protective effect of vitamin D on bone health, total mortality, and the risk of falling, and that aging is associated with gradual loss of muscle mass, function, and strength (sarcopenia). Moreover, that chronic disease might cause losses of body protein by disease-related catabolism, periods of bed rest, or loss of appetite. However, to maintain muscle mass and function, also physical exercise mainly resistance training is needed to stimulate muscle protein synthesis. The ageing process *per se* is not possible to change, but the conditions causing aging, disease, and death are possible to influence, which has been clearly proven by the progress life expectancy during the last 100 years. New knowledge about healthy food patterns and how to combine this with physical activity may keep older adults fit even longer in the future. In the coming year's knowledge about relations between health and microbiota will increase also our knowledge of how to use personalized diet to improve health will increase. Further on new diseases as has not traditionally been associated with nutrition as for instance dementia will probably be of target for nutrition interventions in the future.

**Disclosure of interest**: None to declare.

### Program no. 216

#### Nutritional status for healthy aging

##### Agnes N. Pedersen* Division of Risk assessment and Nutrition, Danish Technical University, National Food Institute, Søborg, Denmark *Presenting author

Elderly are a very inhomogeneous group including those who age successfully, those who have a normal/average aging and the subgroup with accelerated aging.

Normal aging has changed over time, since the life expectancy and years without disability have increased during the last decades. Thus, after the age of 65 one could expect several years of life in a relative good health.

A good nutritional status is an important part of the prevention of age-related illnesses and disability.

Nutritional status includes the dietary intake and status of macro- and micronutrients, as well as the body composition and physical activity.

The Nordic Nutrition recommendations (NNR) aim at a healthy nutritional status that satisfies the nutritional needs and supports the overall good health in the prevention of diet-associated diseases.

In the recent NNR from 2012, there is a special focus on the elderly, for example, for the first time there is a slightly higher protein recommendation for elderly >65 years. This has implications for the other macronutrients as well as the food-based dietary guidelines.

The D-vitamin recommendation is also higher for the elderly, but in general the recommendations for the micronutrients are the same for all healthy adults. The main nutritional challenge is the age related decline in the energy expenditure resulting in a demand for a higher nutrient density in the food servings for elderly.

Several studies have pointed at a higher cut off value for BMI for the *present* elderly, but the increasing prevalence of obesity questions the implications for the *future* elderly. A decreasing muscle mass and muscle strength (sarcopenia) and increasing body fat are linked to disability and illnesses. Thus, a high physical activity level is crucially important for the elderly.

Also more focus on food patterns, a prudent diet, is very relevant for the elderly.

The overall impression and the positive message is that prevention seems to have no upper age-limit. It is never too late to eat a healthy diet and improve your physical activity level, and the relative positive impact almost seems to be unchanged throughout lifetime.

**Disclosure of interest**: None to declare.

### Program no. 217

#### Overweight and obesity – the pros and cons of targeting weight reduction in the overweight and obese elderly

##### Anne M. Beck* Research Unit for Nutrition, Herlev University Hospital, Herlev, Denmark *Presenting author

The deterioration in functional status caused by late life overweight and obesity supersedes that typically associated with aging alone. Being obese leads to decreased muscular strength and reduced ability to perform physical tasks. Moreover, excessive adiposity and muscle deterioration due to sarcopenia are cyclically reinforcing, so that physical frailty can be markedly progressive in older adults with BMI in the higher range. Ultimately, being obese impacts self-care and activities of daily living and can lead to early nursing home placement and increased cost of caring. Due to all this serious consequences, it seems obvious to recommend weight reduction in the overweight and obese elderly. So is it all necessary to consider the pros and cons of such a targeted intervention, instead of just getting started. This will be the focus of this presentation, based on a scoping review of the research in the area.

**Disclosure of interest**: None to declare.

### Program no. 301

#### FODMAP versus gluten sensitivity – do we have clear picture?

##### Knut E.A. Lundin^1,2,3^* ^1^Faculty of Medicine, University of Oslo, Oslo, Norway; ^2^Department of Gastroenterology, Oslo University Hospital, Oslo, Norway; ^3^Centre for Immune Regulation, University of Oslo, Oslo, Norway *Presenting author

Wheat gluten is very well known as the driving antigen in coeliac disease, a frequently seen autoimmune disease characterized by inflammation of the small intestinal mucosa. Most of the patients also have IgA autoantibodies against the endogenous enzyme tissue transglutaminase. The disease is exclusively seen in individuals carrying HLA-DQ2 or -DQ8, and T cell recognizing gluten in the context of these HLA molecules are of importance in the disease development. The disease is frequent as it affects 1–3% of the Scandinavian population. Wheat allergy is another disease caused by proteins in wheat but characterized by IgE mediated anaphylactic reactions. In recent years, a further clinical entity termed “non-coeliac gluten sensitivity” (NCGS) has attracted considerable attention. This condition can be said to exist when the patient has clinical reactions to gluten resembling those seen in coeliac disease, but where the typical inflammation in the mucosa is not found, and the patients are negative for autoantibodies against tissue transglutaminase. Thus, NCGS lack reliable biomarkers, HLA typing is of no use, and the condition can so far only be diagnosed after exclusion diets, re-challenge diets and clinical work-up. However, also this work-up is not standardized, and food challenges for diagnostic and experimental purposes are hampered by lack of reliable placebo vehicles.

Although a wide-spread trend to convert to gluten-free diet is observed in almost all Western societies, the majority of individuals doing so do not have a diagnosis of coeliac disease. This has attracted several scientists to explore the role of wheat gluten versus other food components on the clinical condition of the patients. It turns out that gluten-containing food is also a major contributor to Fermentable Oligo-, Di-, Monosaccharides and Polyols (FODMAP) in the diet, and these are well known to cause problems for patients with Irritable bowel syndrome. In particular, results from Australia suggest that after FODMAP reduction, wheat gluten can no longer cause any harmful effects in NCGS. However, careful re-challenge experiments with FODMAP are lacking, and so does demonstration that NCGS patients on a “gluten-free diet” in fact ingest low amounts of FODMAP. Other blinded re-challenge experiments with FODMAP-free gluten are being conducted, some data are already published, and at least some of these suggest an effect of gluten on the patients‘ symptoms in NCGS. Thus, we do not have a clear picture yet.

**Disclosure of interest**: None to declare.

### Program no. 302

#### Nutrition, gut microbiota, and health – where do we stand?

##### Erika Isolauri* Department of Paediatrics, University of Turku and Turku University Hospital, Turku, Finland *Presenting author

Recent demonstrations that a growing number of clinical conditions, ranging from allergic diseases to obesity, are linked to aberrant gut microbiota composition have fuelled clinical research interest in host–microbe crosstalk. Nutritional modulation of the gut microbiota may provide a new direction to attain prophylactic or therapeutic effects in non-communicable diseases. Above the impact on gut microecology, specific probiotic effects have been attributed to restoration to normal of increased intestinal permeability, improvement of the intestine's immunological barrier functions, alleviation of the intestinal inflammatory response, and reduced generation of proinflammatory cytokines characteristic of local and systemic inflammation. More recent evidence indicates that gut microbiota is also involved in the control of body weight and energy metabolism, affecting the two important causes of obesity: energy acquisition and storage, and contributing to insulin resistance and the inflammatory state characterizing obesity.

A series of clinical intervention studies by the NAMI (Nutrition, Allergy, Mucosal immunology and Intestinal microbiota) research group included a long-term follow-up, to assess the safety and efficacy of perinatal probiotic supplementation in infants with high risk of abnormal early microbial contact. Permanent colonization was not reported, nor adverse effects in long-term follow-up studies. Perinatal probiotic intervention was well-tolerated and succeeded in reducing the risk of atopic eczema and early excessive weight gain. Thus, the impact of nutritional modulation of early gut microbial communities on gut microbiota succession is temporary. However, according to the programming theory, early nutritional environment may permanently shape the risk of disease during the critical period when consolidation of the immune and metabolic phenotype ensues. The specific probiotic intervention promotes healthy immune and metabolic programming, conferring a long-term clinical benefit.

**Disclosure of interest**: None to declare.

### Program no. 303

#### Computational models of energy balance

##### Ola Wallengren* Clinical Nutrition, Sahlgrenska University Hospital, Gothenburg, Sweden *Presenting author

Quantifying the dynamic relationship between changes in dietary energy intake and body weight is a challenge. Simple rules of thumb such as the 7,000-calorie/kg rule, in which it is thought that eliminating 500 kcal/day from one's habitual diet will lead to a loss of approximately 0.5 kg of body weight per week, has been found to drastically overestimate actual bodyweight changes. However, dynamic mathematical models of human energy regulation, macronutrient metabolism, and body weight change are beginning to reach a level of sophistication required for accurate predictions.

Models assume that body weight is compartmentalized into fat and fat-free mass, and they link changes in the mass of these two compartments to corresponding changes in energy and macronutrient stores. Model energy expenditure terms are changes in resting energy expenditure, voluntary physical activity, spontaneous physical activity, the thermic effect of feeding, and the biochemical efficiencies associated with fat and protein synthesis.

Such models represent a quantitative “thought experiment” where the most relevant aspects of whole body macronutrient metabolism are simulated and extended to represent energy and macronutrient balance dynamics in different scenarios. Human experimental data can be integrated in these frameworks and thereby help us better understand the dynamic imbalances of energy and macronutrients that give rise to changes in body weight and composition.

Since these models are based on the energy balance principle, they have been suggested as a simple alternative to existing methods that estimate free-living energy intake which are either notoriously inaccurate or are prohibitively expensive. There is always a degree of individual biological variation however which cannot be captured by a model constructed in part from population averages. Thus, much more research is needed on improving model terms and supplying more accurate empirically derived coefficients.

Applications include modeling the metabolic response in different disease states to estimate the effects of the various treatment modalities. Models simulations can be used in the design of studies and predict the metabolic response to each intervention before the study is conducted and make estimations of long-term results of studies that are not practical to perform in the real world. They can also be applied by health care providers to modify treatment strategies in a timely manner. Applications extend to estimate the effect of policy changes on obesity prevalence and the influence of food supply trends on obesity.

This talk will give an overview of the various approaches that have been used to model body weight, body composition dynamics, and energy regulation in humans. It will highlight implications that these models have provided and give an understanding of how mathematical models can serve as a guide for future research.

**Disclosure of interest**: None to declare.

### Program no. 304

#### Iodine deficiency – a ghost from the past revisiting us

##### Lisbeth Dahl^1^*, Anne-Lise Brantsæter^2^, Liv E. Torheim^3^, Helena F. Nystrøm^4^, Lena Hulthen^5^, Ingibjörg Gunnarsdottir^6^, Iris Erlund^7^, Peter Laurberg^8^, Betina H. Thuesen^9^ and Helle M. Meltzer^2^
^1^National Institute of Nutrition and Seafood Research (NIFES), Bergen; ^2^Norwegian Institute of Public Health; ^3^Oslo and Akershus University College of Applied Sciences, Oslo, Norway; ^4^University of Gothenburg and Sahlgrenska University Hospital; ^5^University of Gothenburg, Gothenburg, Sweden; ^6^University of Iceland and Landspitali-National University Hospital, Reykjavik, Iceland; ^7^National Institute for Health and Welfare, Helsinki, Finland; ^8^Aalborg University Hospital and Aalborg University, Aalborg; ^9^Research Centre for Prevention and Health, Rigshospitalet – Glostrup, Glostrup, Denmark *Presenting author


Iodine deficiency has been recognized as a nutrition challenge with serious health consequences for hundred years. Although the number of iodine deficient countries has decreased since 1990, iodine deficiency is now re-emerging in Europe. Iodine is essential for thyroid hormone production that plays a crucial role in ensuring optimal growth and brain development of the fetus and child. Both low and high intakes of iodine may have severe health consequences not only for children, but also for adults as inadequate iodine intake increases the frequency of thyroid diseases. Monitoring and securing optimal iodine status of a population is of significant public health importance. Historically, iodine deficiency disorders such as goitre and mental retardation were common in Norway, Sweden, and Finland, whereas this was not seen in Iceland and Denmark because of large intake of seafood and higher content of iodine in the drinking water, respectively. From the beginning of the last century, different strategies have been used by the Nordic countries to improve iodine nutrition. Iodization of salt is the method recommended by WHO to correct iodine deficiency in a population. The availability and amount of iodine added to salt differ between the Nordic countries, and iodized salt has been a major source only in Sweden. In Iceland, seafood is still the most important iodine source. In Norway, Finland, and partly in Denmark, milk and dairy products are the main contributing sources of iodine due to iodine fortification of cow's fodder. The current iodine status in the Nordic population is not optimal, and there is clear indication of inadequate iodine intake in subgroups of the population, particularly pregnant women in Norway, Finland, Sweden, and Denmark. In Norway and Finland, a decreasing iodine intake has been associated with reduced intake of milk and dairy products. In Denmark, iodization of salt started in 1998 due to increased frequency of thyroid diseases and, in 2000, mandatory fortification of salt used in the commercial production of bread was introduced. Finland recently launched a new iodine recommendation stating that all salts used in food preparation should be iodized, including mass catering and food industry. The Norwegian Nutrition Council is currently developing a recommendation for an iodine strategy. Suboptimal iodine intake and status in pregnant women in Norway, Sweden, and Denmark gives rise to concern, due to emerging evidence that even mild to moderate iodine deficiency during pregnancy may negatively impact cognitive function and school performance in the children. Inadequate iodine status in risk populations shows that iodine has been a largely overlooked nutrient in the Nordic countries. Common Nordic and national policies and strategies are needed to secure the iodine status in the Nordic region.

**Disclosure of interest**: None to declare.

### Program no. 305

#### Exploring eating behavior in a changing society – challenges and opportunities

##### Eva Roos* Folkhälsan Research Center, Samfundet Folkhälsan, Helsinki, Finland *Presenting author

According to the socioecological model eating behavior is shaped by the immediate context or setting where the person exists, such as family, the school, and workplace. The immediate setting is situated in larger contexts, including the community and society in general, which influence the immediate setting and further the eating behavior of individuals. In a changing society the setting will be changed and further change the eating behavior. In the presentation I will discuss the possible influence on eating behavior of following changes in society; changed population structure, increased immigration and multiculturalism, increased influence of social media, more flexible work, new values, e.g. sustainability, arising in the society.

Changes in society will also influence on our possibilities as a researcher to explore eating behaviors. The eating behaviors become more and more individualized; the responsibility of shaping food eating behaviors has changed from society to the individual. More individualized and multicultural behavior increases the variety in eating behavior. Larger variety in food behavior and faster changes in eating behaviors increases the challenges to survey eating behaviors in the population. Lower response rates impair our possibilities to get representative eating behavior data of the population. Changed population structure and more diverse eating behavior may increase the need of more tailored ways of approaching different population groups and more tailored methods to exploring food behavior of different population groups. Developments of new technologies can influence on our possibilities to explore food behaviors. Use of mobile phones, new digital tools, and use of big data can enhance data collection and also open up ways of collecting new type of food behavior data. Use of new data collection methods using new technologies may be less burdensome for the participants. Limited research resources are a real challenge for researchers today, but may force researchers to do more research collaboration and interdisciplinary research. Increased interdisciplinary research may enable a better overall picture of factors that shape eating behavior and intake of food and nutrients and increase possibilities to develop more innovative and effective ways to change eating behaviors.

**Disclosure of interest**: None to declare.

### Program no. 306

#### Biomarkers for dietary intake-where do we stand?

##### Rikard Landberg^1,2^* ^1^Department of Food Science, Swedish University of Agricultural Sciences (SLU), Uppsala; ^2^Unit of Nutritional Epidemiology, Institute of Environmental Medicine, Karolinska Institutet, Stockholm, Sweden *Presenting author

Dietary biomarkers covering foods and food components provide an objective measure of actual intake and status, and are important tools needed as complements to classical food intake assessments. Dietary biomarkers are typically analyzed in different biofluids and tissues such as blood, serum, plasma, urine, hair, nail, skin, erythrocytes, and adipose tissue. Some of them are nutrients or bioactive compounds and reflect their status or exposure, and some are used as surrogate biomarkers of food intake. Only a few nutrients or foods are currently covered by validated intake biomarkers. Until recently, dietary biomarker discovery was mainly hypothesis driven, i.e. based on knowledge about specific compounds being unique in different foods that could be tested and validated as biomarkers. More recently, data-driven approaches using untargeted metabolomics, mainly mass-spectrometric methods, have entered the scene and are now dominating in biomarker discovery. Rapid development of databases covering compounds in different foods and their metabolites in human samples has facilitated the identification of biomarker candidates but is still a bottle neck in the discovery phase. Once a candidate biomarker has been identified, it needs to be validated and the reproducibility, i.e. the biomarker fluctuation over time, needs to be estimated. The validation process typically involves correlating biomarker concentrations with estimated intake under controlled conditions, estimate the impact of non-dietary determinants, and assess the pharmacokinetics. Estimation of biomarker sensitivity and specificity using receiver operating characteristic (ROC)-curves is also part of the validation. Biomarker reproducibility can be estimated by repeated sampling, typically over a period of couple of months or a year.

In this presentation, I will give an overview of the recent developments in the area of dietary biomarkers. New biomarkers identified for different food groups will be presented and discussed as well as recent standardized approaches for the validation process. New approaches combining dietary biomarkers and traditional dietary assessment to improve accuracy of the intake estimation in epidemiological studies will also be highlighted and discussed. Both data from the recent literature and new results from our laboratory will be presented.

**Disclosure of interest**: The author has received research funding from Barilla Sweden and from Lantmännen Research Foundation for unrelated research projects.

### Program no. 307

#### Public health nutrition education

##### Liv E. Torheim^1^*, Bryndis E. Birgisdottir^2,3^*, Inga Thorsdottir^2,3^, Aileen Robertson^4^, Runa Midtvåge^4^, Chalida Mae Svastisalee^4^, Hanne Gillett^4^, Agneta Yngve^5^ and Arja Erkkilä^6^
^1^Department of Nursing and Health Promotion, Oslo and Akershus University College, Oslo, Norway; ^2^Unit for Nutrition Research, Landspitali University Hospital, Reykjavik, Iceland; ^3^Department of Food Science and Nutrition, University of Iceland, Reykjavik, Iceland; ^4^Department of Nutrition and Midwifery, Metropolitan University College, Copenhagen, Denmark; ^5^School of Hospitality, Culinary Arts and Meal Science, Örebro University, Örebro, Sweden; ^6^Institute of Public Health and Clinical Nutrition, University of Eastern Finland, Kuopio, Finland *Presenting author


Most Nordic health policy priorities are diet related, including reduction of social health inequalities, prevention of non-communicable diseases (NCDs) and healthy aging. Unhealthy dietary patterns, high blood pressure and obesity are major risk factors for NCDs such as cancers, type 2 diabetes, and cardiovascular diseases. There exists enormous potential to promote health and prevent diseases through targeting unhealthy life style, and it is crucial to develop a qualified public health nutrition workforce in the Nordic countries in order to reduce the NCD burden. Professionals with broad capacity within the field of public health nutrition are necessary to identify and respond to the current health challenges. However, public health nutrition has not been defined as a profession in the Nordic countries.

Public health nutrition (PHN) is an evolving profession within nutrition science that focuses on solving nutritional problems affecting population groups rather than those of individuals. Central elements of the profession are to assess the impact of various aspects of the food systems on the nutritional status, health, and health inequalities of population groups, and to develop, recommend, and implement evidence-based measures to improve dietary intake and nutritional status of population groups. These measures may be environmental, educational, social, economic, structural, political, and/or legislative. The knowledge, skills, competencies, and cultural heritage of the broader community should form a basis for all analyses and actions. The competencies required to be an effective PHN practitioner has been described by several scholars as well as by the World Public Health Nutrition Association. However, there is considerable diversity of workforce capacity in different countries. Most countries in the world have under-developed workforces to address PHN issues, including the Nordic countries.

The Nordic network on Education in Public Health Nutrition (NEPHN) was established in 2014 with support from the Nordic Council of Ministers. The aim of the network is to further develop PHN education to train highly qualified experts who can handle nutrition related challenges in the Nordic region and globally. The network facilitates exchange of lecturers, students, innovative educational resources and teaching methods, and supports the consolidation of PHN as a recognized/accredited profession throughout the Nordic region. The network has done a mapping exercise of public health nutrition education offered in the Nordic region, and will develop a common curriculum in PHN and common online courses (MOOC). Academic institutions from each of the Nordic countries are represented in the network. The network is open for all Nordic academic institutions offering public health nutrition education or courses.

**Disclosure of interest**: None to declare.

### Program no. 308

#### A Nordic Food Composition Database – a potential goal for the future?

##### Liisa Valsta, for The Nordic Food Analysis Network (NFAN) c/o Nutrition Unit, National Institute for Health and Welfare, Helsinki, Finland *Presenting author

The Nordic Food Analysis Network, funded by the Nordic Council, is a working group for national food compilers and food chemists in the Nordic Countries. The aims of the network have been to coordinate chemical food analyses done, and to increase synergies and capacity building in the area.

The idea of a Nordic Food Composition Database was originally launched already decades ago. The initiative was put forward by some pioneers in the food composition data area and is based on the evident similarities between the Nordic countries found in the food cultures, and even to some extent languages. Benefits of a closer infrastructure and synergies with common activities have been seen early and they still exist.

Today's information technology provides several options for a closer collaborations between institutions managing the national food composition databases: 1) exploiting internet for distribution of work over organizational barriers, 2) using modular and web-based software, 3) using interfaces integrating information systems in different organizations, and 4) using instant messaging and other tools for computer-supported cooperative work needed in activities divided between several organizations.

As challenges for a closer collaboration or even a common or a “virtual” Nordic food composition database could be seen recent diverging developments in the infrastructures of the food information. In addition, the diversity of nutrient composition in equivalent foods is often real. This is sometimes due to divergence in fortification practices or in the composition of animal feeds in use resulting differences in nutrient values. Amounts of certain ingredients (e.g. salt and sugar) used in equivalent foods vary. Even different procedures still exist in compiling of food information (yield and retention factors, recipe structure).

The European Food Information Resource Network, EuroFIR AISBL (www.eurofir.org) provides harmonized infrastructure and a global dissemination channel for food composition. A common European access point of this kind is absolutely needed also in the future. However, while European food culture may be too wide for daily working together, it could be feasible among the Nordic Countries.

**Disclosure of interest**: None to declare.

### Program no. 309

#### Recent advances in research into a healthy Nordic dietary pattern

##### Matti Uusitupa* Institute of Public Health and Clinical Nutrition, University of Eastern Finland, Kuopio, Finland *Presenting author

There has been increasing interest in a healthy Nordic dietary pattern which may promote health, prevent chronic non-communicable diseases and gives scientific ground for dietary recommendations, including e.g. cardiovascular diseases, metabolic syndrome, type 2 diabetes, and certain cancers. A Healthy Nordic diet may offer an alternative for traditional Mediterranean diet that is extensively studied and also recommended for prevention of non-communicable diseases. A Healthy Nordic dietary pattern is based on local, domestic, and Nordic food items. Few intervention trials on healthy food choices suggest beneficial effects of a healthy Nordic diet, including improvement of lipid profile, lowered blood pressure, and anti-inflammatory properties. Furthermore, observational studies even suggest that following healthy Nordic dietary choices may result in lowered risk of cardiometabolic disorders and mortality. The Sysdiet study was a controlled dietary intervention trial based on food selection principles of a healthy Nordic diet. Typical food choices were rapeseed oil, soft margarines, low fat and fat-free dairy products, Nordic berries, fruits, vegetables, fish, and whole grain (rye, barley oats) products. The study was carried out in six Nordic centers in people with metabolic syndrome (N=166) and it lasted for 18–24 weeks. Diet was isocaloric in order to keep body weight unchanged. Control group followed an average Nordic diet with lower fiber intake but higher intake e.g. saturated fats. As compared to control diet, a Healthy Nordic diet resulted in a better lipid profile and lowered ambulatory blood pressure at the end of the study, and this experimental diet seemed also to be anti-inflammatory also documented by gene expression studies in adipose tissue and peripheral blood mononuclear cells and metabolomics studies. Better adherence to diet resulted in more marked changes in serum lipids and blood pressure. The lecture will review the current data from published studies on a Healthy Nordic diet and give suggestions for future research and implementations to promote the popularity of this Nordic diet.

**Disclosure of interest**: None to declare.

### Program no. 310

#### Challenges involved in studying a traditional Sami diet pattern as a determinant of health in modern time

##### Lena M. Nilsson^1,2^* ^1^Arcum, Arctic Research Centre at Umeå University; ^2^Public Health and Clinical Medicine, Nutritional Research, Umeå University, Umeå, Sweden *Presenting author

Culturally defined dietary patterns are increasingly being used as models for healthy eating. The traditional Sami diet serve as a good example of difficulties and challenges involved in this approach.

A traditional Sami diet includes large amounts of red meat, animal fat, boiled and unfiltered coffee, and small amounts of vegetables and cereals. Despite this deviation from established dietary recommendations, life expectancy and physical health among the Sami do not differ substantially from the majority population.

From an indigenous health perspective this is unique, and from a nutrition epidemiologist's perspective it is challenging. In the NSHDS (Northern Sweden Health and Disease Study) cohort, a high adherence to a traditional Sami diet score was associated with increased mortality, while a high adherence to a low-carbohydrate, high-protein score was not.

Conflicting results like these stress the importance of knowledge of culture, environmental history, nutritional transitions, and modern food trends when interpreting traditional dietary patterns in modern time. When availability of traditional food decreases, which modern food items will substitute these?

**Disclosure of interest**: None to declare.

### Program no. 311

#### Nutrients versus dietary patterns in nutrition epidemiology – methodological considerations

##### Isabel Drake* Clinical Sciences in Malmö, Lund University, Malmö, Sweden *Presenting author

Dietary pattern analysis is now common practice in nutritional epidemiology. Dietary patterns incorporate information on the context in which nutrients are consumed (i.e. foods), both known and unknown bioactive substances present in foods, as well as potential synergistic or antagonistic effects of nutrients. The main methodological challenge of nutritional epidemiology is the reliance on self-reported dietary data measured with error. The effect of a single nutrient or food on disease risk is likely small, and measurement errors combined with collinearity of nutrients and foods are likely to hamper estimation of an effect on disease risk. Dietary pattern analysis is considered as a way to overcome some of these weaknesses, although the different methods used are not without limitations. In this presentation, the main strengths and limitations with a nutrient versus dietary pattern approach in nutritional epidemiology will be highlighted. Particular focus will be on key methodological issues of different methods to study dietary patterns in observational settings. The methods will be illustrated using examples from the large population-based Malmö Diet and Cancer Study.

**Disclosure of interest**: None to declare.

## II. Oral presentations

### Program no. O101

#### Dairy and fish product intake and plasma phospholipid acids in young children

##### Nicolai A. Lund-Blix^1,2,3^*, Kjersti S. Rønningen^1^, Håkon Bøås^3^, German Tapia^3^ and Lene F. Andersen^2^
^1^Department of Paediatric Research, Oslo University Hospital, Oslo, Norway; ^2^Department of Nutrition, University of Oslo, Oslo, Norway; ^3^Division of Epidemiology, Norwegian Institute of Public Health, Oslo, Norway *Presenting author

***Background and aims***: There is a lack of studies comparing dietary assessment methods with biomarkers of fatty acids in children. Our aim was to evaluate the correlation between the reported intake of dairy and fish products assessed by a food frequency questionnaire (FFQ) and the plasma phospholipid acids pentadecanoic acid, EPA and DHA in young children.

***Methods***: Data for the present study was derived from the prospective cohort “Environmental Triggers of Type 1 Diabetes Study” (MIDIA). Infants were recruited from the Norwegian general population during 2001–2007. Out of 908 children followed, 110 children (age 3–10) had sufficient volume of plasma and FFQ filled in within 2 months from blood sampling and were included in this evaluation study. Quantitative determination of fatty acids in the phospholipid fraction of plasma was done by fatty acid methyl ester (FAME) analysis. The suitability of the FFQ for ranking participants was assessed by Spearman correlation analysis.

***Results***: Significant correlations were found between pentadecanoic acid and the total intake of dairy products (*r*=0.32), fat dairy products (*r*=0.39), and cheese products (*r*=0.36). EPA and DHA were significantly correlated with the intake of oily fish (*r*=0.27 and *r*=0.36, respectively) and cod liver/fish oil (*r*=0.51 for both EPA and DHA).

***Conclusion***: The present study suggests that frequency of intake of dairy and fish products high in fat content estimated with an FFQ is moderately correlated with the fatty acids pentadecanoic acid, EPA and DHA in the plasma phospholipid fraction in young children.

**Disclosure of interest**: None to declare.

### Program no. O102

#### Dietary total antioxidant capacity in early school age and subsequent allergic disease

##### Anna Gref^1^, Susanne Rautiainen^1^, Olena Gruzieva^1^, Niclas Håkansson^1^, Inger Kull^1,2,3^, Göran Pershagen^1^, Magnus Wickman^1,2^, Alicja Wolk^1^, Erik Melén^1,2^ and Anna Bergström^1^* ^1^Institute of Environmental Medicine, Karolinska Institutet, Stockholm, Sweden; ^2^Sachs’ Children's Hospital, Södersjukhuset, Stockholm, Sweden; ^3^Department of Clinical Science and Education, Karolinska Institutet, Stockholm, Sweden *Presenting author

***Background and aims***: Dietary antioxidant intake has been hypothesized to influence the development of allergic diseases; however, only a few studies have investigated this association. Our aim was to study the association between total antioxidant capacity (TAC) of the diet at age 8 and the subsequent development of asthma, rhinitis, and sensitization to inhalant allergens between ages 8 and 16, and to assess potential effect modification by known risk factors.

***Methods***: 2,359 children from the Swedish birth cohort BAMSE were analyzed. Dietary TAC at age 8 was estimated by combining information on the child's diet in the past 12 months from a food frequency questionnaire with TAC values of common foods analyzed with the oxygen radical absorbance capacity method. Disease status of asthma and rhinitis were based on questionnaires, and serum IgE antibodies were measured at ages 8 and 16.

***Results***: A statistically significant inverse association was observed between TAC of the diet and incident sensitization to inhalant allergens (adjusted odds ratio: 0.73, 95% confidence interval: 0.55–0.97 for the third compared to the first tertile, *p*-value for trend=0.031). No clear associations were observed between TAC and development of rhinitis or asthma, although an inverse association was observed with incident allergic asthma (asthma in combination with sensitization to inhalant allergens) (adjusted odds ratio: 0.57, 95% confidence interval: 0.34–0.94 for the third compared to the first tertile, *p*-value for trend=0.029).

***Conclusion***: Higher TAC of the diet in early school age may decrease the risk of developing sensitization to inhalant allergens up to adolescence.

**Disclosure of interest**: None to declare

### Program no. O103

#### Dietary habits in adolescence and midlife and risk of breast cancer

##### Alfheidur Haraldsdottir^1,2^*, Johanna E. Torfadottir^2,3^, Unnur A. Valdimarsdottir^2,4,5^, Thor Aspelund^2,6^, Laufey Tryggvadottir^7,8^, Tamara B. Harris^9^, Lenore J. Launer^9^, Lorelei A. Mucci^5,10^, Edward L. Giovannucci^5,10,11^, Hans-Olov Adami^4,5^, Vilmundur Gudnason^6,8^ and Laufey Steingrimsdottir^1,3^
^1^Faculty of Food Science and Human Nutrition, University of Iceland, Reykjavik, Iceland; ^2^Centre of Public Health Sciences, Faculty of Medicine, University of Iceland, Reykjavík, Iceland; ^3^Unit for Nutrition Research, Landspitali National University Hospital, University of Iceland, Reykjavik, Iceland; ^4^Department of Medical Epidemiology and Biostatistics, Karolinska Institutet, Stockholm, Sweden; ^5^Department of Epidemiology, Harvard T.H. Chan School of Public Health, Boston, MA; ^6^The Icelandic Heart Association, Kopavogur, Iceland; ^7^The Icelandic Cancer Registry, Reykjavik, Iceland; ^8^Faculty of Medicine, University of Iceland, Reykjavik, Iceland; ^9^Laboratory of Epidemiology and Population Sciences, Intramural Research Program, National Institute on Aging, Bethesda, MD; ^10^Channing Division of Network Medicine, Department of Medicine, Brigham and Women's Hospital, Harvard Medical School, Boston, MA; ^11^Department of Nutrition, Harvard T.H. Chan School of Public Health, Boston, MA *Presenting author

***Background and aims***: Diet during adolescence might be of significance for breast cancer (BC) risk due to rapid cell proliferation during maturation. Our aim was to explore the effects of high vs. low consumption of milk, fish, cod liver oil, meat, salted or smoked meat, and whole grain products in adolescence and midlife, on BC risk later in life.

***Methods***: We used data from the Reykjavik-AGES study that was initiated in 2002. A total of 3,326 women born between 1908 and 1935 provided retrospective information on dietary habits in adolescence and midlife. The AGES study is a follow-up of the Reykjavik Heart Study, initiated in 1967, and includes numerous metabolic, social, and anthropometric variables. By linkage with the Icelandic Cancer Registry, information on BC diagnoses was available. Cox proportional hazard regression models were used to analyze the risk of BC and adjustments were made for a series of potential confounders, including those related to fertility.

***Results***: During a mean follow-up of 8.1 years, 91 women were diagnosed with BC. For adolescence, we found high consumption of rye bread (daily or more often) compared with low consumption (less than daily) to be associated with BC (HR 1.63, 95% CI 1.04–2.56). Positive association was also found for high midlife consumption of rye bread (HR 1.65, 95% CI 1.05–2.58) compared with low consumption. In addition, high fish consumption (>4 portions per week) in midlife was inversely associated with BC (HR 0.46, 95% CI 0.22–0.97) when compared with lower fish consumption (<2 portions per week).

***Conclusion***: Our findings suggest that high rye bread consumption in early life and midlife may be associated with increased risk of BC, possibly via estrogenic effects of phytoestrogens, found in rye bread. Our findings also suggest that high fish consumption in midlife may be associated with reduced risk of BC.

**Disclosure of interest**: None to declare

### Program no. O104

#### Dietary intakes and gut microbiota composition in a Swedish population

##### Louise Brunkwall^1^*, Ulrika Ericson^1^, Olena Prykhodko^2^, Peter Almgren^1^, Peter Nilsson^3^, Emily Sonestedt^1^, Frida Fåk^2^ and Marju Orho-Melander^1^
^1^Clinical Sciences in Malmö, Lund University, Malmö, Sweden; ^2^Medicon Village, Lund University, Lund, Sweden; ^3^Department of Internal Medicine, Skåne University Hospital, Malmö, Sweden *Presenting author

***Background and aims***: Several studies have shown that diet affects the constitution of the human gut microbiota. However, many of these studies have been conducted within controlled settings with limited generalizability. We aim at investigating if intakes of fiber, plant foods, and meat associate with the gut bacterial composition in the ongoing Malmö Offspring Study (MOS).

***Methods***: The present study included 184 individuals (mean age 41, 51% women, and no antibiotics in previous 6 months) from MOS. DNA was extracted from fecal samples and the 16S rRNA V1-V3 region was sequenced. High-quality sequence reads were binned into operational taxonomic units (OTUs) using QIIME software. Singletons and low abundance OTUs were removed. Dietary data on fiber, whole grain, vegetables, fruit, and meat (g/kJ) were obtained from a validated 4-day dietary record (Riksmaten 2010). We applied a linear regression model adjusted for age and sex, to examine associations between tertiles of dietary intakes and relative abundance of the bacteria (logarithmically transformed). False discovery rate was applied to correct for multiple testing.

***Results***: However, no associations remained significant after correction for multiple testing.

**Conclusion:** Intakes of fiber, whole grain, fruits, and meat showed nominally significant associations with relative abundance of several gut bacterial genera. Currently, we are sequencing 300 feces samples to confirm and extend these findings.

**Table 1:** Association between the food groups and bacteria at genus level

Exposure	Phyla	Genus	P for trend
Fiber	Firmicutes	Acidaminococcus	0.008
	Bacteroidetes	Parabacteroides	0.050
Whole grain	Firmicutes	Phascolarctobacterium	0.012
		Erysipelotrichaceae	0.025
		Turicibacter	0.007
	Bacteroidetes	Paraprevotella	0.003
Fruits	Firmicutes	Blautia	0.017
		Lachnobacterium	0.050
Meat	Proteobacteria	Sutterella	0.014

**Disclosure of interest**: None to declare

### Program no. O105

#### Unsatisfactory knowledge and use of terminology regarding malnutrition, starvation, cachexia, and sarcopenia among dietitians in Belgium, the Netherlands, Norway, and Sweden

##### Lies ter Beek^1^*, Erika Vanhauwaert^2,3^, Frode Slinde^4^, Ylva Orrevall^5^, Christine Henriksen^6^, Madelene Johansson^4^, Carine Vereecken^2^, Elisabet Rothenberg^7^ and Harriët Jager-Wittenaar^1,8^
^1^Research group Healthy Ageing, Allied Health Care and Nursing, Hanze University of Applied Sciences, Groningen, Netherlands; ^2^Faculty of Health and Social Work, Centre of Expertise Healthy Living, University Colleges Leuven-Limburg, Diepenbeek, Belgium; ^3^Department of Clinical and Experimental Medicine, KU Leuven, Leuven, Belgium; ^4^Department of Internal Medicine and Clinical Nutrition, Sahlgrenska Academy, University of Gothenburg, Gothenburg; ^5^Department of Learning, Informatics, Management and Ethics, Karolinska Institutet, Stockholm, Sweden; ^6^Faculty of Medicine, Department of Nutrition, Institute of Basic Medical Sciences, University of Oslo, Oslo, Norway; ^7^Food and Meal Science, Kristianstad University, Kristianstad, Sweden; ^8^Department of Oral and Maxillofacial Surgery, University Medical Center Groningen, University of Groningen, Groningen, Netherlands *Presenting author

***Background and aims***: Clinical signs of malnutrition, starvation, cachexia, and sarcopenia overlap, as they all imply muscle wasting. However, the underlying mechanisms differ fundamentally and therefore distinction between these phenomena has therapeutic and prognostic implications. We aimed to determine whether dietitians in selected European countries have “sufficient knowledge” regarding malnutrition, starvation, cachexia, and sarcopenia, and use these terms in their daily work.

***Methods***: An anonymous online survey was performed among dietitians in Belgium, the Netherlands, Norway, and Sweden. “Sufficient knowledge” was defined as having mentioned at least two of the three common domains of malnutrition: “nutritional balance,” “body composition,” and “functionality and clinical outcome,” and a correct answer to three cases on starvation, cachexia and sarcopenia.

***Results***: From the 712 respondents, 369 were included in the analysis (5% of the invited 7,186). The term “malnutrition” is being used in practice by 88% of the respondents, and starvation, cachexia, and sarcopenia by 3%, 30%, and 12%, respectively. The cases on starvation, cachexia, and sarcopenia were correctly identified by 58, 43, and 74%. Thirteen percent of the respondents had “sufficient knowledge.” The proportion of respondents with “sufficient knowledge” was significantly higher in those working in a hospital or municipality (16%, *p*<0.041), as compared to those working in other settings (7%).

***Conclusion***: The results of our survey among dietitians in four European countries show that the percentage of dietitians with “sufficient knowledge” regarding malnutrition, starvation, cachexia, and sarcopenia is unsatisfactory (13%) and that these terms are not often used by dietitians in daily practice. Identifying cases is performed better than the theoretical understanding of the concept of malnutrition.

**Disclosure of interest**: None to declare

### Program no. O106

#### Association between weight change and mortality in community living older people followed for up to 14 years: the Hordaland Health Study (HUSK)

##### Teresa R. Haugsgjerd^1^*, Jutta Dierkes^2^, Stein E. Vollset^1^, Kathrine J. Vinknes^3^, Ottar K. Nygård^4^, Reinhard Seifert^4^, Gerhard Sulo^1^ and Grethe S. Tell^1^
^1^Department of Global Public Health and Primary Care, University of Bergen, Bergen, Norway; ^2^Department of Clinical Medicine, University of Bergen, Bergen, Norway; ^3^Department of Nutrition, University of Oslo, Oslo, Norway; ^4^Department of Clinical Science, University of Bergen, Bergen, Norway *Presenting author

***Background and aims***: It is unclear what degree of weight changes in older people are associated with mortality. The aim was to study the importance of weight change with regard to mortality in older people.

***Methods***: The cohort includes participants in the Hordaland Health Study 1997–99 (*N*=2,935, age 71–74) who had previously participated in a survey in 1992–93. Participants with weight measured at both surveys were followed for mortality through 2012. Cox proportional hazards models were used to calculate risk of death according to changes in weight. Hazard ratios (HR) with 95% confidence intervals (CIs) for people with stable weight (±<5% weight change) were compared to people who lost (≥5%) or gained (≥5%) weight. Chi-square analyses or t-tests were applied to determine whether the survivors and non-survivors in the weight change groups differed on the baseline characteristics. Cox regression with penalized spline was used to evaluate the association between weight change (in kg) and mortality. Analyses were adjusted for age, sex, physical activity, smoking, diabetes, hypertension, and previous myocardial infarction or stroke. Participants with cancer were excluded.

***Results***: Compared to those with stable weight, participants who lost ≥5% weight had an increased mortality risk (HR 1.59 [95% CI: 1.35–1.89]) while the group with weight gain ≥5% did not (HR 1.07 [95% CI 0.90–1.28]). Among the survivors in both weight change groups, the proportion of men, ever smokers, and participants with diabetes and with previous myocardial infarction were significantly lower than among those who died. Penalized spline identified those who lost more than about 3 kg or gained more than about 12 kg as having increased risk of death.

***Conclusion***: Even a minor weight loss of ≥5% and >3 kg showed increased risk of mortality. Thus, weight should be routinely measured in older people.

**Disclosure of interest**: None to declare

### Program no. O107

#### Iodine intake in pregnancy is associated with neurodevelopment at age 3: results from The Norwegian Birth Cohort Study (MoBa)

##### Marianne H. Abel^1^*, Liv E. Torheim^2^, Margaretha Haugen^3^, Ragnhild E. Brandlistuen^4^, Heidi Aase^4^, Ida H. Caspersen^3^, Jan Alexander^3^, Helle M. Meltzer^3^ and Anne L. Brantsæter^3^
^1^R&D, TINE SA, Oslo, Norway; ^2^Faculty of Health Sciences, Department of Health, Nutrition and Management, Oslo and Akershus University College of Applied Sciences, Oslo, Norway; ^3^Domain for Infection Control and Environmental Health, Norwegian Institute of Public Health, Oslo, Norway; ^4^Department of Child Development and Mental Health, Norwegian Institute of Public Health, Oslo, Norway *Presenting author

***Background and aims***: Severe iodine deficiency (ID) has detrimental effects on fetal brain development. Less is known about potential consequences of mild to moderate ID. Data from MoBa revealed a substantial variation in iodine intake among Norwegian pregnant women, and the majority did not meet the Nordic recommended intake of 175 µg iodine/day. The aim of this study was to explore associations between maternal iodine intake and measures of neurodevelopment at age 3.

***Methods***: For this study, 34,477 children in MoBa born in 2002–2009 met the inclusion criteria (singleton births, no reported use of iodine supplements, or thyroid medication in pregnancy). Iodine intake was calculated from a validated food frequency questionnaire covering first half of pregnancy. We used logistic regression to examine maternal questionnaire reports of language development, communication skills, fine and gross motor development, and behavior problems (externalizing and internalizing) adjusting for confounders.

***Results***: One third of the mothers had iodine intakes <100 µg/day. Preliminary results show that intake of <100 compared to 150–200 µg/day was associated with increased risk of scoring outside predefined cutoff values on all selected outcomes except for motor development (adjusted odds ratios (OR) 1.2–1.7; *p*<0.05). For behavior problems and severe language delay, there was also increased risk at 100–150 µg/day (OR 1.1–1.7; *p*<0.05). High iodine intake (>250 µg/day) was associated with increased risk of any language delay, particularly severe language delay (OR 1.5–2.1; *p*<0.05).

***Conclusion***: This study indicates that maternal mild to moderate ID is associated with increased risk of a range of negative neurodevelopmental outcomes at 3 years. An intake above 250 µg/day was also associated with increased risk.

**Disclosure of interest**: M. H. Abel is an industrial PhD student employed by the dairy company TINE SA. Her PhD project on iodine in collaboration with The Norwegian Institute of Public Health and Oslo and Akershus University College of Applied Sciences is funded by TINE SA and The Research Council of Norway; L. E. Torheim: None to declare; M. Haugen: None to declare; R. E. Brandlistuen: None to declare; H. Aase: None to declare; I. H. Caspersen: None to declare; J. Alexander: None to declare; H. M. Meltzer: None to declare; A. L. Brantsæter: None to declare.

### Program no. O108

#### Effects of a 2-year individualized and family-based lifestyle intervention on physical activity and diet quality in children

##### Anna Viitasalo^1^, Aino-Maija Eloranta^1^*, Niina Lintu^1^, Juuso Väistö^1^, Taisa Venäläinen^1,2^, Sanna Kiiskinen^1^, Panu Karjalainen^1^, Jaana Peltola^1^, Eeva-Kaarina Lampinen^1^, Eero A. Haapala^1,3^, Jussi Paananen^4^, Ursula Schwab^2,5^, Virpi Lindi^1^ and Timo A. Lakka^1,6,7^
^1^Institute of Biomedicine/Physiology, University of Eastern Finland, Kuopio, Finland; ^2^Institute of Public Health and Clinical Nutrition/Clinical Nutrition, University of Eastern Finland, Kuopio, Finland; ^3^Department of Biology of Physical Activity, University of Jyväskylä, Jyväskylä, Finland; ^4^Institute of Biomedicine/Bioinformatics Center, University of Eastern Finland, Joensuu, Finland; ^5^Institute of Clinical Medicine/Internal Medicine, University of Eastern Finland, Kuopio, Finland; ^6^Department of Clinical Physiology and Nuclear Medicine, Kuopio University Hospital, Kuopio, Finland; ^7^Kuopio Research Institute of Exercise Medicine, Kuopio, Finland *Presenting author

***Background and aims***: To investigate the effects of a long-term, individualized and family-based lifestyle intervention on physical activity and diet quality in children.

***Methods***: We carried out a 2-year intervention study in a population sample of 506 children aged 6–8 years in Finland in 2007–2012. We allocated the participants in the intervention group (*n*=306) and the control group (*n*=200) at baseline. The intervention aimed at increasing physical activity and enhancing diet quality by 6 physical activity and diet counseling sessions for the children and their parents and was based on Nordic and national recommendations. We assessed total physical activity and different types of physical activity by a detailed questionnaire and diet by 4-day food records. We studied the effects of the intervention using linear mixed models adjusted for age and sex.

***Results***: Total physical activity (+9 min/d in intervention group vs. −5 min/d in control group, *p*=0.001 for time*group interaction), unsupervised physical activity (+7 min/d vs. −9 min/d, *p*<0.001), and organized sports (+8 min/d vs. +3 min/d, *p*=0.001) increased more in the intervention group as compared with the control group. Consumption of vegetables (+12 g/d vs. −12 g/d, *p*=0.001), high-fat vegetable oil-based margarine (+10 g/d vs. +3 g/d, *p*<0.001) and low-fat milk (+69 g/d vs. +11 g/d, *p*=0.042) and intake of dietary fiber (+1.3 g/d vs. +0.2 g/d, *p*=0.023), vitamin C (+4.5 mg/d vs. −7.2 mg/d, *p*=0.042), and vitamin E (+1.4 mg/d vs. +0.5 mg/d, *p*=0.002) increased more in the intervention group as compared with the control group. Consumption of butter-based spreads did not change in the intervention group but increased in the control group (−1 g/d vs. +2 g/d, *p*=0.002).

***Conclusion***: Individualized and family-based lifestyle intervention increased physical activity and enhanced diet quality in children.

**Disclosure of interest**: None to declare

### Program no. O201

#### Milk intake in middle-aged Norwegians and risk of hip fracture: is there an association? A linkage between the Norwegian Counties Study and the NOREPOS hip fracture database

##### Kristin Holvik^1^*, Haakon E. Meyer^1,2^, Ida Laake^3^, Tone K. Omsland^2^ and Anne J. Søgaard^1^
^1^Department of Chronic Diseases, Norwegian Institute of Public Health, Oslo, Norway; ^2^Institute of Health and Society, University of Oslo, Oslo, Norway; ^3^Department of Vaccines, Norwegian Institute of Public Health, Oslo, Norway *Presenting author

***Background and aims***: A meta-analysis of cohort studies found no association between milk intake and risk of hip fracture in women, and a suggestive (non-significant) protective association in men. In contrast, a study in two Swedish cohorts identified an increased risk of hip fracture with higher milk consumption in women, but no association in men.

***Methods***: We here use data from the third wave of the Norwegian Counties Study carried out in Finnmark, Oppland, and Sogn og Fjordane 1985–88. Diet was assessed by a semi-quantitative FFQ. Height and weight were measured, lifestyle variables were collected through questionnaires, and education level was obtained from Statistics Norway. Incident hip fractures during 1994–2013 were obtained by linkage to the NOREPOS hip fracture database. We performed Cox proportional hazards regression within the genders with frequency of milk consumption as the explanatory variable, adjusted for year of birth, body mass index, and daily smoking.

***Results***: Of 57,194 participants with mean age 45 who responded to the milk questions, 2,128 individuals suffered a hip fracture during 1994–2013 (median follow-up 27 years). Mean daily milk consumption was 3.7 glasses in men and 2.8 glasses in women. There was no significant association between number of glasses of milk consumed and risk of hip fracture in men: HR 0.96 (95% CI 0.91, 1.01) per glass of milk per day, nor in women: HR 1.03 (95% CI 0.98, 1.07) per glass of milk per day. Results were similar with additional adjustment for physical activity level, self-reported diseases, calcium and energy intake, and education level.

***Conclusion***: In line with a previous meta-analysis, we could not demonstrate any relationship between milk consumption and subsequent risk of hip fracture.

**Disclosure of interest**: None to declare

### Program no. O202

#### Vitamin D does not predict dementia or cognitive impairment – a 20-year follow-up study in community living old men

##### Erika K.E. Olsson^1^*, Liisa Byberg^2^, Brita Karlström^1^, Tommy Cederholm^1^, Per Sjögren^1^, Håkan Melhus^3^ and Lena Kilander^4^
^1^Department of Public Health and Caring Sciences, Uppsala, Sweden; ^2^Department of Surgical Sciences, Orthopedics, Uppsala, Sweden; ^3^Department of Medical Sciences, Clinical Pharmacogenomics and Osteoporosis, Uppsala, Sweden; ^4^Department of Public Health and Caring Sciences/Geriatrics, Uppsala University, Uppsala, Sweden *Presenting author

***Background and aims***: Vitamin D deficiency has been suggested as a possible risk factor for dementia and cognitive impairment. However, the results from longitudinal studies are contradictory. The aim was to investigate vitamin D, assessed by various methods, in relation to risk of cognitive impairments.

***Methods***: We measured vitamin D in 1,182, 71-year old Swedish men by plasma 25-hydroxyvitamin D (25(OH)D) with high-pressure liquid chromatography-mass spectrometry, dietary vitamin D intake by 7-day dietary record, and vitamin D synthesis genetic risk score (grs) according to Mendelian randomization. During 20 years of follow-up, 116, 64, and 250 men developed Alzheimer's disease, vascular dementia, or all-cause dementia, and another 80 men declined in the Mini Mental State Examination (MMSE). Hazard (HR) and odds ratio (OR) were calculated by Cox and logistic regression, respectively, and adjusted for potential confounders (age, season, body mass index, education, and physical activity).

***Results***: There were no associations between 25(OH)D or dietary vitamin D with any of the outcomes (crude and adjusted HR/OR 1.0 for all continuous exposures). When exposures were categorized, adjusted HR for all-cause dementia was 0.88 (95% CI, 0.59, 1.31) in men with ≤50 nmol/L plasma 25(OH)D versus men >75 nmol/l. Adjusted HR for all-cause dementia was 0.92 (95% CI, 0.63, 1.32) for the lowest tertile of vitamin D intake versus the highest tertile. The grs of vitamin D was not associated with any of the outcomes, e.g., adjusted HR for the continuous grs for all-cause dementia was 1.04 (95% CI, 0.91, 1.19).

***Conclusion***: In this sample of old men no association between vitamin D measured by plasma 25(OH)D, dietary vitamin D intake or genetic risk scoring, and long-term risk of Alzheimer's disease, all-cause dementia or cognitive impairment was found.

**Disclosure of interest**: None to declare

### Program no. O203

#### Replacing meat with fish in our diet – are there metabolic consequences beyond n-3 fatty acids?

##### Alastair Ross^1^*, Andrew Vincent^1^, Otto Savolainen^1^, Helen Lindqvist^2^, Ingrid Undeland^1^ and Ann-Sofie Sandberg^1^
^1^Food and Nutrition Science, Chalmers University of Technology, Gothenburg, Sweden; ^2^Department of Clinical Nutrition, Gothenburg University, Gothenburg, Sweden *Presenting author

***Background and aims***: Much focus around promoting fish intake is on the increased intake of n-3 fatty acids with fatty fish. There is little research that directly studies replacing meat with fish, and less still on what the broader consequences of this dietary change could be beyond changes to fatty acid profile and standard clinical biomarkers. As part of a broader research programme studying fish and health, we have used metabolomics to understand wider metabolic effects of replacing meat with herring.

***Methods***: We used GC-MS/MS metabolomics analysis on plasma to study the systemic effects of replacing meat with herring in two human studies – one postprandial study and one 4-week diet intervention study.

***Results***: There were clear changes (*p*<0.05) to the plasma metabolome between the herring and meat diets in both studies. In the 4-week intervention study, 22 metabolites changed, with notable changes to asparagine, ornithine, agmatine, glutamine, and glucosamine. In the postprandial study, 50 metabolites changed, with beef intake leading to greater postprandial response of branched chain amino acids, β-alanine and 4-hydroxyproline, while herring led to increases in docosahexaenoic acid and fatty acid C22:1 (cetoleic acid).

***Conclusion***: There are clear metabolic changes when replacing meat with herring beyond those commonly studied in intervention studies. Our research suggests that amino acid metabolism could be affected by the replacement of herring with meat. These results need to be validated in follow-up studies and suggest that health benefits from eating fish may be about much more than n-3 fatty acids.

**Disclosure of interest**: None to declare

### Program no. O204

#### Risk of myocardial infarction – impact of dietary exposure to persistent organic pollutants and intake of long-chain fish fatty acids: results from population-based prospective cohorts

##### Agneta Åkesson^1^*, Marika Berglund^1^, Anders Glynn^2^ and Alicja Wolk^1^
^1^Institute of Environmental Medicine, Karolinska Institutet, Stockholm, Sweden; ^2^Risk Benefit Assessment, Food Agency, Uppsala, Sweden *Presenting author

***Background and aims***: Cardiovascular disease (CVD) is the major cause of death and years of life lost globally. Consumption of fatty fish, rich in long-chain omega-3 fatty acids [eicosapentaenoic acid and docosahexaenoic acid (EPA-DHA)], is encouraged in dietary guidelines and may help prevent CVD. Fatty fish is, however, the main source of persistent organic pollutants such as polychlorinated biphenyls (PCBs) – a major food contaminant proposed to play a role in the etiology of CVD. We explored the associations of validated estimates of dietary PCB exposure and EPA-DHA intake with risk of myocardial infarction (MI) in two large prospective cohorts of middle-aged and elderly women and men.

***Methods***: We used the prospective population-based Swedish Mammography Cohort and the Cohort of Swedish Men with 33,446 and 36,759 cancer- and CVD-free women and men, respectively, at baseline (1997). Validated estimates of dietary PCB exposure and intake of fish fatty acids (eicosapentaenoic acid and docosahexaenoic acid or EPA–DHA) were obtained via a food frequency questionnaire. Incident cases of MI were ascertained through register linkage.

***Results***: During 12 years of follow-up, we ascertained 1,386 and 3,005 incident cases of MI in women and men, respectively. Dietary PCB exposure was associated with statistically significant 20–22% increased risk of MI before and 50–74% after adjustment for EPA–DHA comparing highest quartile/quintile with lowest. EPA–DHA, on the other hand, was only associated lower risk of MI (19–26%) after adjustment for dietary PCB exposure.

***Conclusion***: High dietary PCB exposure may increase the risk of MI in both sexes which in turn can compromise the beneficial effects of long-chain fish fatty acid intake.

**Disclosure of interest**: None to declare

### Program no. O205

#### Metabolic outcomes after an 8-week low-calorie-diet in overweight, pre-diabetic individuals: the role of gender in the PREVIEW study

##### Pia Christensen^1^, Mikael Fogelholm^2^, Margriet Westerterp-Plantenga^3^, Ian Macdonald^4^, J. Alfredo Martinez^5^, Svetoslav Handjiev^6^, Jennie Brand-Miller^7^, Sally Poppitt^8^, Wolfgang Schlicht^9^, Arne Astrup^1^, Kirsi Pietiläinen^2^, Mathijs Drummen^3^, Moira Taylor^4^, Santiago Navas-Carretero^5^, Teodora Handjieva-Darlenska^6^, Shannon Brodie^7^, Marta Silvestre^8^, Julia Thurn^9^, Thomas M. Larsen^1^ and Anne Raben^1^* ^1^Department of Nutrition Exercise and Sports Science, University of Copenhagen, Frederiksberg, Denmark; ^2^Department of Food and Environmental Sciences, University of Helsinki, Helsinki, Finland; ^3^Department of Human Biology, Nutrim, Maastricht University, Maastricht, Netherlands; ^4^Faculty of Medicine and Health Sciences, University of Nottingham, Nottingham, United Kingdom; ^5^Department of Physiology and Nutrition, University of Navarra, Pamplona, Spain; ^6^Clinical Center of Endocrinology, Medical University Sofia, Sofia, Bulgaria; ^7^School of Life and Environmental Sciences & Charles Perkins Centre, University of Sydney, Sydney, Australia; ^8^Human Nutrition Unit, School of Biological Sciences, University of Auckland, Auckland, New Zealand; ^9^Department of Sport and Exercise Science, University of Stuttgart, Stuttgart, Germany *Presenting author

***Background and aims***: The PREVIEW intervention study (www.previewstudy.com) is to date the largest, multinational study aiming to prevent T2D among pre-diabetic individuals with a combination of diet, physical activity, and behavior modification. Initially, all participants follow a low-calorie diet (LCD). The aim was to compare the effect of 8 weeks’ LCD on weight loss (WL) and metabolic outcomes between pre-diabetic men and women.

***Methods***: The participants received LCD (810 kcal daily) for 8 weeks (Cambridge Weight Plan^®^ – CWP). Data from participants who achieved 8% WL were included in the analysis. Two-sided t-tests were used throughout. Linear regressions were applied to test correlations.

***Results***: Of 2,326 individuals eligible for the LCD period, a total of 1,842 (79%) participants (1,225 women and 617 men) completed the WL phase successfully. At baseline, mean (±SD) age was 51.6±11.6 years, BMI 35.3±6.5 kg/m^2^, fasting plasma glucose (FPG) 6.2±0.7 mmol/L, and fasting serum insulin (FSI) 13.4±7.8 mU/L.

Average WL was 10.6±4.0 kg, with men losing 12.7±4.2 kg and women 9.6±3.4 kg (difference between gender, *p*<0.001). The men lost 11.7±3.5% of initial body weight while the women lost 10.2±3.1% (*p*<0.001). FPG decreased by 0.57±0.7 mmol/L in men, and by 0.37 ±0.6 mmol/L in women (*p*<0.001). FSI decreased by 5.8±7.4 mU/L in men and by 3.8±5.4 mU/L in women (*p*<0.001). The linear model showed an association of the WL% as well as gender on the changes in FPG and FSI.

***Conclusion***: An 8 weeks’ LCD intervention resulted in a marked decrease in body weight, FPG, and FSI among pre-diabetic subjects. Significantly larger decreases were seen in men versus women.

**Disclosure of interest**: P. Christensen: None to declare; M. Fogelholm: None to declare; M. Westerterp-Plantenga: None to declare; I. Macdonald: None to declare; J. A. Martinez: None to declare; S. Handjiev: None to declare; J. Brand-Miller: President of the Glycemic Index Foundation, a not-for-profit food endorsement programme, manager of a GI testing service at the University of Sydney and the co-author of books about the GI of foods; S. Poppitt: None to declare; W. Schlicht: None to declare; A. Astrup: Member of the International Carbohydrate Quality Consortium (ICQC) and the advisory boards for McCain Foods Limited, USA, McDonald's, USA, and Weight Watchers, USA. Principal investigator of current or recent research projects supported by grants from Arla Foods AMBA, DK, The Danish Dairy Research Foundation, DK, Global Dairy Platform, USA and the Danish Agriculture and Food Foundation, DK; K. Pietiläinen: None to declare; M. Drummen: None to declare; M. Taylor: None to declare; S. Navas-Carretero: None to declare; T. Handjieva-Darlenska; None to declare; S. Brodie: None to declare; M. Silvestre: None to declare; J. Thurn: None to declare; T. M. Larsen: None to declare; A. Raben: None to declare

### Program no. O206

#### Healthy Nordic diet and obesity: results from a 7-year follow-up in adult Finns

##### Noora Kanerva*, Niina Kaartinen, Mirkka Maukonen and Satu Männistö Department of Health, National Institution for Health and Welfare, Helsinki, Finland *Presenting author

***Background and aims***: The Nordic diet has decreased weight and waist circumference (WC) of overweight participants in randomized controlled trials. We aimed to investigate the long-term association between the Nordic diet and change in weight, BMI and WC among Finnish population.

***Methods***: The DILGOM study in 2007 was a population-based sample of adult Finns (25–75 years) (*n*=5,024). It included a health examination with measured height, weight and WC, and a validated 131-item food frequency questionnaire (FFQ). The follow-up was conducted in 2014 (*n*=3,772) similar to the baseline, using two groups: health examination for subjects from Southern Finland (*n*=1,314) and self-measurement and home-filled questionnaires for subjects from other regions (*n*=2,458). Self-reported anthropometrics were shown to be valid estimates of the measured ones. A dietary score illustrating a healthy Nordic diet (the Baltic Sea Diet Score; BSDS) was calculated. Higher BSDS indicated higher adherence to the diet. The final analysis included 1,400 men and 1,657 women. The association between baseline BSDS as well as changes in BSDS and changes in body anthropometrics during the follow-up were examined, using linear regression. The model was adjusted for age, sex, smoking status, physical activity, and baseline anthropometrics. Participants with diseases that may affect body weight (e.g., type 2 diabetes and cancer) were excluded.

***Results***: Higher baseline BSDS associated with decreased weight (β=−0.06; *P*=0.04) and BMI (β=−0.02; *P*=0.03). Change in BSDS during follow-up was also inversely associated with change in weight (β=−0.06; *P*=0.045) and BMI (β=−0.02; *P*=−0.4). BSDS did not associate with changes in WC in any of the analyses.

***Conclusion***: Higher adherence to a healthy Nordic diet as well as improving the adherence may enhance long-term weight maintenance in adult Finns.

**Disclosure of interest**: None to declare.

### Program no. O207

#### Dairy intake and mortality in Northern Sweden

##### Gianluca Tognon^1^*, Lena M. Nilsson^2^, Dmitry Shungin^3^, Lauren Lissner^1^, Jan-Håkan Jansson^4^, Frida Renström^5,6^, Maria Wennberg^7^, Anna Winkvist^7,8^ and Ingegerd Johansson^9^
^1^Section for Epidemiology and Social Medicine (EPSO), Department of Public Health & Community Medicine, University of Gothenburg, Gothenburg, Sweden; ^2^Arcum, Arctic Research Centre, University of Umeå, Umeå, Sweden; ^3^Department of Odontology, University of Umeå, Umeå, Sweden; ^4^Research Unit Skellefteå, Department of Public Health and Clinical Medicine, University of Umeå, Umeå, Sweden; ^5^Department of Biobank Research, University of Umeå, Umeå, Sweden; ^6^Department of Clinical Sciences, University of Lund, Malmö, Sweden; ^7^Department of Public Health and Clinical Medicine, Nutritional Research, University of Umeå, Umeå, Sweden; ^8^Department of Internal Medicine and Clinical Nutrition, Sahlgrenska Academy, University of Gothenburg, Gothenburg, Sweden; ^9^Department of Odontology, University of Umeå, Umeå, Sweden *Presenting author

***Background and aims***: There is inconclusive evidence about the association between dairy intake and mortality. A recent Swedish study showed a dose-response association between total milk intake and all-cause mortality, without stratifying by fat content. We studied the association of all-cause mortality with milk (total and by fat content) and other dairy product intakes in men and women from the Northern Sweden Diet Database.

***Methods***: 103,256 subjects (50.8% women) aged 25–75 from the Northern Sweden Diet Database were included (7,121 deaths, mean follow-up: 13.7, SD=6.8 years). We tested the association of all-cause mortality with intakes of milk (total or by fat content), fermented milk, cheese, and butter by Cox proportional hazard models, adjusted for age, sex, BMI, smoking status, education, physical activity, energy intake, and examination year. In sensitivity analyses, we further adjusted the models for lactose (as a proxy for galactose), intakes of vitamin D and calcium, diet quality, and excluded extreme intakes or the first 2 years of follow-up. Effect modification by all covariates was tested.

***Results***: High consumers of non-fermented milk and butter had a significantly higher mortality, whereas high consumers of cheese had a lower mortality than subjects with lower intakes. When analyzing milk intake stratified by fat content, the association was confirmed for full fat milk (HR=1.08, 95% CI: 1.05; 1.12). High-fat milk consumers reported lower intakes of unrefined cereals and vitamin C, lower education levels, higher physical activity levels, but a lower BMI compared to low- and medium-fat milk consumers. The sensitivity analyses confirmed all results and no clear effect modification was observed.

***Conclusion***: It is confirmed that non-fermented milk and butter predict all-cause mortality whereas cheese intake is a negative predictor in the Swedish population.

**Disclosure of interest**: None to declare

### Program no. O208

#### Adherence to dietary intervention among independently living individuals with elevated risk of dementia: Finnish Geriatric Intervention Study to prevent cognitive impairment and disability (FINGER)

##### Jenni Lehtisalo^1,2^*, Jaana Lindström^1^, Tiia Ngandu^1,3^, Tiina Laatikainen^1,4^, Timo Strandberg^5,6,7^, Jaakko Tuomilehto^1,2,8,9,10^, Hilkka Soininen^11,12^ and Miia Kivipelto^1,3,12,13^
^1^Department of Chronic Disease Prevention, National Institute for Health and Welfare, Helsinki, Finland; ^2^Department of Public Health, University of Helsinki, Helsinki, Finland; ^3^Center for Alzheimer Research, Karolinska Institutet, Stockholm, Sweden; ^4^Institute of Public Health and Clinical Nutrition, University of Eastern Finland, Kuopio, Finland; ^5^Institute of Health Sciences/Geriatrics, Oulu University Hospital, University of Oulu, Oulu, Finland; ^6^Department of Medicine, University of Helsinki, Helsinki, Finland; ^7^Geriatric Clinic, Helsinki University Hospital, Helsinki, Finland; ^8^South Ostrobothnia Central Hospital, Seinäjoki, Finland; ^9^Center for Vascular Prevention, Danube-University Krems, Krems, Austria; ^10^Diabetes Research Group, King Abdulaziz University, Jeddah, Saudi Arabia; ^11^Department of Neurology, Kuopio University Hospital, Kuopio, Finland; ^12^Institute of Clinical Medicine/Neurology, University of Eastern Finland, Kuopio, Finland; ^13^Aging Research Center, Karolinska Institutet-Stockholm University, Stockholm, Sweden *Presenting author

***Background and aims***: FINGER is a multicenter, randomized, controlled trial (NCT01041989) that demonstrated beneficial effects on cognition with a 2-year multidomain lifestyle intervention including nutritional guidance, exercise, cognitive training, and management of metabolic and vascular risk factors. In the present study, we investigate adherence to dietary intervention.

***Methods***: Independently living individuals aged 60–77 who were at risk of dementia were randomized into intervention (*n*=631) and control (*n*=629) groups. Goal of the dietary intervention was to achieve a healthy, varied diet meeting the Finnish dietary recommendations. The intervention comprised of both individual (3 times) and group counseling sessions (6 times). Data on dietary intakes were collected with a 3-day food records at baseline, after the first year, and at the end of the intervention.

***Results***: All 3 individual counseling sessions were participated by 84% of the intervention group, and at least 3 group sessions by 70%. Intake of saturated fatty acids was lower, and intakes of polyunsaturated fatty acids and fiber, as well as consumption of vegetables, were higher in the intervention group compared with the control group after both the first and the second year (*p*<0.05, respectively). Proportion of those consuming fish was also higher in the intervention group after the first year (*p*<0.01), but no longer after 2 years.

***Conclusion***: Dietary intervention among older participants is very feasible, and several beneficial dietary changes can be achieved simultaneously, also as a part of multidomain intervention. In addition to our goal, prevention of cognitive decline, these dietary changes could be advantageous in prevention and care of cardiovascular and diabetes-related conditions and in maintaining functional capacity among older adults.

**Disclosure of interest**: None to declare.

## III. Poster presentations

### Poster presentation no. P101

#### An examination of the food addiction status in adults: a pilot study

##### İzzet Ülker*, Birsen Yilmaz and Gamze Akbulut Nutrition and Dietetics, Faculty of Health Sciences, Gazi University, Ankara, Turkey *Presenting author

***Background and aims***: Food addiction is a topic discoursing frequently in recent years. Especially, the number of studies and publications regarding food addiction have shown a sharp increase in the scientific literature since early 2000s. Before the concept of food addiction, the first clinical trials have started in particular associated with excessive desire to some foods such as chocolate, sugar, and simple carbohydrate-rich foods. As long as obesity prevalence increases, overeating and binge eating behaviors are seen in the obese people.

***Methods***: This study was conducted on a total of 40 healthy volunteers living in Ankara, Turkey between March–April 2015. A questionnaire form was applied to the participants was including personal information, food frequency questionnaires, anthropometric measurements, eating behaviors, and the Yale Food Addiction Scale.

***Results***: The mean age of individuals was 30.9±11.67 years. According to body mass index (BMI), the half of the individuals was overweight. It was determined that the individuals had lost their controls to consume high fat and carbohydrate-rich foods such as chips, hamburgers, and chocolates While eating disorder behaviors were found significantly higher in men under 25 years of age, it was found similar in above 25 years individuals. It was determined food addiction in both obese men and women (33.3 and 20.0%, respectively). Moreover, in the half of women and 27.3% of the men skipping meal was confirmed food addiction.

***Conclusion***: In this study as similar other studies, consumption of certain foods cannot be controlled. There was a positive relationship between some food addiction and BMI. Skipping a meal can affect food addiction as well as factors such as lifestyle of individuals, genetic and neurobiological factors may be associated with food addiction and eating behavior. Further studies are needed to better understand the etiology of many chronic diseases, notably obesity.

**Disclosure of interest**: None to declare.

### Poster presentation no. P102

#### Dietary inflammation index and chronic diseases

##### İzzet Ülker* and Gamze Akbulut Nutrition and Dietetics, Faculty of Health Sciences, Gazi University, Ankara, Turkey *Presenting author

***Background and aims***: Dietary inflammation index (DII) is a tool that allows to evaluate the effects of the nutrients on inflammatory markers in the organism. Nutrients are defined as pro-inflammatory or anti-inflammatory mediators. After daily consumption of nutrients is calculated via the individual's food frequency/consumption, the anti-inflammatory or proinflammatory effects of the diet can be determined. This review is planned to evaluate the relationship between DII and the chronic diseases.

***Methods***: Many articles related with the topic have been scanned from the databases.

***Results***: Inflammation is a normal biological process involving the immune response. While normal inflammation response occurs against injury and infection, low-level chronic inflammation take places particularly in obese subjects in response to environmental factors. Also, the consumption of foods/nutrients permanently affects the inflammation via inflammatory precursors or anti-inflammatory mechanisms. If inflammation process is not controlled by normal negative feedback mechanism, low-level chronic inflammation may occur in the organism. The chronic inflammation process is associated with many chronic diseases, such as cancer, obesity, cardiovascular disease, etc. Adequate and balanced nutrition is one of the cornerstones of this process to prevent chronic inflammation. While Western-type diet increases inflammation markers, the Mediterranean-type diet decreases inflammation markers. In studies on relationship between DII scores were found significantly higher in study groups with chronic diseases such as breast cancer, cardiovascular diseases than the healthy control group.

***Conclusion***: Nutrition is an important environmental factor affecting the inflammation process. While Mediterranean-type dietary habits decrease inflammation markers, Western-type dietary habits increase inflammation markers. Adequate and balanced diet brings together a variety of foods can be reduced chronic inflammation.

**Disclosure of interest**: None to declare.

### Poster presentation no. P103

#### The life quality (QoL) of the patients with irritable bowel syndrome

##### Birsen Yilmaz* and Gamze Akbulut Nutrition and Dietetics, Faculty of Health Sciences, Gazi University, Ankara, Turkey *Presenting author

***Background and aims***: Irritable Bowel Syndrome (IBS) is a disorder characterized by gastrointestinal symptoms such as changes in bowel habits, abdominal pain, dyspepsia without any infection, organic or metabolic derangement. Although, the etiology of the disease has not been understood. IBS is diagnosed according to the Rome III criteria developed in 2006. The prevalence of the IBS is very common in the world ranged between 1.1 and 45%. This review was planned to evaluate QoL of the patients with IBS.

***Methods***: Many articles have been scanned from the databases related to the topic.

***Results***: IBS is one of the functional bowel diseases that severely reduce the QoL, although it does not affect mortality. The main purpose of medical treatment is abolishing factors which restrict daily activities of patients. Control of impaired motor activity of the intestine contributes to the improvement of symptoms related to the gastrointestinal tract. Moreover, patient-specific planning of medical nutritional therapy (including dietary fiber in the diet), pharmacological treatment, and psychological support have a paramount position in the treatment of the patients with IBS as well as to improve the QoL. When examined the studies in literature, it is seen that the QoL of the patients is lower than the general population. Besides the above-mentioned, it is reported that the training programs organized for patients increase QoL patients, also, reduce gastrointestinal symptoms. Vitamin D deficiency is often seen in patients as well as it is argued that low QoL may closely related with vitamin D. Additionally, although results from clinical trials are variables, the use of prebiotics, probiotics, and symbiotics may be useful for patients through their immune modulators and anti-inflammatory effects.

***Conclusion***: Eventually, the key aspect of improving QoL is multidisciplinary treatment approach. In this context, further studies are needed in order to enhance QoL of patients.

**Disclosure of interest**: None to declare.

### Poster presentation no. P104

#### The composition of buckwheat and its effects on health

##### Birsen Yilmaz*, İzzet Ülker and Gamze Akbult Nutrition and Dietetics, Faculty of Health Sciences, Gazi University, Ankara, Turkey *Presenting author

***Background and aims***: Buckwheat (BW) that is growing fast in the field with broad-leaves is an annual plant. It is classified in the Polygonaceae family, which consists of about 1,200 species of plants. The main structural difference separates the grains is a “dicotyledon.” This review was planned to evaluate composition of BW and its effects.

***Methods***: Many articles have been scanned from the databases related to the topic.

***Results***: There are numerous types of BW. *Fagopyrum esculentum* Moench and *Fagopyrum tataricum* Gaertn are the most common types of it. BW contains protein with a high nutritional value, significant levels of fiber, vitamins (in particular vitamins B_1_ and B_2_), and many other minerals. The main antioxidants of BW are rutin, quercetin, orient, and vitexin. Starch and fiber content of BW is almost the same as cereals. BW also contains basic polyunsaturated fatty acids such as linoleic acid in high levels. When compared with cereal, BW has a balanced amino acid composition due to containing high amounts of basic amino acids (in particular lysine, threonine, tryptophan) and the sulfur-containing amino acids. Antioxidants in BW such as a-tocopherol and phenolic compounds and flavonols affect positively the activity and development of *Lactobacillus* and *Bifidobacteria* in the colon. BW is a substantial food for patients with celiac disease as it does not contain gluten. And also, it is frequently used in the food industry. Recently, developed gluten-free foods (bread, pasta, etc.) in the world are produced from pseudo-cereals such as BW.

***Conclusion***: It has many positive effects on the human health due to the rich nutrient content. According to the studies, BW is referred with “anticancer,” “antidiabetic,” and “anti-inflammatory” effects. Besides, it is discussed that it may be reducing plasma cholesterol levels, body weight, and developing cognitive functions. Further studies are needed to better understanding the mechanism of BW in the organism and the bioactive components.

**Disclosure of interest**: None to declare.

### Poster presentation no. P105

#### Gastro-intestinal graft-versus-host disease (GI GVHD): a guide for recent dietary therapy

##### Gamze Akbulut, İzzet Ülker* and Birsen Yilmaz Nutrition and Dietetics, Faculty of Health Sciences, Gazi University, Ankara, Turkey *Presenting author

***Background and aims***: Graft versus host disease (GVHD) is a common complication of allogeneic hematopoietic stem cell transplantation (SCT) that has high morbidity and mortality ratios. This review was planned to evaluate GVHD and its dietary therapy.

***Methods***: Many articles have been scanned from the databases related to the topic.

***Results***: Acute GVHD (aGVHD), which usually develops in the first 3 months after transplantation is a clinic-pathological syndrome mainly involving skin, liver, and gut. Immunosuppressive therapy is started before transplantation to prevent development of GVHD. A special stage and grading system developed for aGVHD is used to propose treatment. High dose corticosteroid is the first line treatment. Supportive therapy improves quality of life and may affect response to treatment positively. Chronic GVHD (cGVHD) usually appears after 3 months post-transplant. cGVHD which is the most important cause of mortality after SCT except late relapses, needs multidiscipliner approach for treatment. Infection is the most common mortality cause in patients with cGVHD. For this reason antimicrobial drugs should be given during immunosuppressive treatment. In recent years, different prevention and treatment methods had been developed in the light of new data about both acute and cGVHD pathogenesis. As survival rates have improved, there has been an increased focus on supportive care. Nutrition is a supportive-care modality that has been associated with improved tolerance to chemo/radio therapy, improved survival, increased life quality, and decreased risk of infection in patients undergoing GVHD therapy.

***Conclusion***: The gastrointestinal (GI) tract is one of the major organs affected by GVHD. The GI GVHD diet is a four-phase progressive diet, which aims to maximize oral intake and minimize stooling. An appropriate diet level for the GVHD patient is determined by his/her gut function.

**Disclosure of interest**: None to declare.

### Poster presentation no. P106

#### The importance of nutritional status in multiple sclerosis

##### Gamze Akbulut, Birsen Yilmaz* and İzzet Ülker Nutrition and Dietetics, Faculty of Health Sciences, Gazi University, Ankara, Turkey *Presenting author

***Background and aims***: Multiple sclerosis (MS) is an inflammatory demyelinating disease of the central nervous system that causes neurological impairment, which mainly affects young adults. MS is rare in tropical areas, but quite common in developed countries. The distribution of the prevalence and incidence of MS is more complex and uneven than previously supposed and little is known about the wide variations among different ethnic groups in any country and areas at the same latitudes. MS is more common among women than men. This review was planned to evaluate importance of nutritional status in MS.

***Methods***: Many articles have been scanned from the databases related to the topic.

***Results***: Nutritional status and dietary habits in MS patients have not been extensively studied or reported, however individual findings suggest that many patients suffer from various forms of malnutrition. The assessment of nutritional status is essential for a diagnosis of nutritional compromise and for the multidisciplinary management required. The assessment of body mass index (BMI) on the other hand has not proven to be a good indicator of nutritional status. These are acute phase proteins, a situation that would alter specificity for diagnosis of visceral protein malnutrition. Albumin and prealbumin, although widely used, should be used with caution. Increased plasma levels of prealbumin with a short half-life- can indicate adequate response to nutritional support. Neurobehavioral and cognitive functions are related with vitamin B_12_ status. MS patients generally present a decrease of neuroprotective and immunoregulatory vitamins and an increase of total homocysteine concentrations.

***Conclusion***: Further studies are needed to prevent malnutrition and provide an adequate and balanced diet for patients with MS.

**Disclosure of interest**: None to declare.

### Poster presentation no. P107

#### Inpatient's satisfaction with hospital food

##### Áróra R. Ingadóttir^1,2^*, Heiða B. Hilmisdóttir^3^ and Ingibjörg Gunnarsdóttir^1,2,4^
^1^Unit for Nutrition Research, University of Iceland, Landspitali National University Hospital, Reykjavík, Iceland; ^2^Nutrition Department, Landspitali National University Hospital, Reykjavík, Iceland; ^3^Hospital Food and Nutrition Services, Landspitali National University Hospital, Reykjavík, Iceland; ^4^Faculty of Food Science and Human Nutrition, University of Iceland, Reykjavík, Iceland *Presenting author

***Background and aims***: Past studies suggest that energy- and protein intake of surgical patients at Landspítali is inadequate. Hospital food is important for most hospitalized patients, and satisfaction with the food increases the likelihood that patients are better nourished. The aim was to investigate the satisfaction of inpatient hospital food after the implementation of new hospital menus and changes in the kitchen's food order system, which allows patients to choose between different meals.

***Methods***: Patients (*n=*93) admitted to the Department of Cardio Thoracic Surgery at Landspitali the National University Hospital in Iceland, during the period August to December 2013. Satisfaction with the hospital food was assessed with selected questions from the English National Inpatient survey. Energy and protein intake were estimated using a validated plate diagram sheet.

***Results***: Most patients rated the hospital food as very good or good (87%). Only 11% were offered to choose between meals from the menu. The average energy intake was 1,452±389 kcal/day, and the average protein intake was 60±17 g/day, both considerably below estimated requirements (1,953±265 kcal and 82±9 g). However, one-quarter thought they were served too much food.

***Conclusion***: The results suggest that it is unlikely that inadequate energy and protein intake of surgical patients at Landspítali is due to dissatisfaction with the food offered in the hospital. It is necessary to implement individualized service of meals and snacks as appropriate, in accordance to the clinical guidelines on patients’ nutrition, to prevent malnutrition during hospital stay.

**Disclosure of interest**: None to declare.

### Poster presentation no. P108

#### Plasma alkylresorcinol concentrations as a novel measure of compliance to a gluten-free diet

##### Mads Lind^1,2^*, Mia L. Madsen^3^, Jüri Rumessen^4^, Henrik Vestergaard^3^, Rikke J. Gøbel^3^, Torben Hansen^3^, Lotte Lauritzen^2^, Oluf B. Pedersen^3^, Mette Kristensen^3^ and Alastair Ross^1^
^1^Department of Biology and Biological Engineering, Chalmers University of Technology, Gothenburg, Sweden; ^2^Department of Nutrition, Exercise and Sports, Faculty of Science, University of Copenhagen, Frederiksberg, Denmark; ^3^The Novo Nordisk Foundation Center for Basic Metabolic Research, Section of Metabolic Genetics, Faculty of Health and Medical Sciences, University of Copenhagen, Copenhagen, Denmark; ^4^QD-Research Unit and Department of Gastroenterology, Herlev and Gentofte Hospital, Copenhagen, Denmark *Presenting author

***Background and aims***: Many celiac disease (CD) patients experience difficulties in adherence to a gluten-free diet. Methods for testing compliance to a gluten-free diet are costly and cumbersome. A simple biomarker of gluten intake is needed in a clinical setting and will be useful for epidemiological studies investigating wider effects of gluten intake. The aim was to evaluate plasma total alkylresorcinols (AR) levels as a measure of gluten intake.

***Methods***: This randomized, controlled, cross-over intervention study in 52 Danish adults with features of the metabolic syndrome compared 8 weeks gluten-rich and gluten-poor diet separated by a wash-out period of at least 6 weeks. We measure fasting plasma concentrations of AR to determine if they reflected differences in gluten intake. Additionally, we investigated in 118 Danish adults the cross-sectional association between self-reported gluten intake and plasma AR in this and a similar study at baseline.

***Results***: Total plasma AR decreased more during the gluten-poor period compared to the gluten-rich period –124.8 (–156.5; –93.0) vs. –31.8 (–63.1; –0.4) nmol/L, respectively, *p<*0.001. Based on the plasma AR-profile, we built a classification tree to objectively determine compliance and found an overall misclassification error of participants of 3.9%. The cross-sectional study showed a 5.6% (2.4; 8.9) increase in total plasma AR per 1 g-increase in reported gluten intake (*p*<0.001).

***Conclusion***: We propose plasma AR as a new tool to monitor compliance to a gluten-free diet to support clinical work with CD, as well as to help investigations on the possible effects of gluten in the wider population.

**Disclosure of interest**: M. Lind: Partly supported by an unrestricted grant from Cereal Partners Worldwide a joint venture between Nestlé SA and General Mills Ltd.; M. L. Madsen: None to declare; J. Rumessen: None to declare; H. Vestergaard: None to declare; R. J. Gøbel: None to declare; T. Hansen: None to declare; L. Lauritzen: None to declare; O. B. Pedersen: None to declare; M. Kristensen: None to declare; A. Ross: None to declare.

### Poster presentation no. P109

#### The comparison of cardiometabolic risk factors and the prevalence of metabolic syndrome (MetS) among pre-menopausal (pre-M) and post-menopausal (post-M) women

##### Gamze Akbulut, Birsen Yilmaz and İzzet Ülker* Nutrition and Dietetics, Faculty of Health Sciences, Gazi University, Ankara, Turkey *Presenting author

***Background and aims***: The aims of this study were the estimation and comparison of cardiometabolic risk factors and the prevalence of Metabolic Syndrome (MetS) among pre-menopausal (pre-M) and post-menopausal (post-M) women.

***Methods***: This study was conducted on total of 664 (pre-M: 378, post-M: 286) women, aged between 30–64 years. Body weight, waist circumference (WC) were taken from all participants, body mass index (BMI) and waist hip ratio (WHR) were calculated, and lipid profiles were investigated.

***Results***: The mean ages of pre-M women and post-M women were found 42.1±4.50 and 56.2±4.19 years, respectively (*p*<0.001). The mean fasting plasma glucose (FPG), low density lipoprotein cholesterol (LDL-C), triglycerides (TG), and total cholesterol/high density lipoprotein cholesterol (TC/HDL-C) were significantly lower in pre-M women (*p*<0.05) and TC was significantly higher in post-M women (*p<*0.001).

***Conclusion***: Menopause is an important risk factor for metabolic syndrome. MetS was more prevalent among postmenopausal women than among premenopausal women by both criteria.

**Table 1:** Clinical characteristics and biochemical characteristics of the studied population

	pre-M (*n*=378) (±SD)	post-M (*n*=286) (±SD)
BMI (kg/m^2^)	27.3±5.58	28.0±6.27
WC (cm)	88.2±14.60	93.3±13.77**
Waist/hip	0.73±0.10	0.87±0.11**
SAP (mm Hg)	124.8±19.17	136.1±20.71**
FPG (mg/dL)	100.0±37.3	109.0±44.84*
LDL-C (mg/dL)	124.2±39.94	132.0±43.94*
HDL-C (mg/dL)	44.5±10.00	46.1±12.43
TG (mg/dL)	157.0±71.75	172.5±95.43*
TC/HDL-C	4.7±1.64	4.9±1.6*

Values are expressed as mean±standard deviation.

* *p<*0.05

** *p*<0.001.

**Disclosure of interest**: None to declare.

### Poster presentation no. P110

#### Replacement of dietary SFAs with PUFAs upregulates the mRNA expression levels of the LDL receptor and liver X receptor alpha: a double-blind randomized controlled trial

##### Lena Leder^1^*, Stine M. Ulven^1,2^, Inger Ottestad^1,2^, Jacob J. Christensen^1,2^, Vibeke H. Telle-Hansen^3^, Linda Granlund^3^, Lene F. Andersen^1^ and Kirsten B. Holven^1,4^
^1^Department of Nutrition, University of Oslo, Oslo, Norway; ^2^Department of Health, Nutrition and Management, Oslo and Akershus University College of Applied Sciences, Oslo, Norway; ^3^Mills DA, Oslo, Norway; ^4^Norwegian National Advisory Unit on Familial Hypercholesterolemia, Oslo University Hospital, Oslo, Norway *Presenting author

***Background and aims***: Solid evidence indicates that replacing saturated fatty acids (SFAs) with polyunsaturated fatty acids (PUFAs) reduces total cholesterol (total-C) and LDL cholesterol (LDL-C) and thereby coronary heart disease events. The molecular mechanisms of the LDL-C lowering effects are, however, not completely elucidated. To further understand the molecular mechanisms, we examined the gene expression level of lipid-related genes in peripheral blood mononuclear cells (PBMCs) in a human dietary intervention study.

***Methods***: In an 8-week double-blinded study, healthy adults (*n*=95) aged 25–70 years with moderate hypercholesterolemia were randomly assigned to *an* experimental diet group low in SFAs but high in n-6 PUFAs (Ex-group) or a control diet group high in SFAs but low in n-6 PUFAs (C-group). PBMCs were isolated at baseline and end of the study, and the mRNA gene expression analysis was performed using TaqMan Array Micro Fluidic Cards (Applied Biosystems) for RT-qPCR amplification.

***Results***: Exchanging SFAs with PUFAs reduced plasma total-C and LDL-C, increased LDL receptor (*LDLR)*, liver X receptor alpha (*LXRA)*, fatty acid synthase *(FASN)*, and ATP binding cassette subfamily G member 1 *(ABCG1)* mRNA expression and reduced uncoupling protein 2 *(UCP2)* mRNA expression in PBMCs.

***Conclusion***: The main effect of replacing SFAs with PUFAs seems to be mediated through an upregulation of *LDLR* mRNA expression, subsequently increasing the mRNA expression of *LXRA* and *LXRA* target genes.

**Disclosure of interest**: None to declare.

### Poster presentation no. P111

#### Effectiveness of moderate vitamin D3 supplementation among Finnish and Somali women

##### Folasade A. Adebayo^1^*, Suvi Itkonen^1^, Taina Öhman^1^, Essi Skaffari^1^, Elisa Saarnio^1^, Maijaliisa Erkkola^1^, Kevin Cashman^2^ and Christel Lamberg-Allardt^1^
^1^Department of Food and Environmental Sciences, University of Helsinki, Helsinki, Finland; ^2^School of Food and Nutritional Sciences, University College Cork, Cork, Ireland *Presenting author

***Background and aims***: Current vitamin D intake recommendations in the Nordic countries are based on studies in Caucasian populations. However, there can be difference in vitamin D requirement and metabolism between different population groups. In Somali immigrants, alarmingly low serum 25-hydroxyvitamin D (S-25OHD) was observed in Finland. We aimed to evaluate ethnic differences in response of S-25OHD to vitamin D supplementation among Somali (East-African) and Finnish (Caucasian) women.

***Methods***: The study was a 6-month, double-blinded, placebo-controlled randomized trial conducted during the winter period (2014–2015) in the Helsinki area (latitude 60°N). A total of 54 Somali women and 71 Finnish women, aged 21–64, were randomized into three groups (placebo, 10 or 20 µg vitamin D_3_/d). Serum samples for total S-25OHD (25OHD_2_+25OHD_3_) assessment with liquid chromatography-tandem mass spectrometry were taken at baseline, midpoint, and endpoint of the intervention (at 0/3/6 months). Data were analyzed by repeated measures analysis of covariance (adjusted for baseline S-25OHD).

***Results***: Baseline mean S-25OHD concentrations were higher among Finns (60.5 nmol/l) than among Somalis (52.2 nmol/l) (*p*=0.006). Vitamin D supplementation with both 10 µg and 20 µg dosages was effective in each ethnic group (Finns *p*<0.001, *p*<0.001; Somali *p*=0.003, *p*<0.001). Compared to baseline S-25OHD, the absolute mean increases in S-25OHD with 10 µg dose for Finns and Somalis were +8.4 nmol/l and +10.1 nmol/l, respectively, while mean increases with 20 µg dose were +10.6 nmol/l and +17.0 nmol/l, respectively. The absolute mean increases in S-25OHD were slightly higher among Somalis but not statistically significant.

***Conclusion***: Moderate vitamin D_3_ supplementation was effective in increasing S-25OHD in both Finnish and Somali women during the 6 month intervention. However, there were no ethnic differences in response to vitamin D_3_ supplementation between the two groups.

**Disclosure of interest**: None to declare.

### Poster presentation no. P112

#### Factors influencing the choice of oral nutritional supplements prescribed by Swedish dietitians

##### Sara Sjöström^1^, Anna-Karin Snögren^2^, Afsaneh Koochek^3^ and Evelina Liljeberg^4^* ^1^Primary healthcare Västmanland, Västerås, Sweden; ^2^County council of Dalarna, Falun, Sweden; ^3^Department of Public Health and Caring Sciences, Uppsala University, Uppsala, Sweden; ^4^Department of Food, Nutrition and Dietetics, Uppsala University, Uppsala, Sweden *Presenting author

***Background and aims***: Oral Nutritional Supplements (ONS) are commonly used in combination with dietary changes when treating disease-related malnutrition. In Sweden ONS are primarily prescribed by registered dietitians and subsidized by county councils, and there are no standardized guidelines for prescription. The aim of this study was to examine factors influencing the choice of ONS prescriptions.

***Methods***: A newsletter with a link to an online survey was e-mailed to 1,480 members of The Swedish Association of Clinical Dietitians (DRF) as well as to other dietitians with contact details on the DRF's webpage.

***Results***: A total of 133 dietitians, from 17 out of 21 county councils working in various settings, answered the survey. When asked to choose from alternatives of important influencing factors for ONS prescription the three most frequently selected were, patient taste preferences (96%), nutritional requirements (93%), and medical diagnosis (85%). Those were followed by, dietitian's previous experiences of successful ONS (76%), local procurements (62%), food allergy and intolerance (53%), price (23%), dietitian taste preferences (15%), and other alternatives (8%). Over half (52%) of the dietitians rated patient taste preferences as the single most influential factor when prescribing ONS. Although, 51% stated that patients were seldom or otherwise not provided with the opportunity to taste ONS before prescription. One-third (35%) of the dietitians stated that representatives from ONS companies impact their choice for prescription.

***Conclusion***: Patient taste preferences and nutritional requirements were found to be the most important influencing factors for prescribing ONS. In conjunction, it was also found that many patients seldom get the opportunity to try ONS before prescription.

**Disclosure of interest**: None to declare.

### Poster presentation no. P113

#### Glucan from yeast reduced mast cell-induced hyperpermeability in follicle -associated - and villus epithelium from ileal specimens ex vivo

##### John-Peter G. Mall^1^*, Martin T. Winberg^2^, Ida Schoultz^1^, Robert Brummer^1^ and Åsa Keita^2^
^1^School of Medical Sciences, Nutrition-Gut-Brain Interactions Research Centre, Örebro University, Örebro, Sweden; ^2^Division of Clinical Sciences, Department of Clinical and Experimental Medicine Linköping University, Linköping, Sweden *Presenting author

***Background and aims***: Crohn's disease (CD) is characterized by increased gut permeability and chronic inflammation. Our objective was to investigate whether a specific -glucan from yeast was able to counteract mast cell-induced hyperpermeability in ileal specimens from subjects with and without CD, mounted in Ussing chamber.

***Methods***: The effect of -glucan on intestinal permeability was examined in follicle-associated epithelium (FAE) and villus epithelium (VE) from 9 control subjects without IBD and 2 patients with CD. Twelve ileal specimens (obtained after surgery) per subject were mounted in the Ussing chambers. The biopsies were pre-treated with 0.5 mg/ml yeast-derived -glucan for 20 minutes before the mast cell degranulating chemical Compound (C) 48/80 (10 ng/ml) was added. Unstimulated biopsies were used as control. FITC-dextran 4000 and horseradish peroxidase (HRP) were used to measure paracellular and transcellular permeability, respectively. Non-parametric statistical analysis was applied.

***Results***: Administration with C48/80 resulted in nearly 3 times higher permeability compared to control. Treatment with -glucan resulted in a significant reduction of C48/80's effect in both FAE and VE on paracellular permeability (*p*<0.01) and in VE on transcellular permeability (*p*<0.05) in the non-IBD subjects. The results from the two patients with CD followed a similar pattern.

***Conclusion***: Our results showed that -glucan from yeast can reduce C48/80-induced permeability increase in FAE (paracellular) and VE (para – and transcellular) from surgical ileal specimens. Our preliminary results from two patients with CD showed a similar pattern, but more patients are needed to confirm this. These results are part of an ongoing study and could reveal -glucan from yeast as a potential therapeutic agent for patients with CD.

**Disclosure of interest**: None to declare.

### Poster presentation no. P114

#### Single nucleotide polymorphisms in the FADS gene cluster but not the ELOVL2 gene are associated with serum polyunsaturated fatty acid composition and development of allergy (in a Swedish Birth Cohort)

##### Malin Barman^1^*, Staffan Nilsson^2^, Åsa T. Naluai^3^, Anna Sandin^4^, Agnes Wold^5^ and Ann-Sofie Sandberg^1^
^1^Department of Biology and Biological Engineering, Chalmers University of Technology, Gothenburg; ^2^Department of Mathematical Sciences, Chalmers University of Technology, Gothenburg; ^3^Department of Microbiology and Immunology, Institute of Biomedicine, Gothenburg University, Gothenburg, Sweden; ^4^Department of Clinical Sciences, Paediatrics, Umeå University, Umeå, Sweden; ^5^Department of Infectious Diseases, Institute of Biomedicine, University of Gothenburg, Gothenburg, Sweden *Presenting author

***Background and aims***: Polyunsaturated fatty acid (PUFA) composition in phospholipids may affect the risk of developing allergies. Long chain PUFAs can be produced in the body in a reaction pathway catalyzed by desaturases and elongases. The aim was to investigate if polymorphisms in the *FADS* gene cluster and the *ELOVL2* gene were associated with the proportions of PUFAs in serum phospholipids from newborn infants and adolescents or with allergy development in a Swedish birth cohort.

***Methods***: Genotyping was performed in 211 subjects selected from a population based birth cohort in northern Sweden. Four SNPs were analyzed based on previous genome-wide association studies: rs102275 and rs174448 (*FADS* gene cluster) and rs2236212 and rs17606561 (*ELOVL2* gene). Proportions of PUFAs in cord and adolescent serum were available for 118 and 120 subjects, respectively.

***Results***: Minor allele carriers of rs102275 and rs174448 had decreased proportions of 20:4 n-6 in cord and adolescent serum and increased proportions of 20:3 n-6 in cord serum as well as a reduced risk of developing atopic eczema, but not respiratory allergy, at 13 years of age. Similarly, minor allele carriers of rs17606561 had decreased proportions of 20:4 n-6 in cord serum. Polymorphism in the *ELOVL2* gene was not associated with allergy development.

***Conclusion***: Reduced capacity to desaturase n-6 PUFAs due to *FADS* polymorphisms was associated with reduced risk for eczema development, which could indicate a pathogenic role for long-chain PUFAs in allergy development.

**Disclosure of interest**: None to declare.

### Poster presentation no. P115

#### Adding a dietitian to a follow-home team after discharge of geriatric patients at nutritional risk is cost-effective

##### Kerstin Belqaid^1^*, Christopher Brandt^2^, Kerstin Lugnet^3^, Anni L. Nielsen^4^, Anne Pohju^5^, Henrik H. Rasmussen^6^, Nanna M.L. Rasmussen^7^ and Anne M. Beck^8^
^1^Department of Learning, Informatics, Management and Ethics, Karolinska Institutet, Stockholm, Sweden; ^2^Department of Medical Gastroenterology, Rigshospitalet, Copenhagen, Denmark; ^3^Department of Public Health and Caring Sciences, Uppsala University, Uppsala, Sweden; ^4^Department of Oncology, Herlev University Hospital, Herlev, Denmark; ^5^Department of Clinical Nutrition, Helsinki University Hospital, Helsinki, Finland; ^6^Centre for Nutrition and Bowel Disease, Aalborg University Hospital, Aalborg, Denmark; ^7^Department of Clinical Nutrition, Regional Hospital West Jutland, Holstebro, Denmark; ^8^Clinical Nutrition Research Unit, Herlev and Gentofte University Hospital, Gentofte, Denmark *Presenting author

***Background and aims***: A previously published Danish study reported reduced number of re-admissions to hospital among geriatric patients when a dietitian was added to an already established follow-home team. The aim of this study was to investigate cost-effectiveness of the intervention compared to the control group (CG).

***Methods***: Participants, elderly patients at nutritional risk, were at discharge randomly allocated to receive a follow-home team either with a dietitian (intervention group, IG) or without dietitian (CG). The IG received three home visits by the dietitian during a 12-week period. Data included in the economic analysis was number of days in hospital (HD) at an estimated cost of €535/bed/day, use of oral nutritional supplements (ONS) at an estimated amount of one supplement/day at a cost of _2.5/day and time spent by the dietitian (2 h/visit at a cost of €31.5/h).

***Results***: Of the 71 patients included, 34 were in the IG. Of these, 30 patients received the three planned visits. Admission rate after 26 weeks was 28% in the IG and 52% in the CG (*p*=0.074). Cumulated number of re-admission days in the IG was 172 HD and 415 HD in the CG (*p*=0.015). The prevalence of the use of ONS was 48% in the IG and 14% in the CG (*p*=0.001). Estimated cost for the additional dietitian and ONS combined in the IG was €6,426, compared to €1,150 (ONS only) in the CG. Estimated cost for the hospital admissions was €85,594 in the IG and €220,875 in the CG. Based on these estimations, cost savings add up to €3,048 per patient in the IG.

***Conclusion***: This study suggests that the addition of a dietitian to a follow-home team for geriatric patients discharged from hospital could be cost-effective in terms of reducing re-admissions.

**Disclosure of interest**: None to declare.

### Poster presentation no. P116

#### Intake of regular-fat cheese does not affect risk markers of the metabolic syndrome differently compared to reduced-fat cheese

##### Farinaz Raziani^1^, Tine Tholstrup^1^, Marlene D. Kristensen^2^, Matilde L. Svanegaard^1^, Christian Ritz^1^, Arne Astrup^1^ and Anne Raben^1^* ^1^Department of Nutrition, Exercise and Sports, Science, University of Copenhagen, Copenhagen, Denmark; ^2^Novo Nordisk, Hillerød, Denmark *Presenting author

***Background and aims***: Consumption of regular-fat cheese (REG) contributes significantly with cholesterol-rising saturated fat. Therefore, dietary guidelines recommend consumption of REG as opposed to REG. The aim was to compare a high daily intake of REG to an equal amount of reduced-fat cheese (RED) and a carbohydrate control (CHO) on metabolic syndrome (MetS) risk factors in a metabolically vulnerable population.

***Methods***: The study was a 12-week randomized parallel intervention preceded by a 2-week run-in. A total of 47 men and 92 women were randomly allocated to REG (*n*=45), RED (*n*=48) or CHO (*n*=46) diets. Mean±SD for age and BMI was 53.8±12.2 y and 28.7±3.6 kg/m^2^, respectively. Subjects iso-calorically replaced part of their habitual diet with 80 g cheese or 90 g bread+25 g jam per 10 MJ. Blood samples and 3-day dietary records were taken at week 0 and 12. Statistical analyses were done by linear mixed models.

***Results***: There were no differences in the primary LDL-cholesterol or secondary outcomes triacylglycerol, glucose, or insulin concentrations between the diets. Total cholesterol did not differ between the REG and RED diets, but tended to be higher for the REG diet compared to the CHO diet (0.21±0.12, *p*=0.08). HDL-cholesterol did not differ significantly between the REG and RED diets, but tended to increase on the REG diet compared to the CHO diet (0.06±0.03, *p=*0.06).

***Conclusion***: A high daily intake of REG for 12 weeks did not alter MetS risk factors differently compared to an equal intake of RED in a metabolically vulnerable population.

**Disclosure of interest**: F. Raziani: None to declare; T. Tholstrup: None to declare; M. D. Kristensen: None to declare; M. L. Svanegaard: None to declare; C. Ritz: None to declare; A. Astrup: Member of the International Carbohydrate Quality Consortium (ICQC) and the advisory boards for McCain Foods Limited, USA, McDonald's, USA and Weight Watchers, USA. Principal investigator of current or recent research projects supported by grants from Arla Foods AMBA, DK, The Danish Dairy Research Foundation, DK, Global Dairy Platform, USA and the Danish Agriculture and Food Foundation, DK; A. Raben: None to declare.

### Poster presentation no. P117

#### Nutrition in care homes: the effect of nutritional screening, monitoring, and action plans for elderly residents

##### Mette M. Maibom* Health and Elder Care, Lolland Municipality, Maribo, Denmark *Presenting author

***Background and aims***: Elderly residents in Danish care homes tend to experience unintentional weight loss. Studies have shown that 42% of the elderly in Danish care homes experience a 1% weight loss or more in a period of 6 months. Weight loss may result in depression, a lower level of body function and a higher risk of diseases and death. Consequently, the weight loss may also lead to increased care home expenses.

The aim of this study was to evaluate the nutritional screening, planning, and monitoring of care home residents in Denmark and its effects on their body weight maintenance.

***Methods***: Health care workers are responsible for the nutritional screening of care home residents corresponding to The Danish National Board of Social Services’ recommendations as well as for planning and monitoring the residents’ nutritional intake corresponding to the local government's food and meal policy action plan.

The study was designed as a longitudinal study with three data collection points at baseline and at 3 and 6 months post baseline. A total of 333 Danish care home residents (mean age 83, 65% women) in 11 Danish care home facilities participated in the study.

***Results***: About 92.2% of the participants were screened corresponding to The Danish National Board of Social Services recommendation for nutritional screening. About 11.4% and 16.1% of the participants lost more than 1.5 kg over a period of 3 and 6 months, respectively.

***Conclusion***: Nutritional screening, monitoring, and action plans have documented effects on maintaining the body weight of elderly residents in Danish care homes when health care workers are responsible for implementing the actions.

**Disclosure of interest**: None to declare.

### Poster presentation no. P118

#### The cheese-matrix fat content is important for blood lipids, fecal energy excretion, and microbiome in pigs

##### Tanja Thorning^1^*, Anne Raben^1^, Nathalie Bendsen^2^, Henry Jørgensen^3^, Pia Kiilerich^4^, Ylva Ardö^5^, Janne Lorenzen^1^, Karsten Kristiansen^4^ and Arne Astrup^1^
^1^Department of Nutrition, Exercise and Sports, University of Copenhagen, Frederiksberg, Denmark; ^2^Novo Nordisk A/S, Søborg; ^3^Department of Animal Science, University of Aarhus, Tjele, Aarhus, Denmark; ^4^Department of Biology, University of Copenhagen, Copenhagen, Denmark; ^5^Department of Food Science, University of Copenhagen, Frederiksberg, Denmark *Presenting author

***Background and aims***: Cheese and butter have been shown to affect blood lipids differently. The aim of the study was to compare the effect of diets with regular-fat cheese, reduced-fat cheese+butter or butter on fasting blood lipids, fecal fat and energy excretion, and gut microbiome.

***Methods***: A parallel-arm, randomized, controlled intervention study was conducted in 36 LYDD growing sows. A 14-day butter-rich run-in diet was followed by a 14-day intervention on macronutrient-matched diets: regular-fat (25% w/w) cheese (35.3 g/100 g diet) (REG), reduced-fat (13% w/w) cheese (35.3 g/100 g diet)+butter (5.2 g/100 g diet) (RED), or butter (10.6 g/100 g diet) (BUT). Blood was drawn before and after the intervention and feces collected the last 48 h. Statistical analyses for blood lipids, fecal fat and energy were performed using ANCOVA. Microbiome was analyzed with Bonferroni adjusted t-tests.

***Results***: There were no differences in LDL-cholesterol or triglycerides between groups after the intervention. Total cholesterol and HDL-cholesterol were higher in the REG group compared to the BUT group, but only tended to be higher in the RED group. Compared to the BUT group, the REG and RED groups had higher fecal fat excretion, whereas fecal energy excretion was only significantly higher in the REG group. The higher fecal energy excretion in the REG group correlated with a lower microbiome *Firmicutes*-to-*Bacteroidetes* ratio.

***Conclusion***: Dairy fat consumed as cheese or butter caused different cardio-metabolic effects. The differences between reduced-fat cheese+butter and butter were less pronounced than those between regular-fat cheese and butter, suggesting an impact of the cheese-matrix fat content.

**Disclosure of interest**: T. Thorning: None to declare; A. Raben: None to declare; N. Bendsen: None to declare; H. Jørgensen: None to declare; P. Kiilerich: None to declare; Y. Ardö: None to declare; J. Lorenzen: None to declare; K. Kristiansen: None to declare; A. Astrup: Member of the International Carbohydrate Quality Consortium (ICQC) and the advisory boards for McCain Foods Limited, USA, McDonald's, USA and Weight Watchers, USA. Principal investigator of current or recent research projects supported by grants from Arla Foods AMBA, DK, The Danish Dairy Research Foundation, DK, Global Dairy Platform, USA and the Danish Agriculture and Food Foundation, DK.

### Poster presentation no. P119

#### Essential amino acids supplementation and its effects on age related loss of muscle mass and function – a systematic review

##### Marcus Schober^1^* and Elisabet Rothenberg^2^
^1^Sahlgrenska Academy, Gothenburg University, Gothenburg, Sweden; ^2^Food and Meal Science, Kristianstad University, Kristianstad, Sweden *Presenting author

***Background and aims***: Loss of muscle mass, strength and/or function are common in the increasing elderly population, a conceptual and diagnostic term often used for this age related alterations of muscle mass is sarcopenia using dietary supplements, essential amino acids have shown promise to prevent this muscle wasting. The aim of this systematic review was to evaluate currently published data investigating the use of essential amino acids in prevention of age related muscle wasting in individuals with or at risk of sarcopenia over the age of 65.

***Methods***: The electronic databases PubMed and Scopus were searched. Inclusion criteria were experimental studies in English from 1994 to 2015, using essential amino acid supplementation and subjects above 65 years. Search terms conducted were “essential amino acids” and “sarcopenia.” A manual search for studies in found articles was also performed.

***Results***: Eight studies meeting the predetermined inclusion criteria were analyzed. The studies all indicated that intake of essential amino acids could maintain or increase lean body mass and muscle strength/function. +3,7% mean increase (+1,64% placebo) in lean body mass in studies spanning 10 days to 3 months. Strength and functional gains were measured in varying ways but all showed a clear advantage for the intervention group. The best effect was seen in those subjects with sarcopenia.

***Conclusion***: Supplementation with essential amino acids seem to be effective in individuals above age 65 with low muscle mass, strength or function for maintenance or increased lean body mass and muscle strength/function. Optimal dose, intervention period, and adequate combination of amino acids remain to be determined and warrants further research.

**Disclosure of interest**: None to declare.

### Poster presentation no. P120

#### Relationship between micro element levels of public drinking waters and body compositions of people living in Turkey

##### Ihsan Cetin^1^, Mahmut T. Nalbantcilar^2^, Birsen Yilmaz^3^*, Meryem S. Guler^1^, Kezban Tosun^4^ and Selcuk Akin^5^
^1^Department of Nutrition and Dietetics, Batman University Health School, Batman, Turkey; ^2^Faculty of Engineering and Architecture, Department of Geological Engineering, Batman University, Batman, Turkey; ^3^Faculty of Health Sciences, Department of Nutrition and Dietetics, Gazi University, Ankara, Turkey; ^4^Batman State Hospital Diet Outpatient Clinic, Batman, Turkey; ^5^Batman State Hospital Biochemistry Laboratory, Batman, Turkey *Presenting author

***Background and aims***: Microelements content of drinking water may contribute to weight loss by lowering total energy intake and/or altering metabolism. However, there is no extensive research that analyses the potential association between microelements in drinking water and body composition. In this study, we aimed to evaluate the association between some clinically important elements in public drinking water and body compositions in subjects who are average, overweight, and obese.

***Methods***: The population of subjects was divided into three groups (115 female subjects in each group) on the basis of clinical cutoff points of body mass index (BMI) for lean, overweight, and obese subjects. We measured levels of iron (Fe), copper (Cu), cobalt (Co), zinc (Zn), manganese (Mn), molybdenum (Mo), selenium (Se), chromium (Cr), and bromine (Br) in wells of municipal water. To detect element contents, the samples were chemically analyzed with the 2C Full Suite (ACME Analytical Laboratories, Vancouver, Canada) using inductively coupled plasma mass spectrometry. Body composition measurements were performed with the Tanita BC 418 MA analyzer.

***Results***: The values of BMI showed significant positive correlations with Cr in all subject (*p*<0.05).The percentage of obesity showed statistically significant positive correlations with Co and Se in drinking water in all subjects (*p*<0.05, *p*<0.01; respectively). Basal metabolic rate (BMR) showed statistically significant positive correlations with Br (*p*<0.05).

***Conclusion***: The results of the present study show a significant effect of micro element levels in drinking water on weight gain and loss. To protect individuals against obesity and excessive weight gain, dietitians and other experts should encourage patients to check the micro elements content of the drinking water they consume, whether tap or bottled, and choose drinking water most appropriate for their needs and healthy weight loss.

**Disclosure of interest**: None to declare.

### Poster presentation no. P121

#### Fiber-rich pork meat balls as satiety enhancing food products

##### Ursula Kehlet^1^*, Josephine Kofod^2^, Margit Aaslyng^1^ and Anne Raben^2^
^1^DMRI, Danish Technological Institute, Taastrup, Denmark; ^2^Department of Nutrition, Exercise and Sports, University of Copenhagen, Frederiksberg, Denmark *Presenting author

***Background and aims***: In the context of the still rising obesity epidemic, the development of high protein, fiber-rich foods targeting appetite control is beneficial. The aim was to investigate protein-dietary fiber interaction and determine how appetite and energy intake (EI) were affected by 1) fiber addition to meat balls, 2) the protein source (animal vs. vegetable protein), and 3) the food matrix of the dietary fiber (fiber meat balls vs. fiber bread).

***Methods***: In a cross-over study, 36 healthy normal-weight men were served four test meals: meat balls+wheat bread (NoFiber meal); meat balls w/fiber+wheat bread (MeatFiber meal); meat balls+fiber bread (BreadFiber meal); and vegetable meat balls having a natural fiber content+wheat bread (VegFiber meal). Energy density and macronutrient distribution were similar, and the fiber content was 13 g in the three fiber meals. *Ad libitum* EI after 4 h was the primary endpoint. Further, subjective appetite sensations were assessed during the 4 h.

***Results***: *Ad libitum* EI was not significantly different between meals, even though a tendency was seen for a reduced EI after MeatFiber compared with NoFiber (*p*=0.07). MeatFiber also increased satiety and fullness and decreased hunger and prospective food intake compared with NoFiber (meal effect, *p*<0.01). There was a tendency toward an effect of the food matrix, as MeatFiber increased satiety (*p*=0.07) and fullness (*p*=0.08) and decreased hunger (*p*=0.06) compared with BreadFiber. The protein source did not affect the results.

***Conclusion***: Meatballs with fiber were more satiating than meatballs without fibers. The effect seemed to be more pronounced when fiber was added to meatballs compared to when added to bread. No differences were seen between animal and vegetable protein-based meals. In conclusion, meat products with fiber could be used as satiety-enhancing foods targeting consumers who want to maintain or lose body weight.

**Disclosure of interest**: None to declare.

### Poster presentation no. P122

#### Working with nutritional goals: a nutritional 1-day survey at Uppsala University Hospital

##### Sigrid Wegener*, Ingrid Martinsson, Karin Kauppi and Anna-Karin Gunnarsson Department of Orthopedics, University Hospital, Uppsala, Sweden *Presenting author

***Background and aims***: Upon admission to hospital, 20–50% of patients are malnourished. During hospital stay, patients do not receive enough calories. The aim of this 1-day survey was to investigate how many patients were at risk of malnutrition, to what extent the estimated energy requirements was reached, and if there was any documentation on workarounds about nutrition. The survey is done once a year, and 2015 was the 8th time.

***Methods***: Nutritional goals: Energy intake shall reach the level of=75% of estimated energy requirement in=0% of the inpatients. Screening for nutritional status shall be performed in=50% of the inpatients. A care plan should be set up for at least 70% of the patients at risk. The survey is mandatory for all care units. Exclusion criteria are palliative care. Patients that are fasting due to surgery or medical examination were excluded. The results were presented on line within a month from the date of the survey. The staff was offered a web-based introduction to the survey.

***Results***: In total, 418 patients were included in the survey. Out of 208 patients (50%) screened for malnutrition 85 (41%) were at risk. Of patients at risk, 25 (21%) got documented nutritional therapy. One hundred and thirty-three patients (32%) were screened during the first 3 days upon admission. Two hundred and one patients (55%) received 75% of their estimated energy need. One hundred and eleven patients (30%) received 50–75%, and 46 patients (12%) received 25–50% of estimated energy needs. Ten patients (3%) did not obtain 25% of their estimated energy needs.

***Conclusion***: The nutritional goals were not obtained. Measuring alone is not enough to improve routines and outcome. Work on continuous improvement of the nutrition process is now ongoing.

**Disclosure of interest**: None to declare.

### Poster presentation no. P123

#### Weight change and functional outcome in patients after a hip fracture

##### Hanne Rosendahl-Riise^1^, Svanhild Ådnanes^2^, Vilde A. Skodvin^2^, Anette H. Ranhoff^1^ and Jutta Dierkes^2^* ^1^Department of Clinical Science, University of Bergen, Bergen, Norway; ^2^Department of Clinical Medicine, University of Bergen, Bergen, Norway *Presenting author

***Background and aims***: Functional outcomes after a hip fracture are often unsatisfactory, even if the patients have been in relative good age-related health before the fracture. Reasons for reduced functions are due to the fracture itself, but may be also due to dietary and personal factors. However, there is a lack of data concerning the time period immediately following the fracture. Therefore, we investigated the change of weight and anthropometric outcomes and their impact on function during the 3 months following a hip fracture in patients with a first fracture.

***Methods***: Observational follow-up study in patients=65 years with a first hip fracture and who were able to walk independently before the fracture. Anthropometric and dietary assessment in the hospital, during rehabilitation and after the patients returned to their home. Assessment of handgrip strength (HGS) and New Mobility Score (NMS) after the patients returned to their home.

***Results***: In the hospital, 72 patients with a first hip fracture were approached, and in *n*=31, we obtained data from three visits (hospital, 1–2 days after the surgery), rehabilitation (after 17±8 days), at home (after 69±18 days). Average age was 78±10 years, and 23 women and 8 men were included. NMS (range 0–9; 9 indicating best mobility) was on average 5, and HGS was 16±7 kg in women and 31±8 kg in men. Weight change between hospital and at home was –2.0±3.6 kg, and between rehabilitation and at home –1.6±2.6 kg. Main determinants of NMS were age and weight change between hospital and at home, which explained 44% of the variation of NMS. Determinants of HGS were sex and weight change between rehabilitation and at home, which explained 74% of the variation of HGS.

***Conclusion***: Weight loss in older hip fracture patients was associated with reduced mobility and reduced muscle strength. More efforts are needed to maintain weight and muscle strength in these patients.

**Disclosure of interest**: None to declare.

### Poster presentation no. P124

#### Nutritional status in the CKD population treated at Haukeland University Hospital

##### Kristina Sandnes^1^*, Jutta Dierkes^1^, Hans-Peter Marti^1,2^ and Ingegjerd Sekse^1,2^
^1^Department of Clinical Medicine, University of Bergen, Bergen, Norway; ^2^Division of Nephrology, Haukeland University Hospital Bergen, Bergen, Norway *Presenting author

***Background and aims***: Undernutrition is a risk factor for increased health care costs and for mortality in hospitalized patients and prevalence of about 30% has been reported in Norway. There are, however, few data available on the nutritional status of outpatients with chronic diseases. Impaired renal function and its complications are a risk factor for undernutrition, and in addition, nutritional restrictions may limit dietary intake. We wanted to estimate the prevalence of undernutrition in the outpatient population with moderate to severe Chronic Kidney Disease (CKD) treated at Haukeland University Hospital (HUS) and relate this to dietary factors.

***Methods***: Outpatients with an appointment at the department of nephrology at HUS between August and December 2015 were invited. Patients were asked about lifestyle and health conditions, anthropometric measurements were taken, a 24 h dietary recall, NRS2002 and blood and urine samples were obtained. Nutritional status was analyzed taking age, gender, and stage of CKD into account.

***Results***: We included 112 patients with CKD (mean age 63±16 years, 33 female, 79 male), thereof 44 in CKD stage 3 (GFR 59–30 ml/min/1,73 m^2^), 52 in CKD stage 4 (GFR 29–15 ml/min/1,73 m^2^), and 16 in CKD stage 5 (GFR<15 ml/min/1,73 m^2^). Mean BMI was 27.8±5.1 kg/m2, and 32 patients had either type I or II diabetes mellitus. Patients with CKD stage 5 were older and had a lower overall energy and protein intake, but similar NRS2002 scores. Out of 112 patients, three were at nutritional risk, 34 patients were overweight, and 37 were obese.

***Conclusion***: In this outpatient population with moderate to severe CKD, overweight, and obesity rather than undernutrition were more significant nutritional problems. This is surprising as patients with end-stage renal disease are frequently at nutritional risk. However, edema and steroid treatment may affect BMI measurements, making assessment of nutritional risk less reliable. In addition, only 14% of the patients suffered from stage 5 CKD.

**Disclosure of interest**: None to declare.

### Poster presentation no. P125

#### Amino acid and lipid metabolism are differentially affected after diets of herring and pork/chicken

##### Andrew Vincent^1^*, Otto Savolainen^1^, Helen Lindqvist^2^, Mads Lind^3^, Ingrid Undeland^1^, Ann-Sofie Sandberg^1^ and Alastair Ross^1^
^1^Food and Nutrition Science, Chalmers University of Technology, Gothenburg, Sweden; ^2^Clinical Nutrition, Gothenburg University, Gothenburg, Sweden; ^3^Department of Nutrition, Exercise and Sport, University of Copenhagen, Copenhagen, Denmark *Presenting author

***Background and aims***: Replacing red meat with fish is associated with improved health outcomes. We aimed to discover what mechanisms may underlie this association.

***Methods***: Randomized crossover trial including 11 healthy obese men and women (age 24–70 years). Subjects were randomly assigned to 4 weeks of herring diet (150 g baked herring fillets/day, 5 days/week) or reference diet (pork and chicken fillets) and switched diets after a 2 week washout. Plasma metabolites were analyzed at 0, 2, and 4 weeks using gas chromatography-tandem mass spectrometry. Data were analyzed using mixed models adjusted for subject, age, and gender.

***Results***: Nineteen metabolites were found to be significantly different between the two diets. The TCA intermediates citric acid, fumaric acid, and isocitric acid were significantly higher after the meat diet compared to the herring diet, possibly indicating greater flux from lipid metabolism through to the TCA cycle. The glyoxylic acid pathway intermediates glycolic acid and oxalic acid were increased, as was the meat-consumption biomarker methyl histidine. From the herring diet the plasma levels of amino acids asparagine, ornithine, and glutamine were observed to be increased compared to the mixed diet of chicken and pork. Of additional interest was the increase in glucosamine from the herring diet and agmatine from the mixed diet.

***Conclusion***: The results suggest that herring compared to a mixed chicken and pork-based diet has a differential effect on both amino acid and lipid metabolism, especially around the conditionally essential amino acid arginine. In particular, the finding that glucosamine was elevated after regular consumption of herring is intriguing, given its touted role in joint health and protection against arthritis. Our findings support the idea that there are metabolic effects of herring that are not related to its n-3 fatty acid content.

**Disclosure of interest**: None to declare.

### Poster presentation no. P126

#### Lean seafood intake reduces cardiovascular lipid risk factors in healthy subjects – results from a randomized controlled trial with crossover design

##### Bjørn Liaset^1^*, Eli K. Aadland^2^, Mette Schmedes^3^, Ingvild E. Graff^1^, Charles Lavigne^1^, Øyvin Eng^4^, Martine Paquette^5^, Ulrik K. Sundekilde^3^, Lise Madsen^1^, Jette F. Young^6^, Hanne C. Bertram^3^, Asle Holthe^2^, Gunnar Mellgren^4^, Morten R. Clausen^3^ and Helene Jacques^5^
^1^National Institute of Nutrition and Seafood Research, Bergen, Norway; ^2^Bergen University College, Bergen, Norway; ^3^Department of Food Science, Aarhus University, Aarslev, Denmark; ^4^Hormone Laboratory, Haukeland University Hospital, Bergen, Norway; ^5^School of Nutrition, Laval University, Quebec, Canada; ^6^Department of Food Science, Aarhus University, Aarhus, Denmark *Presenting author

***Background and aims***: Randomized controlled studies linking dietary major protein sources to metabolic health are still limited. Therefore, the current study was undertaken to elucidate the potential of two major animal protein sources, lean seafood and non-seafood (meat, egg, and milk), to modulate the risk factors for metabolic disease in healthy subjects.

***Methods***: This study was a randomized, controlled trial with a crossover design. After 3 week run-in periods, and separated by a 5 week wash-out period, 20 healthy subjects (7 men and 13 women) consumed two balanced diets for 4 week that varied in the main protein sources; 60% (11.4 energy%) of total dietary proteins from lean-seafood or non-seafood sources. At day 1 and 28 of each intervention, morning spot urine samples were taken, and fasting and postprandial blood samples were collected before and after, respectively, consumption of test meals with cod or lean beef.

***Results***: The diets did not alter serum insulin and glucose concentrations. However, relative to the non-seafood diet period, the lean-seafood diet period reduced postprandial serum lactate (*P*=0.012), serum triacylglycerol (TAG) (*P*=0.01), and medium-sized VLDL particles (*P*=0.02) concentrations. The lean seafood intervention also prevented the postprandial serum elevation of total- to high-density lipoprotein (HDL) cholesterol ratio (*P*=0.01) relative to the non-seafood intervention. The lean-seafood diet period reduced the urinary levels of L-carnitine (*P*<0.01), 2,6-dimethylheptanoylcarnitine (*P*<0.03), and *N*-2-pyridone-5-carboxamide (2PY) (*P*<0.01), indicating changes in lipid and energy metabolism.

***Conclusion***: The dietary protein source determines postprandial metabolism in healthy subjects in a manner that may have impact on long-term development of insulin-resistance, type 2 diabetes, and cardiovascular disease.

**Disclosure of interest**: None to declare.

### Poster presentation no. P127

#### Gender differences in practice, knowledge, and attitudes regarding food habits and meal patterns among community dwelling older adults

##### Julie E. Johannesson^1^*, Elisabet Rothenberg^2^, Synneve D. Ivanoff^3^ and Frode Slinde^1^
^1^Department of Clinical Nutrition, Sahlgrenska Academy, Gothenburg, Sweden; ^2^School of Education and Environment, Kristianstad, Sweden; ^3^Department of Neuroscience and Physiology, Sahlgrenska Academy, Gothenburg, Sweden *Presenting author

***Background and aims***: To study the gender differences in older adults according to practice, knowledge, and attitudes regarding food habits and meal patterns.

***Methods***: A cross-sectional study in two urban districts of Gothenburg, Sweden. Telephone interviews regarding food habits and meal patterns were conducted. A total of 297 individuals were included, 102 men and 195 women. They were 80 years or older and living in ordinary housing without being dependent upon the municipal home help services or help from another person in Activities of Daily Life, and cognitively intact, defined as having a score of 25 or higher in the Mini Mental State Examination.

***Results***: Almost all participants (99%) ate their main meal at home, and men preferred company at meals more often (*p*<0.001). Women had the sole responsibility to shop for food more often (*p*<0.000), and generally regarded cooking as a routine or something they just had to do. Among men, few (13%) took a great interest in cooking, and 36% of the men stated that cooking was something they were not capable of performing (*p*<0.000). Men had company at meals every day more often (71% vs. 40%). Respondents stated that loneliness took away the enjoyment of cooking and changed their habits when becoming a widow or widower.

***Conclusion***: Women take greater responsibility for the household than men, regardless of marital status. A large proportion of the men thought cooking was something they were not able to do. The findings in this study may indicate a possible gender difference in the need for societal support.

**Disclosure of interest**: None to declare.

### Poster presentation no. P128

#### Prealbumin is a marker of nutritional risk in hyperemesis gravidarum

##### Olga Zybkina^1^ , Jutta Dierkes^1^*, Anne L. B. Monsen^2^ and Jone Trovik^3^
^1^Department of Clinical Medicine, University of Bergen, Bergen, Norway; ^2^Laboratory for Clinical Biochemistry, Haukeland University Hospital, Bergen, Norway; ^3^Department of Clinical Science, University of Bergen, Bergen, Norway *Presenting author

***Background and aims***: Hyperemesis gravidarum (HG) affects 1% of pregnancies and is potentially harmful for mother and fetus. Prealbumin is a marker of nutritional status. We wanted to investigate if prealbumin level was associated with severity and nutritional risks of NVP (nausea and vomiting in pregnancy).

***Methods***: A case-control study including 76 hospitalized patients with HG and 36 healthy controls. Serum prealbumin was correlated to clinical and biochemical nutritional parameters, including 24 h dietary recall.

***Results***: HG patients had longer gestational length than controls (median 8.6 vs. 7.0 weeks, *p=*0.001). Otherwise, the groups were similar regarding pre-pregnant BMI, parity, proportion of HG in earlier pregnancies and weight at inclusion. The prealbumin levels were significant lower in HG versus controls: median 0.19 mg/dL versus 0.23 (95% CI 0.18–0.20 and 0.19–0.24). Compared to the control group, HG patients had significantly lower 24 h energy intake (median 720 kcal vs. 1,646, *p*=0.017), larger weight-change at inclusion (median –3 kg vs. +1 kg, *p*<0.001), higher percentage of ketonuria +3–4 (69% vs. 3% *p<*0.001), and higher PUQE-score (Pregnancy Unique Questionnaire of Emesis and nausea) median 13, 95% CI 13–14 vs. 7, 95% CI 6–9). Prealbumin level, 24 h energy, and protein intake significantly decreased while weight-loss and ketonuria increased across severity of NVP as classified by the three tiered PUQE-score <6, 7–12, and 13–15 (all *p*= 0.013). Prealbumin level was significantly correlated to 24 h protein intake, Pearson *r*=0.339 (*p*=0.008).

***Conclusion***: Prealbumin seems to be a reliable parameter for validating patients with severe NVP/HG who are at high nutritional risk.

**Disclosure of interest**: None to declare.

### Poster presentation no. P201

#### Physico-chemical and sensory properties of yogurt processed from cow's milk and soymilk alone and in combination

##### Tahar Amrouche* on behalf of *Food quality Group*
Department of Agronomy, M. Mammeri University, Tizi Wezzu, Algeria *Presenting author

***Background and aims***: Fermented soymilk, unlike fermented milk, contains no lactose or cholesterol. In addition, soy protein was found to have a greater antioxidative ability in preventing lipid oxidation, compared to casein. This study aimed to assess physico chemical and sensory proprieties of yogurt processed by inoculation of cow's milk and soymilk used alone and in combination with lactic acid bacteria.

***Methods***: Yogurt was processed by inoculation of cow's milk and soymilk used alone and in combination (ratio 1:1 v/v) with freeze-dried culture of *Lactobacillus bulgaricus* and *Streptococcus thermophilus*. Three samples from each processed yogurt were evaluated for protein and fat content, total dry extract, pH, and Dornic acidity. Sensory properties of yogurt produced were assessed by a panel using a commercial yogurt as control.

***Results***: The pH values of yogurt samples ranged from 4.65±0.03 in cow's milk yogurt, 4.60±0.05 in soymilk yogurt, and 4.64±0.01 in the mix. Soymilk yogurt was low in Dornic acidity (81.66±1°D). Fat and total dry extract were highest in cow's milk yogurt: 31±1 g/l and 106.6±0.2 g/l, respectively. There was significant difference (*P*<0.05) in the protein content between cow's milk yogurt (3.1±0.2 g/l) and soymilk yogurt (3.47±0.01 g/l). Soymilk yogurt and cow's milk yogurt and their mix compared favorably well with commercial yogurt in overall acceptability.

***Conclusion***: Soymilk yogurt alone or in combination with cow's milk yogurt can be adopted as substitute to cow's milk yogurt especially by the low-income earners due to its cheaper raw materials, and as protein supplement at household level. It can also be processed with simple processing technology.

**Disclosure of interest**: None to declare.

### Poster presentation no. P202

#### Relationship between acrylamide found in foods and health

##### Birsen Yilmaz* and Nevin Sanlier Department of Nutrition and Dietetics, Faculty of Health Sciences, Gazi University, Ankara, Turkey *Presenting author

***Background and aims***: Acrylamide known names such as 2-propenamide is a monomer in the form of white crystal used polyacrylamide synthesis. After foods with high carbohydrate is applied heat treatment (above 120°C), acrylamide has been occurred. This review was planned to evaluate acrylamide.

***Methods***: Many articles have been scanned from the databases related to the topic.

***Results***: It is reported that asparagine or oils may induce the acrylamide formation. It has damaged to the nervous system, and intake of high amounts may cause adverse effects on hormonal glands and muscles. The presence of acrylamide was detected in prepared or semi-prepared foods such as potato chips (170–2,287 μg/kg), fries (<50–3,500 μg/kg), biscuits/cookies (150–400 μg/kg), and breakfast cereals (<30 to 1,356 μg/kg). Contrarily, the presence of acrylamide in foods prepared by the boiling method (for example boiled potatoes), it may not be detected. While some studies have usually focused on the processing temperature and time of potatoes, some studies have centered on reducing the formation of acrylamide via addition of some matters such as flavones (tricin, apigenin, and luteolin), isoflavones (daidzein, genistein, and daidzein), and reducing sugar. The European Union has determined amount of acrylamide for drinking water (0.1 μg/L) and foods (0.5–0.8 μg/kg/day) as the upper limit.

***Conclusion***: According to the studies, it is recommended that some methods need to be considered to reduce the content of acrylamide in foods. These methods are as follows; avoiding making deep fat frying and paying attention to time-temperature relationship, adding the enzyme asparaginase, storing potatoes <8°C and soaking after peeling, adding citric/ascorbic acid, using yeast fermentation instead of chemical leavening agent, to prevent the Maillard reaction adding some matters such as sulfite/flavon, and using sodium hydrogen carbonate instead of ammonium hydrogen carbonate. Further studies are needed to better understand the mechanisms of acrylamide.

**Disclosure of interest**: None to declare.

### Poster presentation no. P203

#### Assigning fatty acid composition to foods in a comprehensive nutrient calculation system (KBS)

##### Monica H. Carlsen*, Nina Norberg and Anette Hjartåker Department of Nutrition, University of Oslo, Oslo, Norway *Presenting author

***Background and aims***: Recent scientific evidence suggests that the fatty acid composition may be more important in a healthy diet than total fat intake. The content of fat and the fatty acid composition of food items in the Norwegian diet have changed significantly the last 50 years. Department of Nutrition, UiO has developed a set of scientific tools for assessing dietary intake. A vital part of this toolset is the KBS (Kostberegningssystem), a nutrient calculation and food composition database system. Unfortunately, most of the food items in KBS have been lacking fatty acid composition data or had outdated values. The aim of this project was to compile fatty acid values for the 3,100 different food items in the latest version of the KBS food composition database.

***Methods***: Data on fatty acid composition were compiled according to guidelines by FAO. Sources of data included: 1) Annual analytical projects conducted in collaboration with the Norwegian Food Safety Authority; 2) Analytical reports from other Norwegian institutions; 3) Other food composition databases; 4) Scientific articles; and 5) Recipes. Data were checked for suitability according to: 1) Unequivocal identification of food sample; 2) Collection of representative sample; 3) Preparation of food sample; and 4) Number of samples. All procedures used in the project were documented, and all references were archived.

***Results***: Values for 29 individual fatty acids were compiled for 2,830 food items; values for 750 food items were compiled from analytical reports and projects, 550 from other national food composition databases, 1,500 were calculated with recipes, and 30 were compiled from scientific articles. Two hundred and seventy food items did not contain fat.

***Conclusion***: This addition to the food composition database will enable future studies to look at the individual and total effect of fatty acids on health.

**Disclosure of interest**: None to declare.

### Poster presentation no. P204

#### Objective validation and comparison of a web-based food record tool and a food frequency questionnaire using the doubly labeled water technique in a middle-aged population

##### Sanna Nybacka^1^*, Heléne B. Forslund^1^, Elisabet Wirfält^2^, Ingrid Larsson^3^, Ulrika Ericson^2^, Eva W. Lemming^4^, Göran Bergström^5^, Bo Hedblad^2^, Anna Winkvist^1^ and Anna-Karin Lindroos^4^
^1^Department of Internal Medicine and Clinical Nutrition, Institute of Medicine, University of Gothenburg, Gothenburg, Sweden; ^2^Department of Clinical Sciences in Malmö, Lund University, Malmö, Sweden; ^3^Department of Endocrinology, Diabetology and Metabolism, Sahlgrenska University Hospital, Gothenburg, Sweden; ^4^National Food Agency, Uppsala, Sweden; ^5^Sahlgrenska Centre for Cardiovascular and Metabolic Research, Sahlgrenska University Hospital, Gothenburg, Sweden *Presenting author

***Background and aims***: Two web-based dietary assessment tools have been developed for use in large-scale studies in Sweden; the Riksmaten method (a 4-day food record) and the MiniMeal-Q (a food frequency method). The aim was to validate these methods against objectively measured total energy expenditure (TEE) with doubly labeled water (TEE_DLW_), and to compare reported energy and macronutrient intake.

***Methods***: This study was conducted within the Pilot study of Swedish CArdioPulmonary bioImage Study (SCAPIS), which included 1,111 randomly selected men and women aged 50–64 years from the Gothenburg general population. Of these, 200 were enrolled in the SCAPIS Diet sub-study. TEE_DLW_ was measured in a sub-sample (*n=*40).

***Results***: Compared with TEE_DLW_, both methods underestimated energy intake; –2.5±2.9 MJ by the Riksmaten method and –2.3±3.6 MJ by MiniMeal-Q. Mean reporting accuracy was 80 and 82%, respectively. The correlation between reported energy intake and TEE_DLW_ was *r=*0.4 for the Riksmaten method (*P<*0.05) and *r=*0.28 (non-significant) for MiniMeal-Q. Women reported similar average intake of energy and macronutrients in both methods, whereas men reported higher intakes with the Riksmaten method. Energy adjusted correlations ranged from 0.14 (polyunsaturad fat) to 0.77 (alcohol). Bland-Altman plots showed acceptable agreement for energy, protein, and carbohydrate intake, whereas the agreement for fat intake was poorer.

***Conclusion***: Both dietary assessment methods are applicable for middle-aged women and men. According to energy intake data, both methods displayed similar precision on energy intake at group level. However, MiniMeal-Q was less successful in ranking individuals than the Riksmaten method. This limitation may also influence ranking on macronutrients.

**Disclosure of interest**: None to declare.

### Poster presentation no. P206

#### Appetizing muffins designed for nutritional needs of older adults

##### Evelina Tibäck^1^*, Karin Wendin^1^, Elisabet Rothenberg^2^ and Berit Albinsson^1^
^1^Food and Bioscience, SP Technical Research Institute of Sweden, Gothenburg, Sweden; ^2^Food and Meal Sciences, Kristianstad University, Kristianstad, Sweden *Presenting author

***Background and aims***: Thanks to good living conditions, the population of older adults is growing. By increasing age, the prevalence of disease increases and there by the risk of disease related malnutrition (DRM). Reasons for DRM are complex including loss of appetite, mastication problems, social changes, and more. The condition entails prolonged hospital stays and higher health care costs, since DRM elevates the risk of weakened immune system, muscle loss, depression, etc. In Sweden, more than 1/3 of adults admitted to hospitals and 1/2 of residents in nursing homes have (or are at risk of) DRM. In order to counteract DRM, appetizing and nutritious food products are needed.

One way of enabling adequate nutritional intake for those with poor appetite is to offer energy/protein-rich snacks between meals. In Sweden, coffee time is normally a much appreciated part of the day, and muffins are a popular choice among older adults. Designing muffins to suit older adults’ nutritional needs could contribute to decreased DRM. The aim of this study was to investigate added nutritional value along with the sensorial effects of increased fat/protein content in muffins.

***Methods***: Recipes and processing scheme for four different muffins were developed. Nutritional calculations and evaluation of sensory properties were performed. Further moisture content, water activity, weight loss, and size were measured.

***Results***: The fat and protein addition affected the nutritional value and sensory properties of the muffins:

***Conclusion***: Designing muffins for older adults’ nutritional needs is promising and with further recipe/process development appetizing sensory properties can be achieved.

**Table:** 

	Content (g/100 g muffin)		
		
Muffin type	Fat	Protein	Sensory properties
Reference	27	4,9	soft, smooth
+ fat	42	3,8	hard, compact, pointy, low flavors
+ protein (whey)	23	12,4	flat, moist, fatty mouth feel
+ fat and protein	37	10,2	a bit hard, compact, atty mouth feel effects of protein are dominant

**Disclosure of interest**: None to declare.

### Poster presentation no. P207

#### Microstructure of whole grain rye products and its impact on in vitro digestion and responses in humans

##### Daniel Johansson^1^*, Rikard Landberg^1,2^, Marie Alminger^3^ and Maud Langton^1^
^1^Department of Food Science, Swedish University of Agricultural Sciences, Uppsala, Sweden; ^2^Nutritional Epidemiology Unit, Institute of Environmental Medicine, Karolinska Institutet, Stockholm, Sweden; ^3^Division of Food and Nutrition Science, Department of Biology and Biological Engineering, Chalmers University of Technology, Gothenburg, Sweden *Presenting author

***Background and aims***: Over nutrition is a strong contributor to obesity and its related disorders and therefore an important target in improving public health. One way to address this is development of tailored foods, which contribute to beneficial responses, such as reduced feelings of hunger or reduced insulin response. Process-induced changes in microstructure and composition may influence absorption kinetics of nutrients, and consequently postprandial responses. The aim was, therefore, to elucidate how food microstructure affects to postprandial responses. This may aid in the development of healthy foods.

***Methods***: Digestion of a range of rye products, including crisp breads, sourdough bread, and breakfast cereals, as well as soft and crisp white wheat breads were studied using static and dynamic *in vitro* methods. Disintegration and viscosity during gastric digestion, as well as microstructural changes and release of maltose during small intestinal digestion were assessed. The crisp breads were also used in intervention trials, and perceived satiety, glucose, and hormone responses were measured.

***Results***: Microstructure influenced the digestion of the products. The presence of a protein network in the wheat breads resulted in a higher degree of structural disintegration during gastric digestion than for the rye products. Further, the rate of disintegration was slower for products which retained a cohesive starch network after mastication. Slower disintegration rate and intact fibers also appeared to lead to delayed maltose release. For the crisp breads, this also correlated with lower insulin response in humans. However, despite rapid disintegration and degraded fibers the release of maltose was low for sourdough rye bread, possibly due to an amylose layer surrounding the starch granules.

***Conclusion***: In conclusion, microstructural features influence the digestive process with implications for postprandial responses in humans.

**Disclosure of interest**: None to declare.

### Poster presentation no. P209

#### Omega 3 activates PPAR response genes in a high fat diet, but not in a low fat diet, in male Wistar rats. In comparison to omega 6 and specific PPAR-agonists

##### Mari L. Grinna*, Elin Strand, Carine Lindquist, Ottar Nygård, Rolf K. Berge and Bodil Bjørndal Department of Clinical Science, University of Bergen, Bergen, Norway *Presenting author

***Background and aims***: A Western diet combined with a sedentary lifestyle is causative to the current epidemic of overweight and obesity, and their comorbidities. A greater knowledge on how diet and lifestyle affect the human metabolism is essential to cope with this epidemic. Peroxisome proliferator activated receptors (PPAR) are important regulators of overall energy metabolism. These nuclear receptors are affected by both synthetic as well as natural ligands including dietary fatty acids (FAs), making PPAR an executive signaling pathway between dietary intake, gene expression, and energy metabolism. The aim of the study is to compare the regulatory role of dietary omega 3 (n-3) and n-6 FA to the effects exerted by specific PPAR-agonists on energy metabolism.

***Methods***: In the first animal study (*n*=20), male Wistar rats received either placebo (*n=*8), the PPARα-agonist WY-14,643 (*n*=6) or the PPARγ-agonist Rosiglitazone (*n*=6), for 12 days. In the second animal study (*n*=48), male Wistar rats received either a high fat (HF) (*n*=24) or a low fat (LF) (*n*=24) diet supplemented with n-3 (*n*=8), or n-6 (*n*=8) FAs respectively during 4 weeks.

Gene expression in liver and epididymal adipose tissue as well as lipids in plasma and liver was analyzed.

***Results***: We detected that a HF diet compared to a LF diet have impact on the n-3 effect on PPAR target genes. This was not detected for the n-6 diets. N-3 demonstrated a similar pattern as for the PPARa-agonist, in both HF and LF diet, on plasma lipids. Furthermore, the n-6 groups showed a tendency to the same effect on plasma lipids as the PPARγ-agonist.

***Conclusion***: A HF diet seems to be essential for the n-3 effect on PPAR responsive genes. Furthermore, both HF and LF diets supplemented with n-3 demonstrated beneficial effects on plasma lipids.

**Disclosure of interest**: None to declare.

### Poster presentation no. P210

#### Eicosapentaenoic and docosahexaenoic acid – enriched high fat diet ameliorates sarcopenia-related changes in gastrocnemicus skeletal muscle transcriptome of C57BL/6J mice

##### Nikulkumar Soni*, Alastair B. Ross, Nathalie Scheers, Intawat Nookeaw, Britt G. Gabrielsson and Ann-Sofie Sandberg Department of Biology and Biological engineering, Chalmers University of Technology, Gothenburg, Sweden *Presenting author

***Background and aims***: Dietary supplementation with marine fatty acids, such as eicosapentaenoic acid (EPA) and docosahexaenoic acid (DHA) has potential protective effects against accelerated aging or obesity-induced sarcopenia. However, the underlying mechanisms of such effects are still poorly understood. Therefore, we aimed to identify the underlying mechanism by which EPA/DHA delays the onset of sarcopenia in mouse models.

***Methods***: Microarray and bioinformatic analyses were used to detect differentially expressed genes in the gastrocnemicus skeletal muscle (gSkM). Western Blot analyses of selected genes were performed to support the findings and for further mechanistic analyses.

***Results***: Transcriptome data revealed an increase in slow-fiber-specific gene expression in the HFD-EPA/DHA compared to the HFD-corn oil fed mice, which was confirmed by Western blot that showed increased protein expression of Tnnc1, a slow-fiber-type marker. The HFD-EPA/DHA mice also had increased nuclear localization of the Nfatc4 protein, which controls fiber-type composition. The pathway analyses identified increased expression of genes associated with muscle contraction, and increased mitochondrial oxidative phosphorylation and -oxidation in the mice fed HFD-EPA/DHA. Protein analyses of Acc, which inhibits mitochondrial fatty acid -oxidation, were lower in the HFD-EPA/DHA mice. Increased gene expression of *Ppargc1a* in the HFD-EPA/DHA mice could indicate increased mitochondrial biogenesis.

***Conclusion***: These data show that dietary EPA and DHA promoted a slow-fiber-type gene expression that was associated with increased oxidative phosphorylation and -oxidation in gSkM. We suggest that increased consumption of EPA/DHA could prevent muscle deterioration commonly associated with aging or obesity-induced sarcopenia.

**Disclosure of interest**: None to declare.

### Poster presentation no. P211

#### Short-term activation of PPARs, which are important facilitators in fatty acid metabolism, induce changes in hepatic and cardiac fatty acid composition in rats

##### Mari L. Grinna*, Bodil Bjørndal, Pavol Bohov, Rolf K. Berge, Ottar Nygård and Elin Strand Department of Clinical Science, University of Bergen, Bergen, Norway *Presenting author

***Background and aims***: Dietary fatty acids affect certain metabolic pathways, including pathways controlled by the peroxisome proliferator-activated receptors (PPARs). With the increasing focus on intake of diets with different types of fat, including polyunsaturated fatty acids, it was of interest to explore the underlying mechanisms on pathways affected by dietary fatty acids. Thus, the aim was to induce a proposed response on fatty acid composition in hepatic and cardiac tissues through treatment with specific PPAR agonists.

***Methods***: Male Wistar rats were randomized into three groups: a control group receiving placebo (*n*=8); a PPARα agonist group receiving WY-14,643 (*n*=6); and a PPARγ agonist group receiving rosiglitazone (*n*=6) for 12 days. All animals received a low-fat chow diet and were given a daily dose of placebo or agonist orally. Lipids were measured in plasma, liver, and heart, while fatty acid methyl esters were analyzed in liver and heart by gas-liquid chromatography.

***Results***: Treatment with the PPARa agonist was associated with lower plasma total cholesterol, HDL cholesterol, phospholipids (*P<*0.001), and higher hepatic phospholipids (*P=*0.001). Furthermore, hepatic SFA (*P=*0.03) and MUFA (*P<*0.001) were higher, while n-6 (*P=*0.03) and n-3 (*P<*0.001) PUFAs were lower, compared to control (wt %). Treatment with the PPARγ agonist led to lower plasma triglycerides ( agonist led to lower plasma triglycerides (*P*=0.001) and phospholipids (*P=*0.01), lower cardiac total fat (*P=*0.01) and SFA (*P=*0.002), and higher cardiac n-6 PUFA (*P=*0.003), compared to control. Gene expression patterns were supportive of PPARa-specific effects in liver.

***Conclusion***: Short-term treatment with PPAR agonists induced changes in circulating lipids and fatty acid composition in liver and heart. In future studies, we aim to reveal whether similar patterns can be found through diet-induced activation of specific pathways.

**Disclosure of interest**: None to declare.

### Poster presentation no. P301

#### Food and meals at residential care homes for unaccompanied children in Sweden – a pilot study

##### Cecilia Olsson*, Sandra Mellberg and Maria Waling Department of Food and Nutrition, Umeå University, Umeå, Sweden *Presenting author

***Background and aims***: The number of unaccompanied children has increased radically in Sweden, and a majority of these children are placed in residential care homes (HVB-homes). In a report from The Health and Social Care Inspectorate, it was stated that complaints about food were the most common topic brought up in conversations with the children. The objective of this pilot study was to test a questionnaire with the aim to explore the management of food and meals at HVB-homes for unaccompanied children.

***Methods***: A web-based questionnaire was sent to directors at 113 randomly chosen HVB-homes in Sweden. Fifty-five directors filled out the questionnaire (49 municipality and 6 private HVB-homes).

***Results***: A majority of the HVB-homes breakfast (*n*=52) and in-between meals (*n*=46) were prepared at the HVB-home. The same was seen for lunch (*n*=36) and dinner (*n*=39), but the second most common alternative was that food came from an external company (*n*=6 for lunch and *n*=12 for dinner). When food was prepared and cooked at the HVB-home, the staff working at the home was responsible for planning (*n*=41) and cooking the food (=41). It was also common that children were involved in planning (*n*=34) and cooking of the food (*n*=30). Thirty-one of the directors thought that it was important to involve children in the meal situation at the HVB-home, i.e. 8–10 on a scale from 1 to 10 where 1 was “to a little extent” and 10 “to a large extent.” About one quarter of the directors (*n*=15) indicated that food related situations worked not well at all or fairly well, i.e. 1–6 on a scale from 1 to 10 where 1 was “not well at all” and 10 “very well.”

***Conclusion***: Our results indicate that there are HVB-homes where food related situations do not work well. Further research is needed to identify if these results are representative in a larger sample, and to explore the full potential of food and meals in the establishment of unaccompanied children.

**Disclosure of interest**: None to declare.

### Poster presentation no. P302

#### Dietary surveys are Important for benefit and risk assessment

##### Inger T. L. Lillegaard* Norwegian Scientific Committee for Food Safety, Oslo, Norway *Presenting author

***Background and aims***: Food contains a mixture of nutrients, non-nutrients, additives, and environmental contaminants and can therefore not be categorized into healthy or unhealthy. Benefit-risk assessments take account of both pros and cons of foods and are increasingly in demand.

***Methods***: Risk assessment comprises four steps: hazard identification and characterization, exposure assessment, and risk characterization. The exposure assessment is performed using knowledge of how much consumers eat of specific foods combined with the amount of the substance in the specific foods. The quality of the exposure assessment is dependent on the dietary assessment, and relies on available intake data for the foods containing the substance(s) and for intake data covering the whole population, also the high consumers.

***Results***: Fish is a good example. Benefits of fish intake are well documented; however, fish, like all other food, also contains environmental contaminants like mercury and dioxins. The content of contaminants differs between fish species, and different organs of the fish. Cod filet is a main source for mercury, while for dioxins cod liver is the most contaminated organ. Filet is frequently eaten by many Norwegians, and will for the consumers be the main source of exposure. Cod liver is eaten by few but gives a large exposure for those eating it several times per year. Asking questions of both fish-filet and -liver will give the best basis for the benefit-risk assessment.

***Conclusion***: It is important to design and perform dietary surveys in such a way that the data can be used for both health benefit and risk assessments.

**Disclosure of interest**: None to declare.

### Poster presentation no. P303

#### Validation of very long-chain n-3 polyunsaturated fatty acid intake from a food frequency questionnaire using plasma phospholipid fatty acids

##### Jessica Magnusson^1,^*, Sandra Ekström^1^, Inger Kull^1,2,3^, Sara Nilsson^4^, Ulf Risérus^5^, Niclas Håkansson^1^, Erik Melén^1,3^ and Anna Bergström^1^
^1^Institute of Environmental Medicine, Stockholm, Sweden; ^2^Department of Clinical Science and Education, Karolinska Institutet, Stockholm, Sweden; ^3^Sachs′ Children′s Hospital, South General Hospital, Stockholm, Sweden; ^4^Center for Occupational and Environmental Medicine, Stockholm County Council, Stockholm, Sweden; ^5^Department of Public Health and Caring Sciences, Clinical Nutrition and Metabolism, Uppsala University, Uppsala, Sweden *Presenting author

***Background and aims***: Food frequency questionnaires (FFQs) are commonly used to measure childhood diet in epidemiological studies. Yet, these FFQs are rarely validated in children. We aimed to validate very long-chain n-3 polyunsaturated fatty acid (VLCn-3 PUFA) intake from a FFQ against plasma phospholipid proportions among Swedish school-aged children.

***Methods***: At age 8 years, 2,456 children from the BAMSE birth cohort completed a FFQ and left a blood sample. The FFQ was nutrient calculated and energy adjusted using the residual method. In a subset of 940 children, proportions of fatty acids were analyzed in plasma phospholipids with gas chromatography. Proportions of the fatty acids eicosapentaeonic acid (EPA, 20:5), docosapentaeonic acid (DPA, 22:5), docosahexaeonic acid (DHA, 22:6), as well as the sum of these fatty acids (VLCn-3 PUFA), in % of total fatty acids, were compared between the FFQ and plasma phospholipids using the Spearman rank correlation test. Analyses were stratified according to fatty fish intake, gender, and overweight status.

***Results***: Correlation coefficients were all significant, except for DPA (*r*=0.04, *p*=0.28). The highest correlation was observed for DHA (*r*=0.29, *p*<0.001). Stratified analyses revealed a higher correlation for EPA and a lower correlation for DHA for regular (=1/week) compared to irregular (<1/week) fatty fish intake (EPA: *r*=0.28 vs. *r*=0.15; DHA: *r*=0.13 vs. *r*=0.25). Overall, correlations were somewhat higher for boys than girls and for overweight compared to normal weight children.

***Conclusion***: Among children, there is acceptable validity of VLCn-3 PUFA intake estimated from a FFQ. Overall, correlations were higher for boys and overweight children, and depended on frequency of fatty fish intake.

**Disclosure of interest**: None to declare.

### Poster presentation no. P304

#### Postprandial blood sugar and satiety after two identical meals differing only in degrees of processing – the Shake Study

##### Bára H. Thorsteinssdottir, Masa Hribar, Thórhallur Halldórsson, Alfons Ramel, Laufey Steingrímsdóttir and Bryndís E. Birgisdóttir* Unit for Nutrition Research, Faculty of Food Science and Nutrition, University of Iceland and Landspitali University Hospital, Reykjavík, Iceland *Presenting author

***Background and aims***: High postprandial glycemia has been associated with increased risk of metabolic impairments. The aim was to investigate postprandial blood sugar and satiety after consumption of two identical meals differing only in their degree of processing.

***Methods***: A randomized crossover study including 11 healthy adults between 40 and 70 years of age with BMI 29.0±2.0 kg/m^2^ (mean±SD). The participants came fasting to the unit on two occasions. In one visit, they consumed a solid meal consisting of yogurt, fruits, muesli, and water, and in the other visit, they consumed the same meal homogenized to a liquid within the same time period, in a random order. Postprandial blood glucose was measured at regular intervals as well as hunger and desire to eat on a visual analogue (VAS) scale. Four hours after the meal was consumed, ad libitum intake was measured.

***Results***: The meal gave 550 kcal, 14 g protein, 18 g fat, 81 g carbohydrates, and 11g fiber. Using one-way t-test, the mean blood glucose response was slightly higher (0.2–0.3 mmol/L) after the consumption of the liquid meal in comparison to the solid meal at 30, 60, 90, and 150 min (*p*=0.026). Although the inter-individual difference was large, only one out of 11 had modestly lower glucose response to the liquid meal. No significant difference was found for hunger and desire to eat between the two meals (*p*>0.05), and no difference was found in energy intake at an ad libitum meal eaten four hours after the liquid or solid meal.

***Conclusion***: A liquid breakfast may give a higher postprandial glucose response compared to a solid breakfast with identical composition consumed in the same time period. However, no differences were seen in satiety or hunger after the two meals or later ad libitum intake. Processing of food should be studied further with respect to different metabolic reactions in the body after a meal.

**Disclosure of interest**: None to declare.

### Poster presentation no. P305

#### 3-months intervention with folate-rich legume foods improves folate status but affects 1-carbon metabolism differently in healthy females

##### Cornelia M. Witthöft^1,^*, Mohammed Hefni^2^ and Ali Moazzami^3^
^1^Department of Chemistry and Biomedical Sciences, Linnaeus University, Kalmar, Sweden; ^2^Food Industries Department, Mansoura University, Mansoura, Egypt; ^3^Department of Chemistry & Biotechnology, Swedish University of Agricultural Sciences, Uppsala, Sweden *Presenting author

***Background and aims***: After discontinuation of a folic acid flour fortification program in Egypt, alternative strategies to increase folate intake are needed. Bioprocessing, e.g. germination of legumes, enables production of foods with increased folate content. Aim was to determine the effects of folate-rich foods versus supplemental folic acid on folate status and – using NMR metabolomics approach – on one-carbon metabolism.

***Methods***: Fifty-seven women were assigned to parallel interventions: folic acid group (*n*=18) receiving 500 g/d supplemental folic acid, food group (*n*=19) receiving 250 g/d folate from germinated legume foods and orange juice, and control group (n=20), receiving 0 µg/d folate from apple juice. Folate status and metabolic profile of plasma were assessed at baseline and 12 weeks.

***Results***: At baseline, groups show normal folate status and no differences in metabolic profile. After intervention, mean erythrocyte folate concentration increased by 310 (*p*<0.0001) and 300 nmol/L (*p*<0.0001) in the folic acid and food group, respectively. Plasma folate increased by 14 (*p*<0.0001) and 12 nmol/L (*p*<0.0001), and homocysteine decreased by 20 (*p*=0.0015) and 14% (*p*= 0.003) in both active groups, whereas no changes were observed in the control group. Multivariate data analysis revealed distinct metabolic patterns for all groups with glycine as the first discriminating variable. Folic acid supplementation increased plasma glycine (37%, *p*=0.02) and betaine (27%, *p*=0.008) after correction for changes in the control group. No changes were observed for both metabolites in the food group.

***Conclusion***: Folate-rich legume foods showed similar high bioefficacy as supplemental folic acid when improving folate status. However, folic acid supplementation affects one-carbon metabolism in healthy women differently compared to natural food folate.

**Disclosure of interest**: None to declare.

### Poster presentation no. P306

#### Nutritional and environmental sustainability of diets using LCA

##### Cornelia Witthöft^1,^*, Elin Röös^2^, Hanna E. Karlsson^2^ and Cecilia Sundberg^3^
^1^Department of Chemistry and Biomedical Sciences, Linnaeus University, Kalmar, Sweden; ^2^Department of Energy and Technology, Swedish University of Agricultural Sciences, Uppsala, Sweden; ^3^Department of Sustainable Development, Environmental Science and Engineering (SEED), KTH - Royal Institute of Technology, Stockholm, Sweden *Presenting author

***Background and aims***: Current dietary habits neither comply with nutrition recommendations nor are they sustainable. When aiming for sustainable consumption habits, it is important to concurrently estimate both environmental impact and nutritional quality. The aim of this study was to present methods to jointly evaluate the environmental impact and the nutritional quality of diets.

***Methods***: The assessment considered both energy content and 18 macro- and micronutrients in the diet, the climate impact, and use and biodiversity damage potential. Three diets were assessed; a diet corresponding to Nordic Nutrition Recommendations (SNO), the current average Swedish food consumption (Riksmaten), and fashion Low Carbohydrate High Fat diet (LCHF). The content of different nutrients in the diet was normalized based on dietary reference values and presented together with the environmental impacts. This novel approach was compared to a method where the nutritional quality of the different diets was considered by calculating their nutrient density score, and the environmental impact was then expressed per nutrient density score.

***Results***: The new approach showed that nutrition recommendations were well met by SNO, while Riksmaten and LCHF resulted in inadequate intake of specific identified nutrients. Food consumption according to the recommended diet SNO would lower the climate impact by 25% as compared to the current diet (Riksmaten), while the LCHF diet would increase emissions by 21%. When condensing the nutritional quality of a diet using nutrient density score, results are highly dependent on how the score is calculated.

***Conclusion***: The new method for concurrent nutritional and environmental assessment of diets could be a tool for meal planning, for the inclusion of environmental aspects into nutrition recommendations and when developing policy instruments for sustainable food systems.

**Disclosure of interest**: None to declare.

### Poster presentation no. P307

#### Methodological considerations in a pilot study on the effects of a berry enriched smoothie on children's performance in school

##### Ulla Rosander^1,^*, Kimmo Rumpunen^2^, Viktoria Olsson^3^, Mikael Åström^4^, Pia Rosander^3^ and Karin Wendin^3,5,6^
^1^School of Education and Environment, Kristanstad University, Kristianstad; ^2^Department of Plant Breeding, Swedish University of Agricultural Sciences, Balsgård; ^3^School of Education and Environment, Kristianstad Univeristy, Kristianstad; ^4^Department of biostatistics, StatCons, Malmö; ^5^SP Technical Research Institute of Sweden, Gothenburg, Sweden; ^6^Department of Food Science, University of Copenhagen, Copenhagen, Denmark *Presenting author

***Background and aims***: In many countries, the consumption of fruit, berries, and vegetables is about half the recommended. Berries contain bioactive compounds that may affect cognitive functions. School children are often hungry and thirsty during the lectures before lunch and this affects performance. Could a berry-smoothie decrease thirst and hunger, and thereby affect school performance? The aim was to investigate if a cross-over design can be used to study the effects of a smoothie on performance in a school setting.

***Methods***: Methodological challenges included developing an appetizing berry-smoothie and choosing a suitable experimental design that could be adapted to school conditions.

In the pilot study, 236 Swedish children aged 10–12 years participated in a cross-over design and were administered either a berry-smoothie or a fruit-based placebo after the midmorning break. Both beverages provided 5% of the daily energy intake. Performance was assessed using the d2 Test of Attention measuring attention span and concentration. Statistical analyses were performed using the Wilcoxon signed rank test in StatXact v 10.3.

***Results***: The consumption of both the smoothie and the placebo increased the attention span and concentration significantly.

***Conclusion***: The children's performance in the d2 Test of Attention was positively affected by beverage consumption. The effect was attributed to the supplementation of water and energy. In this design, the study did not permit any conclusive results regarding the effect of bioactive compounds on performance. In a coming study, a third group, receiving no beverage, should be included aiming to identify the cause of the effect.

**Disclosure of interest**: None to declare.

### Poster presentation no. P308

#### Using the intervention mapping protocol to develop a family-based weight-management program

##### Tonje H. Stea^1,^*, Sveinung Berntsen^1^, Vigdis Guttormsen^1^, Tommy Haugen^1^, Nina C. Øverby^1^, Kristin Haraldstad^1^, Eivind Meland^2^ and Eirik Abildsnes^2^
^1^Department of Public Health, Sport and Nutrition, University of Agder, Kristiansand, Norway; ^2^Department of Global Public Health and Primary Care, University of Bergen, Bergen, Norway *Presenting author

***Background and aims***: In light of the high prevalence of childhood obesity and lack of evidence for effective intervention programs, we have developed a family-based intervention study, which currently is being implemented in Norwegian municipalities (*n*=11). The aim of the study is to improve lifestyle habits among overweight and obese children, aged 6–10 years old, and enhance parental self-efficacy, family engagement, and parent-child relationships.

***Methods***: The Intervention Mapping protocol was used to develop a tailored family-based weight-management program. In order to gather information on local opportunities and barriers, interviews with key stakeholders and a 2-year pilot study was conducted. The main study has used a quasi-experimental controlled design and local-based Healthy Life Centers, and Public Health Clinics are responsible for recruiting families and conducting the intervention study. The effect of the study will be measured both at completion of the 6 months intervention study, and 6 and 18 months after the intervention period.

The research program has Self Determination Theory as a theoretical framework. The behavioral models and educational strategies include individual family counseling meetings, workshops focusing on family structure, nutrition courses, and physical activity groups providing tailored information and practical learning sessions. Parents will be educated on how to use these strategies at home, to further support their children in improving their behaviors.

***Results***: Data collection and analysis will occur 2015–2018.

***Conclusion***: A systematic and evidence-based approach was used for development of this family-based intervention study targeting overweight and obese children, 6–10 years old. This program, if proven effective, may be directly implemented in all municipal health care institutions in Norway.

**Disclosure of interest**: None to declare.

### Poster presentation no. P309

#### Adolescents’ experiences of using a smartphone dietary assessment application

##### Åsa Svensson^1^, Maria Magnusson^2^ and Christel Larsson^3,^* ^1^Department of Food and Nutrition, Umeå University, Umeå, Sweden; ^2^Department of Public Health and Community Medicine, Public Health Epidemiology Unit, Sahlgrenska Academy, University of Gothenburg, Gothenburg, Sweden; ^3^Department of Food and Nutrition, and Sport Science, University of Gothenburg, Gothenburg, Sweden *Presenting author

***Background and aims***: The use of new technology has potential to increase the participation rate in dietary studies and the validity of collected dietary data. However, to evaluate the usability of developed dietary methods, qualitative studies of participants’ experiences and perceptions are needed. The aim was to explore adolescents’ experience of using a newly developed smartphone dietary assessment application, with a focus on factors that could affect their recording of dietary intake.

***Methods***: Focus group interviews were conducted with 75 participants who had used a newly developed smartphone dietary assessment application in a quantitative evaluation study. The interviews were analyzed using qualitative content analysis, and the theoretical framework of Self Determination Theory was applied.

***Results***: The adolescents’ use of the dietary assessment application was characterized by their struggle to overcome several perceived barriers. Facilitators that helped adolescents complete the method were also identified. Motivation was found to be an important facilitator, and the intrinsically motivated participants completed the method because they found it fun to use. The autonomous extrinsically motivated participant completed the method for “the greater good,” to contribute to the study. The controlled extrinsically motivated participants completed the method to get a reward or avoid punishment. Motivated participants did not bother to complete the method. More motivated participants were assumed to be more able to overcome barriers and needed less other facilitators.

***Conclusion***: In future studies that include food records, systematic work should be conducted to minimize identified barriers and promote identified facilitators. Further research should especially aim at studying methods for and effects of increasing intrinsic motivation by supporting autonomy, competence, and relatedness among adolescents asked to participate in dietary studies.

**Disclosure of interest**: None to declare.

### Poster presentation no. P310

#### Food intake biomarkers and the development of type 2 diabetes in 64 years old Swedish women

##### Otto Savolainen^1,^*, Mads Lind^1,2^, Ann-Sofie Sandberg^1^, Björn Fagerberg^3^, Göran Bergström^3^ and Alastair Ross^1^
^1^Division of Food and Nutrition Science, Chalmers University of Technology, Gothenburg, Sweden; ^2^Department of Nutrition, Exercise and Sports, University of Copenhagen, Copenhagen, Denmark; ^3^Wallenberg Laboratory for Cardiovascular Research at the Center for Cardiovascular and Metabolic Research, Sahlgrenska Academy at Gothenburg University, Gothenburg, Sweden *Presenting author

***Background and aims***: The development of type 2 diabetes (T2D) is a multifactorial process that involves inherent factors such as genetics, and lifestyle factors such as physical inactivity and nutrition. Diet is a major risk factor for developing and in prevention of T2D, yet population-based research on understanding the role of diet in T2D development is hampered due to the reliance on dietary recall methods which are subject to recall bias. Food intake biomarkers can be a more reliable tool for estimating diet and add value to FFQ assessments, as they are a non-subjective measure of food intake, yet the use of multiple food intake biomarkers remains largely untested in observational settings. The aim of the current study was to assess how different food intake biomarkers in plasma are related to the development of T2D after 5 years.

***Methods***: GC-MS/MS metabolomics was used to analyze plasma samples from a prospective cohort of 64 year Swedish women. The relationship between biomarkers and T2D risk based on multiple glucose tolerance (GT) tests or new T2D outcome was analyzed using linear models adjusted for BMI and other risk markers of T2D.

***Results***: Plasma concentrations of alkylresorcinols, biomarkers of whole grain wheat and rye intake, a-tocopherol and eicosapentaenoic acid (EPA) were negatively associated (*p*<0.05) with GT status, while a proposed meat intake biomarker -alanine and a possible fish intake biomarker 3-carboxy-4-methyl-5-propyl-2-furanpropanoic acid (CMPF) were positively associated with GT.

***Conclusion***: We found several food intake biomarkers to be associated with GT status or new T2D independent of BMI. This suggests that diet is a key factor in T2D development, and more work on the role of whole grain, fish, meat, and tocopherol intake is warranted.

**Disclosure of interest**: None to declare.

### Poster presentation no. P311

#### Introduction of complementary foods and allergy development in farm and nonfarm children

##### Karin Jonsson^1,^*, Hilde Brekke^2^, Bill Hesselmar^3^, Agnes Wold^4^ and Ann-Sofie Sandberg^1^
^1^Division of Food Science, Department of Biology and Biological Engineering, Chalmers University of Technology, Gothenburg, Sweden; ^2^Department of Nutrition, Institute of Basic Medical Sciences, University of Oslo, Oslo, Norway; ^3^Department of Paediatrics, Institute of Clinical Sciences, Gothenburg, Sweden; ^4^Clinical Bacteriology Section, Department of Infectious Diseases, University of Gothenburg, Gothenburg, Sweden *Presenting author

***Background and aims***: Postponed introduction and a low variety of complementary foods may increase the risk of allergy development. Growing up on farms is protective against allergy development; we aimed to investigate if this protection partly is explained by introduction practices of complementary foods.

***Methods***: Farm (*n=*28) and nonfarm (*n=*37) children from the same rural area were recruited to the FARMFLORA birth-cohort. Practices of introduction of complementary foods were recorded at 6, 12, and 18 months of age; month of introduction was registered for: partial and exclusive breastfeeding, milk and gluten free and regular formulas, potatoes, vegetables, fruits, berries, nuts, peanuts, legumes, eggs, fish, meat, milk, and flour. Allergy at age 3 was diagnosed by pediatricians.

***Results***: Nuts were introduced earlier and peanuts tended to be introduced earlier to farmers than to nonfarmers. One farm and 10 nonfarm children were allergic. Early introduction of any food or formula was associated with allergy; this association was not significant when considering reverse causation, except for as early as one month. When farmers were excluded, the significance disappeared; the duration of breastfeeding tended to be slightly longer in farmers. Patterns of a relationship between allergy and postponed introduction of flour, eggs, and especially fish were observed. A low variety of food groups introduced at age 6 months tended to be associated with allergy, after reverse causation was accounted for.

***Conclusion***: Nuts were introduced earlier to farm than to nonfarm children. However, food introduction practices did not explain the low allergy prevalence in the farm children; although patterns of postponed introduction of fish, eggs, and flour and increased rates of allergy were observed.

**Disclosure of interest**: None to declare.

### Poster presentation no. P312

#### Diet in 1-year-old farm and nonfarm children and allergy development

##### My Green^1,^*, Karin Jonsson^1^, Agneta Sjöberg^2^, Hilde Brekke^3^, Bill Hesselmar^4^, Agnes Wold^5^ and Ann-Sofie Sandberg^1^
^1^Division of Food Science, Department of Biology and Biological Engineering, Chalmers University of Technology, Gothenburg, Sweden; ^2^Department of Food and Nutrition, and Sport Science, University of Gothenburg, Gothenburg, Sweden; ^3^Department of Nutrition, Institute of Basic Medical Sciences, University of Oslo, Oslo, Norway; ^4^Department of Paediatrics, Institute of Clinical Sciences, Gothenburg, Sweden; ^5^Clinical Bacteriology Section, Department of Infectious Diseases, University of Gothenburg, Gothenburg, Sweden *Presenting author

***Background and aims***: The allergy prevalence is markedly low in farm children. We have previously shown that farm mothers consumed more whole fat dairy products and saturated fat, and less margarines and oils during pregnancy and lactation; a high margarine and oil intake was associated with allergy in the children. Our aim was to evaluate the diet in these children at age 1 year and allergy development.

***Methods***: Twenty-eight farm and 37 nonfarm children from the same rural area were followed in the FARMFLORA birth-cohort. The diet (except added salt) was assessed by 24-h recalls followed by 24-h food diaries. Allergy was diagnosed at age 3 years by pediatricians. The children's diet was related to farm residence and allergy development, and to the Nordic Nutrition Recommendations.

***Results***: Farm children consumed more farm milk, cream, saturated fat, and fat in total than nonfarmers, as well as more oily fish. Nonfarm children had a pattern of a higher intake of margarines, oils, fruits, and vegetables. One farm and 10 nonfarm children developed allergy. Healthy children had a higher intake of seafood than allergic children. Among nonfarmers, allergic children consumed more pork. The median intake of micronutrients in the study population as a whole exceeded the recommended intake for all nutrients except for vitamin D, selenium, and iodine. The only food group that correlated positively with intake of iron was purchased porridge or formula, while foremost milk intake correlated negatively with the iron intake.

***Conclusion***: Farm children consumed more cream and saturated fat than nonfarmers – in line with the mothers’ diet in our previous study. In addition, farm children consumed more oily fish than nonfarmers, which was also the case for healthy children who consumed more seafood than allergic children. Except for a few micronutrients, the children had a satisfactory intake of vitamins and minerals when compared with the Nordic Nutrition Recommendations.

**Disclosure of interest**: None to declare.

### Poster presentation no. P313

#### Variation in modeled healthy diets based on three different food patterns identified from the Danish national diet – and the impact on carbon footprint

##### Ellen Trolle^1,^*, Anne V. Thorsen^2^, Lisbeth Mogensen^3^ and Tue Christensen^1^
^1^National Food Institute, Soeborg, Denmark; ^2^National Food Institute, Technical University of Denmark, Soeborg, Denmark; ^3^Department of Agroecology, Aarhus University, Aarhus, Denmark *Presenting author

***Background and aims***: A healthy diet complies with the national food-based dietary guidelines (FBDG) and Nordic nutrition recommendations (NNR2012). In this study, we aim at 1) developing new healthy diet compositions by a simple diet-modeling technique that ensures a nutrient content in accordance with the recommended values and depending on food preferences and habits, and 2) further optimizing the diet composition with regard to carbon footprint (CF).

***Methods***: We used a simple modeling of the “traditional,” “health conscious,” and “fast food” patterns identified from national dietary data into isocaloric healthy diets that fulfill and the Danish FBDGs and NNR2012 with respect to both micro- and macronutrients. Furthermore, we updated the list of estimated carbon footprint (CF) of food items included in the diets and further optimized the diet composition with regard to CF. Extension of modeling was used to optimize the diets with regard to their estimated carbon footprint (CF).

***Results***: Around 365 food items are included in the three food patterns. Based on literature, CF of these foods is updated, including the contribution from waste, transportation, and cooking at home. Despite variation in the amounts of contribution of foods in each food group and in the composition of foods within each food group, the estimated CFs of the modeled healthy dietary patterns are similar to original Danish patterns. CFs of the CF-optimized dietary patterns similar to each other, and CF of CF-optimized dietary patterns are approximately 25% lower. Only a small contribution to CF from transportation and cooking at home.

***Conclusion***: Different dietary patterns can fulfill dietary recommendations. Specific optimization is needed to lower the CF of the diets.

**Disclosure of interest**: None to declare.

### Poster presentation no. P402

#### Differences in health behavior at 1-year follow-up between attendees of two screening modalities having a negative screening test result, and controls

##### Markus D. Knudsen^1,2,3^*, Anette Hjartåker^1^, Geir Hoff ^2,3,4^, Thomas De Lange^2,5^, Tomm Bernklev^3,6^ and Paula Berstad^2,3^
^1^Department of Nutrition, Institute of Basic Medical Sciences, University of Oslo, Oslo, Norway; ^2^Department of Bowel Cancer Screening, Cancer Registry of Norway, Oslo, Norway; ^3^Department of Research and Development, Telemark Hospital, Skien, Norway; ^4^Department of Health Management and Health Economics, Institute of Health and Society, University of Oslo, Oslo, Norway; ^5^Department of Research, Bærum Hospital, Vestre Viken Hospital Trust, Sandvika, Norway; ^6^Department of Clinical Medicine, University of Oslo, Oslo, Norway *Presenting author

***Background and aims***: Nine out of ten participants in colorectal cancer (CRC) screening have a negative screening test result. It has been hypothesized that getting a negative screening test result may reduce incentives to strive for a healthy lifestyle. The purpose of the present study was to investigate potential differences in changes of health behavior at 1-year follow-up between screen-negative attendees to two different screening modalities and controls not invited to screening.

***Methods***: Participants of both genders, aged 50–74 years, were invited to complete a self-reported lifestyle questionnaire (LSQ) on smoking, body weight, physical activity, alcohol intake, and selected dietary items at baseline and at 1-year follow-up. Participants were randomly assigned to have a fecal immunochemical test for blood (FIT), flexible sigmoidoscopy (FS), or no screening (controls). A total of 14,832 were invited to be screened by FIT or FS; 1,809 and 1,327 individuals tested negative with FIT and FS, respectively. These 3,136 participants also completed the LSQ along with 1,029 of 7,000 controls invited only to complete the LSQ. Differences in changes of health behavior during follow-up between the arms were analyzed using ANCOVA (95% confidence intervals, CI).

***Results***: Participants with a negative FIT screening test result reduced their alcohol consumption significantly more than controls during 1-year follow-up; difference –0.29 glass/week, (95% CI; –0.54 to –0.04). Body weight decreased more in participants with a negative screening test result in the FS arm than in the FIT arm during the 1-year follow-up; difference –0.31 kg, (95% CI; –0.55 to –0.08).

***Conclusion***: The present study does not suggest unfavorable short-term consequences in health behavior after getting a negative CRC screening test result.

**Disclosure of interest**: None to declare.

### Poster presentation no. P403

#### Diet quality in later life: the importance of social factors

##### Ilse Bloom^1,2^*, Karen Jameson^1^, Holly Syddall^1^, Elaine Dennison^1^, Catharine Gale^1,3^, Janis Baird^1^, Cyrus Cooper^1^, Avan A. Sayer^1,4^ and Sian Robinson^1^
^1^MRC Lifecourse Epidemiology Unit, University of Southampton, Southampton, United Kingdom; ^2^Southampton Biomedical Research Centre (in Nutrition), National Institute for Health Research, Southampton, United Kingdom; ^3^Centre for Cognitive Ageing and Cognitive Epidemiology, Department of Psychology, University of Edinburgh, Edinburgh, United Kingdom; ^4^Institute of Neuroscience, Newcastle University, Newcastle upon Tyne, United Kingdom *Presenting author

***Background and aims***: Poor nutrition is common in older people, but relatively little is known about influences on food choice at this age. The aim of the present study was to examine psychosocial influences and their cross-sectional relationships with diet quality in a UK cohort of older community-dwelling men and women.

***Methods***: Between 1998 and 2003, the diets of 1,048 men and 862 women aged 59–73 years, taking part in the Hertfordshire Cohort Study, were assessed by administered food frequency questionnaire. A prudent diet score, derived from a principal component analysis of the dietary data, was calculated for each participant and was used as an indicator of diet quality. High scores indicated frequent consumption of fruit, vegetables, wholegrain cereals, and oily fish. A range of psychosocial factors was assessed using standard questionnaires.

***Results***: Mean prudent diet score was significantly lower in men than in women. In both men and women, diet quality was positively related to social support; specifically, with greater confiding/emotional support. In men but not in women, greater practical support was also associated with a higher diet score. In contrast, a large social network and feeling close to many people were both associated with higher diet scores in women, but not in men. For both men and women, higher overall participation in leisure activities was related to higher diet scores; increased participation in activities of a more cognitive nature, as well as in activities of a more social nature were both associated with a higher diet score.

***Conclusion***: In community-dwelling older adults, a range of social factors that include increased social support, a large social network and increased participation in social and cognitive leisure activities, are associated with better quality diet. Understanding how social factors influence diet in later life will be important for the development of interventions to promote diet quality in older people.

**Disclosure of interest**: None to declare.

### Poster presentation no. P404

#### Nut consumption – cardiovascular health benefits outweigh the carcinogenic effects attributed to aflatoxin exposure

##### Hanna Eneroth^1^*, Karin Leander^2^, Irene Mattisson^1^, Cecilia Nälsén^1^, Stina Wallin^1^ and Agneta Åkesson^2^
^1^Department of Risk Benefit Assessment, National Food Agency, Uppsala, Sweden; ^2^Institute of Environmental Medicine (IMM), Karolinska Institutet, Stockholm, Sweden *Presenting author

***Background and aims***: Nuts are rich in polyunsaturated fatty acids, dietary fiber, and micronutrients but may also be a major source of aflatoxin B1 –a potent liver carcinogen. Although mounting evidence from interventions and prospective cohorts shows that nut consumption reduces cardiovascular disease (CVD) incidence, the balance of risk and benefit has not been fully evaluated. We assessed the impact of increasing nut consumption from the average of 5 to 30 g/day on the estimated number of diagnoses of CVD and liver cancer in the Swedish population.

***Methods***: Based on results from the Spanish PREDIMED-intervention trial on consumption of mixed nuts in relation CVD risk and the national population disease statistics in 2013, we estimated the number of yearly cases attributed to low nut consumption in the Swedish population, aged 50–79 years. By using the Joint FAO/WHO Expert Committee on Food Additives’ approximation of 1 ng aflatoxin B1/kg/bw/day corresponding to an extra risk of cancer of 1 in 10 million/year, we estimated additional cases of liver cancer at 30 g nuts/day.

***Results***: Totally 27,938 first events of non-fatal or fatal CVD occurred in the patient register in 2013. The estimated yearly number of CVD cases preventable by increased nut consumption was 3,382 (95% confidence interval 383–5,556). In total, 544 new cases of liver cancer occurred during the same year. Increased nut consumption may result in 2.7 ng/bw/day aflatoxin exposure corresponding to approximately three additional cases yearly.

***Conclusion***: Based on our assumptions, increased nut consumption would only lead to a few additional cases of liver cancer each year in the Swedish population, whereas a substantially lower number of CVDs could be expected. Thus, the population health benefits provided by increased nut consumption seems to outweigh the risk associated with increased aflatoxin B1 exposure.

**Disclosure of interest**: None to declare.

### Poster presentation no. P405

#### Reproducibility and relative validity of a web-based food frequency questionnaire for Danish adolescents

##### Anne A. Bjerregaard^1^*, Thorhallur I. Halldorsson^1,2^, Sjurdur F. Olsen^1^ and Inge Tetens^3^
^1^Center for Fetal Programming, Department of Epidemiology Research, Statens Serum Institut, Copenhagen, Denmark; ^2^The Unit for Nutrition Research, Faculty of Food Science and Nutrition, School of Health Sciences, University of Iceland, Reykjavik, Iceland; ^3^Risk-Benefit Research Group, National Food Institute, Technical University of Denmark, Søborg, Denmark *Presenting author

***Background and aims***: Examining the relationship between adolescent diet and later health and risk of disease, it is of pivotal importance to develop methods that can assess dietary intake with high precision and accuracy. A self-administered web-based FFQ was developed to examine dietary habits in offsprings in the Danish National Birth Cohort (DNBC). In this study, we evaluate the reproducibility and relative validity of the FFQ among Danish adolescents aged 12–15 years by comparing dietary intake with that of three 24 HR (hazard ratio).

***Methods***: In a nested cohort within the DNBC, 178 participants aged 12–15 years, who completed a 145 food item web-based FFQ, were invited to complete three telephone-based 24 HRs. Of these, 100 were invited to complete a second FFQ.

***Results***: Comparing FFQ1 and FFQ2 revealed that adolescents to a high degree were able to recall overall dietary habits (89–94% agreement). Comparing FFQ1 with 3×24 HRs, classification into the same and opposite quartile for food groups were 35 and 7.5%, respectively. Mean adjusted Spearman correlation was 0.29 for food groups and 0.43 for nutrients. Although, we found suggestions for both under- and overestimation by the FFQ compared to the 24 HRs, mean energy and carbohydrate intake did not differ significantly between the two methods. Overall, no significant differences were observed in intake between the two FFQs.

***Conclusion***: The reproducibility of the FFQ was acceptable, and the adolescents were capable of consistently recalling overall dietary habits and had some difficulties estimating frequency of consumption of regularly consumed food items. Compared with 3×24 HRs, the FFQ can measure intake of energy, macronutrients, and most food groups. Overall, the FFQ and the 24 HR showed reasonable agreement in line with that of other studies among adolescents.

**Disclosure of interest**: None to declare.

### Poster presentation no. P406

#### The impact of vitamin D food fortification on VDSP- standardised serum 25(OH)D in general population in Finland - an 11 year follow-up

##### Suvi Itkonen^1^*, Tuija Jääskeläinen^2^, Annamari Lundqvist^2^, Satu Männistö^2^, Maijaliisa Erkkola^1^ , Kevin Cashman^3^ and Christel Lamberg-Allardt^1^
^1^Calcium Research Unit, Division of Nutrition, Department of Food and Environmental Sciences, University of Helsinki, Helsinki, Finland; ^2^National Institute for Health and Welfare, Helsinki, Finland; ^3^Cork Centre for Vitamin D and Nutrition Research, School of Food and Nutritional Sciences, University College Cork, Cork, Ireland *Presenting author

***Background and aims***: The current, voluntary fortification of fluid milk products (10 µg/l) and fat spreads (20 µg/100 g) started in 2010 in Finland. Dietary vitamin D intake has improved after fortification, but at the same time, the use of vitamin D supplements has increased. Here, we aimed to examine the impact of vitamin D fortification on vitamin D status in Finnish adults between 2000 and 2011.

***Methods***: Our nationally representative sample comprised adults aged ≥30 years belonging to the Health 2000 Survey cohort and its follow-up, the Health 2011 Survey. Serum 25-hydroxyvitamin D concentration (S-25(OH)D) was determined by radioimmunoassay in Health 2000 (*n=*6,134) and by chemiluminescent microparticle immunoassay in Health 2011 (*n*=4,102). The results from Health 2011 were standardized by re-analyzing and re-calibration according to the Vitamin D Standardization Program (VDSP) by LC-MS/MS. Linear regression models were used to assess the impact of fortification on the change in S-25(OH)D (adjusted for sex, age, and season of blood sampling).

***Results***: Preliminary results show that mean S-25(OH)D increased from approximately 45 nmol/l to 75 (standardized 67) nmol/l during the follow-up. In 2000, 10% of subjects used vitamin D supplements, while in 2011 the percentage was 38%. The greatest increase in S-25(OH)D was found among supplement users who also consumed milk. Among non-supplement users, the increase among milk consumers was significantly greater than among those who did not use milk (*P*<0.001).

***Conclusion***: The consumption of vitamin D-fortified fluid milk products was effective in increasing S-25(OH)D concentrations, especially among participants who did not use vitamin D supplements, showing fortification to be an effective strategy in improving vitamin D status at population level. The Health 2000 results will further be standardized accordingly, to eliminate the impact of difference in assays.

**Disclosure of interest**: None to declare.

### Poster presentation no. P407

#### Association between physical home environmental factors and vegetable consumption among Norwegian 3–5 year old children

##### Anne L. Kristiansen*, Mona Bjelland, Anne Himberg-Sundet, Nanna Lien and Lene F. Andersen Department of Nutrition, University of Oslo, Oslo, Norway *Presenting author

***Background and aims***: Associations between home environmental factors and vegetable consumption among preschool children are so far understudied. The aim of the present study was to explore associations between physical home environmental factors and vegetable consumption among Norwegian 3–5 year old children.

***Methods***: To assess the child's vegetable intake and physical home environmental factors, the parent filled in a web-based questionnaire. Linear regression was applied to assess the relationship between vegetable intake and “availability at home,” “accessibility at home,” “serving barriers,” and “purchase barriers.”

***Results***: Parental consent was obtained for 633 children (response rate 38.8%). “Availability at home” were positively associated with an increase in variation, frequency, and amount of vegetables consumed (Table 1). Further, “serving barriers” were negatively associated with variation, frequency, and amount of vegetables consumed.

***Conclusion***: Within the physical home environment of this sample, there were modifiable factors both promoting and hindering vegetable consumption.

**Abstracts P407–Table 1:** *B*-values and 95% confidence interval for adjusted bivariate associations^a^ between vegetable consumption of 3–5 year old children and physical home environment factors in the BRA-study

Factor	Variation in vegetable intake (numbers per month)^b^	Frequency of vegetable intake (times per day)^b^	Amount of vegetables (gram/day)^c^
“Availability at home”	**2.27 (1.69, 2.85)**	**1.14 (0.91, 1.37)**	**42.5 (25.8, 59.3)**
“Accessibility at home”	0.44 (−0.08, 0.96)	**0.38 (0.17, 0.59)**	**17.6 (3.0, 32.1)**
“Serving barriers”	**−2.02 (−2.60, −1.44)**	**−0.98 (−1.22, −0.75)**	−**38.5 (**−**55.5**, −**22.4)**
“Purchase barriers”	−0.10 (−0.52, 0.33)	−0.15 (−0.33, 0.02)	−4.1 (−16.4, 8.1)

a *B* are adjusted for maternal education, child gender and child birth year.

b Total number of children *n*=395.

c Total number of children *n*=197. Values in bold are significant associations.

**Disclosure of interest**: None to declare.

### Poster presentation no. P408

#### Gestational weight gain and dietary intake in the population-based GraviD-study

##### Sara Palm, Frida Rökaas, Linnea Bärebring and Hanna Augustin* Department of Internal Medicine and Clinical Nutrition, University of Gothenburg, Salhgrenska Academy, Gothenburg, Sweden *Presenting author

***Background and aims***: High body mass index (BMI) before pregnancy and high gestational weight gain (GWG) is related to increased risks for mother and child. Therefore, the Institute of Medicine (IOM) has developed BMI-specific guidelines for GWG. Dietary intake may be one modulating factor of GWG, but findings are inconclusive. The aim was to investigate GWG and its relation to dietary intake in the population-based GraviD-study.

***Methods***: In total 1,765 pregnant women were included in this study. Women were recruited in first trimester (T1) at the antenatal care in Gothenburg, Södra Bohuslän, and Södra Älvsborg in 2013–2014. Dietary intake was assessed in third trimester (T3) by the web-based food frequency questionnaire MealQ (*n*=787). Body weight was retrieved from medical records, and GWG was calculated as weight in T3 (week 37) minus weight in T1. Additional background characteristics and pre-pregnancy weight were collected by study questionnaires at inclusion.

***Results***: In T1, mean BMI was 24.5 kg/m^2^, and 63% of the women were normal weight, 25% overweight, and 10% obese. Mean±SD GWG was 13.3±4.9 kg. In total, 36% had gained excessively by gestational week 37, and 40% had gained in line with the IOMs guidelines.

Subgroup analysis among the women who performed MealQ showed that women who gained excessively reported a significantly less frequent intake of fish and a lower intake of fiber, compared to women who gained in line with the IOM guidelines. Women who gained excessively also had a higher BMI before pregnancy than women who gained within the recommendations.

***Conclusion***: In gestational week 37, more than one third of the women had a higher GWG than recommended by the IOM. The women who gained excessively had a higher pre-pregnancy BMI and reported a lower intake of fish and fiber in the third trimester of pregnancy.

**Disclosure of interest**: None to declare.

### Poster presentation no. P409

#### Marketing and promotion of branded food items by adolescent Instagram users

##### Christopher Holmberg^1^* on behalf of EpiLife, John E Chaplin^2^ on behalf of EpiLife, Thomas Hillman^3^, Christina Berg^1^ on behalf of EpiLife and EpiLife – Gothenburg's center for epidemiologic studies on mental health and physical health interacting over the life course ^1^Department of Food & Nutrition, and Sport Science, University of Gothenburg, Gothenburg, Sweden; ^2^Sahlgrenska Academy, Gothenburg University, Gothenburg, Sweden; ^3^Department of Education, Communication and Learning, University of Gothenburg, Gothenburg, Sweden *Presenting author

***Background and aims***: Social media is ubiquitous in the lives of adolescents, and research shows that communication between youths in social media settings can have an impact on their food intake. These networks and applications also provide distinctive opportunities to study adolescents’ dietary communication without interfering with it. The current study aimed to explore that how adolescents communicate images containing food in a commonly used visual-based social media application, Instagram.

***Methods***: To locate adolescent Instagram users, we initiated the search from images appended with the hashtag #14år (“14 years”). This hashtag had been appended to 3,479 images as of March 2014. Though, as users alter their privacy settings, delete their accounts, or change their user names, 1,358 images were not retrievable. We also excluded accounts that we judged did not belong to adolescents (based on written and visual profile information); 1,001 unique Instagram users’ photo streams were thus eligible for analysis. We used content analysis to classify food items and categorize these based on types of food and how the food items were presented by the uploaders.

***Results***: A majority of adolescent users (85%) shared images depicting food items. Most of the images (67.7%) portrayed foods high in calories but low in nutrients. Nearly half of these images were arranged as a still life with food brand names visibly exposed. Numerous of these images were influenced by major food marketing campaigns.

***Conclusion***: The adolescent users’ themselves created images mimicking food advertisements. This has dietary health promotion implications as it becomes more difficult to screen and approach exposure to marketing of unhealthy foods to youths in these widespread networks. Shared images contain personal endorsements, which mean that they may be more influential than commercial food advertising.

**Disclosure of interest**: None to declare.

### Poster presentation no. P410

#### Food patterns, inflammation markers, and risk of post-menopausal breast cancer; a nested case-control study from the Malmö Diet and Cancer cohort

##### Joana Dias^1^*, Isabel Drake^1^, Ulrika Ericson^1^, Bo Gullberg^1^, Bo Hedblad^1^, Gunnar Engström^1^, Signe Borgquist^2^, Jan Nilsson^1^, Gunilla Fredrikson^1^ and Elisabet Wirfält^1^
^1^Department of Clinical Sciences in Malmö, Lund University, Malmö, Sweden; ^2^Department of Clinical Sciences in Lund, Lund University, Lund, Sweden *Presenting author

***Background and aims***: Dietary patterns derived with reduced rank regression (RRR) are linear combinations of food group intakes that explain the maximum variation in a set of response variables (RV). The aim was to assess if food patterns (FP) associated with inflammation markers were associated with invasive post-menopausal breast cancer risk.

***Methods***: A nested case-control study within the Malmö Diet and Cancer (MDC) consisted of women aged 55–73 years, free from cancer at baseline (1991–1996). The RV were three inflammation markers (oxidized-LDL, IL-1β, and TNF-α) analyzed in plasma samples of 446 cases (diagnosed with invasive breast cancer until December 31st 2010) and 910 controls (2 per case, matched on age and date of measurement). A modified diet-history method of high relative validity collected dietary information at baseline.

We derived FP (from 41 food groups) associated with the three RV using RRR. We calculated the odds ratios (OR) for breast cancer risk comparing the higher FP tertiles to the lowest using multivariable unconditional logistic regression. Adjustments were performed for the matching variables, smoking, BMI, waist hip ratio, parity, menopausal hormone therapy (MHT), alcohol, physical activity, and education.

***Results***: Three derived FP explained 3.4% of the total variation in the RV. The first FP explained 1.9% of the variation. Food pattern 3 was characterized by high consumption of soft and light margarines, cottage cheese, fatty fish, lean meats, coffee and water, and low consumption of all ready to eat powders, fiber-rich bread, and sweets. The highest tertile of this FP was associated with increased breast cancer risk compared to the lowest (*p*-trend=0.04), but associations were attenuated with adjustments.

***Conclusion***: We identified three dietary patterns associated with inflammation markers (ox-LDL, IL-1β, and TNF-α). Food pattern 3 was associated with increased breast cancer risk.

**Disclosure of interest**: None to declare.

### Poster presentation no. P411

#### Food choices among adolescents

##### Maria N. Tell*, Mats Nilsson, Marie Golsäter and Hans Lingfors Futurum, Region Jönköping County, Jönköping, Sweden *Presenting author

***Background and aims***: Healthy dietary pattern is found to be associated to other healthy lifestyle behaviors. Associations between choices of certain foods have previously been described among young children. However, there is a scarce knowledge of such relations among adolescents. The aim is to describe how choices of different foods correspond to each other among adolescents.

***Methods***: A cohort of 2,045, 16 year-old upper secondary school students was examined with a health questionnaire used in School Health Service 2009–2010. The responses from a health questionnaire about food frequency were divided into three groups based on the Nordic Nutrition Recommendations 2012 (NNR 2012); healthy choice, moderately healthy, and unhealthy choice. The data were analyzed both with correspondence analysis and logistic regression (uni- and multivariable).

***Results***: The results indicate that most of the healthy and moderately healthy choices of different foods were closely linked to each other, while the unhealthy choices were scattered further apart according to the correspondence analysis. There is one exception; unhealthy choice of sandwich fat seemed to have a close link with healthier selections of food choices.

A high intake of fruit and vegetables was most closely associated to other healthy food choices.

***Conclusion***: There are connections between choices of different food groups among 16-year-old adolescents. A more thorough understanding of choices between different foods could be of importance when designing enquiries for discerning indicators of healthy dietary habits and perhaps also be of importance when trying to promote a healthy diet.

**Disclosure of interest**: None to declare.

### Poster presentation no. P412

#### Changes in sociademographic and lifestyle differences in vitamin D status in Finland: the results from the national health 2000 and health 2011 surveys

##### Tuija Jääskeläinen^1^*, Suvi Itkonen^2^, Annamari Lundqvist^1^, Satu Männistö^1^, Maijaliisa Erkkola^2^ and Christel Lamberg-Allardt^2^
^1^Department of Health, National Institute for Health and Welfare, Helsinki, Finland; ^2^Calcium Research Unit, Division of Nutrition, Department of Food and Environmental Sciences, University of Helsinki, Helsinki, Finland *Presenting author

***Background and aims***: In the Finnish population, vitamin D status has been low, and it has also varied according to sociodemographic and lifestyle factors. To improve vitamin D status at the population level, fortification of fluid milk products and fat spreads with vitamin D was begun in 2003 and was increased further in 2010. The aim of the study was to examine whether sociodemographic and lifestyle differences in vitamin D status have changed in the Finnish general population between 2000 and 2011 (i.e. after fortification).

***Methods***: The study population comprised individuals aged ≥ 30 years from the nationally representative Health 2000 and its follow-up the Health 2011 Surveys. Serum 25-hydroxyvitamin D concentration (25(OH)D) was determined from frozen samples (−70 °C) by radioimmunoassay in Health 2000 (n=6134) and by chemiluminescent microparticle immunoassay in Health 2011 (n=4102). Linear regression models were used to assess the adjusted (age, sex, and month of blood sampling) means.

***Results***: The mean serum 25(OH)D concentration was approximately 45 nmol/l in 2000 and 76 nmol/l in 2011. No differences between sexes were observed in either year. In 2000, higher serum 25(OH)D concentrations were associated with older age, living in southern Finland, being married, and higher education. By 2011, these differences were diminished. The association between higher serum 25(OH)D concentrations and healthy lifestyle (normal weight, leisure-time physical activity, and non-smoking) was observed in both years.

***Conclusion***: Vitamin D status has significantly improved among Finnish adult population after increased food fortification with vitamin D. While sociodemographic differences in vitamin D status have successfully diminished, lifestyle differences have remained almost unchanged.

**Disclosure of interest**: None to declare.

### Poster presentation no. P413

#### Nutritional status and cognitive function in 6–8 months old infants in rural Uganda

##### Grace Muhoozi^1,^*, Prudence Atukunda^2^, Robert Mwadime^2^, Per O. Iversen^3^ and Ane Westerberg^4^
^1^Kyambogo University, Kampala, Uganda; ^2^Makerere University, Kampala, Uganda; ^3^Nutrition, University of Oslo, Oslo, Norway; ^4^Kristiania University College, Oslo, Norway *Presenting author

***Background and aims***: Undernutrition continues to pose challenges to Uganda's children, but there is limited knowledge on its association with physical and intellectual development. We assessed nutritional status and milestone development of 512 children (6–8 months) in South-Western Uganda.

***Methods***: Data of background variables were collected using a questionnaire. Bayley Scales of Infant and Toddler Development (BSID III), and Ages and Stages questionnaires (ASQ) were used to assay child development. Anthropometry was used to determine z-scores for weight-for-age (WAZ), length-for-age (LAZ), weight-for-length (WLZ), and head circumference (HCZ).

***Results***: Prevalence of underweight, stunting, and wasting was 12.1, 24.6, and 4.7%, respectively. Household head education, gender, sanitation, household size, maternal age and education, birth order, poverty likelihood, and dietary diversity scores (DDS) were associated (*p*<0.05) with WAZ, LAZ, and WLZ<2SD. Gender, sanitation, DDS, and poverty were predictors (*p*<0.05) of undernutrition. BSID III indicated development delay of 1.3% in cognitive and language, and 1.6% in motor development. The ASQ indicated delayed development of 24, 9.1, 25.2, 12.2, and 15.1% in communication, fine motor, gross motor, problem solving, and personal social ability, respectively. All nutritional status indicators except HCZ were positively (*p*<0.05) associated with development domains.

***Conclusion***: Undernutrition among the infants was associated with household sanitation, poverty, and low DDS; development domains were positively and significantly associated with nutritional status.

**Disclosure of interest**: None to declare.

### Poster presentation no. P414

#### Factors influencing small child feeding decisions among Kenyan mothers

##### Lauriina Schneider^1,^*, Marja Mutanen^1^, Sari Ollila^2^, Judith Kimiywe^3^ and Crippina Lubeka^4^
^1^Food and Nutritional Sciences, Helsinki, Finland; ^2^Economics and Management, University of Helsinki, Helsinki, Finland; ^3^Food, Nutrition and Dietetics, School of Applied Human Sciences Complex, Nairobi, Kenya; ^4^Food, Nutrition and Dietetics, School of Applied Human Sciences, Kenyatta University, Nairobi, Kenya *Presenting author

***Background and aims***: Much work has been done to improve the health and nutrition of young children in Kenya, but yet the stunting and mortality rates remain rather high.

The present study set out to investigate what issues mothers struggle with in providing their children with proper care and nutrition.

***Methods***: Focus group discussions (FGDs) were held in three mother and child health centers (MCHCs) in slum areas of Nairobi and in three MCHCs in rural areas of Machakos county, Kenya in September 2015. Altogether, 18 FGDs were held with caretakers of children under the age of 2 and with healthcare workers (HCW). The MCHCs were selected by convenient sampling and the individual participants randomly. Discussion guides included breastfeeding- and complementary feeding practices, difficulties faced, sources of information, and sources turned toward for assistance.

After transcription and translation into English, the data were coded into categories reflecting the topics in the discussion guides.

***Results***: The FGDs identified four factors that affected the mother's knowledge and thereby her ability to make decision about child feeding. These were the HCW's knowledge, believes presented by relatives and neighbors, the mothers practical ability to provide food, and the HCW's authority. Also support was important. This came from relatives, HCWs, and husbands. These factors affected to different degrees in the two study areas.

***Conclusion***: Mothers are the primary decision makers when it comes to her child's care and feeding. Many of them actively seek information on the best child feeding practices, but reliable information sources are lacking as HCWs are too busy or incompetent. Due to the strong authority of the HCWs and the increasing influence of media and Internet, it would be important to provide the HCWs with a solid foundation of knowledge about proper young child feeding practices and find new ways of properly educating mothers.

**Disclosure of interest**: None to declare.

### Poster presentation no. P415

#### Effectiveness of a weight loss intervention among postpartum women: results from the randomized controlled LEVA in Real Life trial

##### Ena Huseinovic*, Anna Winkvist, Fredrik Bertz and Hilde Kristin Brekke Internal Medicine and Clinical Nutrition, Institute of Medicine, Gothenburg, Sweden *Presenting author

***Background and aims***: Reproduction has been identified as a trigger for long term weight gain among women. The aim of the current trial is to evaluate the short- and long term effectiveness of a diet behavior modification treatment to produce weight loss among postpartum women within the primary health care setting in Sweden.

***Methods***: During 2011–2014, 110 women with a self-reported body mass index (BMI) of ≥27 kg/m^2^ at 6–15 week postpartum were randomly assigned to diet behavior modification group (D-group) or control group (C-group). Women randomized to D-group (*n*=54) received a 12-wk diet behavior modification treatment by a dietician and were instructed to gradually implement a diet plan based on the Nordic Nutrition Recommendations and to self-weigh ≥3 times per week. Women randomized to C-group (*n*=56) were given a brochure on healthy eating. The primary outcome was change in body weight after 12 weeks and 1 year.

***Results***: At baseline, women had a median (1^st^; 3^rd^ quartile) BMI of 31.0 (28.8; 33.6) kg/m^2^ and the majority (84%) were breastfeeding. After 12 weeks, D-group had a weight change of −6.1 (−8.4; −3.2) kg as compared to −1.6 (−3.5; −0.4) kg in C-group, *p*<0.001. This difference was maintained at the 1 year follow-up with D-group having a weight change of −10.0 (−11.7; −5.9) kg as compared to −4.3 (−10.2; −1.0) kg in C-group, *p*=0.004. Likewise, D-group reduced their BMI, waist circumference, hip circumference, and body fat percentage more than did C-group at both 12 weeks and 1 year (all *p*<0.050). The retention rate was 91 and 85%, respectively.

***Conclusion***: A low intensity diet treatment delivered by a dietitian within the primary health care setting can produce clinically relevant and sustainable weight loss among postpartum women with overweight and obesity.

**Disclosure of interest**: None to declare.

### Poster presentation no. P416

#### Evaluation of the utility of children's and adolescents’ self-reported physical activity level using accelerometers as the reference

##### Anine C. Medin* and Lene F. Andersen Department of Nutrition, University of Oslo, Oslo, Norway *Presenting author

***Background and aims***: Physical activity (PA) and diet are both important modifiable factors that can prevent several chronic diseases; accurate assessments of these are therefore of interest. Objective measures of PA are considered superior to questionnaires, yet the latter are important and much in use due to issues of feasibility and limited resources. Our aim was to evaluate questions addressing self-reported PA, currently being used in a Norwegian dietary survey among children and adolescents, by using accelerometers as an objective reference instrument.

***Methods***: A total of 194 children and adolescents (8–14 years) from Baerum, Norway, wore an accelerometer (Actigraph GT3X+) for 7 days and subsequently completed a Questback form with questions addressing their PA level. Questions were as follows: Q1: “Excluding school hours: How often do you do sports or exercise until you sweat or breathe faster than normal?”; Q2: “How many times per week do you walk or ride your bike TO school?”; Q3: “How many times per week do you walk or ride your bike home FROM school?”. Associations between the self-reported PA and objective measures were assessed.

**Results**: One-way ANOVA showed a significant positive association between increasing PA, measured as counts/min and increased self-reported PA (Q1), *p*<0.001. Q2 and Q3 were not associated with increased PA levels.

***Conclusion***: Q1, but not Q2 and Q3, may alone be utilized as a marker of PA-levels among 8–14 year old children and adolescents in Norway. Still, Q2 and Q3 add interesting contextual data, and may be improved by questioning about the duration of the reported activities related to school transportation.

**Table 1:** Associations between reported PA from Q1 and counts/min from an accelerometer

	Counts/min		
	
Reported PA	N	Mean	SD
Once a week or less	43	556	133
2–3 times per week	92	617	147
4–6 times per week or more	59	676	161
Total	194	621	154

**Disclosure of interest**: None to declare.

### Poster presentation no. P417

#### Vitamin D status during pregnancy in a multiethnic population-based Swedish cohort

##### Linnea Bärebring^1^*, Inez Schoenmakers^2^, Anna Glantz^3^, Lena Hulthén^1^, Åse Jagner^3^, Joy Ellis^4^, Maria Bullarbo^5^ and Hanna Augustin^1^
^1^Department of Internal Medicine and Clinical Nutrition, University of Gothenburg, Salhgrenska Academy, Gothenburg, Sweden; ^2^Medical Research Council Human Nutrition Research, Cambridge, United Kingdom; ^3^Antenatal Care, Primary Health Care, Gothenburg; ^4^Antenatal Care, Primary Health Care, Södra Bohuslän; ^5^Department of Obstetrics and Gynaecology, University of Gothenburg, Salhgrenska Academy, Gothenburg, Sweden *Presenting author

***Background and aims***: Few studies have investigated vitamin D status in pregnant women in Sweden, but data indicate that poor vitamin D status might be common. The aim was to assess vitamin D status and its determinants in a population representative cohort of pregnant women in Sweden.

***Methods***: The study included 2,000 pregnant women in early pregnancy. Blood was drawn in gestational week <17 (T1) and in gestational week >31 (T3), and 25-hydroxyvitamin D (25(OH)D) was analyzed by liquid chromatography tandem-mass spectrometry.

***Results***: Mean (SD) 25(OH)D concentration was 64.5(24.5) nmol/L at T1 and 7% had concentrations <25 nmol/L. Among women born in Africa and Asia, 43 and 37% had concentrations <25 nmol/L, respectively. Factors relating to odds of 25(OH)D concentrations <25 nmol/L in T1 were origin, no vitamin D supplementation, covered clothing style, lower milk intake, and lower age.

Mean 25(OH)D concentration in T3 was 74.6(34.4) nmol/L. Determinants of change in 25(OH)D during pregnancy were origin, season at T3, preferring sun over shade, clothing style, margarine intake at T3, vitamin D supplementation at T3 and having travelled <40°N in the past 6 months. Among women born in Africa and Asia (*n*=316), the effect of season appeared to be smaller than in the whole cohort.

***Conclusion***: Vitamin D deficiency was uncommon among pregnant women in Sweden. However, among women born in Africa and Asia, 25(OH)D concentrations <25 nmol/L in early pregnancy were frequently observed. Factors significantly predicting vitamin D status were vitamin D intake, origin, age, and sun exposure.

**Disclosure of interest**: None to declare.

### Poster presentation no. P418

#### Dietary inflammatory index and the risk of a first myocardial infarction in Northern Sweden

##### Stina Bodén^1^*, Maria Wennberg^2^, Jonas Andersson^3^, Bernt Lindahl^4^, Nitin Shivappa^5,6^, James R. Hébert^5,6^ and Lena M. Nilsson^7^
^1^Radiation Sciences, Oncology, Umeå University, Umeå, Sweden; ^2^Public Health and Clinical Medicine, Nutritional Research, Umeå University, Umeå; ^3^Public Health and Clinical Medicine, Research Unit Skellefteå, Umeå University, Skellefteå, Sweden; ^4^Public Health and Clinical Medicine, Occupational and Environmental Medicine, Umeå University, Umeå, Sweden; ^5^Cancer Prevention and Control Program, Umeå, Sweden; ^6^Epidemiology and Biostatistics, Arnold School of Public Health, University of South Carolina, Columbia, United States; ^7^Public Health and Clinical Medicine, Nutritional Research, Arcum, Umeå University, Umeå, Sweden *Presenting author

***Background and aims***: The relation between low-grade inflammation, potentially derived from a persons’ diet, and cardiovascular disease (CVD) has been investigated using the dietary inflammatory index (DII), and significant associations have been reported. We aimed to examine the association between the DII and the risk of a first myocardial infarction (MI) in a Swedish population since this had not been performed previously.

***Methods***: We conducted a prospective case-control study with 296 cases and 605 controls derived from the large population-based Northern Sweden Health and Disease Study (NSHDS). DII scores were derived from a validated food frequency questionnaire (FFQ) administered in 1991–1999. Conditional logistic regression models, matched on date of health survey, age, and sex, were used to estimate crude and multivariable odds ratio (OR) and 95% confidence interval (CI) using quartile 1 (most anti-inflammatory diet) as referent. The multivariable model included smoking, ApoB/ApoA1, systolic blood pressure, history of diabetes, and education. Energy adjustment of DII was performed using the residual method.

***Results***: (Preliminary). OR of a first MI was higher for participants with higher DII scores (Q2-4) compared to Q1. For Q2–4 ORs were similar to one another; thus no linear trend across the quartiles was seen. Participants in Q2–4 had a 59 percent higher risk of MI relative to Q1 [crude OR (95% CI) for Q2-4: 1.59 (1.08-2.33)]. After multivariable adjustments, OR for Q2–4 relative to Q1 was 1.56 (0.99-2.47). (Note: In June 2016 results from a larger data set will be available).

***Conclusion***: In this study, an anti-inflammatory diet was associated with a lower risk of a first myocardial infarction.

**Disclosure of interest**: None to declare.

### Poster presentation no. P419

#### A systematic review of dietary aspects associated with cancer-related fatigue

##### Hege S. Bekken*, Sveinung Berntsen and Elling Bere Department of Public Health, Sport and Nutrition, University of Agder, Kristiansand, Norway *Presenting author

***Background and aims***: As much as 70–100% of cancer patients experience cancer-related fatigue (CRF). In about 10–30%, CRF consists also after recovery from cancer, giving social and economic implications for both patients and society. Lifestyle factors are found to have an impact on both cancer and CRF. Many cancer patients do not follow dietary recommendations. At present, we do not know whether subjects with CRF should eat according to general recommendations or not. Thus, the aim of the present review was to systematize dietary aspects associated with CRF.

***Methods***: The present review was conducted in accordance with the PRISMA guidelines. Search phrases “chronic fatigue,” “fatigue syndrome,” “cancer survivor,” “nutrition,” “diet,” “food,” “supplement,” “cancer,” “neoplasm,” and “cancer survivor” were applied in a search through databases Cinahl, Medline, Embase, and Scopus in October 2015. Included studies had a quantitative design. Both curative and palliative cancer patients, in addition to cancer survivors, were included. Fatigue scales or quality of life scales including a measure on fatigue were the measured outcome.

***Results***: The search revealed 127 studies, 35 were reviewed full text and 26 met inclusion criteria (*n*_patients_=3,781). Designs of included studies were cross-sectional studies (*n*=10), cohort studies (*n*=5), randomized controlled trials (*n*=6), and intervention studies without control (*n*=5). Higher intake of levocarnitine, selenium, vitamin D, fruit, vegetables, fiber, carbohydrates, energy intake, protein, and dietary supplements was associated with less fatigue. Lower intake of glutamine, protein, and total energy intake, and a higher E% from dietary fat were associated with increased fatigue.

***Conclusion***: Several dietary aspects were reported in relation to CRF. However, present literature is limited in both amount and design, and more studies are needed to state a relationship between diet and CRF.

**Disclosure of interest**: None to declare.

### Poster presentation no. P420

#### Can a lifestyle intervention for pregnant women with obesity have positive effects on weight gain during pregnancy?

##### Åsa Premberg^1^, Ragnar Hanas^2^, Marie Berg^3^ and Karin Haby^4^* ^1^Sahlgrenska Academy, University of Gothenburg, Institute of Health and Care Sciences, Gothenburg, Sweden; ^2^Sahlgrenska Academy, University of Gothenburg, Institute of Clinical Sciences, Gothenburg, Sweden; ^3^Sahlgrenska Academy, University of Gothenburg, Centre for Person-centred Care, Gothenburg, Sweden; ^4^Mödrahälsovård (Mother Health Care), Primärvården (Primary Health Care) Västra Götaland, Gothenburg, Sweden *Presenting author

***Background and aims***: Maternal obesity is increasing and 13% of women assigned to antenatal care (AC) in Sweden have BMI≥30. The risk of complications during pregnancy and delivery, and for the child, increases with increasing BMI and is aggravated by high gestational weight gain (GWG). It is crucial to reduce the burden of adverse maternal and foetal outcomes by minimizing GWG. In this study – Mighty Mums (MM) – a coordinated project with standardized care, given by midwives and supported by dietician and aiming at reducing GWG in obese pregnant women, is evaluated.

***Methods***: All study participants (*n*=1165) received standard AC, and the intervention group (*n*=465) additionally received two extra sessions with the midwife and optional offer of activities toward a more healthy life style: food advice, prescription of physical activity, meetings with a dietician, active guidance to local health centers, pedometers, walking poles. A log was used throughout pregnancy to register weight, activity, food, thoughts, and feelings. The control groups consisted of women (*n*=104) getting standard AC followed prospectively, and women (*n*=700) from adjacent geographical areas followed retrospectively using register data.

***Results***: A previously presented analysis of a pilot group of 50+50 women showed significant effect on GWG (8.6±4.9 kg vs. 12.5±5.1 kg) in the intervention group, and a significantly lower weight at the postnatal checkup versus the first contact with AC (−0.2±5.7 kg vs. +2.0±4.5 kg). A greater proportion of MM also managed to restrict their GWG to less than 7 kg (36% vs. 16%). The result from the full scale study will be presented and discussed at the conference.

***Conclusion***: Our pilot study showed that it is possible to guide the woman in AC towards lifestyle changes that decrease GWG, with a modest and economically realistic effort with simple measures, and we expect similar results from the full study.

**Disclosure of interest**: None to declare.

### Poster presentation no. P421

#### Nutrient intake differences between monozygotic twins discordant for BMI and liver fat

##### Leonie-Helen Bogl^1,2^*, Sari Räsänen^3^, Jaakko Kaprio^1,2^ and Kirsi Pietiläinen^2,3^
^1^Clinicum, Department of Public Health, University of Helsinki, Helsinki, Finland; ^2^Institute for Molecular Medicine Finland (FIMM), University of Helsinki, Helsinki, Finland; ^3^Obesity Research Unit, Research Programs Unit, University of Helsinki, Helsinki, Finland *Presenting author

***Background and aims***: To study whether macro- and micronutrient intakes differ between BMI-discordant monozygotic (MZ) co-twins concordant and discordant for liver fat independent of genetic effects.

***Methods***: The sample consisted of 23 MZ twin pairs discordant for BMI (BMI difference ≥ 3 kg/m^2^), and weight-concordant control pairs (*n*=22, BMI difference <3 kg/m^2^), identified from two nationwide cohorts of Finnish twins. Dietary intake was assessed by 3-day food records. Under-reporters were excluded from the analysis. Liver fat content was measured by proton magnetic resonance spectroscopy.

***Results***: In individuals, energy intake and energy contributing nutrients were higher in overweight as compared to lean subjects. Individuals with fatty liver (liver fat percentage >5.5%) had an increased omega-6 (n-6) to omega-3 (n-3) fatty acid (FA) ratio, and a lower intake of vitamin D and vitamin K. In pairwise analysis, leaner and heavier co-twins of discordant pairs did not differ significantly in macro- or micronutrient intake. When BMI-discordant pairs were further divided into liver fat-discordant and concordant (based on the median liver fat difference, 2.6%), the nutrient intake of heavier co-twins with liver fat differed from that of their leaner co-twins without liver fat, that is, a higher n-6 to n-3 FA ratio and lower vitamin D intake.

***Conclusion***: The balance of the polyunsaturated n-6 to n-3 FA ratio and vitamin D intake in the habitual diet differ between genetically identical twin pairs who differ in liver fat, suggesting that future studies should give attention to these nutrients when examining determinants of non-alcoholic fatty liver disease.

**Disclosure of interest**: None to declare.

### Poster presentation no. P422

#### Levels of iodine, sodium, and dietary fibre in selected Nordic and Estonian foods

##### Helena Pastell^1^*, Ann J[otilde]eleht^2^, Ellen Kielland^3^, Ólafur Reykdal^4^, Veronica Öhrvik^5^, Jorån Østerholt Dalane^3^, Liisa Valsta^6^ and Nordic Food Analysis Network ^1^Finnish Food Safety Authority Evira, Helsinki, Finland; ^2^National Institute for Health Development, Tallinn, Estonia; ^3^Norwegian Food Safety Authority, Oslo, Norway; ^4^Matís, Reykjavik, Iceland; ^5^National Food Agency, Uppsala, Sweden; ^6^National Institute for Health and Welfare, Helsinki, Finland *Presenting author

***Background and aims***: Iodine, sodium, and dietary fibre (DF) levels in food are momentous nutrients due to several aspects. Mild iodine deficiency occurs even in the Nordic countries and fortification practices differ. Quantification of salt in food is today based on sodium concentrations instead of chloride, and policies to lower salt intakes in the Nordic Countries vary. The definition of DF has been revised recently and new methods have been developed to meet the definition. The aim of this study was to determine the contents of iodine, sodium and DF in selected foods and compare the results between the countries participating The Nordic Food Analysis Network.

***Methods***: Pooled samples of whole milk, conventionally produced eggs, organic eggs, low-fat cheese, and farmed salmon (*Salmo salar*) for iodine and sodium analyses, as well as rye flour, wholegrain wheat flour and rolled oats for DF analyses, were collected from Estonia, Finland, Iceland, Norway, and Sweden. The sample collection was based on the supply and the marked shares of each country. Iodine was analyzed using ICP-MS, sodium by ICP-OES technique, and DF contents using the AOAC 2011.25 method.

***Results***: Iodine contents in Icelandic whole milk and conventionally produced eggs, as well as in Swedish organic eggs were two-fold compared to the respective foods in other countries. Some variation was also found in total DF contents of wholegrain wheat flour, where the differences were mainly dominated by the water-insoluble DF fraction. DF contents of the analyzed cereal products were higher than reported earlier in Food Composition Databases due to the new method used.

***Conclusion***: The contents of iodine and DF are not equal in all foods among Nordic countries and Estonia. Differences in animal feeding practices result true compositional differences in foods. Borrowing data from food composition databases from even neighboring countries needs to be done with caution.

**Disclosure of interest**: None to declare.

### Poster presentation no. P424

#### The “Hand Model” – a tool for obese children's individualized portion sizes

##### Hannah Helgegren* and Christel Larsson Department of Food and Nutrition and Sport Science, University of Gothenburg, Gothenburg, Sweden *Presenting author

***Background and aims***: The widely used “plate model” describes the proportions between the 3 main components of food on the plate, but says nothing about the absolute amount of food. Children in treatment at the pediatric obesity unit (POU) in Malmö often describe eating the same portion size as their parents, sometimes more. Portion size tools can be hard to use in daily life and often require the use of lists, kitchen scales, measuring cups, etc. The aim was to develop a simpler tool for individualized portion sizes and at the same time promote a varied diet for healthy growth and development.

***Methods***: During 2011 a POU dietician, together with children in treatment, developed “the Hand Model” (HM). Just like the plate model, the HM uses 3 sections on the plate but then states that each of its 3 main components (starch-rich foods, protein-rich foods, and vegetables) should have about the same volume as the child's fist. When teaching a family the HM the child is showing its fist on each part of the plate, then comparing it to the parent's fist, demonstrating that the child's portion should be smaller than the adults.

***Results***: HM has been used in clinical practice at the POU for almost 5 years, and also by some school nurses. In interviews with professionals using the HM it has been stated:

It's easy to teach. Parents say it's an understandable way to speak about portion size. (Psychologist)

You always have the main tools with you – your hands! (Pediatric nurse)

It shows that the child's portion needs to be smaller than the parent's. It makes sense and seems less unfair to many children. (Dietician)

The parents like that it's so concrete, they use it at home and often the children then try it themselves at the school lunch. (School nurse)

***Conclusion***: HM is perceived useful by different professionals. Further implementation and evaluations studies of the model is needed and will be explored as a part of a PhD project.

**Disclosure of interest**: None to declare.

### Poster presentation no. P425

#### Reported dietary and demographic factors in individuals with high fish consumption: are there gender differences?

##### Therese Karlsson^1^*, Hanne Rosendahl-Riise^1^, Jutta Dierkes^2^, Grethe S. Tell^3^ and Ottar Nygård^1,4^
^1^Department of Clinical Science, University of Bergen, Bergen, Norway; ^2^Department of Clinical Medicine, University of Bergen, Bergen, Norway; ^3^Department of Global Health and Primary Care, University of Bergen, Bergen, Norway; ^4^Department of Heart Disease, Haukeland University Hospital, Bergen, Norway *Presenting author

***Background and aims***: In some epidemiological studies, associations between intake of fish and health outcomes have been reported to differ by gender. Also, intake of fish often varies between men and women. Therefore, the aim was to explore gender differences in self-reported dietary and demographic factors in subjects with high fish consumption from Western Norway.

***Methods***: Subjects were men and women (46–49 y) who participated in the Hordaland Health Study (1997–99) who completed a food frequency questionnaire. High fish consumers, defined as intake of total fish ≥450 g/week (including fatty, lean, processed, and unspecified fish as part of meal and fish as spread), were included in the current analysis.

***Results***: A higher proportion of men were overweight, had higher educational level, and higher household income compared with women (*p*<0.001). Median (IQR) total fish intake was 694 (327) and 617 (258) g/week (*p*<0.001) in men and women, respectively. Intake of different types of fish (percent of total fish intake, men vs. women) was as follows: lean fish (36.7% vs. 38.9%, *p*<0.01), fatty fish (15.1% vs. 15.3%, *p*=0.73), processed fish consumption (21.3% vs. 19.8%, *p*<0.01), and fish as spread (10.8% vs. 7.0%, *p*<0.001).

***Conclusion***: In this cohort of middle-aged high fish consumers, there were gender differences in reported dietary and demographic factors, which may be important to consider as gender-specific confounders or modifying factors.

**Table 1:** Dietary intake

	Men *n*=778	Women *n*=754	*P*^a^
Vegetables, g/d	187 (152)	230 (183)	<0.001
Fruit and berries, g/d	222 (217)	246 (203)	<0.01
Vegetable, fruit and berries ≥500 g/d	39.2	49.9	<0.001
Fiber, g/1,000 kcal	10.5 (3.1)	12.3 (3.7)	<0.001
Alcohol consumption^b^			<0.001
Low	56.6	79.8	
Moderate	38.0	15.8	
High	5.4	4.4	

Values are presented as median (IQR) or proportion.

a Mann-Whitney U test or Fisher's exact test.

b low: <8 g/d; Moderate: men 8–28 g/d, women 8–16 g/d; High: men >28 g/d, women >16 g/d.

**Disclosure of interest**: None to declare.

### Poster presentation no. P426

#### How often is it acceptable for preschoolers to consume sugar-rich foods and drinks? Associations between parents’ views and education level

##### Riikka Kaukonen^1^*, Reetta Lehto^1^, Suvi Määttä^1^, Carola Ray^1^, Nina Sajaniemi^2^, Maijaliisa Erkkola^2^ and Eva Roos^1^
^1^Folkhälsan Research Center, Helsinki, Finland; ^2^Department of Food and Environmental Sciences, University of Helsinki, Helsinki, Finland *Presenting author

***Background and aims***: Finnish 3–6-year-old children's sugar intake is above the recommended limit of 10% of energy intake. Parent-related factors shape the food intake patterns of children. The aim was to investigate parents’ views on acceptable intake frequency of sugar-rich foods and drinks in children and their association with parental education level.

***Methods***: A cross-sectional study was conducted in Finland in autumn 2015. One parent (88% mothers) of 469 three- to six-year-old children reported their highest education level and completed a survey including questions about their views on acceptable intake frequency of common sugar-rich foods and drinks. Distributions were used to describe parents’ views, and chi-square statistic to test their associations with parental education level grouped into three categories (low, intermediate, and high).

***Results***: The majority (>70%) of the parents thought it is acceptable to consume sugared soft drinks, sugared juices, ice cream, sweet pastries, sweet cookies, and sweets and chocolates once a week or less. Sugared cereals and muesli, sugared yoghurts, 100% fruit juices, and cocoa were seen more as “everyday foods” and many (≥40%) of the parents thought it is acceptable to consume these foods two or more times a week. Smaller proportion of parents with highest education level vs. lowest education level reported that it is acceptable to consume sugared cereals and muesli (35% vs. 50%), and cocoa (31% vs. 47%) two or more times a week and sweets and chocolate (75% vs. 89%) sugared soft drinks (14% vs. 33%) and sugared juices (46% vs. 61%) on a weekly basis.

***Conclusion***: Differences in parents’ views on acceptable intake frequency exist between lower and higher educated parents. The relevance of parents’ views in relation to actual food intake should be further studied.

**Disclosure of interest**: None to declare.

### Poster presentation no. P427

#### Beverage consumption pattern among Norwegian adults

##### Mari M. Paulsen*, Janniche B. Myhre and Lene F. Andersen Department of nutrition, University of Oslo, Oslo, Norway *Presenting author

***Background and aims***: Beverage intake may be important contributors for energy intake and overall dietary quality. Despite dietary recommendations and knowledge regarding health effects of different types of beverages, there is scarce knowledge about to which meals and time of the week different beverages are consumed. Our aim was to investigate how beverage consumption varies between different meals and between weekdays and weekend-days in Norwegian adults.

***Methods***: A cross-sectional dietary survey was conducted among 1,787 Norwegian adults in 2010–2011. Two non-consecutive telephone-administered 24 h recalls were used for dietary data collection. The recorded meal types were breakfast, lunch, dinner, supper/evening meal, and snacks.

***Results***: Snacks were the meal type contributing most to intakes of water, coffee, tea (women only), artificial sweetened beverages, and beer. Dinner contributed most to intake of sugar-sweetened beverages and wine, whereas milk and juice were mostly consumed for breakfast. Consumption of sugar-sweetened beverages and artificial sweetened beverages did not differ between weekdays and weekend-days among consumers. The average intake of wine and beer (men only) among consumers was higher on weekend-days, than on weekdays.

***Conclusion***: Beverage consumption pattern in the Norwegian adult population vary between different meal types. Alcohol consumption seems to be higher during weekend-days, compared to weekdays among consumers.

**Disclosure of interest**: None to declare.

### Poster presentation no. P428

#### Weight change and risk of Parkinson's disease – a cohort study

##### Katri Sääksjärvi^1^*, Paul Knekt^1^, Satu Männistö^2^ and Jukka Lyytinen^3^
^1^Department of Health, Health Monitoring Unit, National Institute for Health and Welfare, Helsinki, Finland; ^2^Chronic Disease Prevention Unit, Department of Health, National Institute for Health and Welfare, Helsinki, Finland; ^3^Department of Neurology, Helsinki University Central Hospital, Helsinki, Finland *Presenting author

***Background and aims***: Obesity has been associated with a greater Parkinson's disease (PD) risk, but previous studies have been inconsistent. Only few studies have examined the prediction of weight change on PD risk.

***Methods***: This cohort study includes subjects from the Finnish Mobile Clinic Health Examination Survey conducted in 1973–1976 (*n*=6,314, aged 50–79 years, free from PD at the baseline). Height and weight were measured at the baseline, but also 4–7 years earlier in previous health examination, and body mass index (BMI) was calculated for both time points. A questionnaire provided information on several background factors. During a 22-year follow-up from the baseline (1973–1976), 95 incident PD cases occurred. The Cox's model estimated the strength of association between change in BMI and PD risk, adjusting for age, sex, education, community density, smoking, alcohol, and coffee consumption, and leisure-time physical activity.

***Results***: There was no association between change in BMI and PD risk in total population. However, among those who had BMI ≥25 at the first measurement, weight gaining was associated with increased PD risk (Table 1). Among those who had BMI <25 at the first measurement, moderate weight gaining associated with decreased PD risk.

***Conclusion***: Obesity might be a risk factor for PD. Alternatively change in weight may reflect metabolic pathology in persons at high risk of PD.

**Abstracts P428– Table 1:** Relative risk of PD according to change in BMI, modified by first BMI status

	BMI ≥25 at the first measurement			BMI <25 at the first measurement		
		
Delta BMI	PD cases/*n*	RR	95% CI	PD cases/*n*	RR	95% CI
No change, delta BMI > −0.50 and <0.50	8/918	1.00	–	13/565	1.00	–
Gained weight moderately, delta BMI ≥0.50 and <2	18/847	2.47	1.07–5.70	4/612	0.24	0.08–0.73
Gained weight abundantly, delta BMI ≥2	11/458	3.27	1.31–8.16	4/342	0.46	0.15–1.42
Lost weight moderately, delta BMI ≤−0.50 and > −2	20/1,017	2.34	1.03–5.31	5/537	0.44	0.16–1.25
Lost weight abundantly, delta BMI ≤ −2	7/720	1.35	0.49–3.75	1/143	0.34	0.04–2.60

**Disclosure of interest**: None to declare.

### Poster presentation no. P429

#### Monitoring marketing of food and beverages to children in Norwegian grocery stores – the “Sweep” method

##### Helene Astrup*, Lisa B. Hansen, Ida S. Kaasa, Marte Ekeberg-Sande, Mari M. Paulsen, Knut-Inge Klepp and Lene F. Andersen Department of nutrition, University of Oslo, Oslo, Norway *Presenting author

***Background and aims***: As a follow-up to WHO's guidelines to reduce marketing of unhealthy food and beverages to children (2010), The Food and Drink Industry Professional Practices Committee (MFU) in Norway was established in 2013. The MFU introduced “Guidelines for marketing of food and beverages to children.” According to these guidelines, the product itself, including packaging or product placement in stores, is not considered as marketing. In the present study we mapped marketing in terms of the product itself, including packaging and strategic location in stores distributed all over Norway.

***Methods***: A sweep protocol was developed based on a draft protocol from WHO Euro Action Network to reduce marketing of unhealthy foods and drinks to children. The sweep monitored products marketed for children in 12 food categories. The following were assessed on identified products; product placement in shelf, marketing on packaging and materials like cartoons, pictures of children, celebrities, contests, free gifts, etc. All products were photographed.

***Results***: A total of 103 grocery stores from 50 municipalities were included in the study. Preliminary results showed marketing on products in all 12 categories. Most marketing was found on products in the categories cereals, milk and yoghurt, salty snacks, sweetened beverages, and chocolate and candy. Chocolate and candy were the categories with most extensive marketing on materials in stores. Less marketing was found in the categories vegetables, fruit and berries, and pizza.

***Conclusion***: Guidelines to reduce marketing of unhealthy foods and beverages to children exist, but the product itself is excluded from these guidelines. Several companies seem to exploit this loophole. Our results will demonstrate the scope of this in Norway.

**Disclosure of interest**: None to declare.

### Poster presentation no. P430

#### New Swedish dietary guidelines: good for health and environment. Find your way to eat greener, not too much and be active

##### Åsa B. Konde*, Anette Jansson, Rickard Bjerselius and Jorun S. Färnstrand Science Division, Swedish National Food Agency, Uppsala, Sweden *Presenting author

***Background and aims***: The Swedish National Food Agency has updated the national dietary guidelines. The updated advice is about how to eat healthily and at the same time take into account environmental aspects.

***Methods***: The recommendations regarding healthy eating are based on the Nordic nutritional recommendations (NNR 2012), knowledge of the population's dietary habits, and knowledge of the environmental impact of various food groups.

The development of the guidelines has been done in collaboration with many different stakeholders through a reference group, an open hearing, and an open consultation on the web. Focus groups of consumers were used to develop target group-adjusted material.

***Results***: For the sake of both health and environment, food consumption needs to shift from a large amount of foods of animal origin, to more plant-based foods. Food patterns based on vegetables, whole grains, lean dairy products, fish, and oil decrease the risk of the common population diseases in Sweden – cardiovascular disease, overweight/obesity, type 2 diabetes, and certain types of cancer. This has been focused on in previous dietary guidelines, and has been further strengthened by current research.

As a whole, the recommendations include ten food groups and advice regarding energy balance and physical activity.

***Conclusion***: A switch to a large proportion of plant-based foods is a good choice both for health reasons and for the environment. To integrate health and environment in the work regarding nutritional advice is a new and important step for a sustainable future food consumption.

**Disclosure of interest**: None to declare.

### Poster presentation no. P431

#### Dietary adequacy of lunch meals served and consumed at Danish daycare centers

##### Ellen H. Tørsleff, Ellen Trolle*, Inge Tetens and Anne D. Lassen National Food Institute, Technical University of Denmark, Copenhagen, Denmark *Presenting author

***Background and aims***: The official Danish dietary recommendations for daycare food are under revision due to updated Nordic Nutrient Recommendations 2012, making a comparison of the food consumption in daycare centers with the suggested recommendations required.

The aims of the study were: 1) to compare children's lunch meal intake at Danish daycare centers with the suggested dietary recommendations and 2) to examine the relation between the nutritional content of the served and consumed lunch.

***Methods***: Data were collected from eight daycare centers in rural areas of Denmark, serving in-house prepared lunch. The food served for lunch, and the food waste were weighed at group level for 5 successive days (*n*=40 lunch meals).

The nutritional composition of served and consumed meals was calculated as a mean per child, using GIES (General Intake Estimation System). Ratios between food consumed and served were calculated.

***Results***: The children's mean (SD) age was 4.8 years (0.9), and the mean group size was 26 (11). The children consumed on average 1,346 kJ (365), which was close to the recommended average of 1,350 kJ. However, the energy content varied considerably from 784 to 2,192 kJ. The percent energy from fat (E%) was on average 32 (8) and therefore within the recommended range of 32–34 E%, but the energy from saturated fat was above the recommended <10 E% (11 E% (6)). The macronutrient distribution of the consumed food was well predicted by the served food; the mean ratios were 0.92–1.04, but the ratio of the energy consumed was 0.73.

***Conclusion***: The children's mean intake of energy and macronutrients generally complied well with the recommendations, but the variation in energy content and the energy from saturated fat exceeded the recommendations. Moreover the results indicated that the energy distribution of the served food could be an indicator of consumption, but precaution had to be taken when looking at the energy content.

**Disclosure of interest**: None to declare.

### Poster presentation no. P432

#### The Healthy and Sustainable Dietary and Physical Activity habits score and socio-demographic correlates

##### Helga B. Bjørnarå*, Monica K. Torstveit, Tonje H. Stea, Nina C. Øverby and Elling Bere Department of Public Health, Sport and Nutrition, University of Agder, Kristiansand, Norway *Presenting author

***Background and aims***: Environmental sustainability and public health are connected through diets and physical activity. Enhanced understanding regarding correlates of dietary and physical activity habits is relevant to allow for tailoring of interventions to relevant groups, which could increase adherence to the selected aspects at the population level. Currently, little is known about correlates of a combined approach. Thus, we aimed to: (I) create a combined Healthy and Sustainable Dietary and Physical Activity habits (HSDPA) score and (II) assess potential socio-demographic correlates of the HSDPA score.

***Methods***: Cross-sectional data were obtained from 530 parents of toddlers participating in the healthy and sustainable lifestyle (HSL) project (2014–2015). Multilevel linear mixed models explored associations between potential correlates and selected dietary and physical activity habits, both separately and collapsed into the HSDPA score (possible range: 0–40).

***Results***: The HSDPA score incorporated the following aspects: (I) New Nordic Diet, (II) Local and sustainable foods, (III) Active transportation, and (IV) Non-exercise outdoor activities. For the fully adjusted models, mean scoring on the HSDPA score in total was higher for participants with high education (mean (95% CI): 18.2 (17.4–19.0)), than for those with low education (16.8 (15.8–17.7), *p*=0.002), and for participants living centrally (18.4 (17.6–19.2)), compared to those living less centrally: 16.5 (15.6–17.4), *p* ≤ 0.001). No differences were found for sex, ethnicity, or age.

***Conclusion***: Higher education and centrality singled out as the most relevant correlates of selected dietary and physical activity habits. Our findings indicate that interventions should be tailored to low educated groups and to those living in non-central areas, in order to facilitate lifestyle habits potentially promoting public health and environmental sustainability.

**Disclosure of interest**: None to declare.

### Poster presentation no. P433

#### Adherence to the New Nordic Diet during pregnancy and subsequent risk of overweight and obesity in the Norwegian Mother and Child Cohort Study

##### Marianne Skreden^1^, Neha Agnihotri^1^*, Elling Bere^1^, Elisabet R. Hillesund^1^, Margaretha Haugen^2^ and Nina C. Øverby^1^
^1^Department of Public Health, Sport and Nutrition, University of Agder, Kristiansand, Norway; ^2^Division of Environmental Medicine, Norwegian Institute of Public Health, Oslo, Norway *Presenting author

***Background and aims***: Overweight and obesity among women of reproductive age are major public health threats worldwide. This study aimed to investigate associations between maternal adherence to the New Nordic Diet (NND), a potentially healthy and sustainable diet including whole grains, potatoes, milk, local fruits, root vegetables, cabbages, and foods from the wilderness (fish, berries, and game), during pregnancy and risk of overweight and obesity 8 years post-delivery.

***Methods***: The study was carried out in the Norwegian Mother and Child Cohort Study (MoBa). A total of 17,337 women were included in the analyses Adherence to NND was assessed by a previously developed score (0–10) and trichotomized into low (0–3 points), medium (4–5 points), and high (6–10 points) NND adherence. Binary logistic regression was used to examine the association between NND category and risk of overweight and obesity 8 years post-delivery. Covariates included in the model were maternal age, parity, pre-pregnant BMI, pre-pregnant smoking, maternal educational level, pre-pregnant physical activity, and maternal energy intake. Odds ratios (OR) with 95% confidence interval (CI) were calculated for each NND group with low NND adherence as the reference group.

***Results***: High NND adherence was associated with lower odds of being overweight (OR 0.83; 95% CI 0.72–0.95) and obese (OR 0.69; 95% CI 0.57–0.84) 8 years post-delivery. High NND adherence in women with normal pre-pregnant BMI was associated with lower odds of being overweight 8 years post-delivery (OR 0.78; 95% CI 0.66–0.92). Similarly, high NND adherence in women classified with overweight pre-pregnancy was associated with lower odds of being obese (OR 0.76; 95% CI 0.59–0.98) 8 years post-delivery as compared to low NND adherence.

***Conclusion***: High adherence to NND in pregnancy is associated with long-term lower risk of obesity and overweight.

**Disclosure of interest**: None to declare.

### Poster presentation no. P434

#### Concept and strategies to integrate food in educational activities in preschool. A meal education project in a Swedish municipality

##### Hanna Sepp* Food and Meal Science, School of Education and Environment, Kristianstad University, Kristianstad, Sweden *Presenting author

***Background and aims***: Children's education concerning a healthy lifestyle is one of the most important keys to good health. Thus the preschool has an important task to health promotion and prevention by introducing many different foods, particularly fruit and vegetables, to children in a pleasurable and educational manner. In a recently performed Swedish study food as a tool for learning in everyday activities were explored in preschool. The result shows that support, both individual and structural, is needed in order to make food meaningful among educational activities in preschool. The lack of experienced individual and/or structural support makes it hard to integrate food as a natural part of planned educational activities. This study has as its starting point from this study.

***Methods***: Based on these results, the aims of the teachers and kitchen staffs’ trainings were defined, and an overall concept was deduced. Regarding the concept for the training sessions, it was concluded that the training modules should focus on presenting information on the practical implementation. Furthermore, these modules also include self-efficacy enhancing components and give preschool staff opportunities to share experiences. Regarding the didactic methods applied in the training sessions, constructivist learning approaches that facilitate active participation, reflective thinking, and personal involvement were implemented. Emphasis was put on the didactic methods and how to integrate food in educational activities.

**Disclosure of interest**: None to declare.

### Poster presentation no. P435

#### Iron status in adults and children in Sweden in relation to dietary iron

##### Eva W. Lemming^1^*, Wulf Becker^1^, Cecilia Nälsén^1^, Peter Ridefelt^2^, Veronica Öhrvik^1^, Natalia Kotova^1^ and Anna-Karin Lindroos^1^
^1^Risk and Benefit Assessment, National Food Agency, Uppsala, Sweden; ^2^Medical Sciences, Clinical Chemistry, Uppsala University, Uppsala, Sweden *Presenting author

***Background and aims***: Iron deficiency (ID) is the most common and widespread nutritional disorder in the world in both industrialized and non-industrialized countries, and young women is especially at risk. Iron is primarily stored in the body as ferritin, and there is a direct and positive reflection between total ferritin stores and plasma ferritin. This study aimed to investigate iron status in a national sample of adults (between 18 and 80 years) and children (aged 11–12 years) in Sweden.

***Methods***: Two hundred-eighty women and men and 211 boys and girls were recruited in two different cross sectional surveys conducted by the National Food Agency. Diet was collected in a web-based self-assisted 4-day food record, the Riksmaten-method. Blood samples were collected, and plasma ferritin was measured in a chemiluminescant microparticle immunoassay.

***Results***: There was a significant difference in plasma ferritin levels between the sexes in both adults and children, however dietary iron (gram/10 MJ) was only different in adults. Plasma ferritin <5 µg/L is defined as an indication of depleted iron stores, and this was established in 22 women (15%) and 24 children (11%). Low ferritin levels were more common in younger women. Further, plasma ferritin levels and dietary iron were positively correlated in women (*r*=0.16, *p*<0.05) and girls (*r*=0.24, *p*<0.05).

***Conclusion***: Prevalence of iron-depleted stores in adults accord with international data in industrialized countries. In children, the prevalence of ID was low but still worrying considering that this population is only 11–12 years old and most of them have not reached puberty.

**Table:** 

	Women *n*=150	Men *n*=130	Girls *n=*97	Boys *n*=114
Plasma ferritin (µg/L)	68.8±64*	182±137	20.2±13.7*	24.9±15.4
Dietary iron (g/day)	10.2±3.7*	12.0±3.9	7.8±2.4	8.0±2.6
Dietary iron (gram/10 MJ)	13.3±3.5	12.6±3.3	11.0±2.3	11.2±2.5
Energy MJ/day	7.8±2.0*	9.7±2.5	7.1±2.0	7.2±1.9

* *p*<0.05.

**Disclosure of interest**: None to declare.

### Poster presentation no. P436

#### Associations between Swedish mothers’ and 3- and 5-year-old children's food intake

##### Lena Hansson^1^*, Berit L. Heitmann^2^, Christel Larsson^3^, Per Tynelius^1^, Mikaela Willmer^1^ and Finn Rasmussen^1^
^1^Department of Public Health Sciences, Karolinska Institutet, Stockholm, Sweden; ^2^Research Unit for Dietary Studies, Institute of Preventive Medicine, Copenhagen, Denmark; ^3^Department of Food and Nutrition, and Sport Science, University of Gothenburg, Gothenburg, Sweden *Presenting author

***Background and aims***: Parents’, and especially mothers’, eating habits are presumed to influence young children's emerging eating pattern. However, very few studies have been based on food diaries designed to ease comparability between preschool children and their mothers. This study examined the association between mothers’ and children's food intake, and whether these associations were moderated by child age and place of residence.

***Methods***: Children and mothers were randomly selected from the Register of the Total Population (RTP) in 2008. Families came from eight counties in mid-Sweden. Background variables were collected through self-reports and from the RTP. Mothers’ recorded their total food intake in a food diary during two 4-day periods. Children's food intake was recorded by their mothers using the same method. The study investigated 16 food items or groups. Data from 189 mother–child pairs were analyzed by Spearman rank-order correlation and multiple regression analysis.

***Results***: The strongest correlations between mothers’ and children's food intake were found for pizza (*r=*0.80) and fatty fish (*r=*0.70). The weakest correlations were found for sugared drinks (*r*=0.26) and fruits and berries (*r*=0.24). Children's age moderated the relationship between mother's and children's intake of savory snacks. The association between mother's and children's intake of pizza was stronger for those living in urban than rural areas.

***Conclusion***: Young children's food intakes were correlated with their mother's food intake, however, the strength of the association varied depending on food type investigated. Thus, parental modeling may only play a role in influencing young children's intake for certain food types.

**Disclosure of interest**: None to declare.

### Poster presentation no. P437

#### Non-digestible polysaccharides attenuate mast cell induced hyperpermeability in colonic biopsies

##### John-Peter G. Mall^1^*, Liza Löfvendahl^1^, Åsa Keita^2^, Robert Brummer^1^ and Ida Schoultz^1^
^1^School of Medical Sciences, Nutrition-Gut-Brain Interactions Research Centre, Örebro University, Örebro, Sweden; ^2^Department of Clinical and Experimental Medicine, Division of Clinical Sciences, Linköping University, Linköping, Sweden *Presenting author

***Background and aims***: Nearly 50% of the aging population suffers from stomach problems such as diarrhea and constipation. Our aim was to investigate whether specific non-digestible polysaccharides (NPS) can counteract mast cell-induced permeability increase of colonic biopsies mounted in Ussing chamber.

***Methods***: The effect of NPS on intestinal permeability was examined in colonic biopsies from 14 healthy subjects and 13 elderly subjects with diarrhea and/or constipation. Twelve colonic biopsies per subject were mounted in the Ussing chambers. The biopsies were pre-treated with either 0.5 mg/ml NPS derived from yeast or 0.1 mg/ml NPS derived from wheat, for 20 min before the mast cell degranulating chemical Compound (C) 48/80 (10 ng/ml) was added. Unstimulated biopsies were used as control. FITC-dextran 4000 and horseradish peroxidase (HRP) were used to measure paracellular and transcellular permeability, respectively. Non-parametric statistical analysis was applied.

***Results***: Intervention with C48/80 resulted in 2–3 times higher permeability compared to control biopsies. Treatment with 0.1 mg/ml NPS from wheat together with C48/80 resulted in a significant reduction of C48/80's effect, both on paracellular (*p*<0.05) and transcellular (*p*<0.05) permeability in younger participants (*n*=14). NPS from yeast (0.5 mg/ml) had no effect on young participants but instead showed a significant (*p*<0.05) reduction of C48/80-induced increase in transcellular permeability in elderly (*n*=13).

***Conclusion***: Our results show that the NPS from wheat (0.1 mg/ml) reduced C48/80-induced permeability increase in healthy subjects. A significant decrease in transcellular permeability was observed from the yeast-derived NPS but only in elderly with gastro-intestinal complaints. These results suggest that NPS can counteract C48/80-induced permeability in colon of different populations.

**Disclosure of interest**: None to declare.

### Poster presentation no. P438

#### Food appearances in children's television programs in Iceland and Sweden

##### Steingerdur Olafsdottir^1^ and Christina Berg^2^* ^1^Faculty of Sport, Leisure Studies and Social Education, University of Iceland, Reykjavik, Iceland; ^2^Department of Food and Nutrition and Sport Science, University of Gothenburg, Gothenburg, Sweden *Presenting author

***Background and aims***: Television viewing has been proposed to contribute to increased energy intake. Studying other television content than advertisements is necessary to better understand the relation between children's TV viewing and dietary habits. The aim of the study is to examine the nature and extent of verbal and visual appearance of food and beverages in children's programs in Swedish and Icelandic public service television.

***Methods***: The study objects are popular TV programs (domestic and international) in Swedish and Icelandic television, watched by children up to at least 10 years of age. The analyzed material will consist of approximately 50 h total, broadcast during wintertime (the most popular TV viewing months) in the two countries. All appearances and type of food and beverages are coded as well as the context in which the foods are discussed or appeared.

***Results***: Data collection is in the final phase in Iceland but a paper on the study from Sweden is in press (International Journal of Consumer Studies). Among the Swedish results are that high-calorie and low-nutrient foods (HCLN) constituted 19% of all food appearances in the programs in Swedish television, and fruits and vegetables constituted 39%. More than half of the HCLN foods appearances were with children characters, while only one third of the fruits and vegetables were shown with children.

***Conclusion***: HCLN foods seem to be represented as more attractive than other foods in the programs aired in Sweden, by to a greater extent appearing with children and being consumed or actively handled. The material from Icelandic television is still being coded and analyzed. The comparison of the results from the two countries will be of interest and might indicate the potential to improve the way food and eating are depicted on children's television.

**Disclosure of interest**: None to declare.

### Poster presentation no. P439

#### Motivational interviewing to promote healthy food habits in 4-year olds and their mothers. The PRIMROSE trial

##### Lena Hansson* on behalf of PRIMROSE Department of Public Health Sciences, Karolinska Institutet, Stockholm, Sweden *Presenting author

***Background and aims***: The objective was to evaluate a manualized theory-driven primary preventive intervention aimed at early childhood obesity. The intervention was embedded in Swedish child health services, starting when eligible children were 9–10 months of age, and continuing until the children reached the age of 4.

***Methods***: Child health care centers in eight counties were randomized into intervention and control and included 1,355 families. The intervention group participated in one group session and eight individual sessions with a nurse trained in motivational interviewing, focusing on healthy food habits and physical activity, besides care as usual. The control group received only care as usual. Outcomes were children's and mothers’ eating habits measured by a validated FFQ. The effectiveness of the intervention was assessed in linear and log-binominal regression models using generalized estimating equations and intention-to-treat analysis.

***Results***: There were differences between the intervention and the control groups in the expected direction. However, not all comparisons were statistically significant. Mothers reported a higher consumption frequency of vegetables (MD 0.13 times/day), and a lower consumption frequency of sugared drinks (MD 0.49 t/week), French fries (MD 0.37 t/month), and discretionary calories (MD 0.6 t/w) for the children in the intervention group than in the control group. For mothers, a statistically lower consumption frequency of French fries and discretionary calories was reported in the intervention group than in the control group.

***Conclusion***: There was suggestive evidence for a higher and lower intake of healthy respectively unhealthy foods among children and their mothers in the intervention group than in the control group. However, these small differences might be due to reporting bias.

**Disclosure of interest**: None to declare.

### Poster presentation no. P440

#### Gender-specific associations between eating patterns and sedentary activities in Norwegian 12–13 year old schoolchildren

##### Inger M. Oellingrath^1^* and Martin V. Svendsen^2^
^1^Department of Health Studies, University College of Southeast Norway, Porsgrunn, Norway; ^2^Department of Occupational and Environmental Medicine, Telemark Hospital, Skien, Norway *Presenting author

***Background and aims***: Several sedentary activities (SA) have been related with eating patterns (EPs) in adolescents. The aim of this study was to investigate the gender-specific associations between SA and EPs in a sample of 12–13-year-old Norwegian children.

***Methods***: Respondents were recruited from primary schools in Telemark County in 2010. A parental-reported questionnaire with information on children's dietary habits (retrospective FFQ) and daily time spent on SA outside school was used. Principal component analysis was used to identify EPs. The factor scores were grouped into tertiles. We used multiple logistic regression to calculate adjusted odds ratios (OR_a_) and 95% confidence intervals (CI) for being in the highest tertile of each EP (high adherence). All models were adjusted for physical activity (≥1 h/d MVPA), maternal education, family income, and child overweight (objectively measured).

***Results***: Complete data on diet and SA were obtained for 850 children (423 boys and 427 girls). Four EPs were identified and labeled: “snacking,” “junk,” “varied Norwegian,” and “dieting.” In boys, TV/DVD/video viewing ≥2 h/day was related to high adherence to the “snacking” EP (OR_a_=1.8, 95% CI: 1.1, 3.0) while non-screen activities such as reading, homework/studying ≥2 h/day was related to low adherence to this EP (OR_a_=0.44, 95% CI: 0.24, 0.83). In girls, PC use ≥2 h/day was associated with high adherence to the “dieting” EP (OR_a_=2.6, 95% CI: 1.5, 4.4), while non-screen SA ≥2 h/day was associated with high adherence to the “varied Norwegian” EP (OR_a_=1.7, 95% CI: 1.1, 2.7).

***Conclusion***: Gender-specific associations between time spent on various SA and children's EPs were observed. The findings indicate that measures aiming to improve children's diet should be aware of different food behaviors in boys and girls related to different types of SAs.

**Disclosure of interest**: None to declare.

### Poster presentation no. P441

#### Attitude and knowledge among Icelanders about the risk of high salt intake – data from a joint Nordic project

##### Elva Gisladottir* and Holmfridur Thorgeirsdottir Department of Health Determinants, Directorate of Health in Iceland, Reykjavik, Iceland *Presenting author

***Background and aims***: Salt consumption in Iceland is too high. Little was known about the attitude and knowledge of Icelanders on risk of high salt intake before the Nordic project started. The aim of the project was to increase knowledge about the risk of high salt intake with campaign in the media. It is important to make consumers aware of the risks associated with high salt consumption so they will choose products with less salt and thereby put a pressure on the industry to produce healthier products.

***Methods***: Two web-based surveys with 15 questions on attitude and knowledge regarding salt were conducted in the Nordic countries, in 2014 and in 2015 after the media campaign. Data here are presented for the Icelandic population only.

***Results***: In general, the results in 2015 did not change much from 2014. If there was a change it was in the direction of an increased knowledge regarding salt. Icelanders pay relatively little attention to their salt consumption. Almost a third think they themselves do not consume more salt than recommended, but 3 out of 4 think that the Icelandic population does on average consume more salt than recommended. A majority of Icelanders think that too much salt consumption can have a negative impact on health. Around 16% of Icelanders say they choose foods with the Nordic Keyhole when shopping, a 50% increase from 2014. 80% say it is likely or very likely they would try to reduce their salt intake if they find out that it is too high.

***Conclusion***: Icelanders consume too much salt, but they do not seem to know about it. Icelanders say they are susceptible to reduce their salt intake if finding out that they consume too much. There is a need to find ways to inform the public that the salt intake of Icelanders is too high.

**Disclosure of interest**: None to declare.

### Poster presentation no. P442

#### Sufficient maternal vitamin D status is not associated with gestational diabetes mellitus or birth size in normal-birth-weight infants

##### Helena Hauta-Alus^1,^*, Heli Viljakainen^1^ , Elisa Holmlund-Suila^1^ , Maria Enlund-Cerullo^1^, Jenni Rosendahl^1^, Saara Valkama^1^, Otto Helve^1^, Timo Hytinantti^1^, Outi Mäkitie^1,2,3^ and Sture Andersson^1^
^1^Children's Hospital, University of Helsinki and Helsinki University Hospital, Helsinki, Finland; ^2^Folkhälsan Research Center, Helsinki, Finland; ^3^Karolinska University Hospital, Center for Molecular Medicine, Karolinska Institutet and Clinical Genetics, Stockholm, Sweden *Presenting author

***Background and aims***: The effects of hypovitaminosis D during pregnancy have not been fully elucidated. Vitamin D deficiency has been associated with gestational diabetes mellitus (GDM). The aim was to compare pregnancy 25-hydroxy vitamin D concentration [25(OH)D] between mothers with and without GDM and to explore the impact of maternal factors on infant birth weight (BW) and ponderal index (PI).

***Methods***: Families were recruited after delivery at Kätilöopisto Maternity Hospital, Helsinki, Finland (*n*=747). Only mothers without medication were included. Infants were born at term, and BW was appropriate for gestational age. Birth measurements, gestational weight gain (GWG), and oral glucose tolerance test results (OGTT) were collected from medical records. Maternal 25(OH)D was determined from stored samples obtained in pregnancy. Background characteristics were collected retrospectively with a questionnaire. Uni- and multivariate regressions, and multivariate analysis of covariance were applied.

***Results***: GDM prevalence was 11%, and 99% of mothers were vitamin D sufficient [25(OH)D ≥50 nmol/l]. Maternal 25(OH)D did not differ between GDM-mothers and non-GDM-mothers (mean: 87 nmol/l vs. 89 nmol/l) in crude or adjusted model. Maternal 25(OH)D did not associate significantly with BW and PI in any models. BW and PI were higher among GDM-mothers than non-GDM-mothers (*p*=0.009; 0.027). Mothers’ prepregnancy BMI associated positively with BW and PI (*p*=0.013; 0.049). GWG associated positively with BW (*p*<0.001). Fasting baseline glucose at OGTT associated positively with BW and PI (*p*=0.032; 0.010), also in non-GDM-mothers (*p*=0.025).

***Conclusion***: Several maternal factors including GDM associated with birth weight and infant body composition measured as PI. However, at sufficient maternal 25(OH)D level vitamin D status did not associate with GDM or birth size measures in normal-birth-weight infants.

**Disclosure of interest**: None to declare.

### Poster presentation no. P443

#### Iron deficiency revisited: long-term effects of iron status in teenagers on cognitive performance, academic achievement, and wellbeing in adult life

##### Agneta Sjöberg^1,^*, Daniel Arvidsson^2^, Michael Hoppe^3^ and Lena Hulthén^4^
^1^Department of Food and Nutrition, and Sport Science, University of Gothenburg, Gothenburg, Sweden; ^2^Department of Translational Medicine, Lund University, Lund, Sweden; ^3^Gastroenterology and Hepatology Section, Clinical Nutrition Unit, Sahlgrenska University Hospital, Gothenburg, Sweden; ^4^Department of Internal Medicine and Clinical Nutrition, University of Gothenburg, Gothenburg, Sweden *Presenting author

***Background and aims***: It is not clear what impact iron deficiency during adolescence has on long-term brain function. The Gothenburg Adolescence Study (GAS) was started to evaluate the effects on iron status in vulnerable groups when the general iron fortification of sifted flour was withdrawn in Sweden. GAS provides a perfect baseline for this project with the aim to determine how iron deficiency during adolescence impacts cognitive capability in adulthood. Our theory is that iron status during adolescence has fundamental impact on the adults’ wellbeing and life opportunities.

***Methods***: Two cross-sectional studies were performed in 1994 and 2000 including 2,300 adolescents. At 15–16 years, dietary habits, physical activity/performance, health, growth, and other lifestyle habits were investigated. Blood samples were drawn. By new active consent, a longitudinal cohort is created and baseline data will be linked to population registers and surveys at 32/38 years. Iron status at age 15–16 years is compared to cognitive performance at 18 years as well as wellbeing and academic performance later in life.

***Results***: The prevalence of iron deficiency increased from 37 to 45% in girls while it was 23% in boys both years (serum ferritin ≤15 µg/L).

***Conclusion***: Discussion: It is probable that many of the girls defined as iron deficient at age 15–16 have insufficient iron stores also in adult life. For the boys, growth spurt induced iron deficiency will cease and iron balance will restore. However, the long-term consequences of iron deficiency on brain maturation and possible negative effects on cognitive function are presumed to be equal for both genders. Today's focus on vegetarian eating put the focus on iron. There are potential conflicts between sustainability for environment and sustainability for health. Iron nutrition needs to be revisited with the performance of this project.

**Disclosure of interest**: None to declare.

### Poster presentation no. P444

#### Program drop out and weight loss success during treatment of morbidly obese female patients with type 2 diabetes mellitus

##### Alfons Ramel^1^, Audur Benediktsdóttir^1^, Gudrun Bragadottir^2^, Kristjana Einarsdóttir^1^* and Thorhallur Halldorsson^1^
^1^Unit for Nutrition Research, University of Iceland, Reykjavík, Iceland; ^2^Reykjalundur Rehabilitation Center, Reykjavík, Iceland *Presenting author

***Background and aims***: Weight loss has been suggested to be particularly challenging for obese patients with type 2 diabetes mellitus, although direct comparisons between diabetics and non-diabetics are scarce. The aim was to compare program drop out and weight loss success between diabetic, pre-diabetic, and non-diabetic morbidly obese women following conventional weight loss treatment.

***Methods***: Participants were 181 non-diabetic, 37 pre-diabetic, and 42 diabetic morbidly obese women who participated in a weight loss program at the Reykjalundur Rehabilitation Center, Iceland, between March 2007 and May 2009. Fasting glucose and blood lipids were measured at baseline, with anthropometric measurements conducted at two additional time points; following ambulatory treatment and following stationary treatment. Linear and logistic regression models were used to assess the effect of diabetes on program drop out and weight loss success.

***Results***: Mean BMI of the participants was 44.2±6.4 kg/m^2^ and was similar between the three patient groups. Mean age was lowest for non-diabetic patients (38.5±11.7 years) and highest for diabetic patients (50.2±12.7 years). Total drop-out rate during the program was 37.3% and did not differ significantly between the three groups. Total weight loss was higher for diabetic patients (11.84±7.78 kg) compared with non-diabetic patients (8.78±5.37 kg), due to weight loss difference during ambulatory treatment only (*p*=0.01). The difference was not statistically significant after adjustment for age (*p*=0.08).

***Conclusion***: Direct comparison between obese diabetic and non-diabetic patients suggests no difference in weight loss success between the groups following a lifestyle/weight loss program. Further research is recommended to corroborate these findings.

**Disclosure of interest**: None to declare.

### Poster presentation no. P445

#### New dietary advice in Sweden – how are they perceived? Responses to a holistic approach

##### Karolin Bergman*, Christina Fjellström and Helena Elmståhl Food, Nutrition and Dietetics, Uppsala University, Uppsala, Sweden *Presenting author

***Background and aims***: The connection between food and health are often described in a nutri-centric language, where a reductive focus on nutrients and its implications for health are given a primary status to explain wellbeing. This way of conceptualizing food and health can be seen as reductional nutrition or nutritionism, and has been said to dominate American dietary guidelines. Dietary guidelines in Sweden are provided by the National Food Agency based on scientific research signified in the Nordic nutrition recommendations (NNR) in combination with knowledge about dietary habits in the Swedish population today. The updated Swedish dietary guidelines 2015 are now more holistic and involve other aspects than just the nutritional substituents in food.

***Methods***: When developing Swedish dietary guidelines perspectives from experts and interested parties are regarded, and before launching the updated dietary guidelines 2015 there was an opportunity to give written comments on the proposal open to anyone. By a qualitative thematic analysis of the response documents on the updated guidelines (*n*= 38), we explore the question of how this new more holistic approach of presenting dietary guidelines are reacted upon in terms of how “healthy eating” are discussed in the responses.

***Results***: Preliminary results reveal that in the responses foods are often valued, compared, and argued for by a single nutrient. There is a demand for measurable advice and the Swedish concept “lagom”–“just right” is not appreciated in the advice. But at the same time the importance of remembering the role of food in everyday life is stressed.

***Conclusion***: It could be concluded that the respondents have to a great extent expectations on the dietary guidelines to remain nutritionally reductive and quantified, and find them unclear or unbalanced in the more holistic format.

**Disclosure of interest**: None to declare.

### Poster presentation no. P446

#### Time scarcity and use of ultra-processed food products among Norwegian parents: a cross-sectional study

##### Ingrid Djupegot*, Camilla B. Nenseth, Helga B. T. Bjørnarå, Tonje H. Stea and Elling Bere Department of Public Health, Sport and Nutrition, University of Agder, Kristiansand, Norway *Presenting author

***Background and aims***: Use of ultra-processed food products (e.g. ready-meals, fast food, soft drinks) has expanded rapidly over the last decades. These products require minimal time for preparation, and it is reasonable to assume a possible link between time scarcity and ultra-processed food consumption (UPFC). The main aim of this study was to examine the relationship between time scarcity and UPFC. We also investigated the association between several sociodemographic factors and UPFC.

***Methods***: This study was part of the Healthy and Sustainable lifestyle project in Southern Norway. Participants were 497 kindergarten parents. Three scores were developed as indicators of UPFC: Ultra-processed dinner products, snacks and soft drinks, and fast food away from home. The scores were dichotomized into a high and low consumption group. Chi-square was used to calculate proportions of high versus low UPFC in relation to time scarcity and sociodemographic factors. Binary logistic regression analyses were used to test the relationship between independent variables and UPFC.

***Results***: Participants with medium (OR=1.98, 95% CI=1.26–3.11) and high (OR=1.66, 95% CI=1.03–2.67) time scarcity had higher odds of being in the high consumption group of fast food away from home. Participants with higher education were less likely to have a high consumption of snacks and soft drinks (OR=0.64, 95% CI=0.43–0.96). Native Norwegians were more likely to have a high consumption of snacks and soft drinks (OR=2.82, 95% CI=1.44–5.51) and fast food away from home (OR=2.02, 95% CI=1.05–3.90). Men were more likely to be in the high consumption group of fast food away from home (OR=1.92, 95% CI=1.05–3.54).

***Conclusion***: This study indicates that time scarcity, sex, ethnicity, and educational status are related to different aspects of ultra-processed food consumption. Such knowledge is important in order to develop effective nutritional strategies.

**Disclosure of interest**: None to declare.

### Poster presentation no. P447

#### Determinants of ultra-processed food consumption among young adults: a review of the literature

##### Camilla B. Nenseth*, Ingrid Djupegot, Elling Bere and Tonje Holte Stea Department of Public Health, Sport and Nutrition, University of Agder, Kristiansand, Norway *Presenting author

***Background and aims***: Highly processed foods and beverages (e.g. ready-meals, fast food, soft drinks) have been classified as ultra-processed foods (UPF) and are now to a large extent dominating the global food market. High consumption of UPF has been positively associated with risk of overweight, obesity, and diabetes. In order to reduce consumption of UPF, identifying determinants related to intake of such products is of crucial importance. Young adults are an important and often neglected group for non-communicable disease prevention. Thus, the aim of this paper was to review the literature on determinants of UPF consumption in this group (18–35 years).

***Methods***: Papers were identified from Medline, PsycInfo, SocIndex, and Business Source Complete using search terms indicative of UPF consumption in combination with search terms for determinants. Two researchers independently assessed the material based on inclusion and exclusion criteria, and the strength of evidence was evaluated. A total of 71 articles (published in 1983–2015) investigating a wide range of factors associated with UPF consumption were included.

***Results***: Preliminary findings indicate that younger age and lower socio-economic status are associated with higher use of UPF, and that men have a higher consumption of fast food and soft drinks. TV-watching, stress, cooking, past behavior in childhood/adolescence, availability, modeling, and place of residence (family home/university campus) have also been reported to be associated with UPF consumption. However, further analyses are necessary in order to draw valid conclusions about direction of association.

***Conclusion***: Determinants best supported by evidence are sex, age, and socio-economic status. Future research should investigate determinants more thoroughly, preferably with longitudinal study design and an ecological approach.

**Disclosure of interest**: None to declare.

### Poster presentation no. P448

#### Potential impact of breakfast programs on health and wellbeing in Western Europe

##### Evelyn Hannon^1^* and Bruce Learner^2^
^1^Hannon Nutrition Consultancy, Co. Clare, Dublin, Ireland; ^2^Senior Manager CSR and Partnerships Kellogg Europe, CAF – Corporate Responsibility, Dublin, Ireland *Presenting author

***Background and aims***: School based breakfast programs are increasingly being established in Europe. This project aims to assess the impact of such programs on dietary shortfalls in a number of European countries including Sweden and Denmark.

***Methods***: (i) A literature review was conducted to assess differences in compliance with food- and nutrient-based dietary guidelines and prevalence of suboptimal status of micronutrients and breakfast skipping between low and high socioeconomic groups. MEDLINE databases were searched to collect original studies and reviews published from 1990 to 2015. Studies involving >100 subjects whose dietary intake had been assessed at the individual level and/or used best practice biomarkers reflecting micronutrient status were included. (ii) Dietary modeling was conducted to assess the impact of foods provided at breakfast clubs in addressing dietary shortfalls. Information on foods provided at breakfast clubs was obtained from local organizers in a number of European countries. Macronutrient and micronutrient composition of foods was accessed using local food composition databases.

***Results***: A positive association was found between socioeconomic status and micronutrient and fruit and vegetable intake and daily breakfast consumption. Foods routinely provided at breakfast clubs (e.g. ready to eat breakfast cereals, fruit and vegetables, bread) can provide important contributions (17–50% of Dietary Reference Values) to intakes of key nutrients e.g. vitamin D, folate, vitamin B2, iron, and iodine. Evidence of low intakes of these nutrients exists among lower socioeconomic groups in Europe.

***Conclusion***: These findings may warrant further research and have implications for public health policy in addressing socioeconomic dietary inequalities in Europe. School based breakfast clubs may form part of a multi-pronged approach to address nutritional and health inequalities between people with different socioeconomic status.

**Disclosure of interest**: E. Hannon: None to declare; B. Learner: Received funding for this project by Kellogg Company.

### Poster presentation no. P449

#### Dietary habits across the lifespan and risk of monoclonal gammopathy of undetermined significance

##### Marianna Thordardottir^1^*, Ebba Lindqvist^2^, Sigrun Lund^1^, Rene Costello^3^, Johanna Torfadottir^4^, Debra Burton^3^, Laufey Steingrimsdottir^4^, Neha Korde^5^, Sham Mailankody^5^, Gudny Eiriksdottir^6^, Lenore Launer^7^, Tamara Harris^7^, Ola Landgren^5^, Vilmundur Gudnason^1,6^ and Sigurdur Kristinsson^1,2^
^1^Faculty of Medicine, University of Iceland, Reykjavik, Iceland; ^2^Department of Medicine, Division of Hematology, Karolinska University Hospital and Karolinska Institutet, Stockholm, Sweden; ^3^Multiple Myeloma Section, National Cancer Institute, National Institute of Health, Bethesda, United States; ^4^Unit for Nutrition Research, University of Iceland, Reykjavik, Iceland; ^5^Myeloma Service, Division of Hematologic Oncology, Memorial Sloan-Kettering Cancer Center, New York, United States; ^6^Icelandic Heart Association, Kopavogur, Iceland; ^7^National Institute on Aging, National Institute of Health, Bethesda, United States *Presenting author

***Background and aims***: All multiple myeloma (MM) cases are preceded by the premalignant state, monoclonal gammopathy of undetermined significance (MGUS). The etiology of MGUS and MM is to a large extent unknown. Few studies on the effect of diet on MM have been conducted, and the results have been inconclusive. No studies have been conducted on the effect of diet on MGUS. Our aim was to explore the effect of high versus low intake of fish, fish oil, meat, milk, fruits, vegetables, potatoes, rye bread, whole wheat bread, and oatmeal and muesli on MGUS and MM.

***Methods***: This study was based on participants from the AGES-Reykjavik Study (*n*=5,764). We performed serum protein electrophoresis on all subjects to identify MGUS. Participants answered questionnaires about lifetime dietary habits. Data on MM diagnosis was collected through the Icelandic Cancer Registry. Logistic regression and Cox regression models were used to analyze the risk of MGUS and MM; adjustments were made for age and gender.

***Results***: A total of 300 MGUS (5.2%) cases was identified. We found that high intake of fruits in adolescence and high intake of whole wheat bread in midlife were inversely associated with MGUS (odds ratio (OR)=0.63, 95% confidence interval (CI) 0.52–0.97 and OR=0.76, 95% CI 0.59–1.00, respectively). We also found that high intake of rye bread and potatoes in both adolescence and midlife was inversely associated with MGUS (OR=0.70, 95% CI 0.55–0.95 and OR=0.63, 95% CI 0.45–0.96, respectively). A total of 18 individuals were diagnosed with MM during a mean follow-up of 8.2 years. We found that high fruit intake in late life reduced the risk of progression to MM (hazard ratio (HR)=0.30, 95% CI 0.11–0.82).

***Conclusion***: Our findings suggest that high intake of fruits, rye and whole wheat bread, and potatoes in adolescence and/or midlife may reduce the risk of MGUS, and that high fruit intake in late life may reduce the risk of progression to MM.

**Disclosure of interest**: None to declare.

### Poster presentation no. P450

#### Development of a scoring system to quantify the intake of animal versus vegetable protein and the association with HbA1c and eGFR – a sub-study of the PREVIEW project

##### Grith M. Poulsen^1,^*, Diewertje Sluik^2^, Lars O. Dragsted^1^, Jennie Brand-Miller^3^, Thomas M. Larsen^1^, Sally Poppitt^4^, Marta Silvestre^4^, Edith J. M. Feskens^2^ and Anne Raben^1^
^1^Department of Nutrition, Exercise and Sports Science, University of Copenhagen, Copenhagen, Denmark; ^2^Division of Human Nutrition, Wageningen University, Wageningen, Netherlands; ^3^School of Life and Environmental Sciences & Charles Perkins Centre, The University of Sydney, Sydney, Australia; ^4^Human Nutrition Unit, School of Biological Sciences, University of Auckland, Auckland, New Zealand *Presenting author

***Background and aims***: To assess the impact of a high versus low protein intake in relation to diabetes risk and safety aspects, we developed a scoring tool, in particular on animal versus vegetable protein. Analyses were based on observational study populations included in the PREVIEW project.

***Methods***: We analyzed cross-sectional data from two Dutch observational studies conducted among general adult populations: NQplus (*n*=1,048) and Lifelines (*n*=57,349). Dietary intake data from food-frequency questionnaires were used to develop a protein score, which consisted of two components: percentage of energy from total protein and the ratio of animal to vegetable (A:V) protein. Subjects were divided into 11 strata of total protein intake (en%) and 11 strata of the A:V ratio. A subject could receive a combined score of 0–20 points, where a higher score reflected a higher intake of total protein, and a lower A:V ratio. The associations between the protein score and HbA_1c_ and renal function calculated as eGFR, using the CKD-EPI equation, were examined using multiple linear regression with adjustment for age, sex, education, BMI, prevalent hypertension or hypercholesterolemia, smoking status, alcohol consumption, and physical activity.

***Results***: We found a negative association between the protein score and HbA_1c_ levels in Lifelines (β=−0.037±0.005, *p*<0.001) but not in NQplus. In both Lifelines (β=0.17±0.02, *p*<0.001) and NQplus (β=0.44±0.12, *p*<0.001), there was a positive association between the protein score and eGFR.

***Conclusion***: Our preliminary analyses suggest that a diet rich in protein and with a lower ratio of animal to vegetable (A:V) protein may be associated with a lower risk of diabetes and an increased eGFR.

**Disclosure of interest**: G. M. Poulsen: None to declare; D. Sluik: None to declare; L. O. Dragsted: None to declare; J. Brand-Miller: President of the Glycemic Index Foundation, a non-for-profit food endorsement program, Manager of a GI testing service at the University of Sydney and the co-author of books about the GI foods; T. M. Larsen: None to declare; S. Poppitt: None to declare; M. Silvestre: None to declare; E. J. M. Feskens: None to declare; A. Raben: None to declare.

### Poster presentation no. P451

#### Sweet weekend? Intake of added sugar according to day of the week in Norwegian 4th and 8th graders

##### Jannicke B. Myhre*, Anne M. W. Johansen, Lisa B. Hansen and Lene F. Andersen Department of Nutrition, University of Oslo, Oslo, Norway *Presenting author

***Background and aims***: The intake of added sugar in Norwegian children and adolescents has previously been found to be higher than desirable. Saturday has traditionally been the day when sweets are consumed; commonly known as “Saturday-sweets.” In the present study, we examined if intake of added sugar was higher on Saturdays than on the other days of the week among 4th and 8th graders.

***Methods***: In the national Ungkost-3 survey, 1,322 participants (636, 4th graders, and 686, 8th graders) recorded their diet for 3–4 days using a web-based food record (WebFR) based on the Danish application WebDASC. The WebFR is structured by meals and includes about 570 of the most commonly eaten foods and beverages in Norway. Each food has 2–4 pictures for portion-size assessment. Nutrient intake was calculated using the software KBS, developed at the Department of Nutrition, University of Oslo.

***Results***: In both age groups, the intake of added sugar was higher on Saturdays (20–21 E%) than on both weekdays (8–9 E%), Fridays (13–14 E%) and Sundays (14–15 E%). Added sugar intake was also higher on Fridays and Sundays compared to weekdays (13–15 E% vs. 8–9 E%).

***Conclusion***: Intake of added sugar was on average below the maximum recommended levels on weekdays, but exceeded recommended levels on Fridays, Saturdays, and Sundays. Although sugar intake seemed to spread out across the weekend, a distinct peak was still seen for Saturdays.

**Table 1:** Intake of added sugar according to day of the week in Norwegian 4th and 8th graders

	Weekdays^1^	Friday	Saturday	Sunday
4th grade				
Days (n)	1,566	344	337	279
Participants (n)	636	344	337	279
Added sugar, E% (SD)	8 (7)	14^a^ (9)	21^a^^b^^c^ (11)	15^a^ (9)
8th grade				
Days (n)	1,625	430	419	251
Participants (n)	686	430	419	251
Added sugar, E% (SD)	9 (8)	13^a^ (10)	20^a^^b^^c^ (13)	14^a^ (10)

E%, percentage of energy.

1 Monday to Thursday.

a *p*<0.01 compared to weekdays;

b *p*<0.01 compared to Fridays;

c *p*<0.01 compared to Sundays (linear mixed model).

**Disclosure of interest**: None to declare.

### Poster presentation no. P452

#### Difference in characteristics between responders and non-responders of an FFQ

##### Anja L. Madsen*, Line Tang, Ulla Toft, Betina H. Thuesen and Allan Linneberg Research Centre for Prevention and Health, The Capital Region of Denmark, Glostrup, Denmark *Presenting author

***Background and aims***: A food frequency questionnaire (FFQ) designed to obtain information about food consumption during the last month was part of the health survey “Health2010.” All participants had a health examination but not all completed the FFQ. This gave us opportunity to evaluate differences between responders and non-responders of the FFQ. Furthermore, we evaluated the nutritional calculations.

***Methods***: A random sample of residents in the Southwestern part of Copenhagen aged 18–70 years was invited for the Health2010 study at Research Centre for Prevention and Health. All participants were asked to complete a self-administered 240 food-item FFQ with gender-standardized portion sizes.

***Results***: In total, 1,522 (participation rate: 41%) participated in Health2010, of whom 1,325 (87%) completed the FFQ. More women (56 vs. 44%, *P*<0.0001) than men participated in Health2010, but there was no gender difference in completing the FFQ (*P*=0.16). Responders were older (49 vs. 46 years, *P*=0.0048) and more often rated their diet as healthy (94 vs. 90%, *P*=0.0304). Also their self-estimated social position on a 10-point scale was higher than in non-respondents (6.5 vs. 6.1 point, *P*=0.0256). Number of people in the household and occupational status did not differ between respondents and non-respondents (both *P*>0.69). Mean-measured BMI (26.1±4.4 kg/m^2^, *P*=0.92) was not associated with non-completion of the FFQ. Mean estimated energy intake was 9.3±3.0 MJ/d. Mean estimated physical activity level (PAL) was 1.46±0.49 and 23% had PAL<1.1. There was a negative correlation between BMI and PAL (–0.266, *P<*0.0001).

***Conclusion***: Health2010 participants completing the FFQ were older and rated their own diet healthier than non-respondents. The nutritional calculation seemed reliable at group level. However, 23% of the participants had a low PAL indicating underreporting or setting to small portion sizes.

**Disclosure of interest**: None to declare.

### Poster presentation no. P453

#### Daily salt intake among Danish adults and the association with cardiovascular disease after 11 years of follow-up

##### Betina Thuesen*, Rikke Jacobsen, Ulla Toft, Allan Linneberg and Torben Jørgensen Research Centre for Prevention and Health, The Capital Region of Denmark, Glostrup/Copenhagen, Denmark *Presenting author

***Background and aims***: In order to prevent chronic diseases, WHO recommend a population average consumption of salt less than 5 g/day. Despite ongoing efforts to reduce the salt intake in many developed countries, the actual intake is far above the recommendations in most populations. Whereas the association between salt intake and blood pressure (BP) is well established a causal relation between high salt intake and increased risk of CVD is more controversial. The objective of this study was to evaluate the association between estimated daily salt intake and BP in a cross-sectional setting and in addition the association with events of ischemic heart disease (IHD) or stroke during 11 years of follow-up.

***Methods***: We included 5,793 participants (29% men) aged 30–60 years from the Danish Inter99 cohort. Daily salt intake was estimated from measurements of creatinine and sodium in samples of spot urine. BP was measured at baseline, and all participants were followed in the Danish national patient registry for registration of incident IHD/stroke.

***Results***: The mean estimated daily salt intake among men was 10.2 g (25%/75% percentiles: 8.8 g/11.5g) and among women 7.3 g (25%/75% percentiles: 6.8 g/7.9 g). High salt intake was significantly associated with high BP (*p*<0.0001, linear regression adjusted for sex and age). During follow-up 434 events of IHD/stroke were observed. The association between daily salt intake and IHD/stroke varied between men and women (*p*(interaction)<0.006). However, associations were significant but non-linear for both genders.

***Conclusion***: The estimated daily mean intake of salt was 10.2 g among men and 7.3 g among women. A high daily salt intake was significantly associated with high BP. Also a high daily salt intake was significantly associated with incident IHD/stroke – however the association was complex.

**Disclosure of interest**: None to declare.

### Poster presentation no. P454

#### Meals with salmon has potential to suppress appetite sensations to a greater extent than meals with veal

##### Lone V. Nielsen^1^*, Signe Nyby^2^, Lars Klingenberg^1^, Christian Ritz^1^, Bjørn Liaset^3^, Karsten Kristiansen^2^, Lise Madsen^2,3^ and Anne Raben^1^
^1^Department of Nutrition, Exercise and Sports, University of Copenhagen, Copenhagen, Denmark; ^2^Department of Biology, University of Copenhagen, Copenhagen, Denmark; ^3^National Institute of Nutrition and Seafood Research, Bergen, Norway *Presenting author

***Background and aims***: Proteins increase satiety compared to carbohydrates and fat. However, little is known on how protein from different sources affects appetite and if it is modified by the type of carbohydrate. Therefore, we investigated the acute effects of meals containing proteins from fish versus meat in combination with high and low glycemic index (GI) carbohydrates on subjective appetite sensation, energy intake, and ghrelin.

***Methods***: We included 25 healthy overweight men and women (mean±SD age: 28.8±7.6 years, BMI: 27.5±1.5 kg/m^2^) in a randomized crossover study. Four iso-caloric meals were tested (energy %: 41 carbohydrate, 34 fat, and 25 protein); salmon+mashed potatoes, salmon+pasta, veal+mashed potatoes, and veal+pasta. Subjective appetite sensations were measured at baseline and every half hour until an *ad libitum* lunch was served 3.5 h later. Blood samples were drawn at baseline and every 20 min.

***Results***: Participants were more satiated and full and had lower prospective food consumption (PFC), after salmon+mashed potatoes compared to veal+pasta (*p*<0.05). Palatability was, however, lower for salmon+mashed potatoes (*p*<0.05). After adjusting for palatability, only the difference in PFC between salmon+mashed potatoes and veal+pasta remained significant (*p*<0.05). Ghrelin levels were significantly suppressed after the salmon compared with the veal meals. However, there were no differences in *ad libitum* energy intake (*p*=0.98).

***Conclusion***: The combination of protein sources and the GI of the carbohydrates influence subjective appetite sensations. Our results indicate that salmon has potential to suppress appetite to a greater extent than veal, independently of the carbohydrates GI.

**Disclosure of interest**: None to declare.

### Poster presentation no. P455

#### Maternal diet, gestational weight gain, and inflammatory markers during pregnancy

##### Laufey Hrolfsdottir^1,2^*, Casper G. Schalkwijk^3^, Bryndis E. Birgisdottir^1^, Ingibjorg Gunnarsdottir^1^, Ekaterina Maslova^2,4,5^, Charlotta Granström^2^, Marin Strøm^2^, Sjurdur F. Olsen^2,6^ and Thorhallur I. Halldorsson^1,2^
^1^Unit for Nutrition Research, Landspitali University Hospital and Faculty of Food Science and Nutrition, University of Iceland, Reykjavik, Iceland; ^2^Center for Fetal Programming, Department of Epidemiology Research, Statens Serum Institute, Copenhagen, Denmark; ^3^Department of Internal Medicine, Laboratory of Metabolism and Vascular Medicine, Maastrich University Medical Center, Maastrich, The Netherlands; ^4^Danish Diabetes Academy, Odense, Denmark; ^5^Department of Epidemiology and Biostatistics, Imperial College, London, United Kingdom; ^6^Department of Nutrition, Harvard School of Public Health, Boston, MA, USA *Presenting author

***Background and aims***: Weight gain and poor dietary habits have been associated with low-grade inflammation in non-pregnant subjects. Whether the same holds for pregnant women has not been explored in detail, but is of importance since elevated maternal inflammatory response has been associated with pregnancy complications. Our objective was to examine the associations of gestational weight gain (GWG) and diet with low-grade inflammation in pregnant women.

***Methods***: A cross-sectional analysis of 671 pregnant women. Diet was assessed in gestational week 30 with food frequency questionnaires and interviews. GWG was recorded at weeks 30 and ~37. Markers of inflammation, high-sensitivity C-reactive protein (hsCRP), Serum amyloid A (SAA), Interleukin-6, (IL-6), IL-8, IL-1β, and Tumor necrosis factor α (TNF-α) were quantified with multi-arrays in serum from week 30.

***Results***: Mean±standard deviation for pre-pregnancy body mass index and GWG up to week 30 was 21±2 kg/m^2^ and 10±4 kg, respectively. In adjusted models, each 1-kg increase in GWG was associated with 3% (95% CI: 1, 5) higher hsCRP and 3% (95% CI: 1, 4) higher SAA concentrations. This corresponded to ~18–25% increase in hsCRP and SAA concentrations among those with excessive compared to optimal/suboptimal GWG. Intake of animal protein was positively associated with hsCRP and SAA concentrations while intake of plant protein was inversely associated with these inflammatory markers (*P* for trend<0.05). More precisely, women in the highest compared to lowest quintile of animal protein intake had 25% (95% CI: 2, 53) higher hsCRP concentrations, while those in the highest compared to lowest quintile of plant protein had 24% (95% CI: 6, 38) lower concentrations. A similar pattern was observed for SAA

***Conclusion***: In a cohort of lean pregnant women, excessive GWG, as well as high intake of animal protein, were associated with higher concentrations of markers of inflammation.

**Disclosure of interest**: None to declare.

### Poster presentation no. P456

#### Iodine intake in subgroups of Norwegians – indication of suboptimal iodine intakes from food

##### Anne L. Brantsaeter^1^*, Kristine Nyheim^2^, Nina C. Johansen^2^, Helle M. Meltzer^1^, Helle K. Knutsen^1^ and Sigrun Henjum^2^
^1^Infection Control and Environmental Health, Norwegian Institute of Public Health, Oslo, Norway; ^2^Oslo and Akershus University College of Applied Sciences, Oslo, Norway *Presenting author

***Background and aims***: The Norwegian population has been considered iodine replete for decades. The aim of this study was to assess iodine status in subgroups of the Norwegian population based on age (children, adolescents, adults, and elderly), life cycle (pregnancy), or dietary practice (vegetarians and vegans).

***Methods***: Participants (*n*=276) recorded all food and drink consumed for 2 days and delivered two fasting morning spot urine samples on the following mornings for analysis of urinary iodine concentration (UIC).

***Results***: The median iodine intake in all participants was 103 µg/day, and the median UIC was 77 µg/L. Participants in all subgroups had iodine intakes below Nordic Nutrition Recommendations (Table 1). This was supported by the majority having low UIC. Milk and dairy food was the main iodine source (40–60%) in all subgroups except vegans.

***Conclusion***: Suboptimal iodine intakes in all subgroups were found. Although the participants are not representative of the Norwegian population, the results highlight the need to increase the focus on iodine nutrition in developed countries.

**Abstract 456–Table 1:** Iodine intake from food records and morning spot UIC in subgroups

Subgroup	*N*	Iodine intake, µg/day Median (P5, P95)	Iodine below recommended intake a*n*(%)	UIC bc<100 µg/L *n*(%)
Children 3–9 years	47	93 (52, 197)	31 (66)	19 (40)
Adolescents 10–17 years	46	106 (36, 337)	29 (63)	34 (74)
Adults 18–64 years	68	115 (50, 285)	47 (69)	44 (65)
Elderly 65+ years	23	107 (42, 228)	16 (70)	18 (78)
Pregnant women	40	124 (43, 254)	28 (70)	27 (68)
Ovo-Lacto-Pesco Vegetarians	34	89 (29, 251)	26 (77)	25 (74)
Vegans	18	24 (13, 88)	18 (100)	15 (83)

a NNR12; Nordic Nutrition Recommendation 2012. Recommended intake: 90 µg/d for children 2–5 years, 120 µg/d for children 10–13 years, 175 µg/d for pregnant women, and 150 µg/d for adolescents 14–17 years, adults, elderly and vegetarian group.

b UIC; Urinary Iodine Excretion.

c Excluding five participants with UIC>600 µg/L.

**Disclosure of interest**: None to declare.

### Poster presentation no. P457

#### Association between sugar intake and acute coronary event risk in the Malmö Diet and Cancer cohort

##### Khadija Khalif, Isabel Drake, Peter Wallström, Gunnar Engström and Emily Sonestedt* Department of Clinical Sciences in Malmö, Lund University, Malmö, Sweden *Presenting author

***Background and aims***: Previous studies have suggested that a high intake of sugar-sweetened beverages is positively associated with the risk of a coronary event. However, few studies have examined the association between sucrose (i.e. sugar) and incident coronary events. The aim of the present study was to examine the associations between sucrose intake and coronary event risk.

***Methods***: We performed a prospective analysis on 26,190 individuals (62% women) free from diabetes and without a history of cardiovascular diseases from the Swedish population-based Malmö Diet and Cancer cohort. Over an average of 17 years of follow-up, 2,493 incident cases of coronary events (fatal or non-fatal myocardial infarction or death attributable to ischemic heart disease) were identified from registers. Sucrose intake was obtained from an interview-based diet history method, including 7-day records of prepared meals and cold beverages and a 168-item diet questionnaire covering other foods. Cox proportional hazards regression was used to model the association between sucrose and coronary event risk adjusted for gender, age, energy intake, dietary method, season, smoking, waist, alcohol consumption, physical activity, and educational level. A restricted cubic spline was computed to examine the shape of the association between sucrose intake and coronary event risk.

***Results***: Participants who consumed more than 15% of their energy intake (E%) from sucrose (5% of this population) had a 32% (95% CI=9–59%) increased risk of a coronary event compared with the lowest sucrose consumers (<5 E%). In addition, we observed a non-linear association indicating that the risk increased above the median intake (8.2 E%), and no benefit of having a lower sucrose intake was observed.

***Conclusion***: The results indicated that high sucrose consumption is associated with an increased risk of a coronary event.

**Disclosure of interest**: None to declare.

### Poster presentation no. P458

#### Pre-diagnostic vitamin D levels in older individuals and risk and progression of breast- and prostate cancer

##### Johanna E. Torfadottir^1,2^*, Unnur Valdimarsdóttir^2^, Thor Aspelund^2,3^, Laufey Tryggvadóttir^4^, Mary F. Cotch^5^, Meir Stampfer^6,7^, Lorelei Mucci^6,7^, Edward Giovannucci^6,7^, Vilmundur Gudnason^3,8^ and Laufey Steingrimsdottir^9^
^1^Unit for Nutrition Research, University of Iceland, Reykjavik, Iceland; ^2^Centre of Public Health Sciences, University of Iceland, Reykjavik, Iceland; ^3^The Icelandic Heart Association, Kopavogur, Iceland; ^4^The Icelandic Cancer Registry, Reykjavik, Iceland; ^5^Division of Epidemiology and Clinical Applications, Intramural Research Program, National Eye Institute, National Institutes of Health, Bethesda, MD, USA; ^6^Departments of Nutrition and Epidemiology, Harvard T.H. Chan School of Public Health, Boston, MA, USA; ^7^Channing Division of Network Medicine, Department of Medicine, Brigham and Women's Hospital and Harvard Medical School, Boston, MA, USA; ^8^Faculty of Medicine, University of Iceland, Reykjavik, Iceland; ^9^Unit for Nutrition Research, Faculty for Food Science and Nutrition, University of Iceland, Reykjavik, Iceland *Presenting author

***Background and aims***: Vitamin D status may predict survival after a cancer diagnosis even if it is not associated with cancer risk. Our aim was to explore whether pre-diagnostic levels of vitamin D, in serum of older individuals (66 to 98 years of age) in Iceland were associated with risk of breast and prostate cancer and subsequent survival.

***Methods***: We used data from the Reykjavik-AGES Study initiated in 2002. Participants were 2,136 men and 2,977 women, cancer free at entry when 25-hydroxy-vitamin-D (25-OHD) was measured. Cox proportional hazard regression models were used to assess the association between serum 25-OHD and subsequent cancer diagnosis and all-cause mortality through 2013. Multivariable adjustments were made for potential confounders.

***Results***: Cancer was subsequently diagnosed in 91 women (mean age 80.7 years; 4.6 years after entry) and 151 men (mean age 78.7 years; 3.7 years after entry). Of these, 34 women (37%) and 55 men (36%) died during follow-up. Lowest tertile of serum 25-OHD (≤53 nmol/L) was not significantly associated with breast or prostate cancer risk. However, both men and women in the highest tertile of 25-OHD levels (≥63.7 nmol/L) before cancer diagnosis, were less likely to die from any cause compared with those in the lowest tertile (hazard ratio (HR)=0.42, 95% confidence interval (CI): 0.19–0.96, P_trend_=0.045 for men and HR=0.40, 95% CI: 0.14–1.13, P_trend_=0.088, for women). For breast cancer-specific mortality (*n*=17), the HR was 0.10 (95% CI: 0.02–0.86; P_trend_=0.028) and 0.61 (95% CI: 0.20–1.82; P_trend_=0.281) for prostate cancer-specific mortality (*n*=23).

***Conclusion***: While based on small numbers, our data suggest that higher pre-diagnostic serum 25-OHD levels may be associated with improved survival for cancer patients. Further studies are needed to rule out the potential effect of preclinical disease on vitamin D levels (reverse causation).

**Disclosure of interest**: None to declare.

### Poster presentation no. P459

#### New Norwegian data on weights, measures, and portion sizes for foods

##### Jorån Ø. Dalane^1^*, Ellen Kielland^1^ and Monica H. Carlsen^2^
^1^Labelling and Quality Section, The Norwegian Food Safety Authority, Oslo, Norway; ^2^Department of Nutrition, University of Oslo, Oslo, Norway *Presenting author

***Background and aims***: Data on the weight of foods and portion sizes are of great importance when assessing individual's diet or when conducting dietary surveys. Food habits and food available on the market are continually changing. The Norwegian data on weights, measures, and portion sizes were last time revised several years ago, thus a revision of these data was needed. The aim of this project was to revise these data including weight yield factors and average sizes of glasses, cups, and spoons.

***Methods***: The sample of the foods revised is mainly based on foods in the Norwegian Food Composition Table. Several methods were used in order to collect and analyze new data. When existing data on the weight of foods were not consistent or if the data were lacking, food items were either weighed or information about weight per item were collected from several convenience chains, cafes, kiosks, etc. Data from the last national dietary survey in Norway (Norkost 3) were used to calculate new portion sizes and new average sizes of glasses and cups.

***Results***: The project resulted in new data on 700 foods and new average sizes of glasses, cups, and spoons. Some foods, such as baked goods, are larger now than last time the data were revised. Some of the old portion sizes were not fully applicable anymore, probably because of changes in food habits. Average sizes of glasses and cups are generally larger now than some decades ago. On the contrary, table spoons are smaller than before. Several modern foods, such as sushi, taco products, and exotic fruits are now included in the Norwegian data.

***Conclusion***: Data on weights, measures, and portion sizes of foods and average sizes of glasses, cups, and spoons should be regularly revised in order to reflect changes in food habits and food available on the market.

**Disclosure of interest**: None to declare.

### Poster presentation no. P460

#### Cross-sectional analyses of plasma fatty acid concentrations with depressive symptoms and major depressive disorder

##### Thorhallur I. Halldorsson^1,2,3^, Cindy M. Imai^1,2^, Thor Aspelund^4,5^, Gudny Eiriksdottir^4^, Lenore J. Launer^6^, Inga Thorsdottir^1,2^, Tamara B. Harris^6^, Vilmundur Gudnason^4^, Ingeborg I. A. Brouwer^7^ and Ingibjorg Gunnarsdottir^1,2^* ^1^Unit for Nutrition Research, Landspitali-The National University Hospital of Iceland, University of Iceland, Reykjavik, Iceland; ^2^Faculty of Food Science and Nutrition, School of Health Sciences, University of Iceland, Reykjavik, Iceland; ^3^Centre for Fetal Programming, Department of Epidemiology Research, Statens Serum Institut, Copenhagen, Denmark; ^4^Icelandic Heart Association, Kopavogur, Iceland; ^5^Faculty of Medicine, School of Health Sciences, University of Iceland, Reykjavik, Iceland; ^6^National Institute on Aging, Laboratory of Epidemiology, and Population Sciences, Bethesda, MD, USA; ^7^Department of Health Sciences and the EMGO+ Institute for Health and Care Research, Faculty of Earth and Life Sciences, VU University Amsterdam, Amsterdam, The Netherlands *Presenting author

***Background and aims***: Deficits in omega-3 fatty acids may be associated with depression. However, data from older adults who are at greater risk of poor dietary intake and of developing depression are scarce. Our objective was to investigate plasma phospholipid fatty acids (PUFAs) with respect to depressive symptoms and major depressive disorder in community-dwelling older adults.

***Methods***: Cross-sectional analyses of 1,571 participants in the Age, Gene/Environment Susceptibility-Reykjavik Study aged 67–93 years. Depressive symptoms were measured using the 15-item Geriatric Depression Scale (GDS-15). Major depressive disorder was assessed according to Diagnostic and Statistical Manual of Mental Disorders (DSM-IV) criteria using the Mini-International Neuropsychiatric Interview (MINI).

***Results***: Depressive symptoms were observed in 195 subjects, and cases of major depressive disorder were 27 (10 men and 17 women). Participants with depressive symptoms were less educated, more likely to be smokers, less physically active and consumed cod liver oil less frequently. Difference in GDS-15 scores by tertiles of fatty acid concentration was not significant. Long chain n-3 fatty acid concentrations (EPA+DHA) were inversely related to major depressive disorder, (tertile 2 vs. tertile 1) OR: 0.31 (95% CI: 0.11, 0.86); tertile 3 vs. tertile 1, OR: 0.45 (95% CI: 0.17, 1.21).

***Conclusion***: In our cross sectional analyses, low concentrations of long chain n-3 PUFAs in plasma phospholipids appear to be associated with increased risk of major depressive disorder. However, the results from this study warrant further investigation in prospective setting with sufficiently long follow-up.

**Disclosure of interest**: None to declare.

### Poster presentation no. P461

#### Dietary supplement use in Europe – an overview

##### Natasha L. Welland^1^, Despoina Theofylaktopoulou^2^ and Jutta Dierkes^1^* ^1^Department of Clinical Medicine; ^2^Department of Clinical Science, University of Bergen, Bergen, Norway *Presenting author

***Background and aims***: Use of dietary supplements in Europe has been increasing in the last decades and is currently exceeding 50% in some countries. Given the increasing trend of supplement use in the last decade, up-to-date comparisons between countries are needed. We aimed to present the current status of dietary supplement use in Europe as assessed in recent representative national dietary surveys. The aim was to investigate the prevalence of dietary supplement use in Europe and to characterize the users and usage of supplements.

***Methods***: To collect and evaluate recently available data on dietary supplements use in adults from national dietary surveys conducted in Europe since the year 2000.

***Results***: The frequency of dietary supplement use varied from 5 to 62%, with a mean prevalence of 37%. A north–south gradient was observed, with a higher consumption in the northern countries. Higher frequency of use was associated with age, female gender, and longer duration of education. Results were difficult to compare due to different methodologies of assessment used.

***Conclusion***: Large differences in supplement use in Europe are evident. Despite the differences, use of supplements was consistently higher among women, older age groups, and those with higher education level across countries. The results demonstrated the need for careful interpretation of available data, for national food consumption surveys to adopt a common methodology for comparisons and for development of improved methods for estimating intakes of micronutrients from dietary supplements.

**Disclosure of Interest**: None to declare

### Poster presentation no. P462

#### Seasonal adjustment of vitamin D using cosinor modelling

##### Eirik M. M. Degerud^1^, Rune Hoff^2^, Ottar Nygård^3^, Elin Strand^3^, Dennis Nilsen^4^, Jan E. Nordrehaug^5^, Stefan de Vogel^6^, Oivind Midttun^7^, Per. Ueland^3^ and Jutta Dierkes^1^* ^1^Department of Clinical Medicine, University of Bergen, Bergen, Norway; ^2^Department of Basic Medical Sciences, University of Oslo, Oslo, Norway; ^3^Department of Clinical Science, University of Bergen, Bergen, Norway; ^4^Department of Cardiology, University Hospital Stavanger, Stavanger, Norway; ^5^Department of Heart Disease, Haukeland University Hospital, Bergen, Norway; ^6^Department of Global Public Health and Primary Care, University of Bergen, Norway; ^7^Bevital AS, Bergen, Norway, Norway *Presenting author

***Background and aims***: Seasonal variation in 25-hydroxyvitamin D concentrations (25OHD) is a source of measurement error that can result in misclassification of subjects according to vitamin D status. We aimed to evaluate the performance of a cosinor model as an approach to adjust for this.

***Methods***: From a cosinor model fitted to baseline 25OHD measurements (*n*=4116), we predicted follow-up measurements (*n*=528). Predictions were compared to true follow-up measurements and the error used as a measure of accuracy. Accuracy was compared to predictions from a linear model with season as a four-level categorical variable and to a non-adjustment approach where baseline measurements were carried forward as predictions.

***Results***: Mean squared error is the difference between predictions and follow-up measurements squared. Differences in predictive accuracy of methods were tested with paired t-tests on squared errors.

***Conclusion***: When seasonal variation in 25OHD measurements is present in observational data, adjusting them with cosinor modelling prior to further analysis is preferable to ignoring the problem and more accurate than linear regression.

**Table:** 

Method	Mean squared error	Method comparison *(p*-value)
Cosinor		
Unadjusted	198	Reference
Plus age	199	0.427
Multivariate	201	0.331
No adjustment	221	0.001
Linear regression	246	<0001

**Disclosure of Interest**: None to declare

### Poster presentation no. P463

#### Sucrose in the Finnish diet: focus on differences between age groups

##### Niina Kaartinen*, Marja-Leena Ovaskainen, Liisa Valsta, Noora Kanerva, Satu Männistö Department of Health, National Institute for Health and Welfare, Helsinki, Finland *Presenting author

***Background and aims***: In Finland, sucrose intake has been used as a proxy measure for added sugar. One shortcoming of this approach is that sucrose also occurs naturally in fruits and vegetables. We aimed to quantify the intake of total, added, and naturally occurring sucrose of adult Finns in different age groups.

***Methods***: The National FINDIET 2012 Survey comprised a total of 1708 adults aged 25–74 years with complete 48-hour dietary recall data gathered by trained interviewers in January–March 2012. The sample encompassed 33% of the FINRISK 2012 Study sample. Based on the Finnish national food composition database, food sources of sucrose were sorted out by disaggregating foods into their ingredients. Added sucrose was calculated as the difference of total and naturally occurring sucrose (sucrose from fruits, berries, vegetables, and 100% fruit juice). The association between age group (25–44 years, 45–64 years, 65–74 years) and sucrose intake was determined using non-parametric Kruskal–Wallis test.

***Results***: The mean daily intake of added sucrose across age groups in men ranged from 33.0 g (65–74 years) to 45.4 g (25–44 years). The corresponding intakes for women ranged from 24.3 g to 37.4 g. On average, naturally occurring sucrose covered from 22% (25–44 years) to 30% (65–74 years) of total sucrose in men and from 26% (25–44 years) to 34% (65–74 years) in women. The association between age and total sucrose (E%) was insignificant in both sexes. However, using added and naturally occurring sucrose as dependent variables, differences between age groups were found: added sucrose intake was the highest in the young (7.9 E%, P=0.013 for men, and 7.9 E%, *P*=0.006 for women) and naturally occurring sucrose the highest in the old (2.3 E%, *P*=0.0004 for men, and 2.9 E%, *P*<0.0001 for women).

***Conclusion***: The distinction between added and naturally occurring sucrose seems important when identifying the challenges of different age groups with regard to sugar intake.

**Disclosure of Interest**: None to declare

### Poster presentation no. P464

#### Physical performance and serum 25(OH)D status in community-dwelling old mobility limited adults: a cross-sectional study

##### Åsa von Berens^1*^, Tommy Cederholm^1^, Roger Fielding^2^, Thomas Gustafsson^3^, Dylan Kirn^4^, Jonathan Laussen^4^, Margaretha Nydahl^5^, Kieran Reid^4^ and Afsaneh Koochek^1^
^1^Public Health and Caring Sciences, Clinical nutrition and metabolism, Uppsala, Sweden; ^2^Jean Mayer USDA Human Nutrition Research Center on Aging, Tufts University, Boston, MA, United States; ^3^Department of Laboratory Medicine, Karolinska Institute, Stockholm, Sweden; ^4^Jean Mayer USDA Human Nutrition Research Center on Aging, Tufts University, Boston, MA, United States; ^5^Department of Food, Nutrition and Dietetics, Uppsala University, Uppsala, Sweden *Presenting author

***Background and aims***: Reports on the association between serum concentrations of 25(OH)D and physical function in on older persons is not unanimous, and the risk for vitamin D deficiency increases with advancing age. The aim of this study was to investigate a possible association between serum concentrations of vitamin D (25 (OH)D) and physical function in older adults with mobility limitations.

***Methods***: A cross-sectional study was performed among 507 older community-dwelling (77±5 y) subjects. Mobility was measured by the Short Physical Performance Battery (SPPB, 0-12 p) including its sub-components; chair stand, gait speed and balance. Only subjects with SPPB score ≤ 9 were included. Logistic regression analysis was performed for the SPPB total score and linear regression analyses to relate 25(OH)D to the SPPB subcomponents.

***Results***: No clear association between 25(OH)D and total SPPB score was detected. However, a significant quadratic relationship was observed for serum 25(OH)D and the performance of five repeated chair stands, meaning that at serum levels ≥83 nmol/L higher serum concentrations was associated with faster chair stand test, whereas for serum levels <83 nmol/L this association was not observed.

***Conclusion***: There was no clear association between serum 25(OH) D and physical performance measured as SPPB total score, but an association existed between improved chair stand test and high serum levels of 25(OH)D in community-dwelling older adults.

**Disclosure of Interest**: None to declare

### Poster presentation no. P465

#### Dietary patterns among Finnish preschool children and their parents

##### Henna Vepsäläinen^1^, Liisa Korkalo^1^*, Vera Mikkilä^1^, Kaija Nissinen^1^, Essi Skaffari^1^, Reetta Lehto^2^, Carola Ray^2^, Nina Sajaniemi^3^, Eva Roos^2^, Maijaliisa Erkkola^1^ and the DAGIS consortium group ^1^Department of Food and Environmental Sciences, University of Helsinki, Finland; ^2^Folkhälsan Research Centre, Helsinki, Finland; ^3^Department of Teacher Education, University of Helsinki, Helsinki, Finland *Presenting author

***Background and aims***: Dietary pattern approach has become popular in nutrition science, but there is only limited information on dietary patterns among preschool-aged children and their parents. Our aim was to study familial dependence of dietary patterns among Finnish children and their parents. In addition, we will investigate the associations between socio-economic status (SES) and the dietary patterns.

***Methods***: The present analyses are a part of the DAGIS study that investigates energy balance-related behaviors and stress among preschool children. The participants were 3- to 6-year-old children (*n*=382) from 57 preschools in Finland. Parents filled in 47- (children's diet) and 49-item (parents’ diet) food frequency questionnaires (FFQs) measuring food consumption during the last week. In the children's FFQ, we only measured foods eaten outside daycare. We used principal components analysis to identify dietary patterns using the FFQ food groups as input variables. SES was assessed using self-reported questionnaires.

***Results***: We identified two dietary patterns among the participating children. Pattern 1 was most positively correlated with berries, natural yogurt, plain nuts, and eggs. Pattern 2 was strongly characterized by ice cream, soft drinks, sweet biscuits, and white rice/pasta. Among the parents, two dietary patterns with similar outlines but somewhat different contents were identified: pattern 1 included lots of e.g. fresh fruit, vegetables, and berries. Pattern 2 was characterized by potatoes, sweet pastries, white wholemeal bread, and sausages/luncheon meats. In the upcoming analyses, we will investigate familial associations and SES determinants of the dietary patterns.

***Conclusion***: The study will provide new information on familial dependence and SES determinants of dietary patterns among preschool-children and their parents.

**Disclosure of Interest**: None to declare

### Poster presentation no. P466

#### Participatory research may be an option to decrease socioeconomic bias in recruitment of adolescents to health-related surveys

##### Maria Magnusson^1,2^*, Lena Ljungkrona-Falk^3^, Moa H. Lewis^2^ and John E. Chaplin^4^
^1^Angered Hospital, Angered, Sweden; ^2^Department of Public Health and Community Medicine, Sahlgrenska Academy at the University of Gothenburg, Gothenburg, Sweden; ^3^Primary Healthcare, Närhälsan, Västra Götaland Region, Mariestad, Sweden; ^4^AFBPsS, CPsychol, Inst. Clinical Sciences, Sahlgrenska Academy at the University of Gothenburg, Gothenburg, Sweden *Presenting author

***Background and aims***: The quality of health-related survey results is commonly criticized due to the possibility of selection bias due to low participation of certain groups. Groups that left school prior to higher education, with low income and high rates of unemployment, are commonly under-represented. This kind of selection bias will lead to deficits in societal understanding of different groups’ prerequisites for health and may add to increased health inequity. Increased participation from hard-to recruit-groups is thus one step that would help to reach the WHO goal “Closing the gap”.

The aim of the study was to develop methods with potential to decrease selection bias due to socioeconomic factors, especially in research on adolescents related to lifestyle factors.

***Methods***: Focus group interviews were conducted with adolescents in schools and in a youth recreation center, in areas characterized by social and economic problems in Gothenburg (Sweden). Analysis was conducted by qualitative content analysis.

***Results***: Themes that emerged from the analysis were importance of meaningfulness, in parallel with disappointment due to the belief that their answers are overlooked and under-valued. Questions relating to ethnicity and body weight were considered uncomfortable and potentially embarrassing. Survey questions concerning diet were not regarded as problematic.

***Conclusion***: The call for meaningfulness and feedback could be met by participatory research which draws on shared power between researchers and participants and acknowledges that lay knowledge and scientifically gained knowledge play equally important roles in the research process. The finding that questions about body weight and ethnicity were potentially painful highlights the importance of developing surveys in dialogue with respondent groups.

**Disclosure of Interest**: None to declare

### Poster presentation no. P467

#### Good day – a group activity model for promoting eating and health of under 25 year olds outside working and study life

##### Clarissa Bingham^1^*, Seppo Soine-Rajanummi^1^, Heidi Löflund-Kuusela^1^ and Eeva Ollila^1^
^1^Health Department, Cancer Society of Finland, Helsinki, Finland *Presenting author

***Background and aims***: Socioeconomic health differences are notable in Finland and the risk of social exclusion is emphasized regionally. Young people outside working and study life have a threat of prolonged unemployment at a life stage when independent ways of life become established in adulthood. The Good day group activity model tackles to address these factors through healthy eating and other ways of life.

***Methods***: The Good day group activity model consists of a protocol of group meetings in a course format in the area of Kajaani. The course comprises 6–8 meetings of 3–4 hours during 1–2 months. Course participants consist of 10 young people (<25 years) outside of working and study life recruited by local youth work partners. Three main topics will be handled: eating habits, physical activity, and daily rhythm. Nutritional themes include basics of healthy nutrition, meal patterns, and healthy and economic cooking. The first pilot course will be conducted in May 2016 with further development and regional expansion in autumn 2016.

***Results***: Results of the Good day group activity model will be measured through health behavior measures and changes in ways of life. Eating habits will be assessed by a food frequency questionnaire specially designed for this target group. Other measures include specific precision questions delivered by questionnaire or interview as well as optional risk factor measurements. Consumption frequencies of healthy and unhealthy foods (fruit and vegetables; fast food and confectionary, respectively) act as essential nutritional indicators.

***Conclusion***: The Good day group activity model offers a new approach for reaching young people at risk for social exclusion. The model enables participants to improve eating habits and ways of life as well as coping mechanisms to enhance capability of adjusting to working and study life.

**Disclosure of Interest**: None to declare

### Poster presentation no. P468

#### Unawareness of unhealthy diets is associated with positive indicators of health and health behaviors in Danish adults

##### Mette R. Sørensen^1^*, Jeppe Matthiessen^1^, Lotte Holm^2^, Vibeke K. Knudsen^1^, Elisabeth W. Andersen^3^ and Inge Tetens^1^
^1^National Food Institute, Technical University of Denmark, Søborg, Denmark; ^2^Department of Food and Resource Economics, University of Copenhagen, Frederiksberg C, Denmark; ^3^Department of Applied Mathematics and Computer Science, Technical University of Denmark, Kgs. Lyngby, Denmark *Presenting author

***Background and aims***: Unawareness of unhealthy diets has been emphasized as a potential barrier to the promotion of healthier diets. The aim of this study was to examine the extent to which Danish adults with unhealthy diets were unaware of this and to examine socio-demographic, health, and behavior characteristics associated with this unawareness.

***Methods***: The cross-sectional analysis included 3014 participants aged 18–75 years from the nationwide representative survey The Danish National Survey of Diet and Physical Activity 2011–2013. Data from 7-day pre-coded food diaries, structured interviews, and anthropometric measures were included. A diet index score was applied to divide individuals into tertiles of unhealthy, somewhat healthy, and healthy diets. The estimated diet healthiness was compared with individuals’ perceived diet healthiness (to a high degree healthy, to some degree healthy and not healthy). Being unaware of unhealthy diets was defined as being among the lowest tertiles “unhealthy diets” and perceiving own diets as healthy to a high degree (highly unaware) and to some degree (somewhat unaware). Logistic regression models were used to examine characteristics associated with unawareness of unhealthy diets.

***Results***: Among individuals with unhealthy diets, 13% perceived their diets as healthy to a high degree, 42% to some degree and 45% were aware of having unhealthy diets. Unawareness of unhealthy diets was associated with increasing age, excellent self-rated health, normal weight, and a moderate physical activity level.

***Conclusion***: Half of Danish adults with unhealthy diets were unaware of their unhealthy diets. This tendency was more likely among older adults and adults with positive indicators of health and health behaviors.

**Disclosure of Interest**: None to declare

### Poster presentation no. P469

#### Dietary behavior in early pregnancy and subsequent risk of preeclampsia – an observational study performed within the Norwegian Fit for Delivery study

##### Elisabet R. Hillesund^1^*, Elling Bere^1^, Linda R. Sagedal^2^, Ingvild Vistad^3^ and Nina C. Øverby^1^
^1^Department of Public Health, Sport and Nutrition, University of Agder, Kristiansand S, Norway; ^2^Department of Gynecology, Sorlandet Hospital HF, Kristiansand, Norway; ^3^Department of Gynecology, Sorlandet sykehus HF, Kristiansand, Norway *Presenting author

***Background and aims***: Preeclampsia is a serious complication of pregnancy and a leading cause of perinatal mortality and morbidity worldwide. Risk factors include a metabolic syndrome profile with endothelial dysfunction. Dietary factors may modify risk. In the present study, we aimed to investigate whether maternal dietary behavior in early pregnancy is associated with subsequent risk of preeclampsia.

***Methods***: 591 healthy nulliparous women participating in the randomized controlled Norwegian Fit for Delivery (NFFD) study were included in the analysis. Dietary behavior was operationalized as a diet score constructed from a 43-item food frequency questionnaire completed by participants in early pregnancy, before randomization into a lifestyle intervention. The score comprised 10 subscales addressing meal regularity, beverage consumption, fruit/vegetables, sweets and snacks, portion size, satiety, and awareness of food labeling. The diet score ranged from 0 to 10, with higher score indicating healthier dietary behavior. Participants were categorized as having “low” (0–3), “medium” (4–5), or “high” (6–10) diet score. Risk of preeclampsia with low vs. medium/high diet score was assessed by multivariate logistic regression, adjusted for maternal age, height, BMI, smoking status, education, cohabitant status, and randomization status.

***Results***: A total of 25/591 (4.2%) women developed preeclampsia. Of the 591 participants, 26%, 39%, and 35% were categorized with “low,” “medium,” or “high” diet score, respectively. Women with low diet score in early pregnancy had significantly higher adjusted risk of developing preeclampsia (OR_adj_: 2.99; 95% CI: 1.26–7.11) compared to women with medium/high diet score.

***Conclusion***: Women with low dietary behavior score in early pregnancy had higher risk of developing preeclampsia compared to women with higher scores in the NFFD study.

**Disclosure of Interest**: None to declare

### Poster presentation no. P470

#### Dietary habits of Swedish university students in nutrition science between 2001 and 2015

##### Sara Boman^1^, Maria Bergström^1*^, Anna Blücher^1^, Andreas Håkansson^2^ and Håkan Andersson^1^
^1^Chemistry & Biomedical Sciences, Linnaeus University, Kalmar, Sweden; ^2^School of Education and Environment, Food and Meal Science, Kristianstad University, Kristianstad, Sweden *Presenting author

***Background and aims***: While the Swedish nutrition recommendations have been kept relatively constant in recent years, public attitudes to different diets have been swinging faster. The National food survey (Riksmaten), being performed in Sweden only once per decade, cannot identify any corresponding rapid changes in diets. Hence, our understanding of potential fluctuations is limited. During the last 15 years, nutrition students at the Linnaeus University (formerly University of Kalmar) have reported their food intake in the context of the course *Diet, Nutrition and Health 7,5 hp*. The aim of the current study was to statistically evaluate these data for dietary trends.

***Methods***: Food intake was reported (by weighing or estimating the amounts) for two weekdays and one weekend day per student, along with age, length, sex, and weight. Food intake was translated to nutrient intake using Dietist Net software (Kost & Näringsdata).

***Results***: The result is an extensive data set comprising more than 1100 individuals and more than 2500 days of food intake reports. Admittedly, the data set has some validity problems: the students differ from the Riksmaten study groups in mean age and geographical distribution, and all data were collected during March–April. Students in a nutrition course can also be expected to be more interested and more knowledgeable in the nutrition subject than an average person. Albeit these limitations, it is apparent that these data can be of interest as an indicator for national dietary trends.

***Conclusion***: The results clearly demonstrate a substantial change in nutrient intake from 2006 and onwards, where the energy from carbohydrates decreased from above 50% to below 40%, and where the energy intake from fat increased from about 25% to 36%. Further details, such as the effects on the intake of selected micronutrients, will be presented.

**Disclosure of Interest**: None to declare

### Poster presentation no. P471

#### Socioeconomic status and food habits

##### Irene Mattisson* Department of Risk and Benefit Assessment, National Food Agency, Uppsala, Sweden *Presenting author

***Background and aims***: The association between health and socioeconomic status (SES) is well-known. People with a low SES have higher burden of disease compared to people with high SES. Food habits influence the risk of non-communicable diseases. Therefor it is important to consider the association between SES and food habits in public health work. The aim was to compile scientific literature on the association between SES and food habits in Sweden from 2000 until now.

***Methods***: Based on a structured literature search relevant articles were identified. Also studies with other Nordic countries were included as well as multicenter studies that included Sweden as a center.

***Results***: Many different indicators of SES were used; the most used were length of education, occupational level, income and wealth. Food habits were assessed with different methods and classified after reported food stuffs, calculated intakes of nutrients and/or biomarkers of nutritional status.

A positive association between SES and healthy food habits was found. Education had the strongest association in many of the studies. There were gender differences, women eat healthier than men and this difference can be seen already during adolescents. The effect of migration is more difficult to interpret; the only two studies found from Sweden had diverging results. Food costs affect habits; those with the lowest income ate more of foods high in energy but low in nutrients and less of nutritious foods like vegetables and fish.

No Swedish study had examined the effect of the found differences in food habits across SES groups on risk of disease. Very few studies had analyzed the association between biomarkers and SES. Since biomarkers data does not build on self-reported data they are important complement when studying the association between food habits and SES.

***Conclusion***: The association between food habits and SES might explain some of the differences found in health outcome between different SES groups.

**Disclosure of Interest**: None to declare

### Poster presentation no. P472

#### Riksmaten FLEX- modern technology to assess diet in Sweden

##### Anna K. Lindroos*, Eva W. Lemming, Ninna Lundberg-Hallén, Marianne Arnemo and Ulla-Kaisa K. Hursti National Food Agency, Uppsala, Sweden *Presenting author

***Background and aims***: A major challenge when collecting dietary intake data is to have a valid method that is cost effective, easy to use and provides high quality data. A new web-based self-administered method, Riksmaten FLEX (RM-FLEX), has therefore been developed for the next Swedish national dietary survey in adolescents. Aim: to describe and assess the validity of RM_FLEX.

***Methods***: Face-to-face interviews to explore adolescents’ perceptions on foods and eating preceded the development RM_FLEX which was done in close collaboration between IT-developers and the dietary survey team. The method includes approximately 800 foods adapted from previous surveys, market statistics and adolescent interviews. The method works equally well on computers, tablets and smart phones. For the validation study adolescents in grades 5, 8 and 11 were recruited in Swedish schools Sept 2015-Feb 2016. Approx. 200 adolescents agreed to take part and report food intake 1) for three days in RM-FLEX (yesterday, today and one random day later) and 2) 2x24h recalls (one face-to-face interview and one later telephone interview) in random order. Participants also completed a questionnaire and wore accelerometers for 7 days for estimation of physical activity level. In addition blood samples were drawn for analyses of carotenoids, alkylresorcinols, and fatty acids, and weight and height were measured.

***Results***: Based on experiences from the study smaller adjustments have been made to the method. Tablets and smart phones worked best. The first 1 ½ days were satisfactory completed in class in presence of staff from the NFA. However, the third, randomly generated day, was not completed by all students. The main results from the validation study will be analyzed during the spring and presented for the first time in June 2016.

***Conclusion***: The development of RM-FLEX has been successful and well received in the target group. Improved routines for reminding the students of the third day are needed.

**Disclosure of Interest**: None to declare

### Poster presentation no. P473

#### Educational Material for promoting healthy food habits in all patient groups including those with mild cognitive impairment and communicative difficulties

##### Karin Kauppi^1^*, Louise Hjortenfalk^2^, Ingrid Blankenau^3^, Inger Stevén^4^ and Jenny Edin^3^
^1^Health Promoting Hospital, Uppsala University Hospital; ^2^Public Health Unit; ^3^Health and Habilitation; ^4^Storvreta Heath Center, Primary Care, Uppsala, Sweden *Presenting author

***Background and aims***: The Swedish National Board of Health and Welfare's National Guidelines for Methods of Preventing Disease recommends advanced counselling to patients with unhealthy eating habits. The intervention consists of multiple contacts and includes different aspects on healthy eating approached with a theory-based or structured dialogue. Changes in eating habits need to be individualized and measurable. There was no uniform clinical practice in Uppsala County Council for advanced counselling on eating habits. The aim was to develop an educational material for promoting healthy food habits in all patient groups including those with mild cognitive impairment and communicative difficulties, since they represent a vulnerable group.

***Methods***: A prototype material was tested by dietitians seeing patients with unhealthy eating habits on health centers, psychiatric units and rehabilitation. Dietitians and patients testing the material were asked about usability through an evaluation form. Two observations in patient calls and a structured interview with a dietitian were conducted. After evaluation the material was revised. Simple language and clear images was used to facilitate communication.

***Results***: The material includes twelve themes with facts, illustrations and homework assignments about healthy eating. The starting point is an agenda where the patient chooses areas that need improvement based on the approach of Motivational Interviewing. The assignments are supposed to encourage the patient to work out and maintain new habits and can be evaluated at return visits.

***Conclusion***: Health care can be more equal if the message is tailored for people that have the greatest needs. The expectation is that this material will contribute to patients to get access to information and hence improve their eating habits.

**Disclosure of Interest**: None to declare

### Poster presentation no. P474

#### Modulation of diet-induced-thermogenesis in response to protein and carbohydrate source - a randomized crossover study

##### Signe Nyby^1^, Lone V. Nielsen^2^*, Lars Klingenberg^2^, Christian Ritz^2^, Bjørn Liaset^3^, Karsten Kristiansen^1^, Lise Madsen^1,3^ and Anne Raben^2^
^1^Department of Biology; ^2^Department of Nutrition, Exercise and Sports, University of Copenhagen, Copenhagen, Denmark; ^3^National Institute of Nutrition and Seafood Research, Bergen, Norway *Presenting author

***Background and aims***: Proteins induce higher diet-induced-thermogenesis (DIT) than fat and carbohydrates. Proteins from different sources may affect DIT differently, and mouse studies have shown that the type and amount of carbohydrates also affect the thermic response of protein. We investigated the acute effects of fish protein versus meat-protein in combination with different glycemic index (GI) carbohydrates on DIT, respiratory quotient (RQ), glucose, insulin and C-peptide.

***Methods***: We included 25 healthy overweight men and women (mean±SD age: 28.8±7.6y, BMI: 27.5±1.5kg/m^2^). Four iso-caloric meals (Energy%: 41 carbohydrate, 34 fat, 25 protein) were tested; salmon+mashed potatoes, salmon+pasta, veal+mashed potatoes and veal+pasta. Energy expenditure was measured by indirect calorimetry at baseline and every half hour until an *ad libitum* buffet-style lunch was served 3.5 hours later. Blood samples were drawn at baseline and every 20 minutes.

***Results***: We found a higher DIT after salmon+mashed potatoes compared to salmon+pasta (p=0.001) and veal+pasta (p=0.049), respectively. RQ was higher after mashed potatoes compared to pasta, independent of protein. Glucose peaks were lower after salmon compared to veal in combination with the same carbohydrates. This was reflected in lower peaks of insulin and C-peptide for salmon compared with veal, dependent of carbohydrate.

***Conclusion***: Salmon+mashed potatoes increased DIT compared to the other meals. This result indicates that DIT is sensitive to GI of carbohydrates after intake of salmon protein, but not from veal. The salmon protein seemed to induce lower peaks of glucose, insulin and C-peptide compared to veal protein dependent of carbohydrate source.

**Disclosure of Interest**: None to declare. The study was founded by the Norwegian Seafood research fund (FINS 900842).

### Poster presentation no. P475

#### Variation in nutrient composition of Norwegian beef meat

##### Marije Oostindjer^1^, Bjørg Egelandsdal^1^, Marion Haugen^1^, Pia Kjelsaas^1^, Lene R. Lima^1^, Frøydis Bjerke^2^, Trine Thorkildsen^3^, Kristin Saarem^4^, Anna Haug^5^ and Ellen-Margrethe Hovland^2^* ^1^Department of Chemistry, Biotechnology and Food Science, Norwegian University of Life Sciences, Ås, Norway; ^2^Animalia – the Norwegian Meat and Poultry Research Centre, Oslo, Norway; ^3^MatPrat – Opplysningskontoret for egg og kjøtt, Oslo, Norway; ^4^Nortura SA, Oslo, Norway; ^5^Department of Animal and Aquacultural Sciences, Norwegian University of Life Sciences, Ås, Norway *Presenting author

***Background and aims***: Consumption of red and processed (red) meat in Norway is high compared to the global average, but due to a gradual decrease since 2008 the intake is now just slightly above the maximum amount recommended in the dietary guidelines. In order to identify the role of beef meat in a healthy diet, nutrient composition and variation of nutrients in Norwegian beef meat (2013–2015) was investigated and compared with a previous comprehensive composition analysis (Matvaretabellen (MVT) 2014), rather than the less-comprehensive MVT 2015.

***Methods***: Seventy-two beef samples from Norwegian Red Cattle of various ages and genders were collected over a period of 25 months (2013–2015), from nine regions that represented the Norwegian beef production in terms of production volume and landscapes. Samples for analyses were cut from each carcass on day 4–6 postmortem, minced, standardized to 14% fat content, packed, and frozen at −80°C on day 10–11 postmortem.

***Results***: Among the vitamins and minerals most relevant for human health, significant higher levels of vitamin B_12_ (2.9 vs 1.0 µg/100 g), zinc (4.8 vs 3.8 mg/100 g), and nominally higher values of selenium (8.0 vs 7.0 µg/100 g) were found in the samples compared to MVT2014. Iron levels were slightly higher, while niacin, vitamin B_6_, and phosphor levels were similar to MVT2014. The average hemin content was 0.15 mg/ml (not in MVT2014).

Of these nutrients, iron and vitamin B_12_ showed the largest variation between samples (standard deviation>20% of average).

***Conclusion***: This study presents updated and a more representative nutrient composition of Norwegian beef meat compared to MTV 2014. The cause of variation in vitamin B_12_ is not well documented, and the higher level of iron may be due to the use of averaged bull and cow values. The study has identified possible directions for healthier beef meat obtainable through feeding. As an example, selenium content might be increased.

**Disclosure of Interest**: None to declare

### Poster presentation no. P476

#### “Are nuts goodies?” – planned relapses and the problematics of indulging in weight management

##### Kaija Rautavirta^1*^, Johanna Mäkelä^1^ and Mari Niva^2^
^1^Department of Teacher Education, University of Helsinki, Helsinki, Finland; ^2^Department of Political and Economic Studies, Consumer Society Research Centre, University of Helsinki, Helsinki, Finland *Presenting author

***Background and aims***: Recent years have witnessed an emergence of new e-tools for weight loss, such as online services providing detailed calorie counting in food diaries. In this paper, we examine the relationship between the ideal of a balanced diet promoted by an online weight loss service and the discourses of slimming brought by their users. We focus on the ways in which striving for moderation, including what we call “moderate indulgement” are discussed. In particular, we analyze the ways in which indulgence is justified, conditioned, and forbidden, and the practical solutions that users have established in order to carry out moderation.

***Methods***: We examine dieters’ postings on the discussion forum of a Finnish online weight-loss service “Kilo Club” (www.kiloklubi). The data cover the postings from thread “More than 30 kg to lose” which consists of 1476 pages from 2013 to 2014. The data were analyzed by qualitative content analysis.

***Results***: One of the principles in Kilo Club is that indulgence, having treats, is allowed in moderation. Our preliminary findings suggest that learning how to have treats in a controlled manner was one of most significant problems that the discussants had faced. In some accounts, allowing treats was justified by describing as prize. In some cases, it was regarded best to ban all treats. However, it was also conditioned by allowing it as “planned relapses.” These practical solutions show that the discussants applied the ideals advanced by the service in a creative manner, adapting them to their personal life situations.

***Conclusion***: The findings suggest that the ways in which users negotiate the meanings of indulgence either support or contradict the basic principles of the service. It is a widely shared feature in many diets to totally eliminate indulgence, and our findings show that the repudiation of this view in Kilo Club evokes a diversity of viewpoints and practical solutions.

**Disclosure of Interest**: None to declare

### Poster presentation no. P477

#### Effects of a diet containing water-soluble fish proteins from lean fish on serum cholesterol levels and blood pressure in obese Zucker rats

##### Aslaug Drotningsvik^1^*, Linn A. Vikøren^1^, Ola Flesland^2^ and Oddrun A. Gudbrandsen^1^
^1^Department of Clinical Medicine, University of Bergen, Bergen, Norway; ^2^Vedde AS, Triple Nine Group, Bergen, Norway *Presenting author

***Background and aims***: With the increasing prevalence of obesity, dietary intervention strategies targeting obesity-related metabolic complications such as high cholesterol levels and hypertension are important. Fish consumption is associated with reduced risk of common life-style diseases, and studies in humans and rats have shown beneficial effects of fish proteins on lipid metabolism. Thus, it is interesting to further explore the potential beneficial effects of fish proteins on the metabolic complications seen in a well-established rat model of human obesity. In the present study, we wanted to investigate the effect of water-soluble proteins from lean fish on lipid metabolism and blood pressure in mildly hypertensive, obese Zucker rats.

***Methods***: Twelve obese Zucker fa/fa rats were assigned to either intervention group or control group. The intervention group were fed a diet with 1/3 of the total protein content from water-soluble fish proteins from lean fish and the remaining 2/3 of protein from casein. The control group received casein as the sole protein source. The intervention period lasted 5 weeks. Lipids were measured in fasting serum taken at time of euthanization. Blood pressure was measured at baseline and endpoint.

***Results***: Rats fed water-soluble fish proteins from lean fish had significantly lower serum total cholesterol, HDL cholesterol, and LDL cholesterol concentrations when compared to the control group. The rise in blood pressure (systolic and diastolic) from baseline to endpoint was significantly lower in the intervention group compared to the control group.

***Conclusion***: Water-soluble fish proteins from lean fish affected both serum cholesterol levels and blood pressure in obese Zucker rats and should be further investigated as a potential tool in dietary treatment of obesity-related complications.

**Disclosure of Interest**: A. Drotningsvik: None to declare; L. A. Vikøren: None to declare; O. Flesland: Triple Nine Group is a producer of fish meal; O. A. Gudbrandsen: None to declare

### Poster presentation no. P478

#### Effects of salmon filet intake on blood pressure regulation

##### Linn A. Vikøren^1^, Aslaug Drotningsvik^1^, Iselin Vildmyren^1^, Øyvind Eng^2^, Jutta Dierkes^1^, Gunnar Mellgren^3^ and Oddrun A. Gudbrandsen^1^* ^1^Department of Clinical Medicine, University of Bergen, Bergen, Norway; ^2^Hormone Laboratory, Haukeland University Hospital, Bergen, Norway; ^3^Department of Clinical Science, University of Bergen, Bergen, Norway *Presenting author

***Background and aims***: High blood pressure is a common co-morbidity of obesity, and low fish intake may be a risk factor for developing hypertension. Also, dietary fish protein seems to delay increase in blood pressure in spontaneous hypertensive rats as they age. The aim of the project was to investigate the effects of salmon intake on blood pressure and markers of kidney function in humans and rats with increased risk of developing hypertension due to overweight or obesity.

***Methods***: Healthy adults with BMI>28 kg/m^2^ (*N*=137) and obese, mildly hypertensive male Zucker fa/fa rats (*N*=30) received either salmon or a control diet devoid of fish for 12 and 4 weeks, respectively. The intervention group in the clinical study received 450 g/week of salmon to be implemented in three of their weekly dinners (each with 150 g fish filet). Rats in the intervention group received 25% of their proteins from salmon filet and 75% from casein, whereas the control group received casein as their sole source of protein.

***Results***: In overweight adults, salmon intake resulted in a significantly lower diastolic blood pressure with no change in systolic blood pressure. In obese rats, both systolic and diastolic blood pressure was lower after salmon intake when compared to the control group. Body weight and energy intake remained unchanged throughout the intervention period in all groups, both in the clinical study and in the rat study. In rats, the albumin/creatinine ratio in urine was significantly lower after salmon intake.

***Conclusion***: Consumption of salmon was associated with lower blood pressure in overweight humans and rats without affecting body weight, and may protect against development of kidney damage in obese mildly hypertensive rats.

**Disclosure of Interest**: None to declare

### Poster presentation no. P479

#### Effects of intake of baked and raw fish on serum lipids and fatty acid composition in obese rats

##### Marthe T. Bergseth^1^*, Aslaug Drotningsvik^1^, Linn A. Vikøren^2^, Svein A. Mjøs^3^, Gunnar Mellgren^4^ and Oddrun A. Gudbrandsen^1^
^1^Department of Clinical Medicine, University of Bergen, Bergen, Norway; ^2^Department of Clinical Nutrition, University of Bergen, Bergen, Norway; ^3^Department of Chemistry, University of Bergen, Bergen, Norway; ^4^Department of Clinical Science, University of Bergen, Bergen, Norway *Presenting author

***Background and aims***: Several investigators have shown an interest in the health effects of fish oil and fish protein, where studies in humans and rats suggest that both fish oil and protein may improve lipid metabolism. Less is known whether baked or raw fish poses any difference in relation to benefits on lipid metabolism and health. Attention has been drawn to the increasing consumption of raw fish, sushi, and it is proposed that the large consumption of sushi in the Japanese population is the reason for their low rates of heart disease. We wanted to compare effects of intake of baked and raw fish filet in obese rats on serum lipids and fatty acid composition in serum.

***Methods***: Eighteen male Zucker fa/fa rats were assigned to three experimental groups and fed modified AIN-93G diets for 4 weeks, where 25% of casein was substituted with protein from baked or raw salmon filet, whereas the control group received casein as the only protein source.

***Results***: Rats fed baked salmon had significantly lower serum concentrations of total cholesterol, HDL cholesterol, and LDL cholesterol and higher concentrations of triacylglycerol when compared to control rats. Rats fed raw salmon had significantly lower serum concentrations of HDL cholesterol when compared to controls but did not affect other serum lipids. Rats fed baked or raw salmon had higher concentrations of long-chain n-3 fatty acids in serum when compared to controls.

***Conclusion***: Serum lipids seem to be more affected by baked salmon than raw salmon.

**Disclosure of Interest**: None to declare

### Poster presentation no. P480

#### Advanced dietary counselling improves eating habits in patients with severe depression

##### Lolita Mörk^1^*, Susanne Fredén^1^ and Karin Kauppi^2^
^1^Division of Psychiatry, Uppsala University Hospital, Uppsala, Sweden; ^2^Health Promoting Hospital, Uppsala University Hospital, Uppsala, Sweden *Presenting author

***Background and aims***: In 2012, the Swedish National Board of Health and Welfare published National Guidelines for Methods of Preventing Disease due to unhealthy lifestyles.

The Department of Psychiatry at Uppsala University Hospital has engaged in systematic efforts to promote healthy lifestyles and diet among its patients. In this group, advanced dietary counselling may be an effective method to improve eating habits.

***Methods***: We screened 203 severely depressed men and women at the psychiatric outpatient clinic using a validated food index, and identified 67 (33%) with “unhealthy” eating habits. Thirty five (52%) of those with sub-optimal diet accepted an offer to receive between two and ten advanced diet consultations with a registered dietitian. To date, 32 of the treated patients have been re-screened 6–12 months after the dietary consultations using the same food index.

***Results***: Of the 32 patients for whom we obtained follow-up data, 26 (81%) had improved their eating habits over the study period, whereas 6 (19%) had the same or poorer diet. Eighty-one percent of the patients reached an index in the “healthy” eating range. The patients with the poorest diet at baseline showed the greatest improvement over time, whereas patients with less severe dietary scores showed more modest dietary improvements.

***Conclusion***: It is important to use systematic screening methods such as the validated food index to identify patients with poor eating habits. Eating habits in patients with severe depressions can improve by offering advanced dietary counseling by a registered dietitian.

**Disclosure of Interest**: None to declare

### Poster presentation no. P481

#### Dietary intake among adults and children in the Nordic countries – development from 2011 to 2014

##### Sisse Fagt^1^*, Holmfridur Thorgeirsdottir^2^, Helene B. Enghardt^3^, Ellen Trolle^1^, Lene F. Andersen^4^, Katja Borodulin^5^ and Jeppe Matthiessen^1^
^1^National Food Institute, Copenhagen, Denmark; ^2^Directorate of Health, Reykjavik, Iceland; ^3^National Food Administration, Uppsala, Sweden; ^4^Division of Nutritional Epidemiology, University of Oslo, Oslo, Norway; ^5^THL, Helsinki, Finland *Presenting author

***Background and aims***: In the “Nordic Plan of Action for better health through diet and physical activity” (2006), it was decided to monitor diet, physical activity, and overweight in the Nordic countries. In 2007–2010, the monitoring system was developed and validated. Two data collections were carried out in 2011 and 2014. In this study, the situation in 2014 and development from 2011 to 2014 are reported.

***Methods***: A total of 17,775 adults aged 18–65 years and 4,958 children aged 7–12 years participated in the Nordic monitoring system in 2011 and 2014. The dietary assessment method used is a short Food Frequency Questionnaire covering fruits and vegetables, wholegrain bread, fish, foods rich in saturated fat and added sugar. The intake frequencies of these foods are used to score the diet quality and to estimate the proportion having an unhealthy diet, a medium healthy diet, and a healthy diet.

***Results***: From 2011 to 2014, the proportion of high consumers of sugar-rich foods decreased and consumption of vegetables increased among adults and children. Fish consumption increased also among children. The negative findings among adults were an increase between 2011 and 2014 in the proportion of high consumers of foods rich in saturated fat and a decrease in the mean frequency intake of fish. In addition, wholegrain bread decreased among adults and children.

These findings implied that the overall dietary quality decreased among adults, and the proportion with an unhealthy diet increased from 2011 to 2014 (18.2% vs. 21.5%). The proportion with an unhealthy diet did not change in the child population between 2011 and 2014 (15.4% vs. 15.6%).

***Conclusion***: The diet has become less healthy from 2011 to 2014 among adults in the Nordic countries, but has not changed among children. Hence, the overall diet has not improved in adults and children in the Nordic countries.

**Disclosure of Interest**: None to declare.

### Poster presentation no. P482

#### Effects of water soluble and water insoluble cod proteins on lipid metabolism in overweight adults

##### Iselin Vildmyren^1^*, John Cao^1^, Ida U. Karlsen^1^, Lina B. Haug^1^, Øyvin Eng^2^, Friedemann G. Erchinger^1^, Gunnar Mellgren^3^, Alfred Halstensen^3^ and Oddrun A. Gudbrandsen^1^
^1^Department of Clinical Medicine, University of Bergen, Bergen, Norway; ^2^Hormone Laboratory, Haukeland University Hospital, Bergen, Norway; ^3^Department of Clinical Science, University of Bergen, Bergen, Norway *Presenting author

***Background and aims***: Overweight and obesity are strongly related to the development of lifestyle diseases, such as hypercholesterolemia, which consequently can lead to development of cardiovascular diseases. Studies in rats have shown that dietary fish protein may decrease blood cholesterol by stimulating cholesterol and bile elimination through the enterohepatic circulation. The effects of fish protein intake in humans have been scarcely investigated. The aim of this study was to examine the effects of cod press cake meal (water insoluble proteins) or a mixture of cod press cake meal and cod stickwater (water soluble proteins) on lipid metabolism in overweight adults.

***Methods***: In total, 55 adults with BMI>28 kg/m^2^ were randomized to three experimental groups, and ingested tablets containing cod press cake meal (PC), tablets containing a mixture of cod press cake meal and stickwater (PC+SW) or placebo tablets (control) daily for 8 weeks. The amino acid intake from the intervention tablets was 6 g/day. Blood and feces were collected at baseline and after 8 weeks.

***Results***: Fasting serum total cholesterol, HDL cholesterol, and LDL cholesterol concentrations were not affected by PC, PC+SW, or placebo. Fasting and postprandial non-esterified fatty acids (NEFA) concentrations were significantly reduced, and the HDL/LDL ratio in fasting serum was significantly increased after PC intake, but these parameters were not affected by PC+SW or in the placebo group. Fecal excretion of cholesterol and bile acids was not affected in any of the groups.

***Conclusion***: Water insoluble cod proteins (PC) beneficially affected serum HDL/LDL cholesterol ratio and fasting and postprandial NEFA concentrations, whereas the mixture of water soluble and water insoluble cod proteins (PC+SW) did not affect serum lipids. Dietary supplementations with water insoluble cod protein may improve lipid metabolism in overweight adults.

**Disclosure of Interest**: I. Vildmyren: None to declare; J. Cao: None to declare; I. U. Karlsen: None to declare; L. B. Haug: None to declare; Ø. Eng: None to declare; F. G. Erchinger: None to declare; G. Mellgren: None to declare; A. Halstensen: Shareholder in Halstensen Granit, a company producing fish meal; O. A. Gudbrandsen: None to declare.

### Poster presentation no. P483

#### Modeling of vitamin D intake using data on habitual intake and adding four fortified food items, used in Danish RCT: ODIN FOOD

##### Ida Grønborg*, Inge Tetens, Majken Ege, Tue Christensen and Rikke Andersen Research Group for Risk-Benefit, Danish Technical University, Copenhagen, Denmark *Presenting author

***Background and aims***: In Denmark, the dietary intake of vitamin D is low. At the population level, food fortification may be an effective strategy to increase the intake.

We aimed to model the distribution of vitamin D intake in the Danish adult women using data on the habitual dietary intake of vitamin D (HD) with and without fish. Ensuring that no participant reached the UL of 100 µg/day.

***Methods***: We extracted a sub-sample of the Danish dietary survey population (2011–2013) consisting of women 18–50 years (*n*=855). Based on the women's HD, we modeled four scenarios. The daily contribution from the four fortified foods were: crisp bread 8 µg, yoghurt 2.25 µg, cheese 7 µg, and egg 3 µg.

***Results***: Table 1 shows the total vitamin D intake and the distribution in the calculated scenarios. The result suggests that for a group of women not consuming fish, fortified foods containing above levels, provide a median intake around 22 µg/day. Also, the result suggests that even in the scenario with a habitual diet including fish, adding fortified foods and a supplement, the intake of the 99th percentile does not exceed 50 µg/day.

Scenario 2 has been used to estimate the vitamin D intake in a randomised controlled trial (RCT) in immigrant and ethnic Danish women, part of the European vitamin D project, ODIN.

***Conclusion***: The scenarios estimating vitamin D intake and distribution in Danish women show that no women will be at risk of exceeding the UL by eating a diet including fish, the mentioned fortified foods, and a daily supplement of 10 µg.

**Abstracts P483–Table 1:** Total vitamin D intake in percentiles in Danish women (*n*=855) using four scenarios (µg/day)

Model Per-centiles	0. HD	1. HD without fish	2. HD without fish+4 fortified foods	3. HD without fish+4 fortified foods+supplement	4. HD including fish+4 fortified foods+supplements
5	1,0	0,7	21,0	31,0	31,3
25	1,7	1,1	21,4	31,4	32,0
50	2,6	1,5	21,7	31,7	32,8
75	4,5	1,9	22,1	32,1	34,7
95	11,1	2,6	22,8	32,8	41,3
99	17,7	3,3	23,6	33,6	47,9

**Disclosure of Interest**: None to declare.

### Poster presentation no. P485

#### Perspectives about health outcomes related to food among Nordic children

##### Linda Berggren^1^*, Sanna Talvia^2^, Eldbjørg Fossgard^3^, Unnur B. Arnfjörð^4^, Agneta Hörnell^1^, Anna Ólafsdóttir^4^, Ingibjörg Gunnarsdóttir^5^, Hege Wergedahl^3^, Hanna Lagström^6^, Maria Waling^1^ and Cecilia Olsson^1^
^1^Department of Food and Nutrition, Umeå University, Umeå, Sweden; ^2^Child and Youth Research Institute, Turku, Finland; ^3^Faculty of Education, Bergen University College, Bergen, Norway; ^4^School of Education, University of Iceland, Reykjavik, Iceland; ^5^Unit for Nutrition Research, National University Hospital of Iceland, Reykjavik, Iceland; ^6^Turku Institute of Child and Youth Research, University of Turku, Turku, Finland *Presenting author

***Background and aims***: Dietary intake in school has previously been studied but little is known about Nordic children's perspectives on food healthiness in the school lunch context. This study aims to explore 10-year-old Nordic children's perspectives on outcomes of healthy eating in the school lunch context.

***Methods***: Seventy-two focus groups were conducted in Sweden, Finland, Norway, and Iceland with a total of 423 participants. A flexible topic guide and 14 preselected photos displaying different school lunch contexts were used as stimuli material. Interviews were transcribed and analyzed using thematic analysis.

***Results***: Children reasoned that school lunch are and should be healthy since the food eaten at school has short- and long-term outcomes related to cognitive and physical health. It was commonly expressed that food eaten in school affects school work and functioning in learning activities. It was also stated that food eaten in school can have negative and positive effects on your mood, for example, eating unhealthy food or an insufficient amount of food, puts you in a bad mood which can affect the rest of the school day. The discussions mainly relied on negative short-term effects such as feeling ill and reduced stamina. Some food and food groups such as vegetables, milk, and fish, were mentioned in a more positive sense highlighting the positive short- and long-term outcomes on health. When describing the long-term outcomes of eating, children mentioned that healthy eating helps to build muscles, grow, and prevent diseases, such as cancer and diabetes. Sugar and fat were frequently mentioned as being the cause of overweight and some other diseases.

***Conclusion***: In general, Nordic children have an adequate understanding of established relations between food and health. Yet, we know that many pupils do not eat according to recommendations. This highlights the importance of taking the complexity of food choice into consideration in nutritional education.

**Disclosure of Interest**: None to declare.

### Poster presentation no. P486

#### Socio-economic differences in fruit and vegetable consumption – results from the Swedish national public health survey, 2004–2015

##### Marita Friberg* and Anna Jansson The Public Health Agency of Sweden, Solna, Sweden *Presenting author

***Background and aims***: Regular consumption of fruit and vegetables promotes health and is associated with reduced risk of chronic diseases. The reported intake of fruit and vegetables has, however, remained largely unchanged over the last decade in Sweden. Socio-economic factors are main predictors of fruit and vegetables consumption. In the current study, we used national survey data to evaluate socio-economic differences in fruit and vegetable intake.

***Methods***: Data were derived from the Swedish national public health survey “Health on equal terms” carried out during 2004–2015. Socio-economic factors (education, occupation, and employment) in relation to fruit and vegetable intakes were examined among adult women and men (30–64 years). Separate analyses were conducted for reported fruit and vegetable intakes.

***Results***: More than 80% of the women reported that they consumed fruit and vegetables at least twice a day, the corresponding proportion for men was 30%. Women and men in sparsely populated areas reported a low intake of fruit and vegetables to a larger extent than nationally. Fruit and vegetable intake varied by socio-economic factors, but the gradient was more obvious for vegetables than for fruit. The results also show that socio-economic factors are stronger predictors of fruit and vegetable intake in men than in women. Men in white-collar occupations were 2–4 times more likely to report a higher intake of fruit and vegetables compared to men in blue-collar occupations.

***Conclusion***: There are a clear socioeconomic gradient in fruit and vegetable consumption. The findings also suggest different patterns for fruit and vegetables, as well as for men and women.

**Disclosure of Interest**: None to declare.

### Poster presentation no. P487

#### Dietary intake among 12-month-old Norwegian–Somali and Norwegian–Iraqi infants: the InnBaKost study

##### Navnit K. Grewal^1^*, Lene F. Andersen^2^, Cathrine S. Kolve^1^, Ingrid Kverndalen^1^ and Liv E. Torheim^1^
^1^Department of Nursing and Health Promotion, Faculty of Health Sciences, Oslo and Akershus University College, Oslo, Norway; ^2^Department of Nutrition, Institute of Basic Medical Sciences, University of Oslo, Oslo, Norway *Presenting author

***Background and aims***: In Norway, data on the diet of infants of immigrant background are scarce. The aim of the present study was to describe the dietary intake among 12-month-old Norwegian-Somali and Norwegian-Iraqi infants.

***Methods***: A cross-sectional survey was performed in 2013–2014. We targeted women born in Somalia or Iraq residing in Eastern Norway who had 12-month old children for inclusion. The final sample consisted of 89 mothers of Somali and 77 mothers of Iraqi origin. Data on the infants’ dietary intake were collected through two 24-h multiple-pass recalls conducted face-to-face.

***Results***: In total, 40% of the Norwegian–Somali (NS) infants and 47% of the Norwegian–Iraqi (NI) infants were breastfed (*P=*0.414). Median energy percentages (E%) from protein, fat, and carbohydrates were within the recommended intake ranges, except for saturated fats (12–13%). Median E% from added sugar was low (3%). Median intakes of almost all micronutrients were above the recommended daily intakes. Vegetables were consumed by 95%. Cow's milk was consumed by 71% of NS infants and 64% of NI infants. NS infants had significantly higher median daily intakes (g/day) of commercial infant cereals, commercial fruit purees, fish/fish products, yoghurt, and cow's milk. NI infants had significantly higher daily intakes of white bread, cake, meat/meat products, eggs, fruits, and berries, added sugar, and tea.

***Conclusion***: The findings indicate that the dietary intake of infants of Somali and Iraqi origin in general is in accordance with the Norwegian dietary recommendations.

**Disclosure of Interest**: None to declare.

### Poster presentation no. P489

#### Changes in dietary habits following initiation of a health promoting high school project

##### Anna S. Olafsdottir* Sport and Health Sciences, School of Education, University of Iceland, Reykjavik, Iceland *Presenting author

***Background and aims***: Adolescence is considered a vulnerable period regarding the development of healthy diet behaviors. Teenagers tend to choose foods low in valuable nutrients such as fast food and soda at the expense of healthier foods, especially fruit and vegetables. With this in mind, the aim of this study was to investigate trends in consumption of soda and fruit and vegetables during a 4 year starting period of the holistic health promoting high school project initiated by the Directorate of Health in Iceland. The project is based on four main themes; nutrition, physical activity, mental health, and lifestyles.

***Methods***: Participants were 426 students from two high schools; one initiating the project in 2010 and the other 2012. Measurements were performed these years and a third time in 2014. Semi-quantitative food frequency questionnaires were used to assess dietary habits alongside an extensive assessment of diverse health parameters and behaviors.

***Results***: Overall findings showed a decrease in total soda consumption for both genders over time. Boys decreased their consumption on average from 2.4 to 1.6 L/week over the study period and girls from 1.6 to 1.1 L/week. During that same time, total fruit and vegetable consumption deceased among boys (2.1 vs. 1.6 portions/day from 2010 to 2014), but an increase was seen among girls (1.6 vs. 2.0 portions/day). Similar patterns were seen between time points of measurements for both schools, with changes being most at the initiation of the project in each school.

***Conclusion***: It is possible that changes in dietary habits are strongest during the phase where the focus is put on nutrition and fade when other topics take over in this holistic health behavioral approach. Especially this may apply when choosing fruit and vegetables whereas it may be easier to keep new habits around soda consumption since these were removed from the school setting.

**Disclosure of Interest**: None to declare.

### Poster presentation no. P490

#### BMI change in early school years as a predictor of restrained eating in preadolescence

##### Lena Hansson* and Ting Jia Department of Public Health Sciences, Karolinska Institutet, Stockholm, Sweden *Presenting author

***Background and aims***: Few studies have used longitudinal data to investigate if childhood BMI is a risk factor for developing eating and social behavior problems. The aim of the present study was to assess if change in standardized body mass index (zBMI) during early school years is associated with eating and social behavior in preadolescence and whether there are gender differences in the associations.

***Methods***: The sample consisted of 725 girls and 780 boys born in 1988 and 1989 in Sweden. Repeated measures of height and weight between 7 and 10 years of age were retrospectively extracted from school health records. Children self-reported restrained eating, emotional eating, and external eating (Dutch Eating Behavior Questionnaire) as well as conduct behavior, close friends, and social acceptance (Harter Self-perception Profile for Children) in 2000 when the children were approximately 10–12 years of age. Parents self-reported on similar scales, their educational level, height, and weight.

***Results***: Multiple regression analysis, adjusted for children's initial BMI and age, showed that restrained eating was significantly predicted by change in zBMI in both boys and girls (β=0.12, respectively, β=0.08, *p*<0.05). Additional adjustments for parental BMI, parental education, and parental eating behavior attenuated the associations somewhat. However, for girls the relationship was no longer statistically significant. The association between changes in zBMI and the other eating behaviors as well as social behaviors did not reach statistical significance.

***Conclusion***: Early positive BMI change was found to predict higher restrained eating. These findings have public health implications in relation to prevention of overweight and disordered eating later in life.

**Disclosure of Interest**: None to declare.

### Poster presentation no. P491

#### Evaluation of plasma biomarkers of dietary fat quality: long-term reproducibility and correlations between lipid fractions

##### Matti Marklund^1,^*, Lars Lind^2^ and Ulf Risérus^1^
^1^Public health and caring sciences, Uppsala, Sweden; ^2^Medical sciences, Uppsala University, Uppsala, Sweden *Presenting author

***Background and aims***: Fatty acid (FA) composition in blood compartments is used as biomarkers of dietary fat quality, but compositions may differ between compartments. As most cohorts use single FA measurements to reflect long-term intake, reproducibility data of plasma FAs are warranted. Here, we aimed to investigate intercorrelations of FA compositions in plasma phospholipids (PP) and cholesterol esters (CE), and to evaluate 10-year reproducibility of FA composition in plasma CE.

***Methods***: Composition of FA in PP and CE was assessed in a population-based cohort of 70-year-old Swedes (*n*=940). In a subsample (*n*=539), FA was also measured in CE at ages 75 and 80 years. All FA measurements were performed by gas chromatography, and Spearman's rank correlation coefficients were calculated between proportions of FA measured in plasma PP and CE collected at age 70 years. Plasma CE compositions at ages 70, 75, and 80 years were utilized to assess 10-year reproducibility by calculating intraclass correlation coefficients (ICC) in one-way random-effect models.

***Results***: Moderate to strong correlations (*r*≥0.68) between PP and CE were observed for most FA, except for palmitic acid and stearic acid. Fair to good reproducibility (ICC=0.39–0.70) was observed for most FA, and was greatest for palmitoleic acid and all measured n-6 polyunsaturated FA. Poor reproducibility (ICC<0.40) was observed for α-linoleic acid, pentadecanoic acid, and stearic acid.

***Conclusion***: Overall, FA measured in plasma PP and CE correlated strongly, suggesting that FA composition in these compartments generally can be used interchangeably as biomarkers in epidemiological studies. Overall, moderate reproducibility of plasma FA indicates that single measurements fairly well can estimate average FA levels over a 10-year period.

**Disclosure of interest**: None to declare.

### Poster presentation no. P492

#### Sufficient iodine status in Norwegian adolescents

##### Katina D. Handeland^1,2^*, Jannike Øyen^1^, Karoline L. Espelid^1^, Matilde Odland^1^, Siv Skotheim^3^, Ingvild E. Graff ^1^, Marian Kjellevold^1^, Livar Frøyland^1^, Øyvind Lie^1^, Kjell M. Stormark^3^ and Lisbeth Dahl^1^
^1^Seafood safety and health, National Institute of Nutrition and Seafood Research (NIFES), Bergen, Norway; ^2^Department of Clinical medicine, Faculty of Medicine and Dentistry, University of Bergen, Bergen, Norway; ^3^Regional Centre for Child and Youth Mental Health, Uni Research Health, Bergen, Norway *Presenting author

***Background and aims***: Seafood, milk, and dairy products are the most important sources of iodine in the Norwegian diet. Iodine is an essential constituent of thyroid hormones, and adequate iodine intake during adolescence is of importance since thyroid hormones are required for brain development. The recommended intake of iodine is 150 µg per day for children from 10 years of age, adolescents, and adults. Urinary iodine concentration (UIC) is a good marker of recent iodine intake, and UIC of school-aged children can be used to define a population's iodine status. Levels in the range of 100–199 µg/L indicate adequate iodine intake. The aim of this study was to evaluate iodine status at baseline among adolescents participating in an intervention study (*n*=481).

***Methods***: Adolescents (14–15 years) from eight different schools in Bergen, Norway completed a web-based, semi-quantitative food frequency questionnaire (FFQ) and spot urine samples were collected at the different schools in February 2015. UIC (*n*=300) was determined using inductively coupled plasma spectrometry (ICP-MS).

***Results***: Median UIC was 110 µg/L (range 8–440 µg/L). According to the WHO criteria for assessment of iodine status in a population, 1.7% had severe and 6.3% had moderate iodine deficiency, 29.3% had mild iodine deficiency, 49.3% had optimal iodine status, 10.7% had more than adequate intake, and 2.7% had excessive iodine intake. Data from the FFQ revealed that approximately 42% ate seafood for dinner 2–3 times per week or more and 67% reported a daily consumption of one portion or more of milk and dairy products.

***Conclusion***: Preliminary findings in the present study classify the adolescents as iodine sufficient. FFQ data indicate that 42% had a seafood intake of 2–3 times per week or more. This study suggests that a regular intake of seafood and dairy products is important dietary sources ensuring iodine sufficiency in adolescents.

**Disclosure of interest**: None to declare.

### Poster presentation no. P493

#### Preferences and attitudes with regards to food habits and meal patterns among older adults

##### Amanda Lindblad^1^*, Elisabet Rothenberg^2^, Karin Wendin^2^ and Synneve Dahlin-Ivanoff ^3^
^1^Internal Medicine and Clinical Nutrition, Sahlgrenska Academy, University of Gothenburg, Gothenburg, Sweden; ^2^Kristianstad University Sweden, Kristianstad, Sweden; ^3^Department of health and rehabilitation, Sahlgrenska Academy, University of Gothenburg, Gothenburg, Sweden *Presenting author

***Background and aims***: Longevity increases and older adults range from newly retired to those who passed the centenarian mark. High age poses challenges but also opportunities for society and entrepreneurship. The project Active Ageing – Individualized Meal Solutions for Health and Quality of Life is collaboration between universities, research institutes, food industry, and public sector intended to create a cohesive chain from order, packaging, distribution to safe deliveries and proper waste management, financed by VINNOVA. Aim was to study attitudes, needs, and preferences regarding food habits and meal patterns among older adults living in their own homes.

***Methods***: Participants were recruited from “Elderly Persons in the Risk Zone” 2008–2011 including 459 community-dwelling men and women ≥80 years (Gustafsson et al., 2012). In 2014, a subsample of 221 individuals was invited to answer a questionnaire focusing practice, knowledge, and attitudes regarding food habits and meal pattern. The Regional Ethical Review board in Gothenburg ref nr approved the study: ref nr 650-07, T231-14.

***Results***: 164 answered, ♀73%. Mean age 90.6±2.84 (87–100) years. 123 lived alone ♀86% ♂45% (*p*<0.01). Mean BMI 23.7±3.70. Meal habits were stable with distinct gender differences, women taking a greater responsibility. ♀60 and ♂67% had no medical difficulties. ♀63 and ♂61% would not consider home delivered convenience meals, and ♀87% and ♂78% did not need help when cooking. Top three important practicalities when buying or receiving convenience meals were easy-open package (♀72% ♂70%), easy to get food out (♀38% ♂39%), and easily read (♀35% ♂48%).

***Conclusion***: In this age group, females take a higher responsibility in acquiring and preparing food. A weakness is results based on self-reported data, while these data are less strenuous and entails a high response rate; there is no possibility for follow-up questions.

**Disclosure of interest**: None to declare.

### Poster presentation no. P494

#### Low-FODMAP rye bread helps IBS patients to get enough dietary fiber with reduced gastro-intestinal symptoms

##### Sanna-Maria Hongisto^1^*, Reijo Laatikainen^2^, Jussi Loponen^3^, Riitta Korpela^4^ and Jari Koskenpato^5^
^1^Fazer Bakeries Ltd, Lahti, Helsinki, Finland; ^2^Booston Ltd, Helsinki, Finland; ^3^Fazer Bakeries Ltd, Vantaa, Helsinki, Finland; ^4^Faculty of Medicine, Department of Pharmacology, University of Helsinki, Helsinki, Finland; ^5^Aava Medical Centre, Helsinki, Finland *Presenting author

***Background and aims***: The study evaluated if rye bread low in FODMAPs (Fermentable Oligo-, Di-, Monosaccharides and Polyols) is better tolerated than regular rye bread naturally high in FODMAPs in irritable bowel syndrome (IBS).

***Methods***: In this cross-over, double-blind controlled study patients (*n*=87) were randomized to control or low-FODMAP rye bread groups. Bread was consumed 210/240 grams daily. Four-day food records were collected. Symptoms were recorded by IBS symptom severity scoring (IBS-SSS) and VAS scale. Postprandial 6 h excretion of hydrogen was measured.

***Results***: Seventy three patients completed the study. Dietary intake was similar between the groups. Dietary fiber intake increased by 8 grams/day during the both study periods from baseline. Intake of FODMAPs from bread was 1.05–2.40 g/day lower during the low-FODMAP bread period. There were no significant differences in IBS-SSS between the two periods. VAS measurements of individual symptoms were different (table 1). There was also significant difference between the groups in the sum of weekly symptoms.

***Conclusion***: Low-FODMAP rye bread caused significantly less GI symptoms. Reduced hydrogen excretion with low-FODMAP bread confirms symptoms’ origination from the colonic fermentation. Low-FODMAP rye bread can be a part of low-FODMAP diet, and thus gives a healthy and tolerable means of increasing dietary fiber intake in IBS patients.

**Table 1:** IBS symptoms during the treatment periods

Symptoms (mean VAS, scale 0–100)	Low FODMAP bread	High FODMAP bread	*p*
Flatulence	47 (43–52)	52 (47–56)	0.04
Diarrhea	23 (19–27)	25 (21–30)	0.31
Constipation	24 (19–29)	25 (20–30)	0.66
Abdominal pain	34 (29–38)	38 (33–43)	0.049
Cramping	19 (16–23)	25 (20–29)	0.007
Borgorygmus	33 (28–37)	39 (34–44)	0.001
Dyspepsia	31 (25–36)	34 (29–40)	0.06
Sum of all GI symptoms	30 (27–33)	33 (30–37)	0.02

AUC of breath hydrogen was significantly lower during the low-FODMAP bread consumption (median 52.9 vs. 72.6; *p*=0.01).

**Disclosure of interest**: S.-M. Hongisto: Employee of Fazer Bakeries Ltd; R. Laatikainen: Grant from Fazer Bakeries Ltd.

### Poster presentation no. P495

#### Consumption of saturated fatty acids (SAFA) and the risk on coronary heart disease (CHD)

##### Thomas Sanders^1^*, Ingeborg I. A. Brouwer^2^, Johanna M. Geleijnse^3^ and Gerard Hornstra^4^
^1^Diabetes and Nutritional Sciences Division, King's College London, London, United Kingdom; ^2^Department of Health Sciences and the EMGO+, VU University, Amsterdam, Netherlands; ^3^Division of Human Nutrition, Wageningen University, Wageningen, Netherlands; ^4^Maastricht University (retired) and NUTRI-SEARCH, Gronsveld, Netherlands *Presenting author

***Background and aims***: Discussions on the health impact of dietary saturated fatty acids (SAFA) often suffer from preconceptions, limited expertise, and selective reasoning.

***Methods***: A scientific update is presented on the relation between SAFA intake and CHD risk.

***Results***: SAFA have important metabolic functions, but their consumption is not needed, as they can be synthesized *de novo*. However, because of their functional properties, SAFA are indispensable for the production of fat containing foods.

Replacement macronutrients in fat-modified diets are key to the observed health effects. Higher intakes of SAFA are not associated with higher CHD risks in studies that do not take replacement macronutrients into account. Little data exist on replacement of dietary SAFA by MUFA, but replacing SAFA by PUFA is associated with CHD risk reduction. This has been confirmed by randomized controlled trials. Cohort studies suggest that the food matrix and source of SAFA may also be of importance for their health effects.

One of the causal risk factors for CHD is the plasma low-density lipoprotein cholesterol (LDL-C) concentration. The common dietary SAFA mixture increases LDL-C as compared to mixed carbohydrates and unsaturated fatty acids (UFA). However, whether SAFA specifically affect the larger, less atherogenic LDL-C particles need further study.

As compared to carbohydrates, a higher SAFA intake increases plasma high-density lipoprotein cholesterol (HDL-C) concentrations, which in healthy populations is inversely related to CHD risk. However, a causal role of HDL-C in CHD is questioned, since interventions that increase HDL-C do not reduce CHD mortality in high-risk patients. In such patients, however, HDL-C may be dysfunctional. Therefore, these studies do not allow the rejection of HDL-C as a causal CHD risk factor.

***Conclusion***: The recommendation to replace butter and hard fats by soft margarine and vegetable oils can be considered beneficial for population health.

**Disclosure of interest**: None to declare.

### Poster presentation no. P496

#### Active elderly and their food habits and physical activity

##### Ida S. Grini* and Øydis Ueland Innovation, Consumer and Sensory Sciences, Nofima, Norwegian institute of Food, Fisheries and aquaculture Research, PB 210, 1431, Aas, Norway *Presenting author

***Background and aims***: When people retire today, they have better health than the previous generation. In addition, they are more informed about healthy foods and are more active. The objectives are to achieve a deeper understanding of elderly people's considerations about health (including physical activities) and food-related activities.

***Methods***: Fifty five active senior citizens in the eastern part of Norway were approached on senior bus travels and senior- and fitness centers and asked to fill in a short questionnaire on food habits. Based on the questionnaires, 15 respondents were recruited to an in-depth interview. Data were collected using a semi-structured interview guide.

***Results***: The active elderly were concerned with food, meals, and health. Dietary habits changed over time as the children move out, as they were diagnosed with medical conditions related to diet, or when they were widowed. For instance, when diagnosed with cardiovascular diseases or diabetes type 2, active elderly increased their purchase of fruit, vegetables, and fish. Medical conditions also led to a more conscious choice of products with lower levels of fat, sugar, and salt. However, the respondents were also intent on defending choices of foods that they believed they should have avoided, or eaten less of. They were apparently more worried about becoming overweight than they were of malnutrition. The informants were conscious about being physically active. For daily exercise, they went to the fitness centers, for a walk, or walked instead of driving to the store.

***Conclusion***: In addition to good taste, health aspects are important when choosing food. Active elderly are motivated to carry out physical activity on a regular basis: going for a walk, walking to the stores, or going to the gym. Social meeting places such as fitness- and senior centers may be good venues for communicating health information and promote lifestyle changes for active elderly.

**Disclosure of interest**: None to declare.

### Poster presentation no. P497

#### Interdisciplinary action on salt reduction for public health: the Norwegian salt partnership

##### Erik K. Arnesen^1,^*, Amandine Lamglait^2^ and Ida S. Grini^3^
^1^LHL (The Norwegian Heart & Lung Association), Ås, Norway; ^2^The Norwegian Directorate of Health, Oslo, Norway; ^3^Nofima – Norwegian institute of Food, Fisheries and aquaculture Research, Ås, Norway *Presenting author

***Background and aims***: A population-wide reduction in intake of salt (NaCl) is regarded as one of the “best buys” to reduce the incidence and burden of non-communicable diseases. Norwegian health authorities aim to lower the population's salt consumption to 30% by 2025. To stimulate action on salt reduction in food products and served meals, a national “salt partnership” was proposed. While dialogue with the food industry has been a part of Norwegian nutritional policy for a long time, no formal, committing partnership had previously been established.

***Methods***: Voluntary collaboration within the food industry requires a level playing-field. The Norwegian Directorate of Health invited a range of stakeholders to commit to an agreement on salt reduction in 2014. A collaboration with food and catering industries, trade organizations and associations, NGOs, and research institutions was established under leadership of the Directorate.

***Results***: Interdisciplinary task groups developed the cooperation agreement, plans for skills development, and salt reduction targets for about 100 product groups. This aims at reducing average salt content to 15% by 2018. The salt partnership was launched in 2015, and the cooperation agreement was signed by 53 actors (59 per February 2016). The targets will be revised in 2019.

Implementation of a common monitoring and reporting system, and public awareness campaigns, is the next step. Trends in urinary sodium excretion in the population will be one of the indicators.

***Conclusion***: Lowering the population salt intake is a shared responsibility and requires concerted, accountable work. The salt partnership is crucial to ensure a level playing-field and change current norms for salt. This public-private partnership is widely supported and is a promising initiative to gradually reduce the salt content of the food supply.

**Disclosure of interest**: None to declare.

### Poster presentation no. P498

#### Development of a food-nutrition-health research infrastructure

##### Inge Tetens^1^*, Paul Finglas^2^, Pieter van't Veer^3^ and Karin Zimmermann^4^ on behalf of the EuroDISH consortium ^1^National Food Institute, Technical University of Denmark, Søborg, Denmark; ^2^Institute of Food Research, Norwich Research Park, Norwich, United Kingdom; ^3^WU Agrotechnology & Food Sciences, Wageningen University, Netherlands; ^4^LEI Consument & Keten, LEI Wageningen UR, Wageningen, Netherlands *Presenting author

***Background and aims***: From a public health nutrition perspective, strategies and actions should be built on a scientific substantiated background. The aim of the present project was to assess the needs for a common roadmap and to develop a conceptual framework for a research infrastructure on food related to nutrition and health.

***Methods***: In the FP7 EuroDISH research project, the needs for food-nutrition-health research infrastructures were assessed from the perspective of a “DISH” model consisting of:

- Determinants of dietary behavior – finding out why we choose what we eat and drink
- Intake of foods and nutrients – assessing and evaluating how much we eat and drink
- Status and function of the body – using markers of body stores, biomolecular mechanisms, nutritional health
- Health and disease – assessing the links between nutrition and health outcomes (diseases, quality of life, ageing, fertility).

The project combined knowledge from expert consultations, mapped existing European (including Nordic) research infrastructures and identified gaps, needs, and governance issues. The findings were formulated as recommendations for a roadmap, integrated and developed into a conceptual design of a research infrastructure.

***Results***: The conceptual design consisted of the four DISH pillars in the food, nutrition, and health domain with cross-cutting multidisciplinary research level of study DATA, research on innovative TOOLS, and food and health SERVICES approaches (see figure).

***Conclusion***: The proposed Food-Nutrition-Health Research Infrastructure was developed as an overarching, virtual, integrated, open access research infrastructure to collate, validate, harmonize and connect existing, and future research data for the benefit of researchers, policy makers, industry, and societal organizations in the food-nutrition-health domain.

**Figure:**
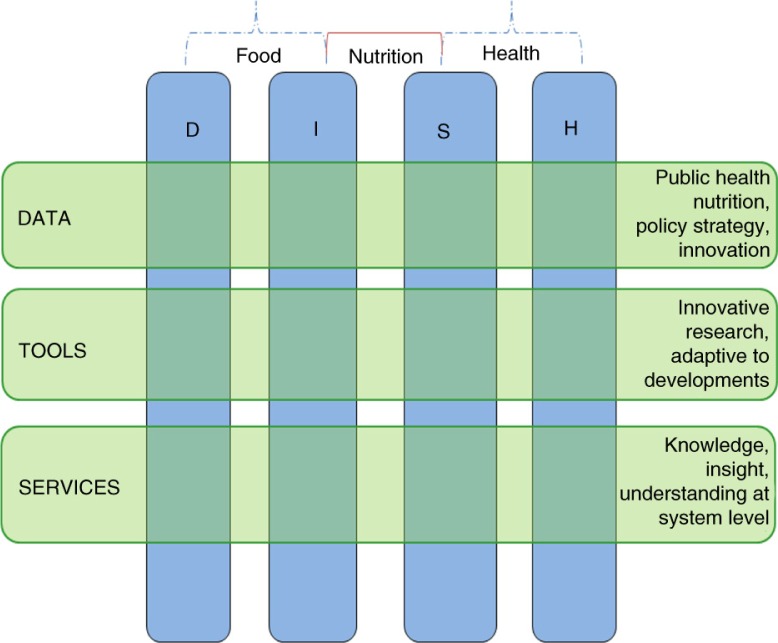


**Disclosure of interest**: None to declare.

### Poster presentation no. P499

#### Short-term effects of chewing gum on satiety and afternoon snack intake in healthy weight and obese women

##### Eunyoung Park^1^, Indika Edirisinghe^1^, Sophie Miquel-Kergoat^2^*, Mike Kelley^2^, Taichi Inui^2^ and Britt Burton-Freeman^1^
^1^Center for nutrition research, Institute for Food Safety and Health- IIT, Chicago, United States; ^2^Foundational Science, Global Innovation Center, Chicago, United States *Presenting author

***Background and aims***: Afternoon snacking contributes significantly to total energy intake. Strategies to enhance the satiety value of lunch and reduce afternoon snacking are of interest for body weight management.

***Objectives***: To assess whether between-meal gum chewing would enhance the satiety response to a fixed lunch meal; to assess the role of cholecystokinin (CCK) as a potential mediator of the response in non-obese healthy weight and obese women.

***Methods***: Fifty unrestrained obese (*n*=25) and non-obese healthy weight (*n*=25) women participated in a two-arm cross-over study assessing multiple (15 min per hour X 3 h) gum chewing (GUM) occurrences or no gum (Control) on subjective ratings of satiety, subsequent sweet and salty snack intake, CCK, and general metabolic responses.

***Results***: GUM compared to Control resulted in significant suppression of hunger, desire to eat, and prospective consumption (*p*<0.05). Total snack energy intake was reduced ~9.3% by GUM but not significantly different from Control (*p*=0.08). However, overall carbohydrate intake was reduced by GUM (*p*=0.03). This was consistent with a reduction in snacks characterized as high carbohydrate, low fat (*p*=0.02). BMI specific effects indicated that GUM reduced pretzel intake in obese women (*p*=0.05) and Oreo cookie intake in healthy weight women (*p*=0.03) 3 hours after lunch. Metabolic responses and CCK did not differ between experimental conditions.

***Conclusion***: Chewing gum intermittently post-lunch enhances perceptions of satiety and may have important implications in reducing afternoon high carbohydrate-snack intake.

**Disclosure of interest**: Study funded by Wrigley (Mars Inc.).

### Poster presentation no. P500

#### Identifying a dietary index to predict a better preserved cognitive function

##### Behnaz Shakersain^1^*, Debora Rizzuto^1^, Susanna C. Larsson^2^, Gerd Faxén-Irving^3^, Laura Fratiglioni^1^ and Weili Xu^1^
^1^Aging Research Center, Department of Neurobiology, Care Sciences and Society, Karolinska Institutet, Stockholm, Sweden; ^2^Division of Nutritional Epidemiology, National Institute of Environmental Medicine, Karolinska Institutet, Stockholm, Sweden; ^3^Karolinska University Hospital, Huddinge, Division of Clinical Nutrition, Department of Neurobiology, Care Sciences and Society, Karolinska Institutet, Karolinska Institutet, Stockholm, Sweden *Presenting author

***Background and aims***: The ideal dietary patterns for preserved cognitive function remain unknown. We aimed to examine the association of a-priori healthy dietary indices with cognitive decline, and to identify a dietary pattern for better preserved cognitive function in the Swedish older adults.

***Methods***: Within a population-based cohort study of the Swedish older adults aged 60+, 2,223 dementia-free participants with Mini-Mental State Examination (MMSE) score ≥27 were identified at baseline, and followed for 6 years. Diet was assessed using a semi-quantitative food frequency questionnaire. MMSE was tested at baseline and follow-ups. Based on a priori knowledge obtained from our previous study on diet-cognition association, the Prudent Diet index (PDI) was developed. Dietary index scores were also calculated for the alternative Mediterranean-DASH Intervention for Neurodegenerative Delay (a-MIND), Mediterranean Diet (MedDietScore), Dietary Approach to Stop Hypertension (DASH), and Baltic Sea Diet (BSD). Each index score was categorized into tertiles indicating low, moderate, and high adherence levels. Data were analyzed using Mixed-effects models.

***Results***: In multi-adjusted models, the middle (β: 0.139, 95% CI: 0.077 to 0.201) and highest tertiles (β: 0.238, 95% CI: 0.175 to 0.300) of the PDI score, and the middle (β: 0.075, 95% CI: 0.012 to 0.138) and highest tertiles (β: 0.126, 95% CI: 0.064 to 0.188) of the a-MIND score, were associated with slower MMSE decline versus lowest tertile. However, only the highest tertile of MedDietScore (β: 0.099, 95% CI: 0.036 to 0.163) was significantly related to less MMSE decline. Receiver operating characteristic curves showed largest area under the curve for the highest tertile of the PDI.

***Conclusion***: Moderate-to-high adherence to Prudent Diet is associated with a better preserved cognitive function compared to other diet indices in the Swedish older adults.

**Disclosure of interest**: None to declare.

### Poster presentation no. P501

#### Women with elevated cholesterol during early pregnancy have offspring with elevated low-density lipoprotein cholesterol concentration at the age of 6–13 years

##### Jacob J. Christensen^1,2^*, Kjetil Retterstøl^1,2^, Kristin Godang^3,4^, Marie C. P. Roland^4,5^, Elisabeth Qvigstad^3^, Jens Bollerslev^3,4^, Thor Ueland^4,6^, Tore Henriksen^4,5^ and Kirsten B. Holven^1,7^
^1^Department of Nutrition, University of Oslo, Oslo, Norway; ^2^The Lipid Clinic; ^3^Department of Endocrinology, Oslo University Hospital, Oslo, Norway; ^4^University of Oslo, Oslo, Norway; ^5^Department of Obstetrics, Oslo University Hospital, Oslo, Norway; ^6^Research Institute for Internal Medicine, Oslo University Hospital, Oslo, Norway; ^7^Norwegian National Advisory Unit on Familial Hypercholesterolemia, Oslo University Hospital, Oslo, Norway *Presenting author

***Background***: Cardiovascular disease (CVD) starts to develop in early life, potentially even *in utero*. A vast amount of data shows that maternal obesity, dysglycemia, diabetes, and undernutrition prior to and during pregnancy associates with offspring CVD risk. However, associations between maternal gestational hypercholesterolemia and offspring CVD risk have scarcely been studied.

***Objective***: To investigate the associations between elevated maternal gestational low-density lipoprotein cholesterol concentration (LDL-C) and CVD risk factors in 6–13 year old offspring.

***Design***: We recruited and examined 6–13 year old children whose mothers participated in the Stork pregnancy cohort, and who had either hypercholesterolemia or hypocholesterolemia during pregnancy, defined as LDL-C over the 90th percentile or below the 10th percentile within the Stork cohort, respectively. We measured CVD risk factors in the children, including body composition, blood pressure, plasma lipids, glucose concentration, and total fatty acid composition, as well as dietary intake.

***Results***: Maternal plasma LDL-C at gestational week 14–16 was 4.0 and 1.4 mmol/L in the hypercholesterolemic (*n*=27) and hypocholesterolemic (*n*=34) groups, respectively (*p*<0.001). Interestingly, offspring plasma LDL-C was 0.4 mmol/L higher in children whose mothers had hypercholesterolemia during early pregnancy (*p*<0.01). There was no difference in birthweight in the two groups, and no other clinical or biochemical CVD risk factors or dietary intake whereas different between the groups at 6–13 years.

***Conclusions***: Women with elevated cholesterol during early pregnancy have offspring with elevated LDL-C at the age of 6–13 years. Promoting a cholesterol-lowering healthy lifestyle among young fertile women with hypercholesterolemia may be particularly beneficial in offering CVD protection – not only for the individual woman, but also for children of the subsequent generation.

**Disclosure of interest**: J. J. Christensen: None to declare; K. Retterstøl: Abbot, Apotek 1, Amgen, Genzyme, Melk.no, Mills DA, MSD, The Norwegian Medical Association, The Norwegian Directorate for Health, Oslo Economics; Pfizer, Sanofi, The Norwegian Medicines Agency; none of which are related to the contents of this manuscript; K. Godang: None to declare; M. C. P. Roland: None to declare; E. Qvigstad: Lilly and MSD; none of which are related to the contents of this manuscript; J. Bollerslev: None to declare; T. Ueland: None to declare; T. Henriksen: None to declare; K. B. Holven: Tine DA, Mills DA, Olympic Seafood, Amgen, Sanofi and Pronova; none of which are related to the contents of this manuscript.

